# Isovolumic relaxation strain imaging is an accurate and sensitive approach for detection of active diastolic dysfunction: A preclinical study

**DOI:** 10.1002/ame2.70147

**Published:** 2026-02-28

**Authors:** Jingjing Liang, Juncheng Wang, Jun Cheng, Yanggan Wang

**Affiliations:** ^1^ Department of Geriatrics General Medical Center, Zhongnan Hospital of Wuhan University Wuhan China; ^2^ Medical Research Institute of Wuhan University Wuhan China; ^3^ Frontier Science Center for Immunology and Metabolism, Wuhan University Wuhan China

**Keywords:** active diastolic dysfunction, heart failure, heart failure with preserved ejection fraction, isovolumic relaxation, strain imaging

## Abstract

**Background:**

Currently, there is no imaging‐based method for directly detecting active diastolic dysfunction. This study aimed to evaluate the efficacy of isovolumic relaxation strain imaging (IVSI) in assessing active diastolic dysfunction in preclinical settings.

**Methods:**

Active diastolic function was assessed in C57BL/6J mice subjected to transverse aortic constriction (TAC) or a sham operation using daily conventional echocardiography and strain imaging over a 14‐day period, with follow‐ups at weeks 4 and 8. A modified approach was developed to accurately identify the isovolumic relaxation time (IVRT) using an apical three‐chamber view.

**Results:**

The novel imaging protocol successfully identified IVRT in each mouse. TAC mice exhibited significant alterations in E/A and E/E′ ratios from day 12 to 14, while the average strain rate detected by IVSI showed a significant decrease from day 4. After 8 weeks, TAC mice developed severe heart failure with reduced ejection fraction, but conventional echocardiography failed to detect diastolic dysfunction.

**Conclusion:**

IVSI continued to indicate obvious diastolic alterations. Together, these data suggested that IVSI is an effective imaging‐based method for direct detection of active diastolic dysfunction with high accuracy and sensitivity.

## Graphical abstract

A modified approach was developed to accurately identify the isovolumetric relaxation time (IVRT) using an apical three‐chamber view. Compared with conventional echocardiography, isovolumic relaxation strain imaging (IVSI) helps to detect the earlier alternations of diastolic function in transverse aortic constriction (TAC) mice. The minimum acceleration of the average strain rate during the isovolumic relaxation time serves as a promising indicator for the early diastolic disfunction in TAC mice. IVSI is an effective imaging‐based method for direct detection of active diastolic dysfunction with high accuracy and sensitivity.

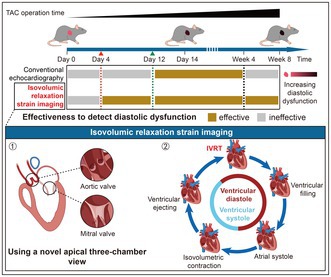

## 1 INTRODUCTION

Diastolic dysfunction, characterized by impaired ventricular filling during the relaxation phase, typically exists in the left ventricle.^1^, ^2^ This condition emerges earlier than systolic dysfunction, which often requires substantial myocardial injury or hypertrophy progression for clinical manifestation.^3^, ^4^, ^5^ The pathophysiology distinguishes two diastolic components: active relaxation during early diastole and passive compliance in later phases. Active relaxation, an energy‐dependent process initiated in isovolumic relaxation, relies on efficient sarcoplasmic Ca^2+^ reuptake to facilitate myofilament dissociation and rapid pressure decline. In contrast, passive compliance reflects extracellular matrix mechanics without energy expenditure.^6^, ^7^, ^8^ Notably, subtle cardiomyocyte impairment or hypertrophy in early HF preferentially disrupts active relaxation mechanisms, positioning its evaluation as a potential diagnostic marker for incipient diastolic dysfunction.^9^, ^10^ However, accurate methods to measure active diastolic capacity remain scarce in both preclinical and clinical settings, especially in cardiac imaging research.

Pulse wave Doppler echocardiography is currently the standard method for diagnosing diastolic dysfunction, yet its hemodynamic indices (early diastolic transmitral flow velocity [E]/peak early‐diastolic annular velocity [E′] and E/late diastolic transmitral flow velocity [A] ratios) demonstrate critical limitations in reflecting intrinsic myocardial status.^11^ These limitations become particularly pronounced in end‐stage cardiac remodeling where structural adaptations induce paradoxical “pseudonormalization” patterns. Specifically, significant ventricular dilation alters myocardial compliance and reduces transmitral pressure gradients, causing proportional decreases in both early diastolic mitral inflow velocity and annular tissue velocity, a phenomenon that leads to non‐significant E/E′ ratios despite advanced disease. Concurrent neurohormonal overactivation and chronic atrial stretch further confound interpretation by attenuating atrial contraction, thereby normalizing E/A ratios.^12^ Such pathophysiological masking underscores the necessity for novel diagnostic modalities that directly assess active diastolic capacity, bypassing the confounding effects of structural remodeling.

Speckle‐tracking echocardiography (STE), commonly called strain imaging, is a high‐resolution modality quantifying myocardial deformation throughout the cardiac cycle and provides superior functional assessment compared to conventional echocardiography.^13^, ^14^, ^15^ Previous studies have demonstrated that strain imaging significantly surpasses conventional metrics, such as left ventricular ejection fraction (LVEF), in assessing systolic dysfunction.^16^, ^17^ However, the superiority of strain imaging over traditional indicators, such as E/A and E/E′, in detecting diastolic dysfunction has not been well explored. This study introduces isovolumic relaxation strain imaging (IVSI), a novel STE‐derived technique enabling simultaneous aortic/mitral valve tracking for isovolumic relaxation time (IVRT) quantification, and cycle‐phase‐specific analysis of early diastolic mechanics. We validated IVSI's efficacy in a 8‐week longitudinal study comparing transverse aortic constriction (TAC) and sham‐operated C57BL/6J mice, demonstrating its enhanced sensitivity in detecting diastolic dysfunction relative to conventional parameters.

## 2 MATERIALS AND METHODS

### 2.1 TAC operation

All animal studies were conducted in accordance with the guidelines of the Animal Care and Use Committee of Wuhan University, approved by the Animal Care and Use Committee of the Laboratory Animal Center, Medical Research Institute of Wuhan University (approval no. MRI2021‐LAC18). Pressure‐overload cardiac hypertrophy and HF were induced by TAC, as previously described.^18^ Sixty adult male C57BL/6J mice (6–8 weeks old) were randomly divided into sham‐operated and TAC groups at a 1:1 ratio. Twenty mice underwent non‐invasive imaging, and forty others were used for invasive pressure‐volume (PV) loop analysis. TAC was performed using a 28‐gauge needle placed on the aortic arch between the innominate and left common carotid arteries, which was then ligated with 7‐0 prolene. After removing the needle, the aortic arch was constricted, and differential bilateral carotid pulsatility was checked to confirm successful banding. The sham‐operated group underwent a similar procedure without ligation.

### 2.2 Conventional echocardiography

Transthoracic echocardiography was performed daily for 14 consecutive days and at weeks 4 and 8 post‐surgery. To mitigate the potential influence of daily anesthesia on cardiac function, a standardized maintenance protocol was applied. Isoflurane concentration was titrated to 1.0%–1.5% (administered with 1 L/min O_2_ gas) to maintain a target heart rate (HR) of 590–610/400–450 bpm across examinations. Each imaging session was limited to 15–20 min to reduce cumulative anesthetic exposure, particularly concerning for diastolic function. Core body temperature was maintained at 37 ± 0.5°C using a heating pad with rectal probe feedback. The sham‐operated group underwent the same anesthesia protocol, enabling controlled comparisons. Although anesthesia may affect myocardial relaxation, the consistent methodology and between‐group comparisons help minimize its confounding impact on the assessment of diastolic dysfunction by IVSI. M‐mode imaging was performed immediately after anesthesia induction when the heart rate was relatively high (approximately 590–610 bpm). Subsequently, as anesthesia stabilized and the heart rate decreased to the target range of 400–450 bpm, PW Doppler and other detailed measurements were acquired. This protocol ensured both the capture of stable images and physiological relevance during detailed functional assessments.

Echocardiographic parameters were acquired using the Visualsonics Vevo 2100 system with a 30‐MHz probe. Conventional echocardiographic systolic parameters were measured digitally from M‐mode images in short‐axis views, including left ventricular (LV) internal dimensions at end‐diastole and end‐systole (LVIDd, LVIDs), and the anterior and posterior wall thicknesses at end‐diastole and end‐systole (LVAWd, LVAWs, LVPWd, LVPWs). HR was also recorded. Diastolic parameters were measured using pulsed wave Doppler and tissue Doppler in the apical four‐chamber views, including E, A, E′, IVRT, isovolumic contraction time (IVCT), ejection time (ET), left atrial area (LAA), and HR. Calculated parameters included ejection fraction (EF), fractional shortening (FS), LV volume at end‐diastole and end‐systole (LV Vold/Vols), E/E′ ratio and E/A ratio.

### 2.3 Strain imaging based on speckle tracking

Strain imaging analysis was conducted using the VevoStrain software (version 3.2.6) on ultrasound B‐Mode images, based on 2D wall motion tracking algorithms. This software and its underlying algorithm have been validated for the use in murine models, providing reliable assessment of myocardial deformation.^19^, ^20^, ^21^ The software tracks the motion of the endocardium and epicardium, providing detailed profiles of velocity, displacement, strain, and strain rate. Cardiac cycles were selected between two respiration cycles, and an initial contour was defined on the B‐Mode images. Strain rate and velocity were calculated as the rate of distance change and strain with respect to time, respectively, and peak values were identified using the software. In cardiac mechanics, positive and negative values represent opposing directional changes in myocardial motion: contraction during systole generates positive values, while relaxation in diastole produces negative values. For curve smoothing, the raw strain data were filtered using a low‐pass Butterworth filter with a cutoff frequency of 20 Hz to reduce noise while preserving physiological signals. The filter order was set to 2, and the processing was implemented in the VevoStrain software. Throughout all imaging sessions, core body temperature was monitored using a rectal probe and maintained at 37 ± 0.5°C with a heated imaging platform, in accordance with established guidelines for murine cardiovascular phenotyping.^19^, ^22^

To ensure the reproducibility of segmentation and tracking, intra‐observer and inter‐observer variability were assessed. Two independent observers (JL and JW) performed the segmentation and tracking on a subset of images (*n* = 10) at two different time points. Intraclass correlation coefficient (ICC) and Bland–Altman analysis were used to evaluate the agreement. The ICC values for strain parameters were above 0.90 for both intra‐observer (ICC = 0.95, 95% CI = 0.91–0.98) and inter‐observer (ICC = 0.92, 95% CI = 0.87–0.96) assessments, indicating excellent reproducibility. Bland–Altman plots showed minimal bias with limits of agreement within ±5% for strain measurements.

### 2.4 Apical three‐chamber view for isovolumic diastolic function

The novel apical three‐chamber view was obtained by rotating the standard four‐chamber view approximately 90° counterclockwise. This view clearly visualizes the left ventricle, left atrium, aorta, mitral valve, and partial aortic valve. IVRT was defined as the period from aortic valve closure to mitral valve opening.

### 2.5 PV loop analyses

Invasive PV loop analyses were performed on postoperative days 4 and 7 in mice following TAC to assess diastolic function. Mice anesthetized with 1.0%–1.5% isoflurane. Following tracheotomy, the animals were mechanically ventilated using a rodent piston ventilator. The Mikro‐Tip PV catheter was advanced into the left ventricle via the pre‐pierced apex to acquire real‐time PV loops. Systolic and diastolic performance were evaluated by calculating the maximum rate of left ventricular pressure rise (max) and decline (min), respectively, as the first derivatives of the ventricular pressure trajectory. The relaxation time constant (Tau, ms) was calculated using Weiss method in LabChart software. Steady‐state PV loops were recorded and averaged over 10–15 consecutive cardiac cycles to ensure reproducibility. Data analysis was conducted using LabChart software, in which volumetric calibration was based on cuvette calibration and which incorporated parallel conductance compensation via hypertonic saline injection to enhance accuracy.

## 3 STATISTICAL ANALYSES

Statistical analyses were conducted using R software (version 4.3.0). Welch's corrected *t*‐tests were employed to compare cardiac features between sham‐operated and TAC mice. Repeated measures data were analyzed with linear mixed‐effects models (LMM). The model included the experimental group and postoperative period as fixed effects. The lme4 package was used for model fitting, and the lmerTest package was applied to obtain *p* values using Satterthwaite's approximation for degrees of freedom. To account for multiple comparisons, *p* values were adjusted for the false discovery rate (FDR) via the p.adjust function in R. Statistical significance was considered when *p* < 0.05. A priori power analysis was conducted to determine the minimum sample size required for this study. Based on pilot experiment data (*n* = 4 per group), the minimal acceleration of radial strain rate, identified as the primary outcome measure, showed a mean of 0.022 (SD = 0.017) in the TAC group and 0.056 (SD = 0.023) in the sham‐operated group, corresponding to a large effect size (Cohen's *d* = 1.68). Using GPower software (version 3.1.9.7) with an alpha level of 0.05 and a desired power of 0.80 for a two‐tailed independent *t*‐test, the analysis indicated that a minimum sample size of *n* = 6 per group was required to detect this effect. However, to account for potential attrition during the 8‐week longitudinal study and enhance the robustness of our findings, we increased the sample size to *n* = 10 per group for the main imaging cohort.

## 4 RESULTS

### 4.1 Development of IVSI for evaluating active diastolic dysfunction

**FIGURE 1:**
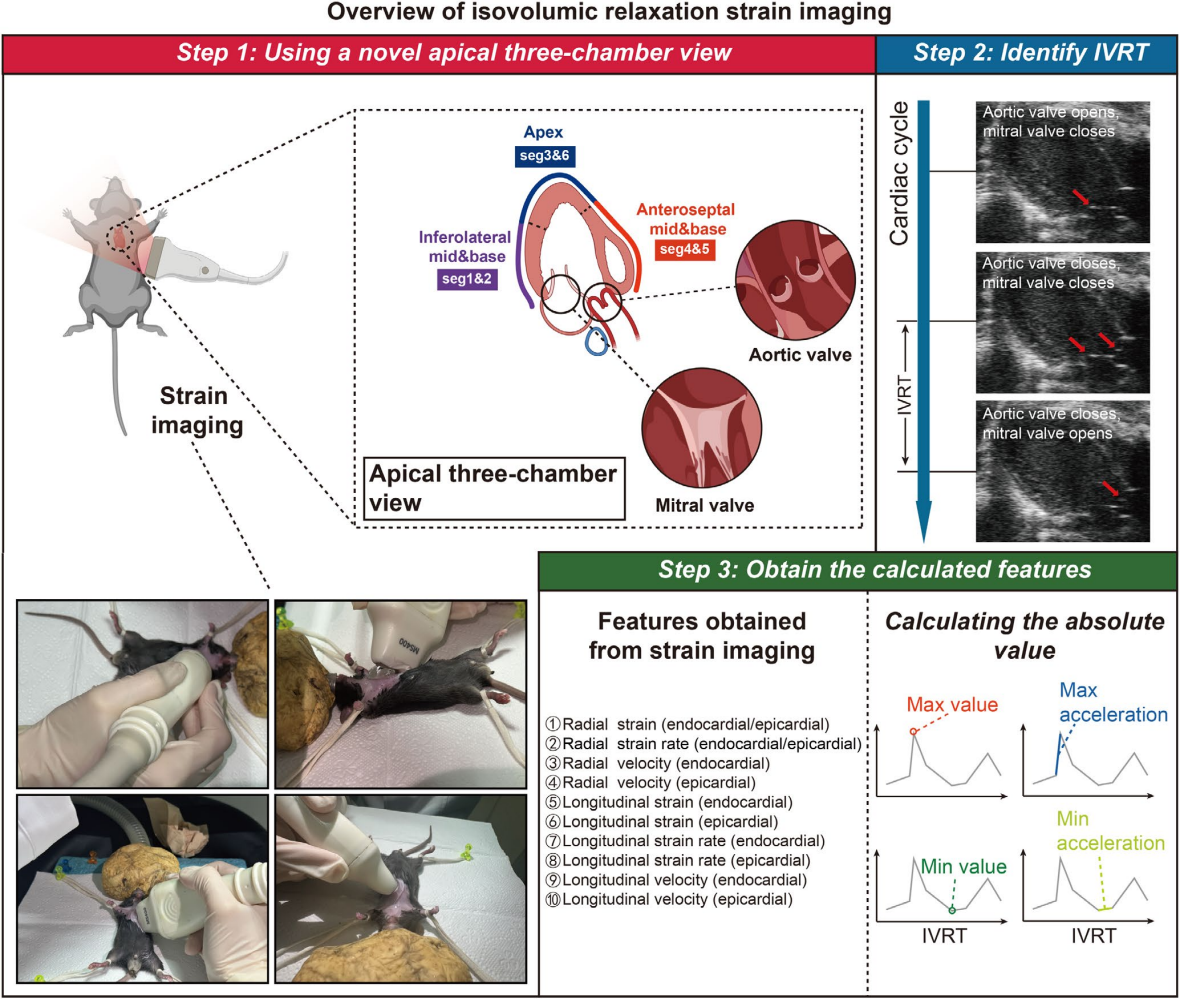
Schematic overview of the IVSI methodology. The standard four‐chamber view was rotated approximately 90° counterclockwise to obtain a modified apical three‐chamber view. From this derived view, the IVRT was measured, and strain‐derived parameters were acquired. A custom R script was developed to process the strain data, automatically extracting values and calculating peak strain, minimum strain, maximum acceleration, and minimum acceleration to ensure reproducibility of IVSI measurements. IVRT, isovolumic relaxation time; IVSI, isovolumic relaxation strain imaging.

To assess active diastolic dysfunction in mice, we developed a novel IVSI technique based on strain imaging. This approach utilized a modified apical three‐chamber view, obtained by rotating the standard four‐chamber view approximately 90° counterclockwise. This optimized view clearly visualized the left ventricle, left atrium, aorta, mitral valve, and aortic valve simultaneously. Within this view, the IVRT was precisely defined as the interval from aortic valve closure to mitral valve opening for each cardiac cycle. Strain imaging analyses during the IVRT were conducted. Key strain imaging parameters, including strain, strain rate and velocity, were extracted from the original strain imaging acquisition files, and their maximum and minimum values as well as accelerations were calculated using the R software (Figure 1). The R code for data extraction and processing is included in Supporting Information 1 to ensure transparency and reproducibility of the IVSI.

### 4.2 Detection of diastolic dysfunction by conventional echocardiography

**FIGURE 2:**
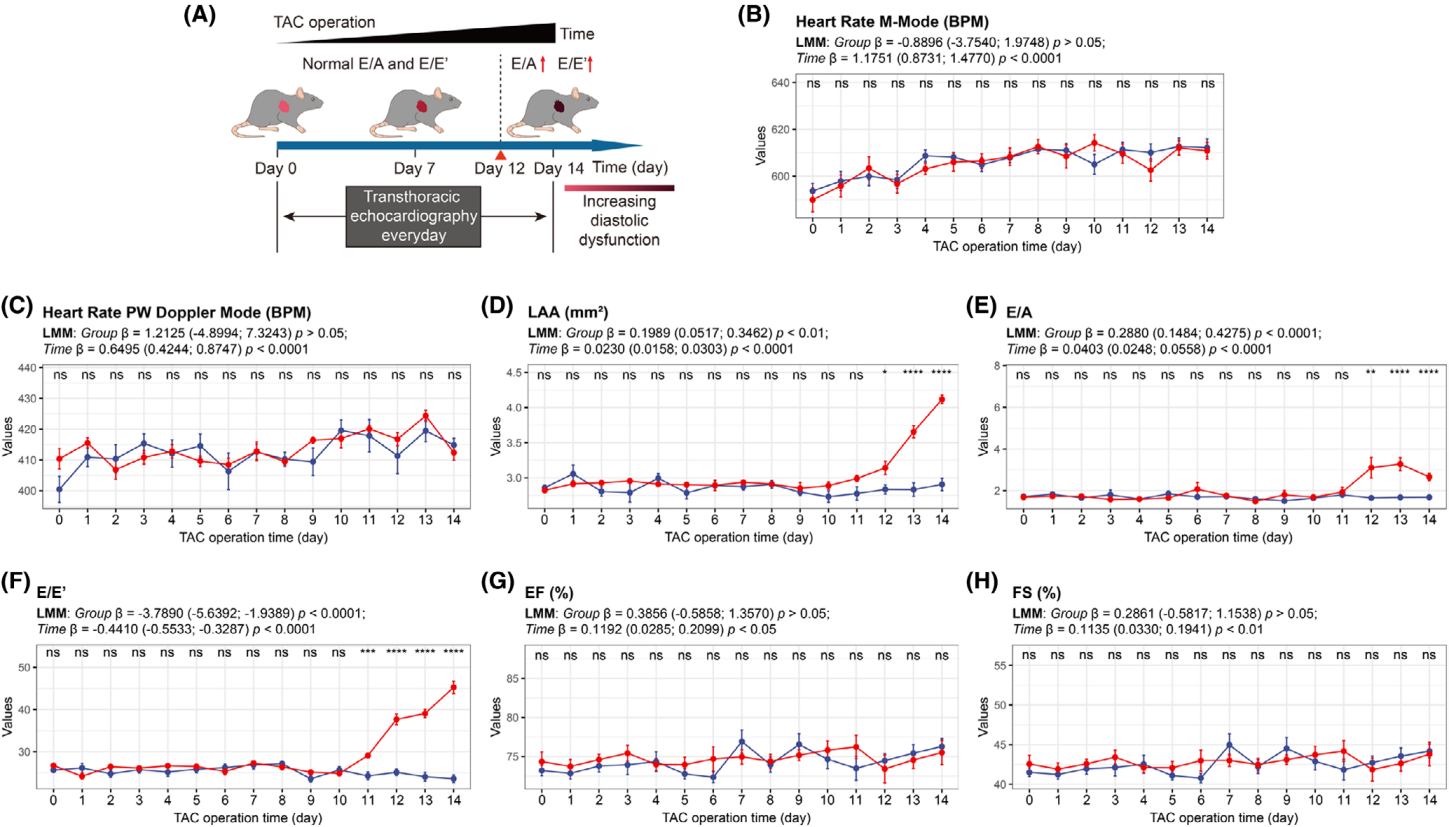
Serial conventional echocardiographic measurements in sham‐operated and TAC mice over the 14‐day post‐operative period. (A) Conventional echocardiography was performed in both sham‐operated and TAC mice over a 14‐day period post‐operation. (B–H) The alternations of heart rate M‐mode (B), heart rate PW Doppler mode (C), LAA (D), E/A (E), E/E′ (F), EF (G) and FS (H) in the sham‐operated and TAC mice. EF, ejection fraction; FS, fractional shortening; LAA, left atrial area; ns, not significant; TAC, transverse aortic constriction; *p < 0.05; **p < 0.01; ***p < 0.001; ****p < 0.0001.

We performed conventional echocardiography, the primary clinical assessment method, in both sham‐operated and TAC mice over a 14‐day period post‐operation to compare the sensitivity of conventional echocardiography and IVSI (Figure 2A). The postoperative body weight changes of the two groups of mice over time are presented in Figure S1. No significant differences in heart rate were observed between groups (all *p* > 0.05, Figure 2B,C). By day 12, TAC mice exhibited impaired diastolic function, evidenced by increased LAA (*p* < 0.05, Figure 2D), E/A ratio (*p* < 0.01, Figure 2E) and E/E′ ratio (*p* < 0.001, Figure 2F). The specific LAAs for the two groups of mice over the 14‐day period are provided in Table S1. In contrast, systolic function parameters, including EF (all *p* > 0.05, Figure 2G) and FS (all *p* > 0.05, Figure 2H and Table S1), remained unchanged throughout the study period in both groups. Notably, conventional echocardiographic parameters of wall thickness, including LVAWd, LVAWs, LVPWd, and LVPWs, showed no evidence of hypertrophy in TAC mice within the first 14 days post‐surgery (Table S1), which may appear inconsistent with expected pressure‐overload‐induced remodeling kinetics. This apparent discrepancy could be attributed to the following factors: First, the early phase of hypertrophy may primarily involve cellular and molecular adaptations (e.g., cardiomyocyte hypertrophy and altered calcium handling) that precede macroscopic structural changes detectable by echocardiography. Second, the murine TAC model may exhibit a delayed hypertrophic response compared to other species or models due to differences in hemodynamic loading conditions and compensatory mechanisms.

### 4.3 Detection of diastolic dysfunction by diastolic strain imaging

**FIGURE 3:**
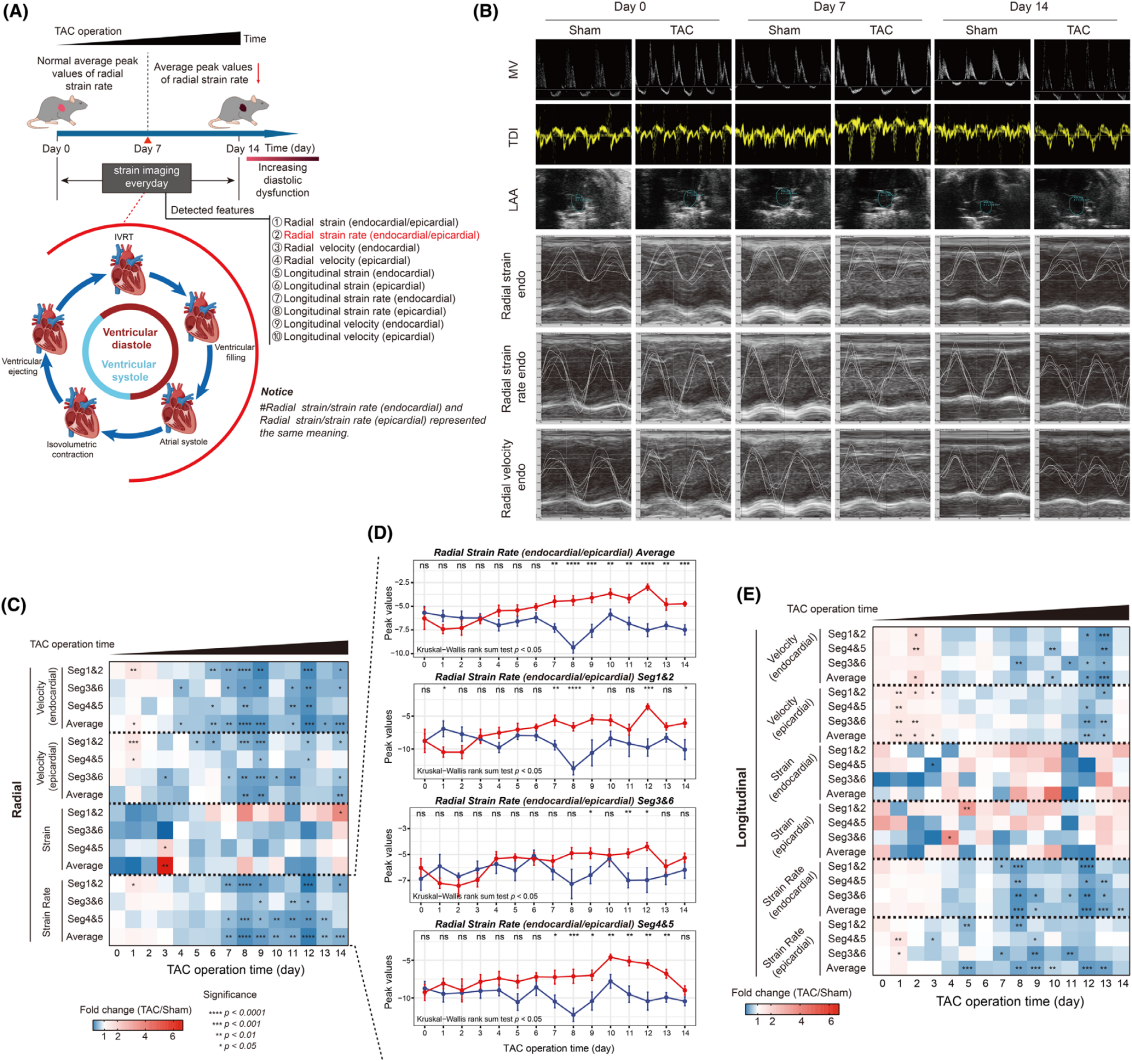
The efficacy of diastolic strain imaging in detecting diastolic dysfunction of TAC mice over a 14‐day period post‐operation. (A) Experimental timeline and analysis framework for diastolic strain imaging. (B) Representative images from conventional echocardiography (top) and diastolic strain imaging (bottom) in sham and TAC mice at day 0 (baseline), day 7, and day 14. (C) Quantitative analysis of radial strain parameters shows progressive diastolic impairment in TAC mice compared to sham controls. From top to bottom: endocardial radial velocity, epicardial radial velocity, radial strain, and radial strain rate. (D) Average peak radial strain rate is significantly reduced in TAC mice from day 7 onward, indicating impaired diastolic relaxation. (E) Longitudinal strain parameters similarly show declining trends in TAC mice, including endocardial longitudinal velocity, epicardial longitudinal velocity, longitudinal strain, and longitudinal strain rate. ns, not significant; *p < 0.05; **p < 0.01; ***p < 0.001; ****p < 0.0001.

To preliminarily evaluate the utility of strain imaging for detecting diastolic dysfunction, we analyzed strain parameters throughout the entire diastole in both TAC and sham‐operated mice. Key assessed parameters included radial strain, radial strain rate, endocardial and epicardial radial velocity; longitudinal strain, longitudinal strain rate and longitudinal velocity across both endocardial and epicardial layers (Figure 3A). The representative images of conventional echocardiography and diastolic strain imaging of these mice in these groups were displayed in Figure 3B.

From day 7 to day 14, TAC mice showed decreased peak radial velocity (endocardial/epicardial) and strain rate (Figure 3C). The average peak radial strain rate declined significantly and continuously during this period (*p* < 0.01, Figure 3D), with similar trends observed in longitudinal strain rate (endocardial/epicardial; Figure 3E). It is important to note that the strain rate analysis here refers to the strain rate measured during the early diastolic phase, as defined in previously published studies.^20^ The results of the LMM analysis on diastolic strain imaging are provided in Table S2. Of note, a transient increase in radial strain rate was observed in sham‐operated animals at day 8 (Figure 3D), which may reflect physiological variability or adaptive responses to the surgical stress and anesthesia regimen. However, this phenomenon was not sustained and did not affect the overall statistical significance or conclusions regarding diastolic impairment in TAC mice. As the temporal derivative of strain, strain rate reflects deformation speed. These results demonstrated that diastolic strain imaging identified declining diastolic function in TAC mice as early as postoperative day 7, indicating that the radial strain rate may serve as a key indicator of impaired diastolic performance.

### 4.4 Assessment of systolic dysfunction in TAC mice by systolic strain imaging

We also assessed cardiac systolic function in TAC mice using systolic strain imaging during systole (Figure S2A). Representative images of conventional echocardiography and systolic strain imaging analyses from these mice were presented in Figure S2B.

Radial analysis revealed significant systolic dysfunction in TAC mice commencing at postoperative day 7 (Figure S2C). Both the average peak radial endocardial velocity (Figure S2D) and radial strain (Figure S2E) in TAC mice exhibited consistent and significant declines. Meanwhile, longitudinal analysis detected systolic impairment at day 8 (Figure S3). The results of the LMM analysis on systolic strain imaging are provided in Table S3. These temporal and directional differences highlight the heterogeneous myocardial responses to loading stress. The decline in radial strain (reflecting wall thickening impairment) marks acute compensatory failure, while altered longitudinal strain (reflecting apex‐to‐base shortening) suggests progressive subendocardial fiber injury and cumulative remodeling effects. Collectively, significant systolic dysfunction was evident by day 7 post‐TAC, with radial endocardial velocity and radial strain emerging as key indicators.

### 4.5 Early detection of active diastolic dysfunction by IVSI in TAC mice

Diastolic strain imaging and systolic strain imaging indicated that both diastolic and systolic dysfunction appeared by postoperative day 7 in TAC mice. However, previous research suggested that diastolic dysfunction emerges earlier than systolic dysfunction during the progression of HF^4^. This inconsistency may stem from diastolic strain imaging confounding active relaxation with passive relaxation. Therefore, we developed a novel method, IVSI, described in detail above, to directly assess active diastolic function in mice.

**FIGURE 4:**
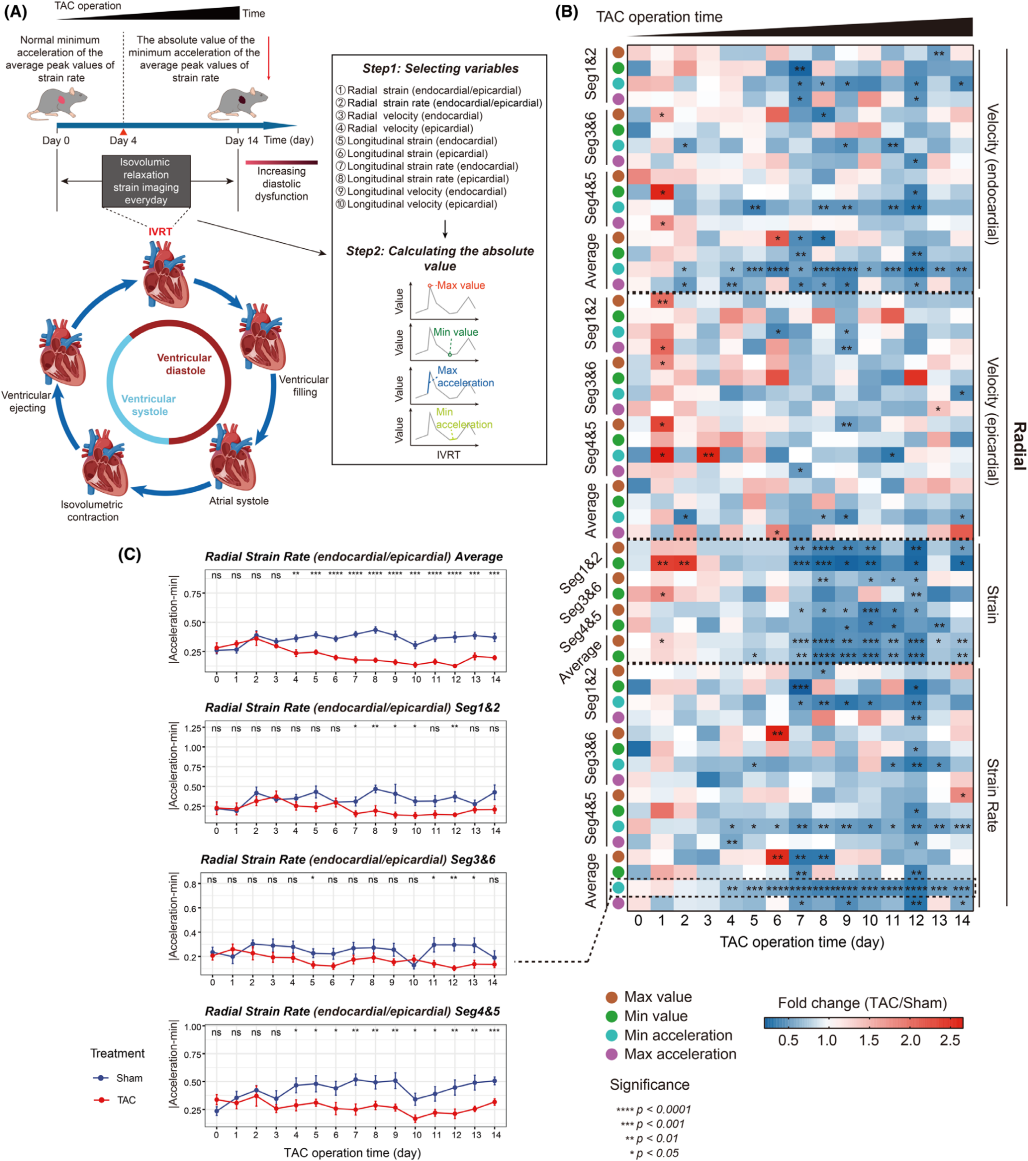
Evaluation of diastolic dysfunction by IVSI in TAC mice during the 14‐day post‐operative period. (A) IVSI was performed in both sham‐operated and TAC mice over a 14‐day period post‐operation. (B) The alternations of the maximal value, minimal value, maximal acceleration, and minimal acceleration of radial velocity (endocardial/epicardial), strain, and strain rate of the mice over a 14‐day period. (C) The minimal acceleration of peak radial strain rate in TAC mice showed a stable decrease from day 4 to day 14. ns, not significant; *p < 0.05; **p < 0.01; ***p < 0.001; ****p < 0.0001.

To further characterize myocardial dynamics during the IVRT, we extended our analysis beyond the 10 previously described parameters. Specifically, we calculated the maximal value, minimal value, maximal acceleration, and minimal acceleration of each parameter during IVRT and analyzed their absolute values (Figure 4A). The corresponding R codes used to process these data can be found in Supporting Information 1.

IVSI analysis demonstrated significant and stable reduction in minimal acceleration of radial endocardial velocity and radial strain rate in TAC mice as early as postoperative day 4 (Figure 4B). Notably, segments 4 and 5 exhibited a similar early decline in radial strain rate minimal acceleration (Figure 4C). Segments 4 and 5 specifically correspond to the mid‐anteroseptal and basal anteroseptal regions, respectively. The coordinate system was defined with the apex as the origin, the base‐to‐apex direction as the longitudinal axis, and the radial direction perpendicular to the epicardial surface. These alterations represent the initial site of diastolic impairment progression in the TAC mice. In contrast, minimal acceleration of longitudinal strain rate showed no consistent change (Figure S4). Notably, LMM analysis indicated that the minimal acceleration of radial strain rate was significantly reduced in TAC mice (*β* = −0.1404, 95% confidence interval [CI] = −0.164 to −0.1167, FDR <0.0001) and showed a significant association with postoperative time (*β* = −0.0036, 95% CI = −0.0063 to −0.0009, FDR <0.05, Table S4), suggesting that this parameter is influenced by both TAC surgery and the duration after the procedure. Taken together, IVSI is a valuable method for detecting early diastolic dysfunction in TAC mice, with the minimal acceleration of radial strain rate as the most sensitive indicator.

### 4.6 Comparison of the PV loop with IVSI for detection of early diastolic dysfunction

Currently, the PV loop is considered the gold standard for diagnosing diastolic dysfunction. Given the invasive nature of this method, we additionally included 40 mice to compare the sensitivity of PV‐loop and IVSI in detecting diastolic function. We selected the 4th and 7th days when IVSI and diastolic strain rate showed the earliest changes as the observation points. These 40 mice were randomly divided into two groups (*n* = 20 per group): a sham‐operated and a TAC group. On day 4 after surgery, 20 mice (10 TAC and 10 sham) underwent PV loop analysis, conventional echocardiography, and IVSI evaluation. The rest of mice underwent the same examination on the day 7. Aortic velocity measurements confirmed successful constriction in TAC mice at both day 4 and day 7 (both *p* < 0.0001; Figures S5 and S6). The results showed that on postoperative day 4, none of the PV loop‐derived parameters, including max, −*d*P/*dt* min, and Tau, exhibited significant changes (all *p* > 0.05). Consistent with previous findings, echocardiographic parameters also showed no significant alterations (all *p* > 0.05). However, the established key IVSI parameter, the minimal acceleration of radial strain rate, was significantly reduced in TAC mice (*p* < 0.05, Figure S5). In assessments performed on postoperative day 7, −*d*P/*dt* min and Tau derived from PV loop analysis showed significant decreases and increases in the TAC group, respectively (both *p* < 0.001), while the minimal acceleration of radial strain rate measured by IVSI also demonstrated a significant reduction (*p* < 0.0001, Figure S6). These findings suggest that IVSI may possess higher sensitivity than PV loop for detecting early‐stage diastolic dysfunction.

### 4.7 Superiority of IVSI in detecting diastolic dysfunction in TAC mice with severe HF

Patients with severe HF usually also show significant ventricular enlargement, and may exhibit pseudo‐normal E/A and non‐significant E/E′ ratios, reflecting the limitations of conventional echocardiography. Therefore, we compared conventional echocardiography and IVSI measurement results between sham‐operated and TAC mice at weeks 2, 4 and 8 to re‐confirm the sensitivity of IVSI.

**FIGURE 5:**
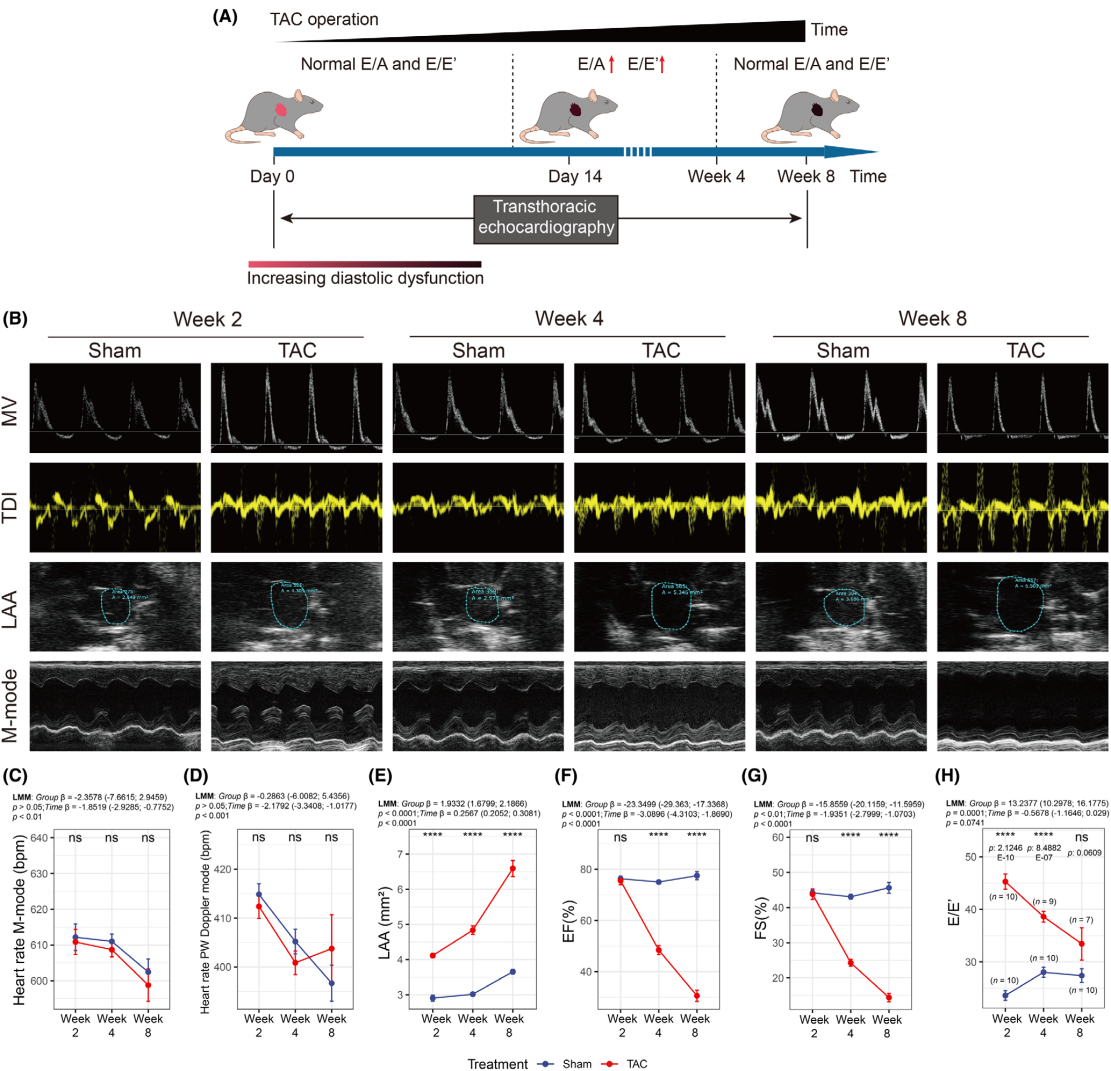
Serial conventional echocardiographic assessments in sham‐operated and TAC mice at 2, 4, and 8 weeks post‐surgery. (A) Conventional echocardiography was performed in both sham‐operated and TAC mice at weeks 2, 4, and 8 post‐operation. (B) The representative images of conventional echocardiography of TAC and sham‐operated mice at weeks 2, 4, and 8. (C–H) The alternations of heart rate M‐mode (C), heart rate PW Doppler mode (D), LAA (E), EF (F), FS (G), and E/E′ (H) in the sham‐operated and TAC mice. ns, not significant; ****p < 0.0001.

Cardiac function in TAC and sham‐operated mice was serially assessed by conventional echocardiography at weeks 2, 4, and 8 post‐operation (Figure 5A). The changing sample sizes due to attrition are summarized in Figure S7. Briefly, of the 10 TAC mice initially randomized, 3 died during the follow‐up period (1 between weeks 2 and 4 and 2 between weeks 4 and 8), resulting in 7 TAC mice available for analysis at week 8. All sham‐operated mice survived, with 10 mice included throughout the study. Representative images were presented in Figure 5B. Aortic flow velocity, which serves as a key indicator of the severity of aortic arch stenosis in these mice, is presented in Table S5. Echocardiographic analysis revealed no significant intergroup differences in heart rate at any time point (Figure 5C,D). Progressive LAA enlargement was observed in the TAC group over time (Figure 5E and Table S6). While EF (Figure 5F) and FS (Figure 5G) declined significantly in TAC mice at weeks 4 and 8 with remarkable ventricular dilation (Table S6), indicating progressive HF deterioration. The E/E′ ratio, a measure of diastolic function, showed no difference up to week 8 (Figure 5H and Table S6). These pseudonormal results highlight the limitations of conventional echocardiography in detecting diastolic dysfunction in the context of cardiac structural remodeling.

**FIGURE 6:**
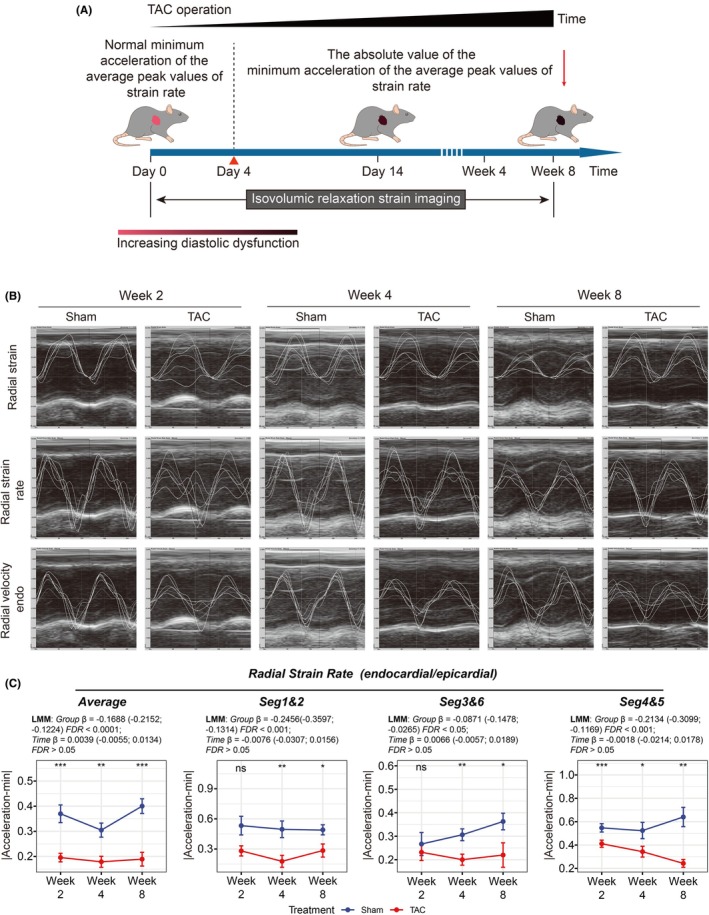
Radial assessment of diastolic dysfunction by IVSI in TAC mice at 2, 4, and 8 weeks post‐operation. (A) IVSI was performed in both sham‐operated and TAC mice at weeks 2, 4, and 8 post‐operation. (B) The representative images of IVSI in TAC and sham‐operated mice at weeks 2, 4, and 8. (C) A progressive reduction in the minimal acceleration of peak radial strain rate was observed in TAC mice at weeks 4 and 8. ns, not significant; *p < 0.05; **p < 0.01; ***p < 0.001.

Active diastolic function was subsequently assessed in TAC and sham‐operated mice by IVSI at postoperative weeks 2, 4, and 8 (Figure 6A). The representative images of IVSI were displayed in Figure 6B. We detected a significant reduction in the minimal acceleration of radial strain rate in TAC mice at both weeks 4 and 8 (Figure 6C). These findings demonstrated that IVSI can accurately detect diastolic dysfunction in TAC mice, even in advanced heart failure with significant structural remodeling, further validating the method's reliability and stability.

## 5 DISCUSSION

Impaired active diastole disrupts ventricular filling, elevates atrial pressures, and promotes pulmonary/systemic congestion, and is a key pathological mechanism in heart failure with preserved ejection fraction (HFpEF), which comprises about 50% of global HF cases.^23^, ^24^, ^25^ Accurate assessment is critical for early HF diagnosis, classification, and treatment. While pulse wave Doppler echocardiography is the standard clinical method, it has limitations in severe HF with left ventricular enlargement.^11^, ^26^, ^27^ Furthermore, although the murine model is the most popular animal model for laboratory research, there is so far no reliable technique for accurate evaluation of active diastolic dysfunction, especially in non‐invasive cardiac imaging. To address this gap, we developed IVSI, a strain imaging‐based method for assessing active relaxation dysfunction. In an 8‐week study of TAC versus sham‐operated mice, IVSI demonstrated superior sensitivity and accuracy to conventional echocardiography in detecting diastolic dysfunction.

The superior sensitivity of IVSI is clearly demonstrated by comparison with conventional diastolic strain imaging. While conventional analysis of the entire diastole detected a reduction in average peak radial strain rate beginning at postoperative day 7 (Figure 3D), IVSI, by measuring minimal acceleration of radial strain rate specifically during the IVRT, revealed a significant decrease as early as day 4 (Figure 4C). This three‐day advance in detection is a key advantage of IVSI method. Conventional diastolic strain imaging assesses global deformation across diastole, indicating the combined impact of both the active relaxation phase (IVRT and early filling) and the later passive filling phase. As a result, in early diastolic dysfunction, subtle impairments in active relaxation may be obscured. In contrast, IVSI, a marker purely of active relaxation, isolates the IVRT, offering a more direct and specific measure. Therefore, IVSI represents a superior approach to conventional strain analysis, enabling earlier identification of initial active diastolic dysfunction.

In IVSI analysis, the minimum acceleration of radial strain rate exhibited the highest sensitivity and stability for diagnosing active diastolic dysfunction in TAC mice, suggesting its potential as a diagnostic marker. The efficacy of the method stems from its pathophysiological basis. The minimum acceleration of radial strain rate quantifies the rate of change in myocardial fiber extension velocity radially during IVRT. Impaired sarcoplasmic reticulum calcium reuptake delays active myocardial relaxation and therefore directly reduces the minimum acceleration of radial strain rate (reflecting slower deceleration). This mechanistic link likely underpins the diagnostic robustness of the method, yet the molecular pathways underlying progressive dysfunction warrant further elucidation.

Additionally, IVSI analysis also identified the mid‐to‐base anteroseptum as the earliest site of diastolic dysfunction in TAC mice, highlighting the spatial specificity of this technique. Aortic arch ligation induces pressure‐overload, directly exposing the anteroseptal region (adjacent to the left ventricular outflow tract) to elevated afterload.^28^ During the systole, high‐velocity blood flow passively stretches cardiomyocytes, causing metabolic dysregulation and early relaxation impairment. Mechanical stretch further exacerbates dysfunction due to Laplace's law.^29^, ^30^ The basal anteroseptum's larger radius of curvature concentrates wall stress, promoting ventricular hypertrophy and fibrosis.^31^ Molecularly, pressure‐overload increases myocardial oxygen demand. The stress‐concentrated anteroseptum experiences hypoxia, thereby reducing adenosine triphosphate (ATP) synthesis and elevating reactive oxygen species (ROS). Since active diastoles are ATP dependent, energy depletion and ROS damage directly impair cardiomyocyte relaxation.^32^, ^33^, ^34^

Routine echocardiography faces challenges in detecting subtle diastolic dysfunction, particularly with insignificant E/E′ changes and pseudonormalized E/A ratios.^35^ These cases often require complex multiparametric analysis or biomarkers (e.g., NT‐proBNP), which potentially delays diagnosis.^36^ The diagnostic challenges of pseudonormalization are especially evident in advanced heart failure and conditions such as long‐standing hypertension, hypertrophic cardiomyopathy, and cardiac amyloidosis, where conventional indices like E/E′ and E/A lose sensitivity due to structural remodeling and altered loading. In such scenarios, IVSI adds value as a complementary tool. Unlike NT‐proBNP, a sensitive biomarker of wall stress but limited by non‐cardiac influences and lack of relaxation specificity, IVSI directly quantifies intrinsic myocardial relaxation velocity during the IVRT, offering a more targeted functional assessment. Similarly, while left atrial strain and tissue Doppler imaging reflect atrial remodeling and myocardial motion, both can be confounded by pseudonormalization. By focusing on the isovolumic phase, which is relatively load independent, IVSI may identify impaired active relaxation even when other parameters appear paradoxically normalized. Therefore, the integration of IVSI into a multi‐parametric diagnostic algorithm, combining NT‐proBNP for wall stress, LA strain for atrial function and chronicity, and IVSI for active relaxation, could significantly enhance the early detection and phenotyping of diastolic dysfunction, especially in complex cases where pseudonormalization confounds conventional echocardiographic assessment.

To date there are no direct imaging methods for assessing active diastolic dysfunction in mice. Active diastole reflects cardiomyocyte function, while passive relaxation relates to extracellular matrix regulation.^6^ Currently used murine diastolic function parameters (e.g., E/E′ and E/A ratios) conflate both relaxation components, potentially biasing experimental results and causing discrepancies between animal‐level and cardiomyocyte‐level results. The introduction of IVSI offers a potential solution to this problem. This approach isolates active relaxation, enabling precise diastolic assessment in mice. This method is also straightforward to implement: it only requires a rotational adjustment of the conventional four‐chamber view to achieve the novel three‐chamber view proposed in this study. Additionally, corresponding R software coding for data processing and calculations has been provided in the Supporting Information section.

The IVSI technique holds significant potential for clinical translation. Its foundation in speckle‐tracking echocardiography, a well‐established modality in clinical cardiology, facilitates relatively straightforward integration into existing workflows without major new investments. Crucially, IVSI addresses a key diagnostic gap by directly quantifying active relaxation, which is particularly valuable in HFpEF where diastolic dysfunction occurs despite preserved ejection fraction. This capability to detect impaired relaxation prior to structural changes could enable earlier intervention and improved risk stratification. For human application, IVSI would primarily require technical rather than conceptual modifications, as it can be implemented using standard apical three‐chamber views. The automated analysis pipeline developed here (provided in Supporting Information 1) could be integrated into existing ultrasound platforms, enhancing accessibility for clinicians. Furthermore, the segment‐specific analysis offered by IVSI allows for targeted assessment of regional diastolic function, beneficial in conditions like ischemic heart disease or hypertrophic cardiomyopathy characterized by heterogeneous impairment. Future studies should establish normative values, validate diagnostic accuracy in human HFpEF cohorts, and determine prognostic utility. The technique's sensitivity to early functional changes also suggests applications in monitoring treatment response and guiding therapy for diastolic dysfunction.

A systematic evaluation of IVSI must consider its load dependence and technical validation. Although strain and strain rate are inherently load sensitive, the acceleration of strain rate during IVRT, as a higher‐order derivative, may be less directly affected by acute preload and afterload changes than volumetric or Doppler flow indices, since it primarily reflects the kinetics of active, energy‐dependent relaxation. Future studies specifically manipulating loading conditions are needed to clarify this relationship. Technically, the reliable measurement of IVSI hinges on accurate identification of the IVRT interval, which is highly dependent on imaging parameters, particularly temporal resolution (frame rate) and ultrasound beam characteristics. A sufficiently high frame rate (>200 fps in mice, approximating >60 fps in humans) is critical to precisely capture aortic and mitral valve events and minimize blurring of the IVRT window, thereby reducing noise in strain rate acceleration calculations. Spatial resolution, determined by transducer frequency and beam width, also influences valve tracking clarity. In this study, the use of a 30‐MHz probe with optimized frame rates, as detailed in the Methods, ensured high temporal resolution. The resulting excellent intra‐ and inter‐observer reproducibility underscores the robustness of the approach when these technical factors are controlled.

It is noteworthy that daily anesthesia could influence diastolic function, as isoflurane, like other volatile anesthetics, has known dose‐dependent effects on myocardial relaxation.^37^, ^38^ In this study, however, a low concentration of isoflurane (1.0%–1.5%) was used to maintain anesthesia with heart rate consistently controlled within a narrow range (590–610/400–450 bpm) to minimize anesthetic impact. The observation of significant diastolic differences between TAC and sham‐operated mice under identical anesthesia levels, suggests that the dysfunction in TAC mice stems from pathological remodeling rather than anesthesia‐related artifacts. Although the cumulative effects of repeated anesthesia are inevitable, employing anesthesia‐free imaging approaches, such as awake echocardiography, is technically challenging for IVSI measurement.

We acknowledge that the open‐chest TAC procedure used in our study has inherent limitations, including procedural variability and postoperative mortality. These concerns are being effectively addressed by the development of minimally invasive TAC (MTAC) techniques under ultrasound guidance. As reported, MTAC, performed via a suprasternal incision, can achieve lower operative mortality and reduce variability in the induced pressure gradient, thereby significantly improving the model's reproducibility.^39^, ^40^, ^41^ Although our current experimental model utilized the modified conventional TAC approach and achieved stable outcomes, the implementation of MTAC in future studies should be optimized for enhanced survival rates and reproducibility, which would certainly facilitate a clearer longitudinal assessment of disease progression, particularly the earliest phases of diastolic dysfunction.

The limitations of this study should be emphasized. First, this study only used the TAC mouse model to investigate the sensitivity and accuracy of IVSI in detecting active diastolic dysfunction. Other animal models with diastolic dysfunction, such as the coronary artery ligation mouse model, the high‐fat diet mouse model, and mouse models with diastolic function‐related gene knockouts, were not explored. Second, although we chose male mice to develop cardiac hypertrophy because male mice typically develop more pronounced hypertrophy than the female, the exclusive use of male mice limits the generalizability of our findings to female populations.^42^ Given the well‐documented sex differences in cardiac remodeling pathways, diastolic function regulation, and heart failure progression, future studies should explicitly examine whether IVSI demonstrates similar sensitivity and accuracy in female models of diastolic dysfunction.

## 6 CONCLUSIONS

IVSI is a novel ultrasound strain‐based method for direct assessment of active diastolic dysfunction, demonstrating sensitivity and accuracy in preclinical settings. This technique enables precise detection of impaired active relaxation, offering a novel methodological approach for diastolic evaluation across preclinical and clinical studies.

## AUTHOR CONTRIBUTIONS

**Jingjing Liang:** Data curation; formal analysis; writing – original draft. **Juncheng Wang:** Formal analysis; validation. **Jun Cheng:** Data curation. **Yanggan Wang:** Conceptualization; funding acquisition; writing – review and editing.

## FUNDING INFORMATION

This work was supported by the National Natural Science Foundation of China (NSFC, Grant Nos. 82070348, 81270304, 81873507, 82370393 and 81420108004) and the Key R&D Projects in Hubei Province (Grant 2022BCA002).

## CONFLICT OF INTEREST STATEMENT

No potential conflict of interest was reported by the author(s).

## ETHICS STATEMENT

All animal studies were conducted in accordance with the guidelines of the Animal Care and Use Committee of Wuhan University, approved by the Animal Care and Use Committee of the Laboratory Animal Center, Medical Research Institute of Wuhan University (approval no. MRI2021‐LAC18).

## Supporting information


**Figure S1.** Changes in body weight over time in mice following sham or TAC operation. TAC, transverse aortic constriction.
**Figure S2.** The efficacy of systolic strain imaging in detecting systolic dysfunction of TAC mice over a 14‐day period post‐operation. (A) Systolic strain imaging was performed in both sham‐operated and TAC mice over a 14‐days period post‐operation. (B) The representative images of conventional echocardiography and systolic strain imaging of TAC and sham‐operated mice on day 0, day 7, and day 14. (C) The alternations of the radical velocity (endocardial/epicardial), strain, and strain rate of the mice over a 14‐day period. (D) The average peak radial velocity (endocardial) of TAC mice showed a stable decrease from day 7 to day 14. (E) The average peak radial strain of TAC mice showed a stable decrease from day 7 to day 14.
**Figure S3.** The systolic strain imaging indicated the alternations of the longitudinal velocity (endocardial/epicardial), strain (endocardial/epicardial), and strain rate (endocardial/epicardial) of the mice over a 14‐day period.
**Figure S4.** The IVSI indicated the alternations of the maximal value, minimal value, maximal acceleration, and minimal acceleration of longitudinal velocity (endocardial/epicardial), strain, and strain rate of the mice over a 14‐day period. IVSI, isovolumic relaxation strain imaging.
**Figure S5.** Comparison of the sensitivity between PV loop and IVSI on the fourth day after TAC surgery. (A) Twenty mice were randomly assigned to TAC and sham groups (*n* = 10 per group). On day 4 after TAC surgery, diastolic function was assessed using PV loop, echocardiography, and IVSI. (B–D) Results of PV loop (B), echocardiography (C), and IVSI (D) in mice from the two groups. PV loop, pressure‐volume loop.
**Figure S6.** Comparison of the sensitivity between PV loop and IVSI on the seventh day after TAC surgery. (A) Twenty mice were randomly assigned to TAC and sham groups (*n* = 10 per group). On day 7 after TAC surgery, diastolic function was assessed using PV loop, echocardiography, and IVSI. (B–D) Results of PV loop (B), echocardiography (C), and IVSI (D) in mice from the two groups.
**Figure S7.** Sample size at each time point for sham‐operated and TAC mice.

Data S1.

Data S2.
