# Supplementary material for: Isovolumic relaxation strain imaging is an accurate and sensitive approach for detection of active diastolic dysfunction: A preclinical study
Source: Animal Model Exp Med. 2026 Feb 28;9(3):572–85. doi: 10.1002/ame2.70147 (PMC13176106; doi:10.1002/ame2.70147)
Supplement: Supplementary file 1 — Figure S1. Changes in body weight over time in mice following sham or TAC operation. TAC, transverse aortic constriction. Figure S2. The efficacy of systolic strain imaging in detecting systolic dysfunction of TAC mice over a 14‐day period post‐operation. (A) Systolic strain imaging was performed in both sham‐operated and TAC mice over a 14‐days period post‐operation. (B) The representative images of conventional echocardiography and systolic strain imaging of TAC and sham‐operated mice on day 0, day 7, and day 14. (C) The alternations of the radical velocity (endocardial/epicardial), strain, and strain rate of the mice over a 14‐day period. (D) The average peak radial velocity (endocardial) of TAC mice showed a stable decrease from day 7 to day 14. (E) The average peak radial strain of TAC mice showed a stable decrease from day 7 to day 14. Figure S3. The systolic strain imaging indicated the alternations of the longitudinal velocity (endocardial/epicardial), strain (endocardial/epicardial), and strain rate (endocardial/epicardial) of the mice over a 14‐day period. Figure S4. The IVSI indicated the alternations of the maximal value, minimal value, maximal acceleration, and minimal acceleration of longitudinal velocity (endocardial/epicardial), strain, and strain rate of the mice over a 14‐day period. IVSI, isovolumic relaxation strain imaging. Figure S5. Comparison of the sensitivity between PV loop and IVSI on the fourth day after TAC surgery. (A) Twenty mice were randomly assigned to TAC and sham groups (n = 10 per group). On day 4 after TAC surgery, diastolic function was assessed using PV loop, echocardiography, and IVSI. (B–D) Results of PV loop (B), echocardiography (C), and IVSI (D) in mice from the two groups. PV loop, pressure‐volume loop. Figure S6. Comparison of the sensitivity between PV loop and IVSI on the seventh day after TAC surgery. (A) Twenty mice were randomly assigned to TAC and sham groups (n = 10 per group). On day 7 after TAC surgery, diasto [file AME2-9-572-s002.docx]

**Supplementary Figures**


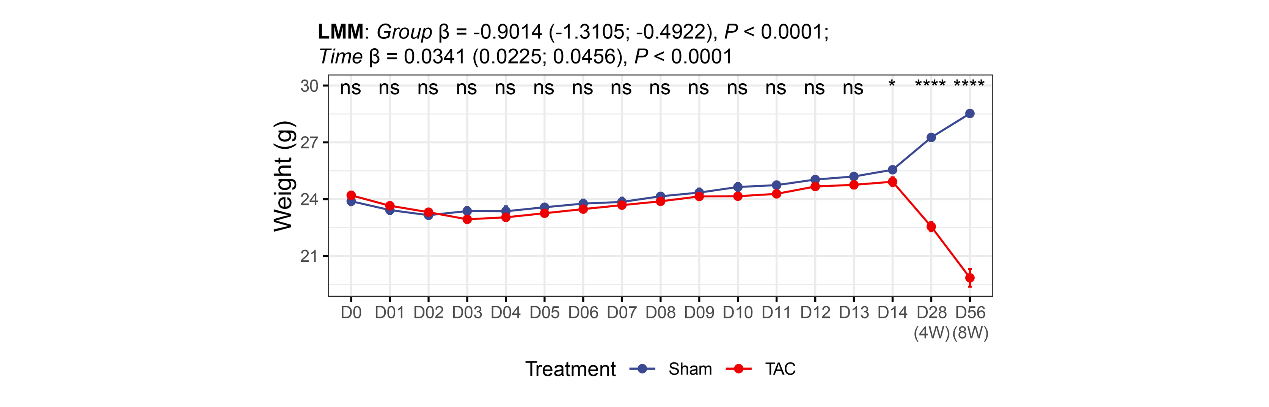


**Figure S1.** Changes in body weight over time in mice following sham or TAC operation. *TAC, transverse aortic constriction*.


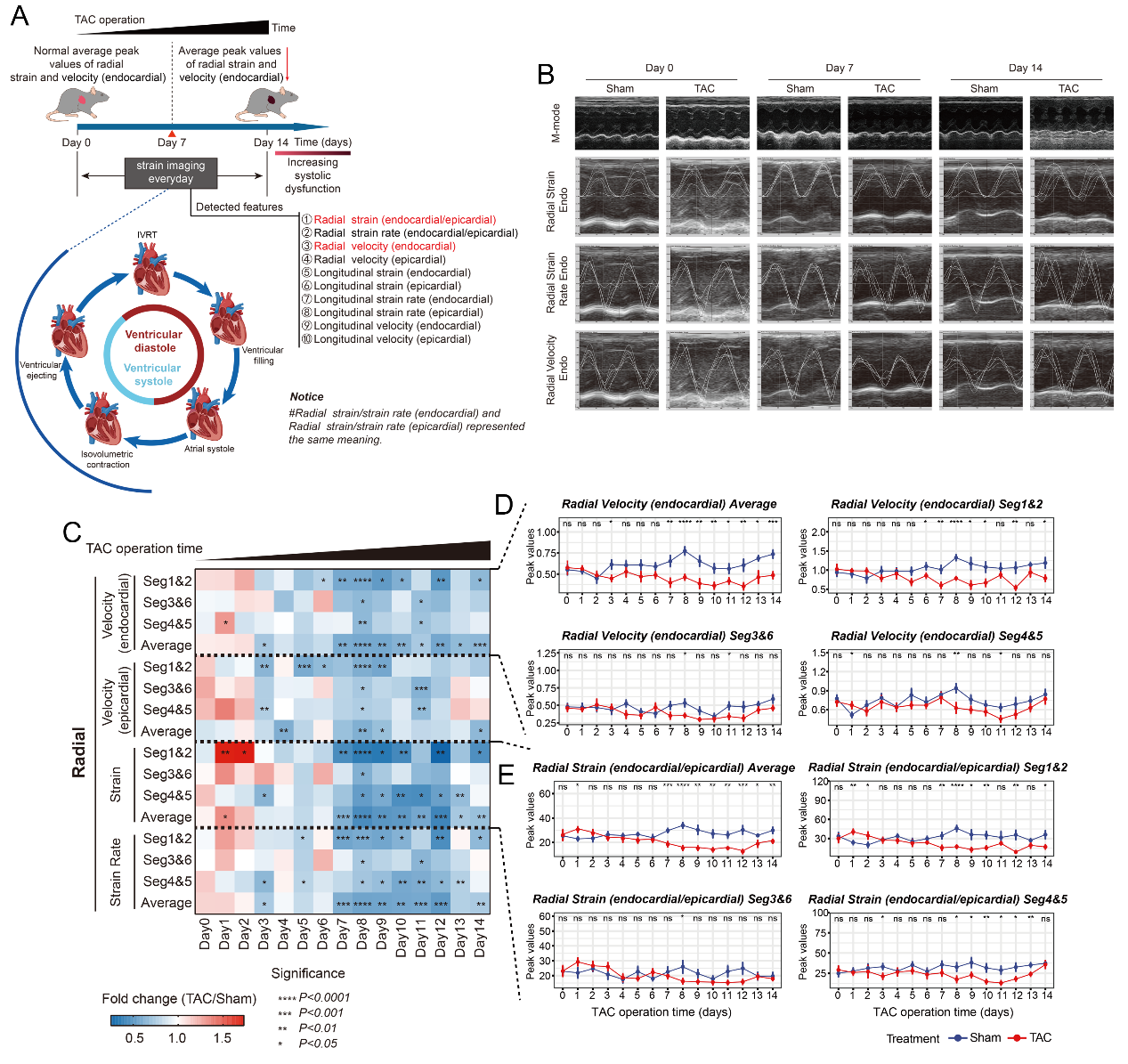


**Figure S2.** The efficacy of systolic strain imaging in detecting systolic dysfunction of TAC mice over a 14-days period post-operation. **A** Systolic strain imaging was performed in both sham-operated and TAC mice over a 14-days period post-operation. **B** The representative images of conventional echocardiography and systolic strain imaging of TAC and sham-operated mice on day 0, day 7, and day 14. **C** The alternations of the radical velocity (endocardial/epicardial), strain, and strain rate of the mice over a 14-days period. **D** The average peak radial velocity (endocardial) of TAC mice showed a stable decrease from day 7 to day 14. **E** The average peak radial strain of TAC mice showed a stable decrease from day 7 to day 14.


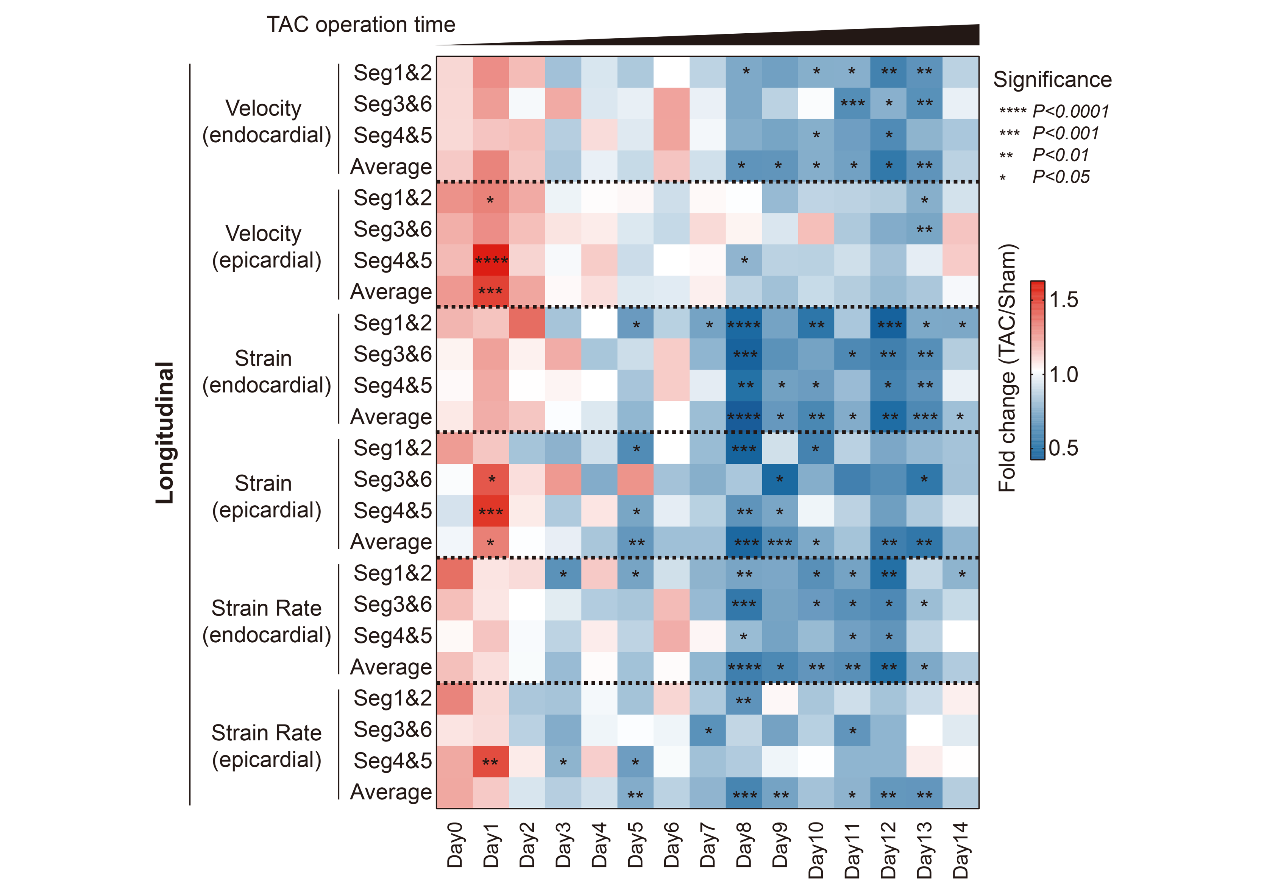


**Figure S3.** The systolic strain imaging indicated the alternations of the longitudinal velocity (endocardial/epicardial), strain (endocardial/epicardial), and strain rate (endocardial/epicardial) of the mice over a 14-days period.


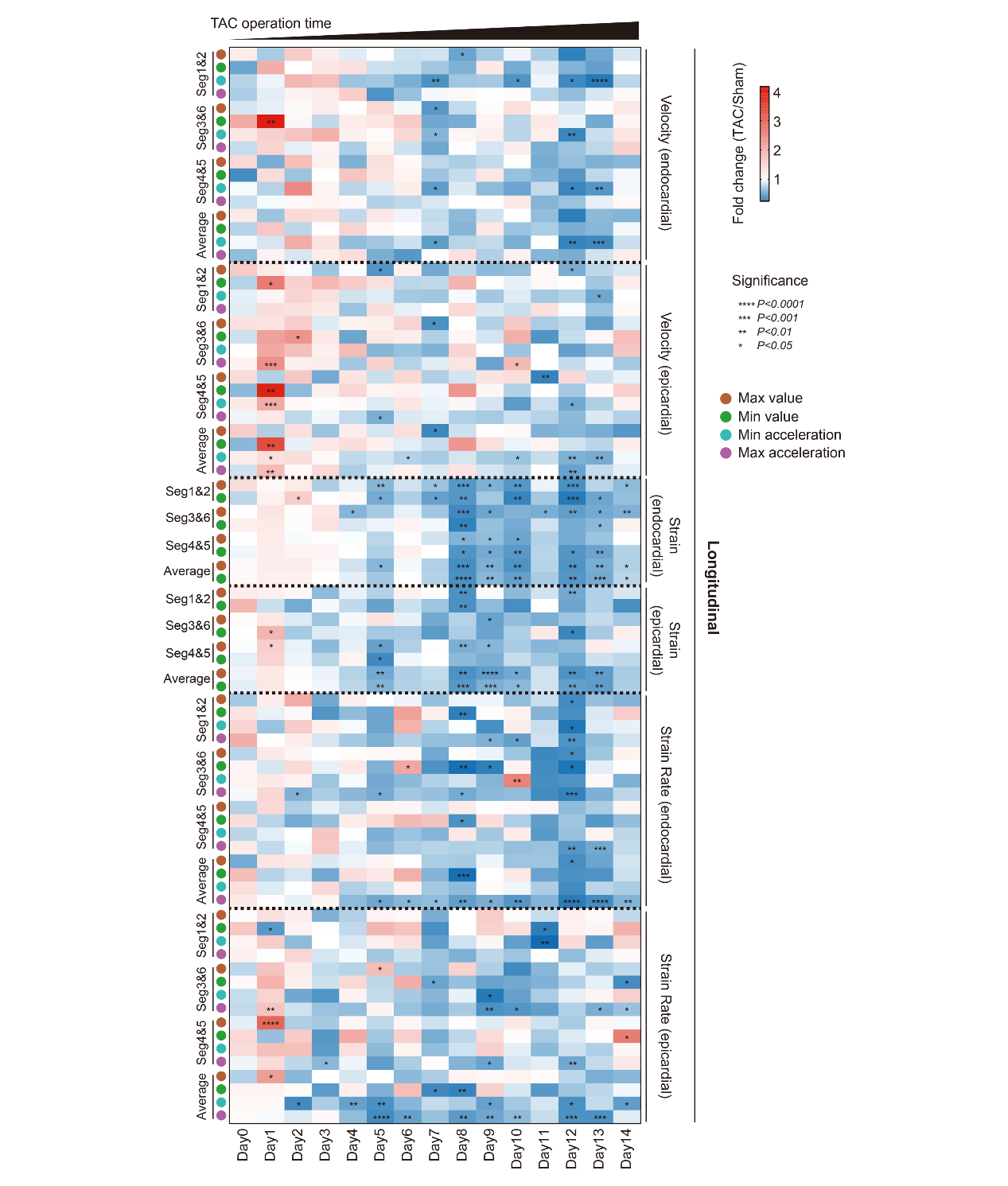


**Figure S4.** The IVSI indicated the alternations of the maximal value, minimal value, maximal acceleration, and minimal acceleration of longitudinal velocity (endocardial/epicardial), strain, and strain rate of the mice over a 14-days period. *IVSI, isovolumic relaxation strain imaging*.


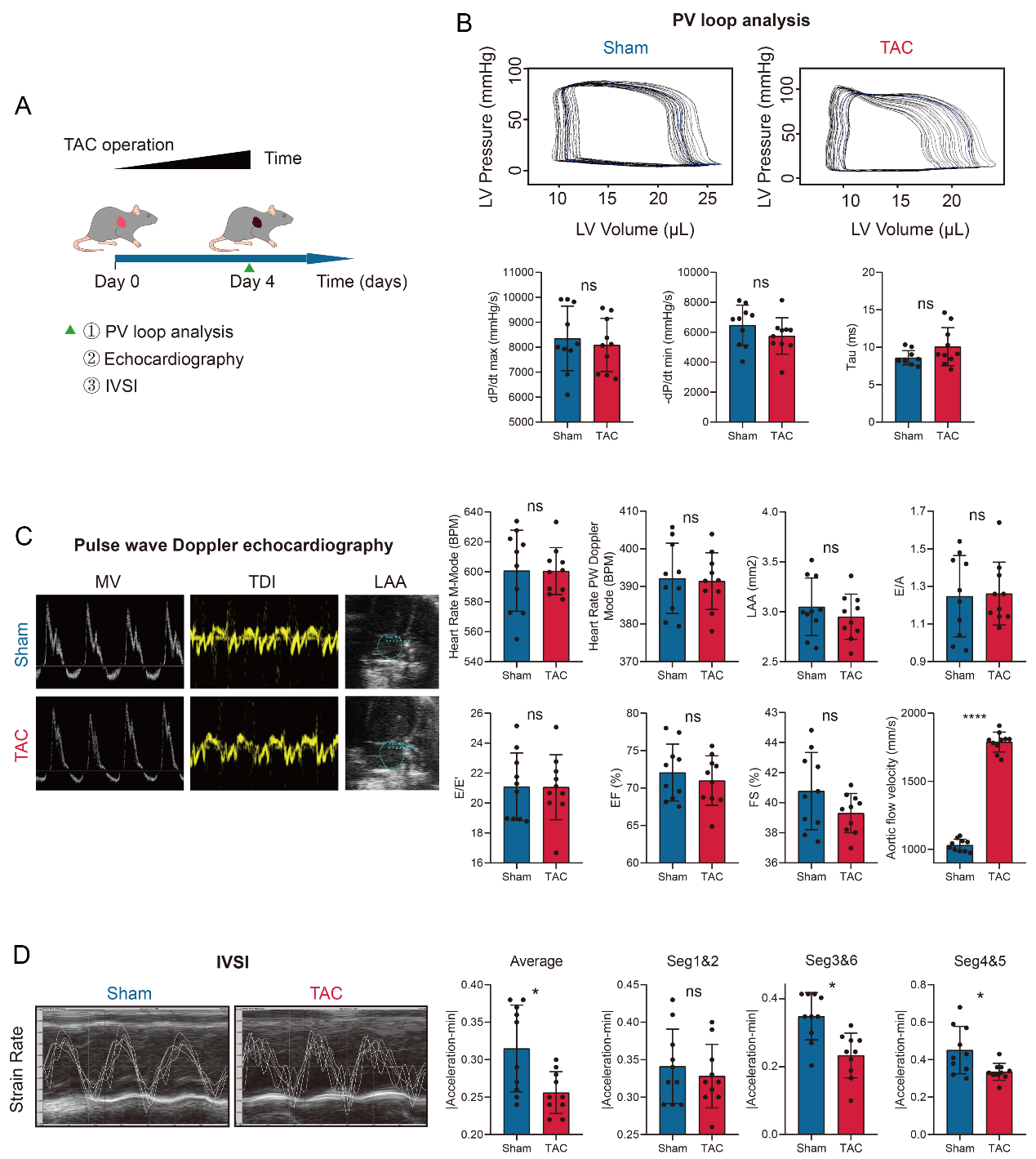


**Figure S5.** Comparison of the sensitivity between PV loop and IVSI on the fourth day after TAC surgery. **A** Twenty mice were randomly assigned to TAC and sham groups (n = 10 per group). On day 4 after TAC surgery, diastolic function was assessed using PV loop, echocardiography, and IVSI. **B-D** Results of PV loop (**B**), echocardiography (**C**), and IVSI (**D**) in mice from the two groups. *PV loop, pressure-volume loop.*


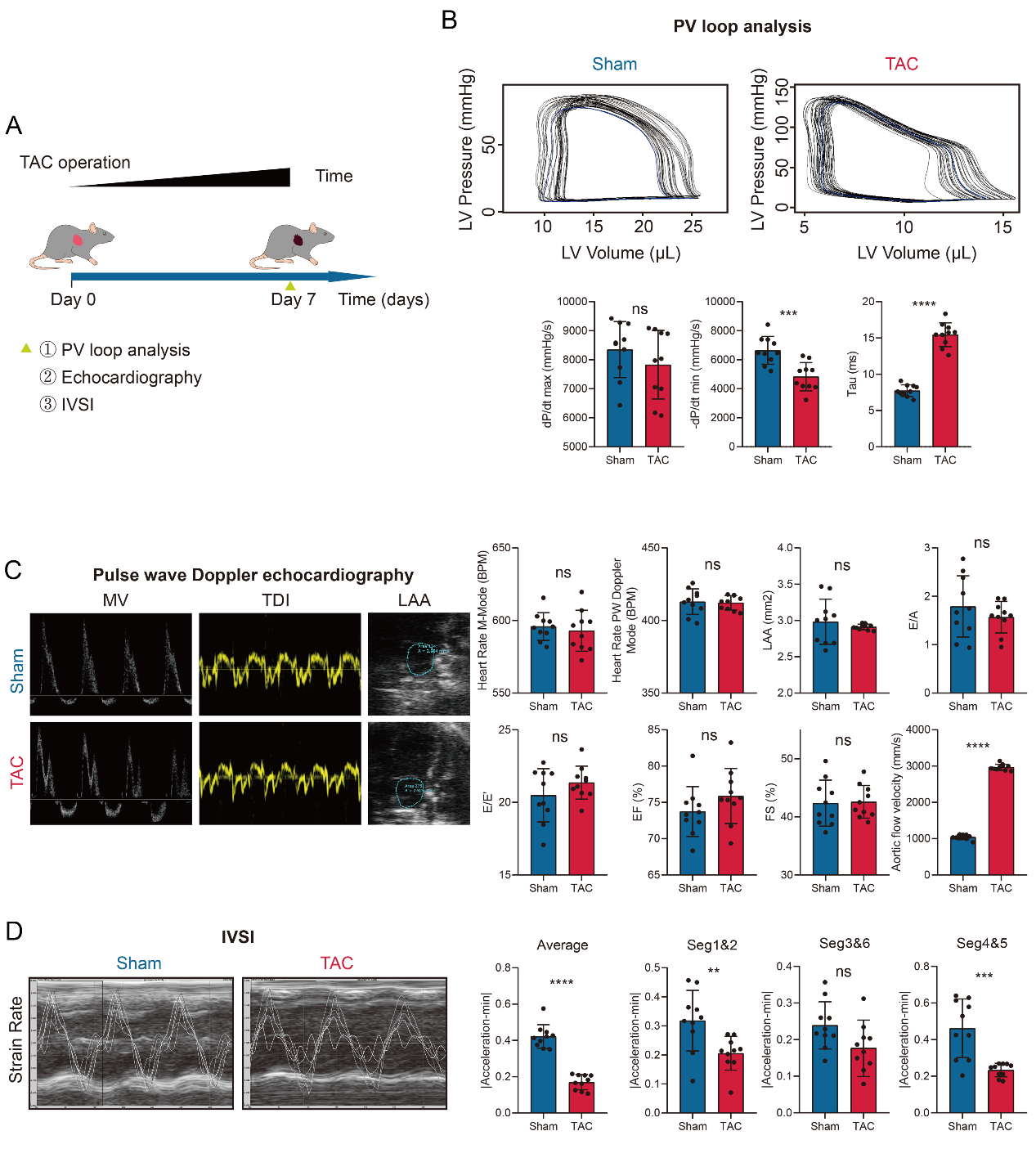


**Figure S6.** Comparison of the sensitivity between PV loop and IVSI on the seventh day after TAC surgery. **A** Twenty mice were randomly assigned to TAC and sham groups (n = 10 per group). On day 7 after TAC surgery, diastolic function was assessed using PV loop, echocardiography, and IVSI. **B-D** Results of PV loop (**B**), echocardiography (**C**), and IVSI (**D**) in mice from the two groups.


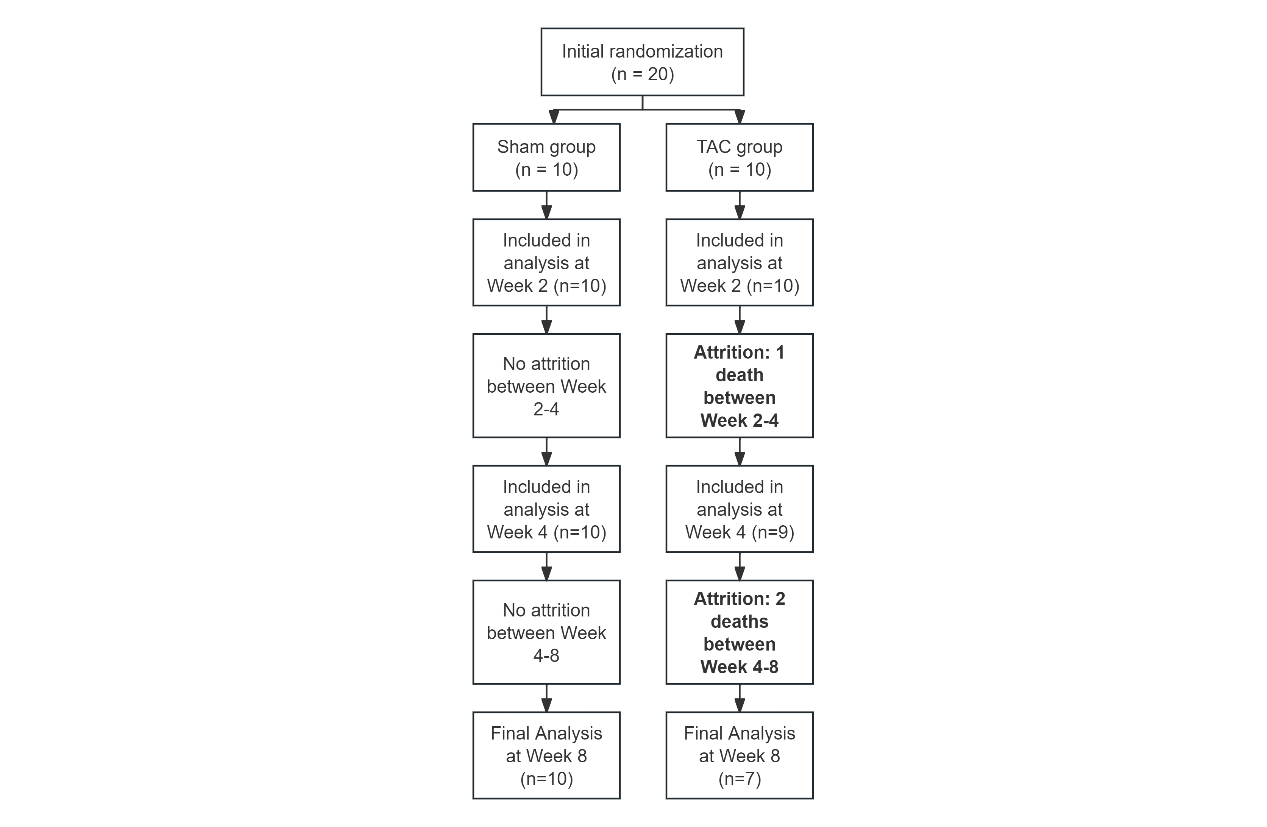


**Figure S7.** Sample size at each time point for sham-operated and TAC mice.
