# Supplementary material for: Isovolumic relaxation strain imaging is an accurate and sensitive approach for detection of active diastolic dysfunction: A preclinical study
Source: Animal Model Exp Med. 2026 Feb 28;9(3):572–85. doi: 10.1002/ame2.70147 (PMC13176106; doi:10.1002/ame2.70147)
Supplement: Supplementary file 2 — Data S1. [file AME2-9-572-s003.docx]

**The R code used for data collection and processing**

Content

[Part 1 Extract data from the original strain imaging acquisition files 1](#_Toc199860103)

[Part 2 Calculate maximum and minimum values as well as accelerations 109](#_Toc199860104)

# Part 1 Extract data from the original strain imaging acquisition files

library(xlsx)

dir="D:\\Data\\TAC_imaging\\raw_data"

type="HF"

setwd(dir)

files=grep(".xlsx",dir(),value=T)

as.data.frame(files)

outTab=data.frame()

for (i in files[1:length(files)]){

time1 = unlist(strsplit(i,split = " "))

time2=time1[2]

time3 = substr(time2,1,2)

#velocity

velocity_data <- read.xlsx(i,sheetIndex = 2, header = F)

colnames(velocity_data) <- velocity_data[3,]

name1 = paste("fr0",time3,sep = "")

col5 = grep(name1,colnames(velocity_data))

col4 = col5-1

col3 = col5-2

col2 = col5-3

col1 = col5-4

Timepoint1 = as.numeric(velocity_data[4,col1])

Timepoint2 = as.numeric(velocity_data[4,col2])

Timepoint3 = as.numeric(velocity_data[4,col3])

Timepoint4 = as.numeric(velocity_data[4,col4])

Timepoint5 = as.numeric(velocity_data[4,col5])

Time_duration1 = Timepoint2 - Timepoint1

Time_duration2 = Timepoint3 - Timepoint2

Time_duration3 = Timepoint4 - Timepoint3

Time_duration4 = Timepoint5 - Timepoint4

##Radial_Velocity_Endo

Radial_Velocity_Endo_seg001_point1 = as.numeric(velocity_data[8,col1])

Radial_Velocity_Endo_seg001_point2 = as.numeric(velocity_data[8,col2])

Radial_Velocity_Endo_seg001_point3 = as.numeric(velocity_data[8,col3])

Radial_Velocity_Endo_seg001_point4 = as.numeric(velocity_data[8,col4])

Radial_Velocity_Endo_seg001_point5 = as.numeric(velocity_data[8,col5])

Radial_Velocity_Endo_seg001_a1 = (Radial_Velocity_Endo_seg001_point2 - Radial_Velocity_Endo_seg001_point1)/Time_duration1

Radial_Velocity_Endo_seg001_a2 = (Radial_Velocity_Endo_seg001_point3 - Radial_Velocity_Endo_seg001_point2)/Time_duration2

Radial_Velocity_Endo_seg001_a3 = (Radial_Velocity_Endo_seg001_point4 - Radial_Velocity_Endo_seg001_point3)/Time_duration3

Radial_Velocity_Endo_seg001_a4 = (Radial_Velocity_Endo_seg001_point5 - Radial_Velocity_Endo_seg001_point4)/Time_duration4

Radial_Velocity_Endo_seg001_a_min = min(c(Radial_Velocity_Endo_seg001_a1,Radial_Velocity_Endo_seg001_a2,Radial_Velocity_Endo_seg001_a3,Radial_Velocity_Endo_seg001_a4))

Radial_Velocity_Endo_seg001_a_max = max(c(Radial_Velocity_Endo_seg001_a1,Radial_Velocity_Endo_seg001_a2,Radial_Velocity_Endo_seg001_a3,Radial_Velocity_Endo_seg001_a4))

Radial_Velocity_Endo_seg002_point1 = as.numeric(velocity_data[9,col1])

Radial_Velocity_Endo_seg002_point2 = as.numeric(velocity_data[9,col2])

Radial_Velocity_Endo_seg002_point3 = as.numeric(velocity_data[9,col3])

Radial_Velocity_Endo_seg002_point4 = as.numeric(velocity_data[9,col4])

Radial_Velocity_Endo_seg002_point5 = as.numeric(velocity_data[9,col5])

Radial_Velocity_Endo_seg002_a1 = (Radial_Velocity_Endo_seg002_point2 - Radial_Velocity_Endo_seg002_point1)/Time_duration1

Radial_Velocity_Endo_seg002_a2 = (Radial_Velocity_Endo_seg002_point3 - Radial_Velocity_Endo_seg002_point2)/Time_duration2

Radial_Velocity_Endo_seg002_a3 = (Radial_Velocity_Endo_seg002_point4 - Radial_Velocity_Endo_seg002_point3)/Time_duration3

Radial_Velocity_Endo_seg002_a4 = (Radial_Velocity_Endo_seg002_point5 - Radial_Velocity_Endo_seg002_point4)/Time_duration4

Radial_Velocity_Endo_seg002_a_min = min(c(Radial_Velocity_Endo_seg002_a1,Radial_Velocity_Endo_seg002_a2,Radial_Velocity_Endo_seg002_a3,Radial_Velocity_Endo_seg002_a4))

Radial_Velocity_Endo_seg002_a_max = max(c(Radial_Velocity_Endo_seg002_a1,Radial_Velocity_Endo_seg002_a2,Radial_Velocity_Endo_seg002_a3,Radial_Velocity_Endo_seg002_a4))

Radial_Velocity_Endo_seg003_point1 = as.numeric(velocity_data[10,col1])

Radial_Velocity_Endo_seg003_point2 = as.numeric(velocity_data[10,col2])

Radial_Velocity_Endo_seg003_point3 = as.numeric(velocity_data[10,col3])

Radial_Velocity_Endo_seg003_point4 = as.numeric(velocity_data[10,col4])

Radial_Velocity_Endo_seg003_point5 = as.numeric(velocity_data[10,col5])

Radial_Velocity_Endo_seg003_a1 = (Radial_Velocity_Endo_seg003_point2 - Radial_Velocity_Endo_seg003_point1)/Time_duration1

Radial_Velocity_Endo_seg003_a2 = (Radial_Velocity_Endo_seg003_point3 - Radial_Velocity_Endo_seg003_point2)/Time_duration2

Radial_Velocity_Endo_seg003_a3 = (Radial_Velocity_Endo_seg003_point4 - Radial_Velocity_Endo_seg003_point3)/Time_duration3

Radial_Velocity_Endo_seg003_a4 = (Radial_Velocity_Endo_seg003_point5 - Radial_Velocity_Endo_seg003_point4)/Time_duration4

Radial_Velocity_Endo_seg003_a_min = min(c(Radial_Velocity_Endo_seg003_a1,Radial_Velocity_Endo_seg003_a2,Radial_Velocity_Endo_seg003_a3,Radial_Velocity_Endo_seg003_a4))

Radial_Velocity_Endo_seg003_a_max = max(c(Radial_Velocity_Endo_seg003_a1,Radial_Velocity_Endo_seg003_a2,Radial_Velocity_Endo_seg003_a3,Radial_Velocity_Endo_seg003_a4))

Radial_Velocity_Endo_seg004_point1 = as.numeric(velocity_data[11,col1])

Radial_Velocity_Endo_seg004_point2 = as.numeric(velocity_data[11,col2])

Radial_Velocity_Endo_seg004_point3 = as.numeric(velocity_data[11,col3])

Radial_Velocity_Endo_seg004_point4 = as.numeric(velocity_data[11,col4])

Radial_Velocity_Endo_seg004_point5 = as.numeric(velocity_data[11,col5])

Radial_Velocity_Endo_seg004_a1 = (Radial_Velocity_Endo_seg004_point2 - Radial_Velocity_Endo_seg004_point1)/Time_duration1

Radial_Velocity_Endo_seg004_a2 = (Radial_Velocity_Endo_seg004_point3 - Radial_Velocity_Endo_seg004_point2)/Time_duration2

Radial_Velocity_Endo_seg004_a3 = (Radial_Velocity_Endo_seg004_point4 - Radial_Velocity_Endo_seg004_point3)/Time_duration3

Radial_Velocity_Endo_seg004_a4 = (Radial_Velocity_Endo_seg004_point5 - Radial_Velocity_Endo_seg004_point4)/Time_duration4

Radial_Velocity_Endo_seg004_a_min = min(c(Radial_Velocity_Endo_seg004_a1,Radial_Velocity_Endo_seg004_a2,Radial_Velocity_Endo_seg004_a3,Radial_Velocity_Endo_seg004_a4))

Radial_Velocity_Endo_seg004_a_max = max(c(Radial_Velocity_Endo_seg004_a1,Radial_Velocity_Endo_seg004_a2,Radial_Velocity_Endo_seg004_a3,Radial_Velocity_Endo_seg004_a4))

Radial_Velocity_Endo_seg005_point1 = as.numeric(velocity_data[12,col1])

Radial_Velocity_Endo_seg005_point2 = as.numeric(velocity_data[12,col2])

Radial_Velocity_Endo_seg005_point3 = as.numeric(velocity_data[12,col3])

Radial_Velocity_Endo_seg005_point4 = as.numeric(velocity_data[12,col4])

Radial_Velocity_Endo_seg005_point5 = as.numeric(velocity_data[12,col5])

Radial_Velocity_Endo_seg005_a1 = (Radial_Velocity_Endo_seg005_point2 - Radial_Velocity_Endo_seg005_point1)/Time_duration1

Radial_Velocity_Endo_seg005_a2 = (Radial_Velocity_Endo_seg005_point3 - Radial_Velocity_Endo_seg005_point2)/Time_duration2

Radial_Velocity_Endo_seg005_a3 = (Radial_Velocity_Endo_seg005_point4 - Radial_Velocity_Endo_seg005_point3)/Time_duration3

Radial_Velocity_Endo_seg005_a4 = (Radial_Velocity_Endo_seg005_point5 - Radial_Velocity_Endo_seg005_point4)/Time_duration4

Radial_Velocity_Endo_seg005_a_min = min(c(Radial_Velocity_Endo_seg005_a1,Radial_Velocity_Endo_seg005_a2,Radial_Velocity_Endo_seg005_a3,Radial_Velocity_Endo_seg005_a4))

Radial_Velocity_Endo_seg005_a_max = max(c(Radial_Velocity_Endo_seg005_a1,Radial_Velocity_Endo_seg005_a2,Radial_Velocity_Endo_seg005_a3,Radial_Velocity_Endo_seg005_a4))

Radial_Velocity_Endo_seg006_point1 = as.numeric(velocity_data[13,col1])

Radial_Velocity_Endo_seg006_point2 = as.numeric(velocity_data[13,col2])

Radial_Velocity_Endo_seg006_point3 = as.numeric(velocity_data[13,col3])

Radial_Velocity_Endo_seg006_point4 = as.numeric(velocity_data[13,col4])

Radial_Velocity_Endo_seg006_point5 = as.numeric(velocity_data[13,col5])

Radial_Velocity_Endo_seg006_a1 = (Radial_Velocity_Endo_seg006_point2 - Radial_Velocity_Endo_seg006_point1)/Time_duration1

Radial_Velocity_Endo_seg006_a2 = (Radial_Velocity_Endo_seg006_point3 - Radial_Velocity_Endo_seg006_point2)/Time_duration2

Radial_Velocity_Endo_seg006_a3 = (Radial_Velocity_Endo_seg006_point4 - Radial_Velocity_Endo_seg006_point3)/Time_duration3

Radial_Velocity_Endo_seg006_a4 = (Radial_Velocity_Endo_seg006_point5 - Radial_Velocity_Endo_seg006_point4)/Time_duration4

Radial_Velocity_Endo_seg006_a_min = min(c(Radial_Velocity_Endo_seg006_a1,Radial_Velocity_Endo_seg006_a2,Radial_Velocity_Endo_seg006_a3,Radial_Velocity_Endo_seg006_a4))

Radial_Velocity_Endo_seg006_a_max = max(c(Radial_Velocity_Endo_seg006_a1,Radial_Velocity_Endo_seg006_a2,Radial_Velocity_Endo_seg006_a3,Radial_Velocity_Endo_seg006_a4))

##Average values per frame Radial Velocity Endo

Average_values_per_frame_Radial_Velocity_Endo_point1 = as.numeric(velocity_data[17,col1])

Average_values_per_frame_Radial_Velocity_Endo_point2 = as.numeric(velocity_data[17,col2])

Average_values_per_frame_Radial_Velocity_Endo_point3 = as.numeric(velocity_data[17,col3])

Average_values_per_frame_Radial_Velocity_Endo_point4 = as.numeric(velocity_data[17,col4])

Average_values_per_frame_Radial_Velocity_Endo_point5 = as.numeric(velocity_data[17,col5])

Average_values_per_frame_Radial_Velocity_Endo_a1 = (Average_values_per_frame_Radial_Velocity_Endo_point2 - Average_values_per_frame_Radial_Velocity_Endo_point1)/Time_duration1

Average_values_per_frame_Radial_Velocity_Endo_a2 = (Average_values_per_frame_Radial_Velocity_Endo_point3 - Average_values_per_frame_Radial_Velocity_Endo_point2)/Time_duration2

Average_values_per_frame_Radial_Velocity_Endo_a3 = (Average_values_per_frame_Radial_Velocity_Endo_point4 - Average_values_per_frame_Radial_Velocity_Endo_point3)/Time_duration3

Average_values_per_frame_Radial_Velocity_Endo_a4 = (Average_values_per_frame_Radial_Velocity_Endo_point5 - Average_values_per_frame_Radial_Velocity_Endo_point4)/Time_duration4

Average_values_per_frame_Radial_Velocity_Endo_a_min = min(c(Average_values_per_frame_Radial_Velocity_Endo_a1,Average_values_per_frame_Radial_Velocity_Endo_a2,Average_values_per_frame_Radial_Velocity_Endo_a3,Average_values_per_frame_Radial_Velocity_Endo_a4))

Average_values_per_frame_Radial_Velocity_Endo_a_max = max(c(Average_values_per_frame_Radial_Velocity_Endo_a1,Average_values_per_frame_Radial_Velocity_Endo_a2,Average_values_per_frame_Radial_Velocity_Endo_a3,Average_values_per_frame_Radial_Velocity_Endo_a4))

##Longitudinal_Velocity_Endo

Longitudinal_Velocity_Endo_seg001_point1 = as.numeric(velocity_data[21,col1])

Longitudinal_Velocity_Endo_seg001_point2 = as.numeric(velocity_data[21,col2])

Longitudinal_Velocity_Endo_seg001_point3 = as.numeric(velocity_data[21,col3])

Longitudinal_Velocity_Endo_seg001_point4 = as.numeric(velocity_data[21,col4])

Longitudinal_Velocity_Endo_seg001_point5 = as.numeric(velocity_data[21,col5])

Longitudinal_Velocity_Endo_seg001_a1 = (Longitudinal_Velocity_Endo_seg001_point2 - Longitudinal_Velocity_Endo_seg001_point1)/Time_duration1

Longitudinal_Velocity_Endo_seg001_a2 = (Longitudinal_Velocity_Endo_seg001_point3 - Longitudinal_Velocity_Endo_seg001_point2)/Time_duration2

Longitudinal_Velocity_Endo_seg001_a3 = (Longitudinal_Velocity_Endo_seg001_point4 - Longitudinal_Velocity_Endo_seg001_point3)/Time_duration3

Longitudinal_Velocity_Endo_seg001_a4 = (Longitudinal_Velocity_Endo_seg001_point5 - Longitudinal_Velocity_Endo_seg001_point4)/Time_duration4

Longitudinal_Velocity_Endo_seg001_a_min = min(c(Longitudinal_Velocity_Endo_seg001_a1,Longitudinal_Velocity_Endo_seg001_a2,Longitudinal_Velocity_Endo_seg001_a3,Longitudinal_Velocity_Endo_seg001_a4))

Longitudinal_Velocity_Endo_seg001_a_max = max(c(Longitudinal_Velocity_Endo_seg001_a1,Longitudinal_Velocity_Endo_seg001_a2,Longitudinal_Velocity_Endo_seg001_a3,Longitudinal_Velocity_Endo_seg001_a4))

Longitudinal_Velocity_Endo_seg002_point1 = as.numeric(velocity_data[22,col1])

Longitudinal_Velocity_Endo_seg002_point2 = as.numeric(velocity_data[22,col2])

Longitudinal_Velocity_Endo_seg002_point3 = as.numeric(velocity_data[22,col3])

Longitudinal_Velocity_Endo_seg002_point4 = as.numeric(velocity_data[22,col4])

Longitudinal_Velocity_Endo_seg002_point5 = as.numeric(velocity_data[22,col5])

Longitudinal_Velocity_Endo_seg002_a1 = (Longitudinal_Velocity_Endo_seg002_point2 - Longitudinal_Velocity_Endo_seg002_point1)/Time_duration1

Longitudinal_Velocity_Endo_seg002_a2 = (Longitudinal_Velocity_Endo_seg002_point3 - Longitudinal_Velocity_Endo_seg002_point2)/Time_duration2

Longitudinal_Velocity_Endo_seg002_a3 = (Longitudinal_Velocity_Endo_seg002_point4 - Longitudinal_Velocity_Endo_seg002_point3)/Time_duration3

Longitudinal_Velocity_Endo_seg002_a4 = (Longitudinal_Velocity_Endo_seg002_point5 - Longitudinal_Velocity_Endo_seg002_point4)/Time_duration4

Longitudinal_Velocity_Endo_seg002_a_min = min(c(Longitudinal_Velocity_Endo_seg002_a1,Longitudinal_Velocity_Endo_seg002_a2,Longitudinal_Velocity_Endo_seg002_a3,Longitudinal_Velocity_Endo_seg002_a4))

Longitudinal_Velocity_Endo_seg002_a_max = max(c(Longitudinal_Velocity_Endo_seg002_a1,Longitudinal_Velocity_Endo_seg002_a2,Longitudinal_Velocity_Endo_seg002_a3,Longitudinal_Velocity_Endo_seg002_a4))

Longitudinal_Velocity_Endo_seg003_point1 = as.numeric(velocity_data[23,col1])

Longitudinal_Velocity_Endo_seg003_point2 = as.numeric(velocity_data[23,col2])

Longitudinal_Velocity_Endo_seg003_point3 = as.numeric(velocity_data[23,col3])

Longitudinal_Velocity_Endo_seg003_point4 = as.numeric(velocity_data[23,col4])

Longitudinal_Velocity_Endo_seg003_point5 = as.numeric(velocity_data[23,col5])

Longitudinal_Velocity_Endo_seg003_a1 = (Longitudinal_Velocity_Endo_seg003_point2 - Longitudinal_Velocity_Endo_seg003_point1)/Time_duration1

Longitudinal_Velocity_Endo_seg003_a2 = (Longitudinal_Velocity_Endo_seg003_point3 - Longitudinal_Velocity_Endo_seg003_point2)/Time_duration2

Longitudinal_Velocity_Endo_seg003_a3 = (Longitudinal_Velocity_Endo_seg003_point4 - Longitudinal_Velocity_Endo_seg003_point3)/Time_duration3

Longitudinal_Velocity_Endo_seg003_a4 = (Longitudinal_Velocity_Endo_seg003_point5 - Longitudinal_Velocity_Endo_seg003_point4)/Time_duration4

Longitudinal_Velocity_Endo_seg003_a_min = min(c(Longitudinal_Velocity_Endo_seg003_a1,Longitudinal_Velocity_Endo_seg003_a2,Longitudinal_Velocity_Endo_seg003_a3,Longitudinal_Velocity_Endo_seg003_a4))

Longitudinal_Velocity_Endo_seg003_a_max = max(c(Longitudinal_Velocity_Endo_seg003_a1,Longitudinal_Velocity_Endo_seg003_a2,Longitudinal_Velocity_Endo_seg003_a3,Longitudinal_Velocity_Endo_seg003_a4))

Longitudinal_Velocity_Endo_seg004_point1 = as.numeric(velocity_data[24,col1])

Longitudinal_Velocity_Endo_seg004_point2 = as.numeric(velocity_data[24,col2])

Longitudinal_Velocity_Endo_seg004_point3 = as.numeric(velocity_data[24,col3])

Longitudinal_Velocity_Endo_seg004_point4 = as.numeric(velocity_data[24,col4])

Longitudinal_Velocity_Endo_seg004_point5 = as.numeric(velocity_data[24,col5])

Longitudinal_Velocity_Endo_seg004_a1 = (Longitudinal_Velocity_Endo_seg004_point2 - Longitudinal_Velocity_Endo_seg004_point1)/Time_duration1

Longitudinal_Velocity_Endo_seg004_a2 = (Longitudinal_Velocity_Endo_seg004_point3 - Longitudinal_Velocity_Endo_seg004_point2)/Time_duration2

Longitudinal_Velocity_Endo_seg004_a3 = (Longitudinal_Velocity_Endo_seg004_point4 - Longitudinal_Velocity_Endo_seg004_point3)/Time_duration3

Longitudinal_Velocity_Endo_seg004_a4 = (Longitudinal_Velocity_Endo_seg004_point5 - Longitudinal_Velocity_Endo_seg004_point4)/Time_duration4

Longitudinal_Velocity_Endo_seg004_a_min = min(c(Longitudinal_Velocity_Endo_seg004_a1,Longitudinal_Velocity_Endo_seg004_a2,Longitudinal_Velocity_Endo_seg004_a3,Longitudinal_Velocity_Endo_seg004_a4))

Longitudinal_Velocity_Endo_seg004_a_max = max(c(Longitudinal_Velocity_Endo_seg004_a1,Longitudinal_Velocity_Endo_seg004_a2,Longitudinal_Velocity_Endo_seg004_a3,Longitudinal_Velocity_Endo_seg004_a4))

Longitudinal_Velocity_Endo_seg005_point1 = as.numeric(velocity_data[25,col1])

Longitudinal_Velocity_Endo_seg005_point2 = as.numeric(velocity_data[25,col2])

Longitudinal_Velocity_Endo_seg005_point3 = as.numeric(velocity_data[25,col3])

Longitudinal_Velocity_Endo_seg005_point4 = as.numeric(velocity_data[25,col4])

Longitudinal_Velocity_Endo_seg005_point5 = as.numeric(velocity_data[25,col5])

Longitudinal_Velocity_Endo_seg005_a1 = (Longitudinal_Velocity_Endo_seg005_point2 - Longitudinal_Velocity_Endo_seg005_point1)/Time_duration1

Longitudinal_Velocity_Endo_seg005_a2 = (Longitudinal_Velocity_Endo_seg005_point3 - Longitudinal_Velocity_Endo_seg005_point2)/Time_duration2

Longitudinal_Velocity_Endo_seg005_a3 = (Longitudinal_Velocity_Endo_seg005_point4 - Longitudinal_Velocity_Endo_seg005_point3)/Time_duration3

Longitudinal_Velocity_Endo_seg005_a4 = (Longitudinal_Velocity_Endo_seg005_point5 - Longitudinal_Velocity_Endo_seg005_point4)/Time_duration4

Longitudinal_Velocity_Endo_seg005_a_min = min(c(Longitudinal_Velocity_Endo_seg005_a1,Longitudinal_Velocity_Endo_seg005_a2,Longitudinal_Velocity_Endo_seg005_a3,Longitudinal_Velocity_Endo_seg005_a4))

Longitudinal_Velocity_Endo_seg005_a_max = max(c(Longitudinal_Velocity_Endo_seg005_a1,Longitudinal_Velocity_Endo_seg005_a2,Longitudinal_Velocity_Endo_seg005_a3,Longitudinal_Velocity_Endo_seg005_a4))

Longitudinal_Velocity_Endo_seg006_point1 = as.numeric(velocity_data[26,col1])

Longitudinal_Velocity_Endo_seg006_point2 = as.numeric(velocity_data[26,col2])

Longitudinal_Velocity_Endo_seg006_point3 = as.numeric(velocity_data[26,col3])

Longitudinal_Velocity_Endo_seg006_point4 = as.numeric(velocity_data[26,col4])

Longitudinal_Velocity_Endo_seg006_point5 = as.numeric(velocity_data[26,col5])

Longitudinal_Velocity_Endo_seg006_a1 = (Longitudinal_Velocity_Endo_seg006_point2 - Longitudinal_Velocity_Endo_seg006_point1)/Time_duration1

Longitudinal_Velocity_Endo_seg006_a2 = (Longitudinal_Velocity_Endo_seg006_point3 - Longitudinal_Velocity_Endo_seg006_point2)/Time_duration2

Longitudinal_Velocity_Endo_seg006_a3 = (Longitudinal_Velocity_Endo_seg006_point4 - Longitudinal_Velocity_Endo_seg006_point3)/Time_duration3

Longitudinal_Velocity_Endo_seg006_a4 = (Longitudinal_Velocity_Endo_seg006_point5 - Longitudinal_Velocity_Endo_seg006_point4)/Time_duration4

Longitudinal_Velocity_Endo_seg006_a_min = min(c(Longitudinal_Velocity_Endo_seg006_a1,Longitudinal_Velocity_Endo_seg006_a2,Longitudinal_Velocity_Endo_seg006_a3,Longitudinal_Velocity_Endo_seg006_a4))

Longitudinal_Velocity_Endo_seg006_a_max = max(c(Longitudinal_Velocity_Endo_seg006_a1,Longitudinal_Velocity_Endo_seg006_a2,Longitudinal_Velocity_Endo_seg006_a3,Longitudinal_Velocity_Endo_seg006_a4))

##Average values per frame Longitudinal Velocity Endo

Average_values_per_frame_Longitudinal_Velocity_Endo_point1 = as.numeric(velocity_data[30,col1])

Average_values_per_frame_Longitudinal_Velocity_Endo_point2 = as.numeric(velocity_data[30,col2])

Average_values_per_frame_Longitudinal_Velocity_Endo_point3 = as.numeric(velocity_data[30,col3])

Average_values_per_frame_Longitudinal_Velocity_Endo_point4 = as.numeric(velocity_data[30,col4])

Average_values_per_frame_Longitudinal_Velocity_Endo_point5 = as.numeric(velocity_data[30,col5])

Average_values_per_frame_Longitudinal_Velocity_Endo_a1 = (Average_values_per_frame_Longitudinal_Velocity_Endo_point2 - Average_values_per_frame_Longitudinal_Velocity_Endo_point1)/Time_duration1

Average_values_per_frame_Longitudinal_Velocity_Endo_a2 = (Average_values_per_frame_Longitudinal_Velocity_Endo_point3 - Average_values_per_frame_Longitudinal_Velocity_Endo_point2)/Time_duration2

Average_values_per_frame_Longitudinal_Velocity_Endo_a3 = (Average_values_per_frame_Longitudinal_Velocity_Endo_point4 - Average_values_per_frame_Longitudinal_Velocity_Endo_point3)/Time_duration3

Average_values_per_frame_Longitudinal_Velocity_Endo_a4 = (Average_values_per_frame_Longitudinal_Velocity_Endo_point5 - Average_values_per_frame_Longitudinal_Velocity_Endo_point4)/Time_duration4

Average_values_per_frame_Longitudinal_Velocity_Endo_a_min = min(c(Average_values_per_frame_Longitudinal_Velocity_Endo_a1,Average_values_per_frame_Longitudinal_Velocity_Endo_a2,Average_values_per_frame_Longitudinal_Velocity_Endo_a3,Average_values_per_frame_Longitudinal_Velocity_Endo_a4))

Average_values_per_frame_Longitudinal_Velocity_Endo_a_max = max(c(Average_values_per_frame_Longitudinal_Velocity_Endo_a1,Average_values_per_frame_Longitudinal_Velocity_Endo_a2,Average_values_per_frame_Longitudinal_Velocity_Endo_a3,Average_values_per_frame_Longitudinal_Velocity_Endo_a4))

##Radial Velocity Epi

Radial_Velocity_Epi_seg001_point1 = as.numeric(velocity_data[34,col1])

Radial_Velocity_Epi_seg001_point2 = as.numeric(velocity_data[34,col2])

Radial_Velocity_Epi_seg001_point3 = as.numeric(velocity_data[34,col3])

Radial_Velocity_Epi_seg001_point4 = as.numeric(velocity_data[34,col4])

Radial_Velocity_Epi_seg001_point5 = as.numeric(velocity_data[34,col5])

Radial_Velocity_Epi_seg001_a1 = (Radial_Velocity_Epi_seg001_point2 - Radial_Velocity_Epi_seg001_point1)/Time_duration1

Radial_Velocity_Epi_seg001_a2 = (Radial_Velocity_Epi_seg001_point3 - Radial_Velocity_Epi_seg001_point2)/Time_duration2

Radial_Velocity_Epi_seg001_a3 = (Radial_Velocity_Epi_seg001_point4 - Radial_Velocity_Epi_seg001_point3)/Time_duration3

Radial_Velocity_Epi_seg001_a4 = (Radial_Velocity_Epi_seg001_point5 - Radial_Velocity_Epi_seg001_point4)/Time_duration4

Radial_Velocity_Epi_seg001_a_min = min(c(Radial_Velocity_Epi_seg001_a1,Radial_Velocity_Epi_seg001_a2,Radial_Velocity_Epi_seg001_a3,Radial_Velocity_Epi_seg001_a4))

Radial_Velocity_Epi_seg001_a_max = max(c(Radial_Velocity_Epi_seg001_a1,Radial_Velocity_Epi_seg001_a2,Radial_Velocity_Epi_seg001_a3,Radial_Velocity_Epi_seg001_a4))

Radial_Velocity_Epi_seg002_point1 = as.numeric(velocity_data[35,col1])

Radial_Velocity_Epi_seg002_point2 = as.numeric(velocity_data[35,col2])

Radial_Velocity_Epi_seg002_point3 = as.numeric(velocity_data[35,col3])

Radial_Velocity_Epi_seg002_point4 = as.numeric(velocity_data[35,col4])

Radial_Velocity_Epi_seg002_point5 = as.numeric(velocity_data[35,col5])

Radial_Velocity_Epi_seg002_a1 = (Radial_Velocity_Epi_seg002_point2 - Radial_Velocity_Epi_seg002_point1)/Time_duration1

Radial_Velocity_Epi_seg002_a2 = (Radial_Velocity_Epi_seg002_point3 - Radial_Velocity_Epi_seg002_point2)/Time_duration2

Radial_Velocity_Epi_seg002_a3 = (Radial_Velocity_Epi_seg002_point4 - Radial_Velocity_Epi_seg002_point3)/Time_duration3

Radial_Velocity_Epi_seg002_a4 = (Radial_Velocity_Epi_seg002_point5 - Radial_Velocity_Epi_seg002_point4)/Time_duration4

Radial_Velocity_Epi_seg002_a_min = min(c(Radial_Velocity_Epi_seg002_a1,Radial_Velocity_Epi_seg002_a2,Radial_Velocity_Epi_seg002_a3,Radial_Velocity_Epi_seg002_a4))

Radial_Velocity_Epi_seg002_a_max = max(c(Radial_Velocity_Epi_seg002_a1,Radial_Velocity_Epi_seg002_a2,Radial_Velocity_Epi_seg002_a3,Radial_Velocity_Epi_seg002_a4))

Radial_Velocity_Epi_seg003_point1 = as.numeric(velocity_data[36,col1])

Radial_Velocity_Epi_seg003_point2 = as.numeric(velocity_data[36,col2])

Radial_Velocity_Epi_seg003_point3 = as.numeric(velocity_data[36,col3])

Radial_Velocity_Epi_seg003_point4 = as.numeric(velocity_data[36,col4])

Radial_Velocity_Epi_seg003_point5 = as.numeric(velocity_data[36,col5])

Radial_Velocity_Epi_seg003_a1 = (Radial_Velocity_Epi_seg003_point2 - Radial_Velocity_Epi_seg003_point1)/Time_duration1

Radial_Velocity_Epi_seg003_a2 = (Radial_Velocity_Epi_seg003_point3 - Radial_Velocity_Epi_seg003_point2)/Time_duration2

Radial_Velocity_Epi_seg003_a3 = (Radial_Velocity_Epi_seg003_point4 - Radial_Velocity_Epi_seg003_point3)/Time_duration3

Radial_Velocity_Epi_seg003_a4 = (Radial_Velocity_Epi_seg003_point5 - Radial_Velocity_Epi_seg003_point4)/Time_duration4

Radial_Velocity_Epi_seg003_a_min = min(c(Radial_Velocity_Epi_seg003_a1,Radial_Velocity_Epi_seg003_a2,Radial_Velocity_Epi_seg003_a3,Radial_Velocity_Epi_seg003_a4))

Radial_Velocity_Epi_seg003_a_max = max(c(Radial_Velocity_Epi_seg003_a1,Radial_Velocity_Epi_seg003_a2,Radial_Velocity_Epi_seg003_a3,Radial_Velocity_Epi_seg003_a4))

Radial_Velocity_Epi_seg004_point1 = as.numeric(velocity_data[37,col1])

Radial_Velocity_Epi_seg004_point2 = as.numeric(velocity_data[37,col2])

Radial_Velocity_Epi_seg004_point3 = as.numeric(velocity_data[37,col3])

Radial_Velocity_Epi_seg004_point4 = as.numeric(velocity_data[37,col4])

Radial_Velocity_Epi_seg004_point5 = as.numeric(velocity_data[37,col5])

Radial_Velocity_Epi_seg004_a1 = (Radial_Velocity_Epi_seg004_point2 - Radial_Velocity_Epi_seg004_point1)/Time_duration1

Radial_Velocity_Epi_seg004_a2 = (Radial_Velocity_Epi_seg004_point3 - Radial_Velocity_Epi_seg004_point2)/Time_duration2

Radial_Velocity_Epi_seg004_a3 = (Radial_Velocity_Epi_seg004_point4 - Radial_Velocity_Epi_seg004_point3)/Time_duration3

Radial_Velocity_Epi_seg004_a4 = (Radial_Velocity_Epi_seg004_point5 - Radial_Velocity_Epi_seg004_point4)/Time_duration4

Radial_Velocity_Epi_seg004_a_min = min(c(Radial_Velocity_Epi_seg004_a1,Radial_Velocity_Epi_seg004_a2,Radial_Velocity_Epi_seg004_a3,Radial_Velocity_Epi_seg004_a4))

Radial_Velocity_Epi_seg004_a_max = max(c(Radial_Velocity_Epi_seg004_a1,Radial_Velocity_Epi_seg004_a2,Radial_Velocity_Epi_seg004_a3,Radial_Velocity_Epi_seg004_a4))

Radial_Velocity_Epi_seg005_point1 = as.numeric(velocity_data[38,col1])

Radial_Velocity_Epi_seg005_point2 = as.numeric(velocity_data[38,col2])

Radial_Velocity_Epi_seg005_point3 = as.numeric(velocity_data[38,col3])

Radial_Velocity_Epi_seg005_point4 = as.numeric(velocity_data[38,col4])

Radial_Velocity_Epi_seg005_point5 = as.numeric(velocity_data[38,col5])

Radial_Velocity_Epi_seg005_a1 = (Radial_Velocity_Epi_seg005_point2 - Radial_Velocity_Epi_seg005_point1)/Time_duration1

Radial_Velocity_Epi_seg005_a2 = (Radial_Velocity_Epi_seg005_point3 - Radial_Velocity_Epi_seg005_point2)/Time_duration2

Radial_Velocity_Epi_seg005_a3 = (Radial_Velocity_Epi_seg005_point4 - Radial_Velocity_Epi_seg005_point3)/Time_duration3

Radial_Velocity_Epi_seg005_a4 = (Radial_Velocity_Epi_seg005_point5 - Radial_Velocity_Epi_seg005_point4)/Time_duration4

Radial_Velocity_Epi_seg005_a_min = min(c(Radial_Velocity_Epi_seg005_a1,Radial_Velocity_Epi_seg005_a2,Radial_Velocity_Epi_seg005_a3,Radial_Velocity_Epi_seg005_a4))

Radial_Velocity_Epi_seg005_a_max = max(c(Radial_Velocity_Epi_seg005_a1,Radial_Velocity_Epi_seg005_a2,Radial_Velocity_Epi_seg005_a3,Radial_Velocity_Epi_seg005_a4))

Radial_Velocity_Epi_seg006_point1 = as.numeric(velocity_data[39,col1])

Radial_Velocity_Epi_seg006_point2 = as.numeric(velocity_data[39,col2])

Radial_Velocity_Epi_seg006_point3 = as.numeric(velocity_data[39,col3])

Radial_Velocity_Epi_seg006_point4 = as.numeric(velocity_data[39,col4])

Radial_Velocity_Epi_seg006_point5 = as.numeric(velocity_data[39,col5])

Radial_Velocity_Epi_seg006_a1 = (Radial_Velocity_Epi_seg006_point2 - Radial_Velocity_Epi_seg006_point1)/Time_duration1

Radial_Velocity_Epi_seg006_a2 = (Radial_Velocity_Epi_seg006_point3 - Radial_Velocity_Epi_seg006_point2)/Time_duration2

Radial_Velocity_Epi_seg006_a3 = (Radial_Velocity_Epi_seg006_point4 - Radial_Velocity_Epi_seg006_point3)/Time_duration3

Radial_Velocity_Epi_seg006_a4 = (Radial_Velocity_Epi_seg006_point5 - Radial_Velocity_Epi_seg006_point4)/Time_duration4

Radial_Velocity_Epi_seg006_a_min = min(c(Radial_Velocity_Epi_seg006_a1,Radial_Velocity_Epi_seg006_a2,Radial_Velocity_Epi_seg006_a3,Radial_Velocity_Epi_seg006_a4))

Radial_Velocity_Epi_seg006_a_max = max(c(Radial_Velocity_Epi_seg006_a1,Radial_Velocity_Epi_seg006_a2,Radial_Velocity_Epi_seg006_a3,Radial_Velocity_Epi_seg006_a4))

##Average values per frame Radial Velocity Epi

Average_values_per_frame_Radial_Velocity_Epi_point1 = as.numeric(velocity_data[43,col1])

Average_values_per_frame_Radial_Velocity_Epi_point2 = as.numeric(velocity_data[43,col2])

Average_values_per_frame_Radial_Velocity_Epi_point3 = as.numeric(velocity_data[43,col3])

Average_values_per_frame_Radial_Velocity_Epi_point4 = as.numeric(velocity_data[43,col4])

Average_values_per_frame_Radial_Velocity_Epi_point5 = as.numeric(velocity_data[43,col5])

Average_values_per_frame_Radial_Velocity_Epi_a1 = (Average_values_per_frame_Radial_Velocity_Epi_point2 - Average_values_per_frame_Radial_Velocity_Epi_point1)/Time_duration1

Average_values_per_frame_Radial_Velocity_Epi_a2 = (Average_values_per_frame_Radial_Velocity_Epi_point3 - Average_values_per_frame_Radial_Velocity_Epi_point2)/Time_duration2

Average_values_per_frame_Radial_Velocity_Epi_a3 = (Average_values_per_frame_Radial_Velocity_Epi_point4 - Average_values_per_frame_Radial_Velocity_Epi_point3)/Time_duration3

Average_values_per_frame_Radial_Velocity_Epi_a4 = (Average_values_per_frame_Radial_Velocity_Epi_point5 - Average_values_per_frame_Radial_Velocity_Epi_point4)/Time_duration4

Average_values_per_frame_Radial_Velocity_Epi_a_min = min(c(Average_values_per_frame_Radial_Velocity_Epi_a1,Average_values_per_frame_Radial_Velocity_Epi_a2,Average_values_per_frame_Radial_Velocity_Epi_a3,Average_values_per_frame_Radial_Velocity_Epi_a4))

Average_values_per_frame_Radial_Velocity_Epi_a_max = max(c(Average_values_per_frame_Radial_Velocity_Epi_a1,Average_values_per_frame_Radial_Velocity_Epi_a2,Average_values_per_frame_Radial_Velocity_Epi_a3,Average_values_per_frame_Radial_Velocity_Epi_a4))

##Longitudinal Velocity Epi

Longitudinal_Velocity_Epi_seg001_point1 = as.numeric(velocity_data[47,col1])

Longitudinal_Velocity_Epi_seg001_point2 = as.numeric(velocity_data[47,col2])

Longitudinal_Velocity_Epi_seg001_point3 = as.numeric(velocity_data[47,col3])

Longitudinal_Velocity_Epi_seg001_point4 = as.numeric(velocity_data[47,col4])

Longitudinal_Velocity_Epi_seg001_point5 = as.numeric(velocity_data[47,col5])

Longitudinal_Velocity_Epi_seg001_a1 = (Longitudinal_Velocity_Epi_seg001_point2 - Longitudinal_Velocity_Epi_seg001_point1)/Time_duration1

Longitudinal_Velocity_Epi_seg001_a2 = (Longitudinal_Velocity_Epi_seg001_point3 - Longitudinal_Velocity_Epi_seg001_point2)/Time_duration2

Longitudinal_Velocity_Epi_seg001_a3 = (Longitudinal_Velocity_Epi_seg001_point4 - Longitudinal_Velocity_Epi_seg001_point3)/Time_duration3

Longitudinal_Velocity_Epi_seg001_a4 = (Longitudinal_Velocity_Epi_seg001_point5 - Longitudinal_Velocity_Epi_seg001_point4)/Time_duration4

Longitudinal_Velocity_Epi_seg001_a_min = min(c(Longitudinal_Velocity_Epi_seg001_a1,Longitudinal_Velocity_Epi_seg001_a2,Longitudinal_Velocity_Epi_seg001_a3,Longitudinal_Velocity_Epi_seg001_a4))

Longitudinal_Velocity_Epi_seg001_a_max = max(c(Longitudinal_Velocity_Epi_seg001_a1,Longitudinal_Velocity_Epi_seg001_a2,Longitudinal_Velocity_Epi_seg001_a3,Longitudinal_Velocity_Epi_seg001_a4))

Longitudinal_Velocity_Epi_seg002_point1 = as.numeric(velocity_data[48,col1])

Longitudinal_Velocity_Epi_seg002_point2 = as.numeric(velocity_data[48,col2])

Longitudinal_Velocity_Epi_seg002_point3 = as.numeric(velocity_data[48,col3])

Longitudinal_Velocity_Epi_seg002_point4 = as.numeric(velocity_data[48,col4])

Longitudinal_Velocity_Epi_seg002_point5 = as.numeric(velocity_data[48,col5])

Longitudinal_Velocity_Epi_seg002_a1 = (Longitudinal_Velocity_Epi_seg002_point2 - Longitudinal_Velocity_Epi_seg002_point1)/Time_duration1

Longitudinal_Velocity_Epi_seg002_a2 = (Longitudinal_Velocity_Epi_seg002_point3 - Longitudinal_Velocity_Epi_seg002_point2)/Time_duration2

Longitudinal_Velocity_Epi_seg002_a3 = (Longitudinal_Velocity_Epi_seg002_point4 - Longitudinal_Velocity_Epi_seg002_point3)/Time_duration3

Longitudinal_Velocity_Epi_seg002_a4 = (Longitudinal_Velocity_Epi_seg002_point5 - Longitudinal_Velocity_Epi_seg002_point4)/Time_duration4

Longitudinal_Velocity_Epi_seg002_a_min = min(c(Longitudinal_Velocity_Epi_seg002_a1,Longitudinal_Velocity_Epi_seg002_a2,Longitudinal_Velocity_Epi_seg002_a3,Longitudinal_Velocity_Epi_seg002_a4))

Longitudinal_Velocity_Epi_seg002_a_max = max(c(Longitudinal_Velocity_Epi_seg002_a1,Longitudinal_Velocity_Epi_seg002_a2,Longitudinal_Velocity_Epi_seg002_a3,Longitudinal_Velocity_Epi_seg002_a4))

Longitudinal_Velocity_Epi_seg003_point1 = as.numeric(velocity_data[49,col1])

Longitudinal_Velocity_Epi_seg003_point2 = as.numeric(velocity_data[49,col2])

Longitudinal_Velocity_Epi_seg003_point3 = as.numeric(velocity_data[49,col3])

Longitudinal_Velocity_Epi_seg003_point4 = as.numeric(velocity_data[49,col4])

Longitudinal_Velocity_Epi_seg003_point5 = as.numeric(velocity_data[49,col5])

Longitudinal_Velocity_Epi_seg003_a1 = (Longitudinal_Velocity_Epi_seg003_point2 - Longitudinal_Velocity_Epi_seg003_point1)/Time_duration1

Longitudinal_Velocity_Epi_seg003_a2 = (Longitudinal_Velocity_Epi_seg003_point3 - Longitudinal_Velocity_Epi_seg003_point2)/Time_duration2

Longitudinal_Velocity_Epi_seg003_a3 = (Longitudinal_Velocity_Epi_seg003_point4 - Longitudinal_Velocity_Epi_seg003_point3)/Time_duration3

Longitudinal_Velocity_Epi_seg003_a4 = (Longitudinal_Velocity_Epi_seg003_point5 - Longitudinal_Velocity_Epi_seg003_point4)/Time_duration4

Longitudinal_Velocity_Epi_seg003_a_min = min(c(Longitudinal_Velocity_Epi_seg003_a1,Longitudinal_Velocity_Epi_seg003_a2,Longitudinal_Velocity_Epi_seg003_a3,Longitudinal_Velocity_Epi_seg003_a4))

Longitudinal_Velocity_Epi_seg003_a_max = max(c(Longitudinal_Velocity_Epi_seg003_a1,Longitudinal_Velocity_Epi_seg003_a2,Longitudinal_Velocity_Epi_seg003_a3,Longitudinal_Velocity_Epi_seg003_a4))

Longitudinal_Velocity_Epi_seg004_point1 = as.numeric(velocity_data[50,col1])

Longitudinal_Velocity_Epi_seg004_point2 = as.numeric(velocity_data[50,col2])

Longitudinal_Velocity_Epi_seg004_point3 = as.numeric(velocity_data[50,col3])

Longitudinal_Velocity_Epi_seg004_point4 = as.numeric(velocity_data[50,col4])

Longitudinal_Velocity_Epi_seg004_point5 = as.numeric(velocity_data[50,col5])

Longitudinal_Velocity_Epi_seg004_a1 = (Longitudinal_Velocity_Epi_seg004_point2 - Longitudinal_Velocity_Epi_seg004_point1)/Time_duration1

Longitudinal_Velocity_Epi_seg004_a2 = (Longitudinal_Velocity_Epi_seg004_point3 - Longitudinal_Velocity_Epi_seg004_point2)/Time_duration2

Longitudinal_Velocity_Epi_seg004_a3 = (Longitudinal_Velocity_Epi_seg004_point4 - Longitudinal_Velocity_Epi_seg004_point3)/Time_duration3

Longitudinal_Velocity_Epi_seg004_a4 = (Longitudinal_Velocity_Epi_seg004_point5 - Longitudinal_Velocity_Epi_seg004_point4)/Time_duration4

Longitudinal_Velocity_Epi_seg004_a_min = min(c(Longitudinal_Velocity_Epi_seg004_a1,Longitudinal_Velocity_Epi_seg004_a2,Longitudinal_Velocity_Epi_seg004_a3,Longitudinal_Velocity_Epi_seg004_a4))

Longitudinal_Velocity_Epi_seg004_a_max = max(c(Longitudinal_Velocity_Epi_seg004_a1,Longitudinal_Velocity_Epi_seg004_a2,Longitudinal_Velocity_Epi_seg004_a3,Longitudinal_Velocity_Epi_seg004_a4))

Longitudinal_Velocity_Epi_seg005_point1 = as.numeric(velocity_data[51,col1])

Longitudinal_Velocity_Epi_seg005_point2 = as.numeric(velocity_data[51,col2])

Longitudinal_Velocity_Epi_seg005_point3 = as.numeric(velocity_data[51,col3])

Longitudinal_Velocity_Epi_seg005_point4 = as.numeric(velocity_data[51,col4])

Longitudinal_Velocity_Epi_seg005_point5 = as.numeric(velocity_data[51,col5])

Longitudinal_Velocity_Epi_seg005_a1 = (Longitudinal_Velocity_Epi_seg005_point2 - Longitudinal_Velocity_Epi_seg005_point1)/Time_duration1

Longitudinal_Velocity_Epi_seg005_a2 = (Longitudinal_Velocity_Epi_seg005_point3 - Longitudinal_Velocity_Epi_seg005_point2)/Time_duration2

Longitudinal_Velocity_Epi_seg005_a3 = (Longitudinal_Velocity_Epi_seg005_point4 - Longitudinal_Velocity_Epi_seg005_point3)/Time_duration3

Longitudinal_Velocity_Epi_seg005_a4 = (Longitudinal_Velocity_Epi_seg005_point5 - Longitudinal_Velocity_Epi_seg005_point4)/Time_duration4

Longitudinal_Velocity_Epi_seg005_a_min = min(c(Longitudinal_Velocity_Epi_seg005_a1,Longitudinal_Velocity_Epi_seg005_a2,Longitudinal_Velocity_Epi_seg005_a3,Longitudinal_Velocity_Epi_seg005_a4))

Longitudinal_Velocity_Epi_seg005_a_max = max(c(Longitudinal_Velocity_Epi_seg005_a1,Longitudinal_Velocity_Epi_seg005_a2,Longitudinal_Velocity_Epi_seg005_a3,Longitudinal_Velocity_Epi_seg005_a4))

Longitudinal_Velocity_Epi_seg006_point1 = as.numeric(velocity_data[52,col1])

Longitudinal_Velocity_Epi_seg006_point2 = as.numeric(velocity_data[52,col2])

Longitudinal_Velocity_Epi_seg006_point3 = as.numeric(velocity_data[52,col3])

Longitudinal_Velocity_Epi_seg006_point4 = as.numeric(velocity_data[52,col4])

Longitudinal_Velocity_Epi_seg006_point5 = as.numeric(velocity_data[52,col5])

Longitudinal_Velocity_Epi_seg006_a1 = (Longitudinal_Velocity_Epi_seg006_point2 - Longitudinal_Velocity_Epi_seg006_point1)/Time_duration1

Longitudinal_Velocity_Epi_seg006_a2 = (Longitudinal_Velocity_Epi_seg006_point3 - Longitudinal_Velocity_Epi_seg006_point2)/Time_duration2

Longitudinal_Velocity_Epi_seg006_a3 = (Longitudinal_Velocity_Epi_seg006_point4 - Longitudinal_Velocity_Epi_seg006_point3)/Time_duration3

Longitudinal_Velocity_Epi_seg006_a4 = (Longitudinal_Velocity_Epi_seg006_point5 - Longitudinal_Velocity_Epi_seg006_point4)/Time_duration4

Longitudinal_Velocity_Epi_seg006_a_min = min(c(Longitudinal_Velocity_Epi_seg006_a1,Longitudinal_Velocity_Epi_seg006_a2,Longitudinal_Velocity_Epi_seg006_a3,Longitudinal_Velocity_Epi_seg006_a4))

Longitudinal_Velocity_Epi_seg006_a_max = max(c(Longitudinal_Velocity_Epi_seg006_a1,Longitudinal_Velocity_Epi_seg006_a2,Longitudinal_Velocity_Epi_seg006_a3,Longitudinal_Velocity_Epi_seg006_a4))

##Average values per frame Longitudinal Velocity Epi

Average_values_per_frame_Longitudinal_Velocity_Epi_point1 = as.numeric(velocity_data[56,col1])

Average_values_per_frame_Longitudinal_Velocity_Epi_point2 = as.numeric(velocity_data[56,col2])

Average_values_per_frame_Longitudinal_Velocity_Epi_point3 = as.numeric(velocity_data[56,col3])

Average_values_per_frame_Longitudinal_Velocity_Epi_point4 = as.numeric(velocity_data[56,col4])

Average_values_per_frame_Longitudinal_Velocity_Epi_point5 = as.numeric(velocity_data[56,col5])

Average_values_per_frame_Longitudinal_Velocity_Epi_a1 = (Average_values_per_frame_Longitudinal_Velocity_Epi_point2 - Average_values_per_frame_Longitudinal_Velocity_Epi_point1)/Time_duration1

Average_values_per_frame_Longitudinal_Velocity_Epi_a2 = (Average_values_per_frame_Longitudinal_Velocity_Epi_point3 - Average_values_per_frame_Longitudinal_Velocity_Epi_point2)/Time_duration2

Average_values_per_frame_Longitudinal_Velocity_Epi_a3 = (Average_values_per_frame_Longitudinal_Velocity_Epi_point4 - Average_values_per_frame_Longitudinal_Velocity_Epi_point3)/Time_duration3

Average_values_per_frame_Longitudinal_Velocity_Epi_a4 = (Average_values_per_frame_Longitudinal_Velocity_Epi_point5 - Average_values_per_frame_Longitudinal_Velocity_Epi_point4)/Time_duration4

Average_values_per_frame_Longitudinal_Velocity_Epi_a_min = min(c(Average_values_per_frame_Longitudinal_Velocity_Epi_a1,Average_values_per_frame_Longitudinal_Velocity_Epi_a2,Average_values_per_frame_Longitudinal_Velocity_Epi_a3,Average_values_per_frame_Longitudinal_Velocity_Epi_a4))

Average_values_per_frame_Longitudinal_Velocity_Epi_a_max = max(c(Average_values_per_frame_Longitudinal_Velocity_Epi_a1,Average_values_per_frame_Longitudinal_Velocity_Epi_a2,Average_values_per_frame_Longitudinal_Velocity_Epi_a3,Average_values_per_frame_Longitudinal_Velocity_Epi_a4))

#Displacement

Displacement_data <- read.xlsx(i,sheetIndex = 3, header = F)

colnames(Displacement_data) <- Displacement_data[3,]

name1 = paste("fr0",time3,sep = "")

col5 = grep(name1,colnames(Displacement_data))

col4 = col5-1

col3 = col5-2

col2 = col5-3

col1 = col5-4

Timepoint1 = as.numeric(Displacement_data[4,col1])

Timepoint2 = as.numeric(Displacement_data[4,col2])

Timepoint3 = as.numeric(Displacement_data[4,col3])

Timepoint4 = as.numeric(Displacement_data[4,col4])

Timepoint5 = as.numeric(Displacement_data[4,col5])

Time_duration1 = Timepoint2 - Timepoint1

Time_duration2 = Timepoint3 - Timepoint2

Time_duration3 = Timepoint4 - Timepoint3

Time_duration4 = Timepoint5 - Timepoint4

##Radial Displacement Endo

Radial_Displacement_Endo_seg001_point1 = as.numeric(Displacement_data[8,col1])

Radial_Displacement_Endo_seg001_point2 = as.numeric(Displacement_data[8,col2])

Radial_Displacement_Endo_seg001_point3 = as.numeric(Displacement_data[8,col3])

Radial_Displacement_Endo_seg001_point4 = as.numeric(Displacement_data[8,col4])

Radial_Displacement_Endo_seg001_point5 = as.numeric(Displacement_data[8,col5])

Radial_Displacement_Endo_seg001_a1 = (Radial_Displacement_Endo_seg001_point2 - Radial_Displacement_Endo_seg001_point1)/Time_duration1

Radial_Displacement_Endo_seg001_a2 = (Radial_Displacement_Endo_seg001_point3 - Radial_Displacement_Endo_seg001_point2)/Time_duration2

Radial_Displacement_Endo_seg001_a3 = (Radial_Displacement_Endo_seg001_point4 - Radial_Displacement_Endo_seg001_point3)/Time_duration3

Radial_Displacement_Endo_seg001_a4 = (Radial_Displacement_Endo_seg001_point5 - Radial_Displacement_Endo_seg001_point4)/Time_duration4

Radial_Displacement_Endo_seg001_a_min = min(c(Radial_Displacement_Endo_seg001_a1,Radial_Displacement_Endo_seg001_a2,Radial_Displacement_Endo_seg001_a3,Radial_Displacement_Endo_seg001_a4))

Radial_Displacement_Endo_seg001_a_max = max(c(Radial_Displacement_Endo_seg001_a1,Radial_Displacement_Endo_seg001_a2,Radial_Displacement_Endo_seg001_a3,Radial_Displacement_Endo_seg001_a4))

Radial_Displacement_Endo_seg002_point1 = as.numeric(Displacement_data[9,col1])

Radial_Displacement_Endo_seg002_point2 = as.numeric(Displacement_data[9,col2])

Radial_Displacement_Endo_seg002_point3 = as.numeric(Displacement_data[9,col3])

Radial_Displacement_Endo_seg002_point4 = as.numeric(Displacement_data[9,col4])

Radial_Displacement_Endo_seg002_point5 = as.numeric(Displacement_data[9,col5])

Radial_Displacement_Endo_seg002_a1 = (Radial_Displacement_Endo_seg002_point2 - Radial_Displacement_Endo_seg002_point1)/Time_duration1

Radial_Displacement_Endo_seg002_a2 = (Radial_Displacement_Endo_seg002_point3 - Radial_Displacement_Endo_seg002_point2)/Time_duration2

Radial_Displacement_Endo_seg002_a3 = (Radial_Displacement_Endo_seg002_point4 - Radial_Displacement_Endo_seg002_point3)/Time_duration3

Radial_Displacement_Endo_seg002_a4 = (Radial_Displacement_Endo_seg002_point5 - Radial_Displacement_Endo_seg002_point4)/Time_duration4

Radial_Displacement_Endo_seg002_a_min = min(c(Radial_Displacement_Endo_seg002_a1,Radial_Displacement_Endo_seg002_a2,Radial_Displacement_Endo_seg002_a3,Radial_Displacement_Endo_seg002_a4))

Radial_Displacement_Endo_seg002_a_max = max(c(Radial_Displacement_Endo_seg002_a1,Radial_Displacement_Endo_seg002_a2,Radial_Displacement_Endo_seg002_a3,Radial_Displacement_Endo_seg002_a4))

Radial_Displacement_Endo_seg003_point1 = as.numeric(Displacement_data[10,col1])

Radial_Displacement_Endo_seg003_point2 = as.numeric(Displacement_data[10,col2])

Radial_Displacement_Endo_seg003_point3 = as.numeric(Displacement_data[10,col3])

Radial_Displacement_Endo_seg003_point4 = as.numeric(Displacement_data[10,col4])

Radial_Displacement_Endo_seg003_point5 = as.numeric(Displacement_data[10,col5])

Radial_Displacement_Endo_seg003_a1 = (Radial_Displacement_Endo_seg003_point2 - Radial_Displacement_Endo_seg003_point1)/Time_duration1

Radial_Displacement_Endo_seg003_a2 = (Radial_Displacement_Endo_seg003_point3 - Radial_Displacement_Endo_seg003_point2)/Time_duration2

Radial_Displacement_Endo_seg003_a3 = (Radial_Displacement_Endo_seg003_point4 - Radial_Displacement_Endo_seg003_point3)/Time_duration3

Radial_Displacement_Endo_seg003_a4 = (Radial_Displacement_Endo_seg003_point5 - Radial_Displacement_Endo_seg003_point4)/Time_duration4

Radial_Displacement_Endo_seg003_a_min = min(c(Radial_Displacement_Endo_seg003_a1,Radial_Displacement_Endo_seg003_a2,Radial_Displacement_Endo_seg003_a3,Radial_Displacement_Endo_seg003_a4))

Radial_Displacement_Endo_seg003_a_max = max(c(Radial_Displacement_Endo_seg003_a1,Radial_Displacement_Endo_seg003_a2,Radial_Displacement_Endo_seg003_a3,Radial_Displacement_Endo_seg003_a4))

Radial_Displacement_Endo_seg004_point1 = as.numeric(Displacement_data[11,col1])

Radial_Displacement_Endo_seg004_point2 = as.numeric(Displacement_data[11,col2])

Radial_Displacement_Endo_seg004_point3 = as.numeric(Displacement_data[11,col3])

Radial_Displacement_Endo_seg004_point4 = as.numeric(Displacement_data[11,col4])

Radial_Displacement_Endo_seg004_point5 = as.numeric(Displacement_data[11,col5])

Radial_Displacement_Endo_seg004_a1 = (Radial_Displacement_Endo_seg004_point2 - Radial_Displacement_Endo_seg004_point1)/Time_duration1

Radial_Displacement_Endo_seg004_a2 = (Radial_Displacement_Endo_seg004_point3 - Radial_Displacement_Endo_seg004_point2)/Time_duration2

Radial_Displacement_Endo_seg004_a3 = (Radial_Displacement_Endo_seg004_point4 - Radial_Displacement_Endo_seg004_point3)/Time_duration3

Radial_Displacement_Endo_seg004_a4 = (Radial_Displacement_Endo_seg004_point5 - Radial_Displacement_Endo_seg004_point4)/Time_duration4

Radial_Displacement_Endo_seg004_a_min = min(c(Radial_Displacement_Endo_seg004_a1,Radial_Displacement_Endo_seg004_a2,Radial_Displacement_Endo_seg004_a3,Radial_Displacement_Endo_seg004_a4))

Radial_Displacement_Endo_seg004_a_max = max(c(Radial_Displacement_Endo_seg004_a1,Radial_Displacement_Endo_seg004_a2,Radial_Displacement_Endo_seg004_a3,Radial_Displacement_Endo_seg004_a4))

Radial_Displacement_Endo_seg005_point1 = as.numeric(Displacement_data[12,col1])

Radial_Displacement_Endo_seg005_point2 = as.numeric(Displacement_data[12,col2])

Radial_Displacement_Endo_seg005_point3 = as.numeric(Displacement_data[12,col3])

Radial_Displacement_Endo_seg005_point4 = as.numeric(Displacement_data[12,col4])

Radial_Displacement_Endo_seg005_point5 = as.numeric(Displacement_data[12,col5])

Radial_Displacement_Endo_seg005_a1 = (Radial_Displacement_Endo_seg005_point2 - Radial_Displacement_Endo_seg005_point1)/Time_duration1

Radial_Displacement_Endo_seg005_a2 = (Radial_Displacement_Endo_seg005_point3 - Radial_Displacement_Endo_seg005_point2)/Time_duration2

Radial_Displacement_Endo_seg005_a3 = (Radial_Displacement_Endo_seg005_point4 - Radial_Displacement_Endo_seg005_point3)/Time_duration3

Radial_Displacement_Endo_seg005_a4 = (Radial_Displacement_Endo_seg005_point5 - Radial_Displacement_Endo_seg005_point4)/Time_duration4

Radial_Displacement_Endo_seg005_a_min = min(c(Radial_Displacement_Endo_seg005_a1,Radial_Displacement_Endo_seg005_a2,Radial_Displacement_Endo_seg005_a3,Radial_Displacement_Endo_seg005_a4))

Radial_Displacement_Endo_seg005_a_max = max(c(Radial_Displacement_Endo_seg005_a1,Radial_Displacement_Endo_seg005_a2,Radial_Displacement_Endo_seg005_a3,Radial_Displacement_Endo_seg005_a4))

Radial_Displacement_Endo_seg006_point1 = as.numeric(Displacement_data[13,col1])

Radial_Displacement_Endo_seg006_point2 = as.numeric(Displacement_data[13,col2])

Radial_Displacement_Endo_seg006_point3 = as.numeric(Displacement_data[13,col3])

Radial_Displacement_Endo_seg006_point4 = as.numeric(Displacement_data[13,col4])

Radial_Displacement_Endo_seg006_point5 = as.numeric(Displacement_data[13,col5])

Radial_Displacement_Endo_seg006_a1 = (Radial_Displacement_Endo_seg006_point2 - Radial_Displacement_Endo_seg006_point1)/Time_duration1

Radial_Displacement_Endo_seg006_a2 = (Radial_Displacement_Endo_seg006_point3 - Radial_Displacement_Endo_seg006_point2)/Time_duration2

Radial_Displacement_Endo_seg006_a3 = (Radial_Displacement_Endo_seg006_point4 - Radial_Displacement_Endo_seg006_point3)/Time_duration3

Radial_Displacement_Endo_seg006_a4 = (Radial_Displacement_Endo_seg006_point5 - Radial_Displacement_Endo_seg006_point4)/Time_duration4

Radial_Displacement_Endo_seg006_a_min = min(c(Radial_Displacement_Endo_seg006_a1,Radial_Displacement_Endo_seg006_a2,Radial_Displacement_Endo_seg006_a3,Radial_Displacement_Endo_seg006_a4))

Radial_Displacement_Endo_seg006_a_max = max(c(Radial_Displacement_Endo_seg006_a1,Radial_Displacement_Endo_seg006_a2,Radial_Displacement_Endo_seg006_a3,Radial_Displacement_Endo_seg006_a4))

##Average_values_per_frame_Radial_Displacement_Endo

Average_values_per_frame_Radial_Displacement_Endo_point1 = as.numeric(Displacement_data[17,col1])

Average_values_per_frame_Radial_Displacement_Endo_point2 = as.numeric(Displacement_data[17,col2])

Average_values_per_frame_Radial_Displacement_Endo_point3 = as.numeric(Displacement_data[17,col3])

Average_values_per_frame_Radial_Displacement_Endo_point4 = as.numeric(Displacement_data[17,col4])

Average_values_per_frame_Radial_Displacement_Endo_point5 = as.numeric(Displacement_data[17,col5])

Average_values_per_frame_Radial_Displacement_Endo_a1 = (Average_values_per_frame_Radial_Displacement_Endo_point2 - Average_values_per_frame_Radial_Displacement_Endo_point1)/Time_duration1

Average_values_per_frame_Radial_Displacement_Endo_a2 = (Average_values_per_frame_Radial_Displacement_Endo_point3 - Average_values_per_frame_Radial_Displacement_Endo_point2)/Time_duration2

Average_values_per_frame_Radial_Displacement_Endo_a3 = (Average_values_per_frame_Radial_Displacement_Endo_point4 - Average_values_per_frame_Radial_Displacement_Endo_point3)/Time_duration3

Average_values_per_frame_Radial_Displacement_Endo_a4 = (Average_values_per_frame_Radial_Displacement_Endo_point5 - Average_values_per_frame_Radial_Displacement_Endo_point4)/Time_duration4

Average_values_per_frame_Radial_Displacement_Endo_a_min = min(c(Average_values_per_frame_Radial_Displacement_Endo_a1,Average_values_per_frame_Radial_Displacement_Endo_a2,Average_values_per_frame_Radial_Displacement_Endo_a3,Average_values_per_frame_Radial_Displacement_Endo_a4))

Average_values_per_frame_Radial_Displacement_Endo_a_max = max(c(Average_values_per_frame_Radial_Displacement_Endo_a1,Average_values_per_frame_Radial_Displacement_Endo_a2,Average_values_per_frame_Radial_Displacement_Endo_a3,Average_values_per_frame_Radial_Displacement_Endo_a4))

##Longitudinal_Displacement_Endo

Longitudinal_Displacement_Endo_seg001_point1 = as.numeric(Displacement_data[21,col1])

Longitudinal_Displacement_Endo_seg001_point2 = as.numeric(Displacement_data[21,col2])

Longitudinal_Displacement_Endo_seg001_point3 = as.numeric(Displacement_data[21,col3])

Longitudinal_Displacement_Endo_seg001_point4 = as.numeric(Displacement_data[21,col4])

Longitudinal_Displacement_Endo_seg001_point5 = as.numeric(Displacement_data[21,col5])

Longitudinal_Displacement_Endo_seg001_a1 = (Longitudinal_Displacement_Endo_seg001_point2 - Longitudinal_Displacement_Endo_seg001_point1)/Time_duration1

Longitudinal_Displacement_Endo_seg001_a2 = (Longitudinal_Displacement_Endo_seg001_point3 - Longitudinal_Displacement_Endo_seg001_point2)/Time_duration2

Longitudinal_Displacement_Endo_seg001_a3 = (Longitudinal_Displacement_Endo_seg001_point4 - Longitudinal_Displacement_Endo_seg001_point3)/Time_duration3

Longitudinal_Displacement_Endo_seg001_a4 = (Longitudinal_Displacement_Endo_seg001_point5 - Longitudinal_Displacement_Endo_seg001_point4)/Time_duration4

Longitudinal_Displacement_Endo_seg001_a_min = min(c(Longitudinal_Displacement_Endo_seg001_a1,Longitudinal_Displacement_Endo_seg001_a2,Longitudinal_Displacement_Endo_seg001_a3,Longitudinal_Displacement_Endo_seg001_a4))

Longitudinal_Displacement_Endo_seg001_a_max = max(c(Longitudinal_Displacement_Endo_seg001_a1,Longitudinal_Displacement_Endo_seg001_a2,Longitudinal_Displacement_Endo_seg001_a3,Longitudinal_Displacement_Endo_seg001_a4))

Longitudinal_Displacement_Endo_seg002_point1 = as.numeric(Displacement_data[22,col1])

Longitudinal_Displacement_Endo_seg002_point2 = as.numeric(Displacement_data[22,col2])

Longitudinal_Displacement_Endo_seg002_point3 = as.numeric(Displacement_data[22,col3])

Longitudinal_Displacement_Endo_seg002_point4 = as.numeric(Displacement_data[22,col4])

Longitudinal_Displacement_Endo_seg002_point5 = as.numeric(Displacement_data[22,col5])

Longitudinal_Displacement_Endo_seg002_a1 = (Longitudinal_Displacement_Endo_seg002_point2 - Longitudinal_Displacement_Endo_seg002_point1)/Time_duration1

Longitudinal_Displacement_Endo_seg002_a2 = (Longitudinal_Displacement_Endo_seg002_point3 - Longitudinal_Displacement_Endo_seg002_point2)/Time_duration2

Longitudinal_Displacement_Endo_seg002_a3 = (Longitudinal_Displacement_Endo_seg002_point4 - Longitudinal_Displacement_Endo_seg002_point3)/Time_duration3

Longitudinal_Displacement_Endo_seg002_a4 = (Longitudinal_Displacement_Endo_seg002_point5 - Longitudinal_Displacement_Endo_seg002_point4)/Time_duration4

Longitudinal_Displacement_Endo_seg002_a_min = min(c(Longitudinal_Displacement_Endo_seg002_a1,Longitudinal_Displacement_Endo_seg002_a2,Longitudinal_Displacement_Endo_seg002_a3,Longitudinal_Displacement_Endo_seg002_a4))

Longitudinal_Displacement_Endo_seg002_a_max = max(c(Longitudinal_Displacement_Endo_seg002_a1,Longitudinal_Displacement_Endo_seg002_a2,Longitudinal_Displacement_Endo_seg002_a3,Longitudinal_Displacement_Endo_seg002_a4))

Longitudinal_Displacement_Endo_seg003_point1 = as.numeric(Displacement_data[23,col1])

Longitudinal_Displacement_Endo_seg003_point2 = as.numeric(Displacement_data[23,col2])

Longitudinal_Displacement_Endo_seg003_point3 = as.numeric(Displacement_data[23,col3])

Longitudinal_Displacement_Endo_seg003_point4 = as.numeric(Displacement_data[23,col4])

Longitudinal_Displacement_Endo_seg003_point5 = as.numeric(Displacement_data[23,col5])

Longitudinal_Displacement_Endo_seg003_a1 = (Longitudinal_Displacement_Endo_seg003_point2 - Longitudinal_Displacement_Endo_seg003_point1)/Time_duration1

Longitudinal_Displacement_Endo_seg003_a2 = (Longitudinal_Displacement_Endo_seg003_point3 - Longitudinal_Displacement_Endo_seg003_point2)/Time_duration2

Longitudinal_Displacement_Endo_seg003_a3 = (Longitudinal_Displacement_Endo_seg003_point4 - Longitudinal_Displacement_Endo_seg003_point3)/Time_duration3

Longitudinal_Displacement_Endo_seg003_a4 = (Longitudinal_Displacement_Endo_seg003_point5 - Longitudinal_Displacement_Endo_seg003_point4)/Time_duration4

Longitudinal_Displacement_Endo_seg003_a_min = min(c(Longitudinal_Displacement_Endo_seg003_a1,Longitudinal_Displacement_Endo_seg003_a2,Longitudinal_Displacement_Endo_seg003_a3,Longitudinal_Displacement_Endo_seg003_a4))

Longitudinal_Displacement_Endo_seg003_a_max = max(c(Longitudinal_Displacement_Endo_seg003_a1,Longitudinal_Displacement_Endo_seg003_a2,Longitudinal_Displacement_Endo_seg003_a3,Longitudinal_Displacement_Endo_seg003_a4))

Longitudinal_Displacement_Endo_seg004_point1 = as.numeric(Displacement_data[24,col1])

Longitudinal_Displacement_Endo_seg004_point2 = as.numeric(Displacement_data[24,col2])

Longitudinal_Displacement_Endo_seg004_point3 = as.numeric(Displacement_data[24,col3])

Longitudinal_Displacement_Endo_seg004_point4 = as.numeric(Displacement_data[24,col4])

Longitudinal_Displacement_Endo_seg004_point5 = as.numeric(Displacement_data[24,col5])

Longitudinal_Displacement_Endo_seg004_a1 = (Longitudinal_Displacement_Endo_seg004_point2 - Longitudinal_Displacement_Endo_seg004_point1)/Time_duration1

Longitudinal_Displacement_Endo_seg004_a2 = (Longitudinal_Displacement_Endo_seg004_point3 - Longitudinal_Displacement_Endo_seg004_point2)/Time_duration2

Longitudinal_Displacement_Endo_seg004_a3 = (Longitudinal_Displacement_Endo_seg004_point4 - Longitudinal_Displacement_Endo_seg004_point3)/Time_duration3

Longitudinal_Displacement_Endo_seg004_a4 = (Longitudinal_Displacement_Endo_seg004_point5 - Longitudinal_Displacement_Endo_seg004_point4)/Time_duration4

Longitudinal_Displacement_Endo_seg004_a_min = min(c(Longitudinal_Displacement_Endo_seg004_a1,Longitudinal_Displacement_Endo_seg004_a2,Longitudinal_Displacement_Endo_seg004_a3,Longitudinal_Displacement_Endo_seg004_a4))

Longitudinal_Displacement_Endo_seg004_a_max = max(c(Longitudinal_Displacement_Endo_seg004_a1,Longitudinal_Displacement_Endo_seg004_a2,Longitudinal_Displacement_Endo_seg004_a3,Longitudinal_Displacement_Endo_seg004_a4))

Longitudinal_Displacement_Endo_seg005_point1 = as.numeric(Displacement_data[25,col1])

Longitudinal_Displacement_Endo_seg005_point2 = as.numeric(Displacement_data[25,col2])

Longitudinal_Displacement_Endo_seg005_point3 = as.numeric(Displacement_data[25,col3])

Longitudinal_Displacement_Endo_seg005_point4 = as.numeric(Displacement_data[25,col4])

Longitudinal_Displacement_Endo_seg005_point5 = as.numeric(Displacement_data[25,col5])

Longitudinal_Displacement_Endo_seg005_a1 = (Longitudinal_Displacement_Endo_seg005_point2 - Longitudinal_Displacement_Endo_seg005_point1)/Time_duration1

Longitudinal_Displacement_Endo_seg005_a2 = (Longitudinal_Displacement_Endo_seg005_point3 - Longitudinal_Displacement_Endo_seg005_point2)/Time_duration2

Longitudinal_Displacement_Endo_seg005_a3 = (Longitudinal_Displacement_Endo_seg005_point4 - Longitudinal_Displacement_Endo_seg005_point3)/Time_duration3

Longitudinal_Displacement_Endo_seg005_a4 = (Longitudinal_Displacement_Endo_seg005_point5 - Longitudinal_Displacement_Endo_seg005_point4)/Time_duration4

Longitudinal_Displacement_Endo_seg005_a_min = min(c(Longitudinal_Displacement_Endo_seg005_a1,Longitudinal_Displacement_Endo_seg005_a2,Longitudinal_Displacement_Endo_seg005_a3,Longitudinal_Displacement_Endo_seg005_a4))

Longitudinal_Displacement_Endo_seg005_a_max = max(c(Longitudinal_Displacement_Endo_seg005_a1,Longitudinal_Displacement_Endo_seg005_a2,Longitudinal_Displacement_Endo_seg005_a3,Longitudinal_Displacement_Endo_seg005_a4))

Longitudinal_Displacement_Endo_seg006_point1 = as.numeric(Displacement_data[26,col1])

Longitudinal_Displacement_Endo_seg006_point2 = as.numeric(Displacement_data[26,col2])

Longitudinal_Displacement_Endo_seg006_point3 = as.numeric(Displacement_data[26,col3])

Longitudinal_Displacement_Endo_seg006_point4 = as.numeric(Displacement_data[26,col4])

Longitudinal_Displacement_Endo_seg006_point5 = as.numeric(Displacement_data[26,col5])

Longitudinal_Displacement_Endo_seg006_a1 = (Longitudinal_Displacement_Endo_seg006_point2 - Longitudinal_Displacement_Endo_seg006_point1)/Time_duration1

Longitudinal_Displacement_Endo_seg006_a2 = (Longitudinal_Displacement_Endo_seg006_point3 - Longitudinal_Displacement_Endo_seg006_point2)/Time_duration2

Longitudinal_Displacement_Endo_seg006_a3 = (Longitudinal_Displacement_Endo_seg006_point4 - Longitudinal_Displacement_Endo_seg006_point3)/Time_duration3

Longitudinal_Displacement_Endo_seg006_a4 = (Longitudinal_Displacement_Endo_seg006_point5 - Longitudinal_Displacement_Endo_seg006_point4)/Time_duration4

Longitudinal_Displacement_Endo_seg006_a_min = min(c(Longitudinal_Displacement_Endo_seg006_a1,Longitudinal_Displacement_Endo_seg006_a2,Longitudinal_Displacement_Endo_seg006_a3,Longitudinal_Displacement_Endo_seg006_a4))

Longitudinal_Displacement_Endo_seg006_a_max = max(c(Longitudinal_Displacement_Endo_seg006_a1,Longitudinal_Displacement_Endo_seg006_a2,Longitudinal_Displacement_Endo_seg006_a3,Longitudinal_Displacement_Endo_seg006_a4))

##Average_values_per_frame_Longitudinal_Displacement_Endo

Average_values_per_frame_Longitudinal_Displacement_Endo_point1 = as.numeric(Displacement_data[30,col1])

Average_values_per_frame_Longitudinal_Displacement_Endo_point2 = as.numeric(Displacement_data[30,col2])

Average_values_per_frame_Longitudinal_Displacement_Endo_point3 = as.numeric(Displacement_data[30,col3])

Average_values_per_frame_Longitudinal_Displacement_Endo_point4 = as.numeric(Displacement_data[30,col4])

Average_values_per_frame_Longitudinal_Displacement_Endo_point5 = as.numeric(Displacement_data[30,col5])

Average_values_per_frame_Longitudinal_Displacement_Endo_a1 = (Average_values_per_frame_Longitudinal_Displacement_Endo_point2 - Average_values_per_frame_Longitudinal_Displacement_Endo_point1)/Time_duration1

Average_values_per_frame_Longitudinal_Displacement_Endo_a2 = (Average_values_per_frame_Longitudinal_Displacement_Endo_point3 - Average_values_per_frame_Longitudinal_Displacement_Endo_point2)/Time_duration2

Average_values_per_frame_Longitudinal_Displacement_Endo_a3 = (Average_values_per_frame_Longitudinal_Displacement_Endo_point4 - Average_values_per_frame_Longitudinal_Displacement_Endo_point3)/Time_duration3

Average_values_per_frame_Longitudinal_Displacement_Endo_a4 = (Average_values_per_frame_Longitudinal_Displacement_Endo_point5 - Average_values_per_frame_Longitudinal_Displacement_Endo_point4)/Time_duration4

Average_values_per_frame_Longitudinal_Displacement_Endo_a_min = min(c(Average_values_per_frame_Longitudinal_Displacement_Endo_a1,Average_values_per_frame_Longitudinal_Displacement_Endo_a2,Average_values_per_frame_Longitudinal_Displacement_Endo_a3,Average_values_per_frame_Longitudinal_Displacement_Endo_a4))

Average_values_per_frame_Longitudinal_Displacement_Endo_a_max = max(c(Average_values_per_frame_Longitudinal_Displacement_Endo_a1,Average_values_per_frame_Longitudinal_Displacement_Endo_a2,Average_values_per_frame_Longitudinal_Displacement_Endo_a3,Average_values_per_frame_Longitudinal_Displacement_Endo_a4))

##Radial_Displacement_Epi

Radial_Displacement_Epi_seg001_point1 = as.numeric(Displacement_data[34,col1])

Radial_Displacement_Epi_seg001_point2 = as.numeric(Displacement_data[34,col2])

Radial_Displacement_Epi_seg001_point3 = as.numeric(Displacement_data[34,col3])

Radial_Displacement_Epi_seg001_point4 = as.numeric(Displacement_data[34,col4])

Radial_Displacement_Epi_seg001_point5 = as.numeric(Displacement_data[34,col5])

Radial_Displacement_Epi_seg001_a1 = (Radial_Displacement_Epi_seg001_point2 - Radial_Displacement_Epi_seg001_point1)/Time_duration1

Radial_Displacement_Epi_seg001_a2 = (Radial_Displacement_Epi_seg001_point3 - Radial_Displacement_Epi_seg001_point2)/Time_duration2

Radial_Displacement_Epi_seg001_a3 = (Radial_Displacement_Epi_seg001_point4 - Radial_Displacement_Epi_seg001_point3)/Time_duration3

Radial_Displacement_Epi_seg001_a4 = (Radial_Displacement_Epi_seg001_point5 - Radial_Displacement_Epi_seg001_point4)/Time_duration4

Radial_Displacement_Epi_seg001_a_min = min(c(Radial_Displacement_Epi_seg001_a1,Radial_Displacement_Epi_seg001_a2,Radial_Displacement_Epi_seg001_a3,Radial_Displacement_Epi_seg001_a4))

Radial_Displacement_Epi_seg001_a_max = max(c(Radial_Displacement_Epi_seg001_a1,Radial_Displacement_Epi_seg001_a2,Radial_Displacement_Epi_seg001_a3,Radial_Displacement_Epi_seg001_a4))

Radial_Displacement_Epi_seg002_point1 = as.numeric(Displacement_data[35,col1])

Radial_Displacement_Epi_seg002_point2 = as.numeric(Displacement_data[35,col2])

Radial_Displacement_Epi_seg002_point3 = as.numeric(Displacement_data[35,col3])

Radial_Displacement_Epi_seg002_point4 = as.numeric(Displacement_data[35,col4])

Radial_Displacement_Epi_seg002_point5 = as.numeric(Displacement_data[35,col5])

Radial_Displacement_Epi_seg002_a1 = (Radial_Displacement_Epi_seg002_point2 - Radial_Displacement_Epi_seg002_point1)/Time_duration1

Radial_Displacement_Epi_seg002_a2 = (Radial_Displacement_Epi_seg002_point3 - Radial_Displacement_Epi_seg002_point2)/Time_duration2

Radial_Displacement_Epi_seg002_a3 = (Radial_Displacement_Epi_seg002_point4 - Radial_Displacement_Epi_seg002_point3)/Time_duration3

Radial_Displacement_Epi_seg002_a4 = (Radial_Displacement_Epi_seg002_point5 - Radial_Displacement_Epi_seg002_point4)/Time_duration4

Radial_Displacement_Epi_seg002_a_min = min(c(Radial_Displacement_Epi_seg002_a1,Radial_Displacement_Epi_seg002_a2,Radial_Displacement_Epi_seg002_a3,Radial_Displacement_Epi_seg002_a4))

Radial_Displacement_Epi_seg002_a_max = max(c(Radial_Displacement_Epi_seg002_a1,Radial_Displacement_Epi_seg002_a2,Radial_Displacement_Epi_seg002_a3,Radial_Displacement_Epi_seg002_a4))

Radial_Displacement_Epi_seg003_point1 = as.numeric(Displacement_data[36,col1])

Radial_Displacement_Epi_seg003_point2 = as.numeric(Displacement_data[36,col2])

Radial_Displacement_Epi_seg003_point3 = as.numeric(Displacement_data[36,col3])

Radial_Displacement_Epi_seg003_point4 = as.numeric(Displacement_data[36,col4])

Radial_Displacement_Epi_seg003_point5 = as.numeric(Displacement_data[36,col5])

Radial_Displacement_Epi_seg003_a1 = (Radial_Displacement_Epi_seg003_point2 - Radial_Displacement_Epi_seg003_point1)/Time_duration1

Radial_Displacement_Epi_seg003_a2 = (Radial_Displacement_Epi_seg003_point3 - Radial_Displacement_Epi_seg003_point2)/Time_duration2

Radial_Displacement_Epi_seg003_a3 = (Radial_Displacement_Epi_seg003_point4 - Radial_Displacement_Epi_seg003_point3)/Time_duration3

Radial_Displacement_Epi_seg003_a4 = (Radial_Displacement_Epi_seg003_point5 - Radial_Displacement_Epi_seg003_point4)/Time_duration4

Radial_Displacement_Epi_seg003_a_min = min(c(Radial_Displacement_Epi_seg003_a1,Radial_Displacement_Epi_seg003_a2,Radial_Displacement_Epi_seg003_a3,Radial_Displacement_Epi_seg003_a4))

Radial_Displacement_Epi_seg003_a_max = max(c(Radial_Displacement_Epi_seg003_a1,Radial_Displacement_Epi_seg003_a2,Radial_Displacement_Epi_seg003_a3,Radial_Displacement_Epi_seg003_a4))

Radial_Displacement_Epi_seg004_point1 = as.numeric(Displacement_data[37,col1])

Radial_Displacement_Epi_seg004_point2 = as.numeric(Displacement_data[37,col2])

Radial_Displacement_Epi_seg004_point3 = as.numeric(Displacement_data[37,col3])

Radial_Displacement_Epi_seg004_point4 = as.numeric(Displacement_data[37,col4])

Radial_Displacement_Epi_seg004_point5 = as.numeric(Displacement_data[37,col5])

Radial_Displacement_Epi_seg004_a1 = (Radial_Displacement_Epi_seg004_point2 - Radial_Displacement_Epi_seg004_point1)/Time_duration1

Radial_Displacement_Epi_seg004_a2 = (Radial_Displacement_Epi_seg004_point3 - Radial_Displacement_Epi_seg004_point2)/Time_duration2

Radial_Displacement_Epi_seg004_a3 = (Radial_Displacement_Epi_seg004_point4 - Radial_Displacement_Epi_seg004_point3)/Time_duration3

Radial_Displacement_Epi_seg004_a4 = (Radial_Displacement_Epi_seg004_point5 - Radial_Displacement_Epi_seg004_point4)/Time_duration4

Radial_Displacement_Epi_seg004_a_min = min(c(Radial_Displacement_Epi_seg004_a1,Radial_Displacement_Epi_seg004_a2,Radial_Displacement_Epi_seg004_a3,Radial_Displacement_Epi_seg004_a4))

Radial_Displacement_Epi_seg004_a_max = max(c(Radial_Displacement_Epi_seg004_a1,Radial_Displacement_Epi_seg004_a2,Radial_Displacement_Epi_seg004_a3,Radial_Displacement_Epi_seg004_a4))

Radial_Displacement_Epi_seg005_point1 = as.numeric(Displacement_data[38,col1])

Radial_Displacement_Epi_seg005_point2 = as.numeric(Displacement_data[38,col2])

Radial_Displacement_Epi_seg005_point3 = as.numeric(Displacement_data[38,col3])

Radial_Displacement_Epi_seg005_point4 = as.numeric(Displacement_data[38,col4])

Radial_Displacement_Epi_seg005_point5 = as.numeric(Displacement_data[38,col5])

Radial_Displacement_Epi_seg005_a1 = (Radial_Displacement_Epi_seg005_point2 - Radial_Displacement_Epi_seg005_point1)/Time_duration1

Radial_Displacement_Epi_seg005_a2 = (Radial_Displacement_Epi_seg005_point3 - Radial_Displacement_Epi_seg005_point2)/Time_duration2

Radial_Displacement_Epi_seg005_a3 = (Radial_Displacement_Epi_seg005_point4 - Radial_Displacement_Epi_seg005_point3)/Time_duration3

Radial_Displacement_Epi_seg005_a4 = (Radial_Displacement_Epi_seg005_point5 - Radial_Displacement_Epi_seg005_point4)/Time_duration4

Radial_Displacement_Epi_seg005_a_min = min(c(Radial_Displacement_Epi_seg005_a1,Radial_Displacement_Epi_seg005_a2,Radial_Displacement_Epi_seg005_a3,Radial_Displacement_Epi_seg005_a4))

Radial_Displacement_Epi_seg005_a_max = max(c(Radial_Displacement_Epi_seg005_a1,Radial_Displacement_Epi_seg005_a2,Radial_Displacement_Epi_seg005_a3,Radial_Displacement_Epi_seg005_a4))

Radial_Displacement_Epi_seg006_point1 = as.numeric(Displacement_data[39,col1])

Radial_Displacement_Epi_seg006_point2 = as.numeric(Displacement_data[39,col2])

Radial_Displacement_Epi_seg006_point3 = as.numeric(Displacement_data[39,col3])

Radial_Displacement_Epi_seg006_point4 = as.numeric(Displacement_data[39,col4])

Radial_Displacement_Epi_seg006_point5 = as.numeric(Displacement_data[39,col5])

Radial_Displacement_Epi_seg006_a1 = (Radial_Displacement_Epi_seg006_point2 - Radial_Displacement_Epi_seg006_point1)/Time_duration1

Radial_Displacement_Epi_seg006_a2 = (Radial_Displacement_Epi_seg006_point3 - Radial_Displacement_Epi_seg006_point2)/Time_duration2

Radial_Displacement_Epi_seg006_a3 = (Radial_Displacement_Epi_seg006_point4 - Radial_Displacement_Epi_seg006_point3)/Time_duration3

Radial_Displacement_Epi_seg006_a4 = (Radial_Displacement_Epi_seg006_point5 - Radial_Displacement_Epi_seg006_point4)/Time_duration4

Radial_Displacement_Epi_seg006_a_min = min(c(Radial_Displacement_Epi_seg006_a1,Radial_Displacement_Epi_seg006_a2,Radial_Displacement_Epi_seg006_a3,Radial_Displacement_Epi_seg006_a4))

Radial_Displacement_Epi_seg006_a_max = max(c(Radial_Displacement_Epi_seg006_a1,Radial_Displacement_Epi_seg006_a2,Radial_Displacement_Epi_seg006_a3,Radial_Displacement_Epi_seg006_a4))

##Average_values_per_frame_Radial_Displacement_Epi

Average_values_per_frame_Radial_Displacement_Epi_point1 = as.numeric(Displacement_data[43,col1])

Average_values_per_frame_Radial_Displacement_Epi_point2 = as.numeric(Displacement_data[43,col2])

Average_values_per_frame_Radial_Displacement_Epi_point3 = as.numeric(Displacement_data[43,col3])

Average_values_per_frame_Radial_Displacement_Epi_point4 = as.numeric(Displacement_data[43,col4])

Average_values_per_frame_Radial_Displacement_Epi_point5 = as.numeric(Displacement_data[43,col5])

Average_values_per_frame_Radial_Displacement_Epi_a1 = (Average_values_per_frame_Radial_Displacement_Epi_point2 - Average_values_per_frame_Radial_Displacement_Epi_point1)/Time_duration1

Average_values_per_frame_Radial_Displacement_Epi_a2 = (Average_values_per_frame_Radial_Displacement_Epi_point3 - Average_values_per_frame_Radial_Displacement_Epi_point2)/Time_duration2

Average_values_per_frame_Radial_Displacement_Epi_a3 = (Average_values_per_frame_Radial_Displacement_Epi_point4 - Average_values_per_frame_Radial_Displacement_Epi_point3)/Time_duration3

Average_values_per_frame_Radial_Displacement_Epi_a4 = (Average_values_per_frame_Radial_Displacement_Epi_point5 - Average_values_per_frame_Radial_Displacement_Epi_point4)/Time_duration4

Average_values_per_frame_Radial_Displacement_Epi_a_min = min(c(Average_values_per_frame_Radial_Displacement_Epi_a1,Average_values_per_frame_Radial_Displacement_Epi_a2,Average_values_per_frame_Radial_Displacement_Epi_a3,Average_values_per_frame_Radial_Displacement_Epi_a4))

Average_values_per_frame_Radial_Displacement_Epi_a_max = max(c(Average_values_per_frame_Radial_Displacement_Epi_a1,Average_values_per_frame_Radial_Displacement_Epi_a2,Average_values_per_frame_Radial_Displacement_Epi_a3,Average_values_per_frame_Radial_Displacement_Epi_a4))

##Longitudinal_Displacement_Epi

Longitudinal_Displacement_Epi_seg001_point1 = as.numeric(Displacement_data[47,col1])

Longitudinal_Displacement_Epi_seg001_point2 = as.numeric(Displacement_data[47,col2])

Longitudinal_Displacement_Epi_seg001_point3 = as.numeric(Displacement_data[47,col3])

Longitudinal_Displacement_Epi_seg001_point4 = as.numeric(Displacement_data[47,col4])

Longitudinal_Displacement_Epi_seg001_point5 = as.numeric(Displacement_data[47,col5])

Longitudinal_Displacement_Epi_seg001_a1 = (Longitudinal_Displacement_Epi_seg001_point2 - Longitudinal_Displacement_Epi_seg001_point1)/Time_duration1

Longitudinal_Displacement_Epi_seg001_a2 = (Longitudinal_Displacement_Epi_seg001_point3 - Longitudinal_Displacement_Epi_seg001_point2)/Time_duration2

Longitudinal_Displacement_Epi_seg001_a3 = (Longitudinal_Displacement_Epi_seg001_point4 - Longitudinal_Displacement_Epi_seg001_point3)/Time_duration3

Longitudinal_Displacement_Epi_seg001_a4 = (Longitudinal_Displacement_Epi_seg001_point5 - Longitudinal_Displacement_Epi_seg001_point4)/Time_duration4

Longitudinal_Displacement_Epi_seg001_a_min = min(c(Longitudinal_Displacement_Epi_seg001_a1,Longitudinal_Displacement_Epi_seg001_a2,Longitudinal_Displacement_Epi_seg001_a3,Longitudinal_Displacement_Epi_seg001_a4))

Longitudinal_Displacement_Epi_seg001_a_max = max(c(Longitudinal_Displacement_Epi_seg001_a1,Longitudinal_Displacement_Epi_seg001_a2,Longitudinal_Displacement_Epi_seg001_a3,Longitudinal_Displacement_Epi_seg001_a4))

Longitudinal_Displacement_Epi_seg002_point1 = as.numeric(Displacement_data[48,col1])

Longitudinal_Displacement_Epi_seg002_point2 = as.numeric(Displacement_data[48,col2])

Longitudinal_Displacement_Epi_seg002_point3 = as.numeric(Displacement_data[48,col3])

Longitudinal_Displacement_Epi_seg002_point4 = as.numeric(Displacement_data[48,col4])

Longitudinal_Displacement_Epi_seg002_point5 = as.numeric(Displacement_data[48,col5])

Longitudinal_Displacement_Epi_seg002_a1 = (Longitudinal_Displacement_Epi_seg002_point2 - Longitudinal_Displacement_Epi_seg002_point1)/Time_duration1

Longitudinal_Displacement_Epi_seg002_a2 = (Longitudinal_Displacement_Epi_seg002_point3 - Longitudinal_Displacement_Epi_seg002_point2)/Time_duration2

Longitudinal_Displacement_Epi_seg002_a3 = (Longitudinal_Displacement_Epi_seg002_point4 - Longitudinal_Displacement_Epi_seg002_point3)/Time_duration3

Longitudinal_Displacement_Epi_seg002_a4 = (Longitudinal_Displacement_Epi_seg002_point5 - Longitudinal_Displacement_Epi_seg002_point4)/Time_duration4

Longitudinal_Displacement_Epi_seg002_a_min = min(c(Longitudinal_Displacement_Epi_seg002_a1,Longitudinal_Displacement_Epi_seg002_a2,Longitudinal_Displacement_Epi_seg002_a3,Longitudinal_Displacement_Epi_seg002_a4))

Longitudinal_Displacement_Epi_seg002_a_max = max(c(Longitudinal_Displacement_Epi_seg002_a1,Longitudinal_Displacement_Epi_seg002_a2,Longitudinal_Displacement_Epi_seg002_a3,Longitudinal_Displacement_Epi_seg002_a4))

Longitudinal_Displacement_Epi_seg003_point1 = as.numeric(Displacement_data[49,col1])

Longitudinal_Displacement_Epi_seg003_point2 = as.numeric(Displacement_data[49,col2])

Longitudinal_Displacement_Epi_seg003_point3 = as.numeric(Displacement_data[49,col3])

Longitudinal_Displacement_Epi_seg003_point4 = as.numeric(Displacement_data[49,col4])

Longitudinal_Displacement_Epi_seg003_point5 = as.numeric(Displacement_data[49,col5])

Longitudinal_Displacement_Epi_seg003_a1 = (Longitudinal_Displacement_Epi_seg003_point2 - Longitudinal_Displacement_Epi_seg003_point1)/Time_duration1

Longitudinal_Displacement_Epi_seg003_a2 = (Longitudinal_Displacement_Epi_seg003_point3 - Longitudinal_Displacement_Epi_seg003_point2)/Time_duration2

Longitudinal_Displacement_Epi_seg003_a3 = (Longitudinal_Displacement_Epi_seg003_point4 - Longitudinal_Displacement_Epi_seg003_point3)/Time_duration3

Longitudinal_Displacement_Epi_seg003_a4 = (Longitudinal_Displacement_Epi_seg003_point5 - Longitudinal_Displacement_Epi_seg003_point4)/Time_duration4

Longitudinal_Displacement_Epi_seg003_a_min = min(c(Longitudinal_Displacement_Epi_seg003_a1,Longitudinal_Displacement_Epi_seg003_a2,Longitudinal_Displacement_Epi_seg003_a3,Longitudinal_Displacement_Epi_seg003_a4))

Longitudinal_Displacement_Epi_seg003_a_max = max(c(Longitudinal_Displacement_Epi_seg003_a1,Longitudinal_Displacement_Epi_seg003_a2,Longitudinal_Displacement_Epi_seg003_a3,Longitudinal_Displacement_Epi_seg003_a4))

Longitudinal_Displacement_Epi_seg004_point1 = as.numeric(Displacement_data[50,col1])

Longitudinal_Displacement_Epi_seg004_point2 = as.numeric(Displacement_data[50,col2])

Longitudinal_Displacement_Epi_seg004_point3 = as.numeric(Displacement_data[50,col3])

Longitudinal_Displacement_Epi_seg004_point4 = as.numeric(Displacement_data[50,col4])

Longitudinal_Displacement_Epi_seg004_point5 = as.numeric(Displacement_data[50,col5])

Longitudinal_Displacement_Epi_seg004_a1 = (Longitudinal_Displacement_Epi_seg004_point2 - Longitudinal_Displacement_Epi_seg004_point1)/Time_duration1

Longitudinal_Displacement_Epi_seg004_a2 = (Longitudinal_Displacement_Epi_seg004_point3 - Longitudinal_Displacement_Epi_seg004_point2)/Time_duration2

Longitudinal_Displacement_Epi_seg004_a3 = (Longitudinal_Displacement_Epi_seg004_point4 - Longitudinal_Displacement_Epi_seg004_point3)/Time_duration3

Longitudinal_Displacement_Epi_seg004_a4 = (Longitudinal_Displacement_Epi_seg004_point5 - Longitudinal_Displacement_Epi_seg004_point4)/Time_duration4

Longitudinal_Displacement_Epi_seg004_a_min = min(c(Longitudinal_Displacement_Epi_seg004_a1,Longitudinal_Displacement_Epi_seg004_a2,Longitudinal_Displacement_Epi_seg004_a3,Longitudinal_Displacement_Epi_seg004_a4))

Longitudinal_Displacement_Epi_seg004_a_max = max(c(Longitudinal_Displacement_Epi_seg004_a1,Longitudinal_Displacement_Epi_seg004_a2,Longitudinal_Displacement_Epi_seg004_a3,Longitudinal_Displacement_Epi_seg004_a4))

Longitudinal_Displacement_Epi_seg005_point1 = as.numeric(Displacement_data[51,col1])

Longitudinal_Displacement_Epi_seg005_point2 = as.numeric(Displacement_data[51,col2])

Longitudinal_Displacement_Epi_seg005_point3 = as.numeric(Displacement_data[51,col3])

Longitudinal_Displacement_Epi_seg005_point4 = as.numeric(Displacement_data[51,col4])

Longitudinal_Displacement_Epi_seg005_point5 = as.numeric(Displacement_data[51,col5])

Longitudinal_Displacement_Epi_seg005_a1 = (Longitudinal_Displacement_Epi_seg005_point2 - Longitudinal_Displacement_Epi_seg005_point1)/Time_duration1

Longitudinal_Displacement_Epi_seg005_a2 = (Longitudinal_Displacement_Epi_seg005_point3 - Longitudinal_Displacement_Epi_seg005_point2)/Time_duration2

Longitudinal_Displacement_Epi_seg005_a3 = (Longitudinal_Displacement_Epi_seg005_point4 - Longitudinal_Displacement_Epi_seg005_point3)/Time_duration3

Longitudinal_Displacement_Epi_seg005_a4 = (Longitudinal_Displacement_Epi_seg005_point5 - Longitudinal_Displacement_Epi_seg005_point4)/Time_duration4

Longitudinal_Displacement_Epi_seg005_a_min = min(c(Longitudinal_Displacement_Epi_seg005_a1,Longitudinal_Displacement_Epi_seg005_a2,Longitudinal_Displacement_Epi_seg005_a3,Longitudinal_Displacement_Epi_seg005_a4))

Longitudinal_Displacement_Epi_seg005_a_max = max(c(Longitudinal_Displacement_Epi_seg005_a1,Longitudinal_Displacement_Epi_seg005_a2,Longitudinal_Displacement_Epi_seg005_a3,Longitudinal_Displacement_Epi_seg005_a4))

Longitudinal_Displacement_Epi_seg006_point1 = as.numeric(Displacement_data[52,col1])

Longitudinal_Displacement_Epi_seg006_point2 = as.numeric(Displacement_data[52,col2])

Longitudinal_Displacement_Epi_seg006_point3 = as.numeric(Displacement_data[52,col3])

Longitudinal_Displacement_Epi_seg006_point4 = as.numeric(Displacement_data[52,col4])

Longitudinal_Displacement_Epi_seg006_point5 = as.numeric(Displacement_data[52,col5])

Longitudinal_Displacement_Epi_seg006_a1 = (Longitudinal_Displacement_Epi_seg006_point2 - Longitudinal_Displacement_Epi_seg006_point1)/Time_duration1

Longitudinal_Displacement_Epi_seg006_a2 = (Longitudinal_Displacement_Epi_seg006_point3 - Longitudinal_Displacement_Epi_seg006_point2)/Time_duration2

Longitudinal_Displacement_Epi_seg006_a3 = (Longitudinal_Displacement_Epi_seg006_point4 - Longitudinal_Displacement_Epi_seg006_point3)/Time_duration3

Longitudinal_Displacement_Epi_seg006_a4 = (Longitudinal_Displacement_Epi_seg006_point5 - Longitudinal_Displacement_Epi_seg006_point4)/Time_duration4

Longitudinal_Displacement_Epi_seg006_a_min = min(c(Longitudinal_Displacement_Epi_seg006_a1,Longitudinal_Displacement_Epi_seg006_a2,Longitudinal_Displacement_Epi_seg006_a3,Longitudinal_Displacement_Epi_seg006_a4))

Longitudinal_Displacement_Epi_seg006_a_max = max(c(Longitudinal_Displacement_Epi_seg006_a1,Longitudinal_Displacement_Epi_seg006_a2,Longitudinal_Displacement_Epi_seg006_a3,Longitudinal_Displacement_Epi_seg006_a4))

##Average_values_per_frame_Longitudinal_Displacement_Epi

Average_values_per_frame_Longitudinal_Displacement_Epi_point1 = as.numeric(Displacement_data[56,col1])

Average_values_per_frame_Longitudinal_Displacement_Epi_point2 = as.numeric(Displacement_data[56,col2])

Average_values_per_frame_Longitudinal_Displacement_Epi_point3 = as.numeric(Displacement_data[56,col3])

Average_values_per_frame_Longitudinal_Displacement_Epi_point4 = as.numeric(Displacement_data[56,col4])

Average_values_per_frame_Longitudinal_Displacement_Epi_point5 = as.numeric(Displacement_data[56,col5])

Average_values_per_frame_Longitudinal_Displacement_Epi_a1 = (Average_values_per_frame_Longitudinal_Displacement_Epi_point2 - Average_values_per_frame_Longitudinal_Displacement_Epi_point1)/Time_duration1

Average_values_per_frame_Longitudinal_Displacement_Epi_a2 = (Average_values_per_frame_Longitudinal_Displacement_Epi_point3 - Average_values_per_frame_Longitudinal_Displacement_Epi_point2)/Time_duration2

Average_values_per_frame_Longitudinal_Displacement_Epi_a3 = (Average_values_per_frame_Longitudinal_Displacement_Epi_point4 - Average_values_per_frame_Longitudinal_Displacement_Epi_point3)/Time_duration3

Average_values_per_frame_Longitudinal_Displacement_Epi_a4 = (Average_values_per_frame_Longitudinal_Displacement_Epi_point5 - Average_values_per_frame_Longitudinal_Displacement_Epi_point4)/Time_duration4

Average_values_per_frame_Longitudinal_Displacement_Epi_a_min = min(c(Average_values_per_frame_Longitudinal_Displacement_Epi_a1,Average_values_per_frame_Longitudinal_Displacement_Epi_a2,Average_values_per_frame_Longitudinal_Displacement_Epi_a3,Average_values_per_frame_Longitudinal_Displacement_Epi_a4))

Average_values_per_frame_Longitudinal_Displacement_Epi_a_max = max(c(Average_values_per_frame_Longitudinal_Displacement_Epi_a1,Average_values_per_frame_Longitudinal_Displacement_Epi_a2,Average_values_per_frame_Longitudinal_Displacement_Epi_a3,Average_values_per_frame_Longitudinal_Displacement_Epi_a4))

#Strain

Strain_data <- read.xlsx(i,sheetIndex = 4, header = F)

colnames(Strain_data) <- Strain_data[3,]

name1 = paste("fr0",time3,sep = "")

col5 = grep(name1,colnames(Strain_data))

col4 = col5-1

col3 = col5-2

col2 = col5-3

col1 = col5-4

Timepoint1 = as.numeric(Strain_data[4,col1])

Timepoint2 = as.numeric(Strain_data[4,col2])

Timepoint3 = as.numeric(Strain_data[4,col3])

Timepoint4 = as.numeric(Strain_data[4,col4])

Timepoint5 = as.numeric(Strain_data[4,col5])

Time_duration1 = Timepoint2 - Timepoint1

Time_duration2 = Timepoint3 - Timepoint2

Time_duration3 = Timepoint4 - Timepoint3

Time_duration4 = Timepoint5 - Timepoint4

##Radial_Strain_Endo

Radial_Strain_Endo_seg001_point1 = as.numeric(Strain_data[8,col1])

Radial_Strain_Endo_seg001_point2 = as.numeric(Strain_data[8,col2])

Radial_Strain_Endo_seg001_point3 = as.numeric(Strain_data[8,col3])

Radial_Strain_Endo_seg001_point4 = as.numeric(Strain_data[8,col4])

Radial_Strain_Endo_seg001_point5 = as.numeric(Strain_data[8,col5])

Radial_Strain_Endo_seg001_a1 = (Radial_Strain_Endo_seg001_point2 - Radial_Strain_Endo_seg001_point1)/Time_duration1

Radial_Strain_Endo_seg001_a2 = (Radial_Strain_Endo_seg001_point3 - Radial_Strain_Endo_seg001_point2)/Time_duration2

Radial_Strain_Endo_seg001_a3 = (Radial_Strain_Endo_seg001_point4 - Radial_Strain_Endo_seg001_point3)/Time_duration3

Radial_Strain_Endo_seg001_a4 = (Radial_Strain_Endo_seg001_point5 - Radial_Strain_Endo_seg001_point4)/Time_duration4

Radial_Strain_Endo_seg001_a_min = min(c(Radial_Strain_Endo_seg001_a1,Radial_Strain_Endo_seg001_a2,Radial_Strain_Endo_seg001_a3,Radial_Strain_Endo_seg001_a4))

Radial_Strain_Endo_seg001_a_max = max(c(Radial_Strain_Endo_seg001_a1,Radial_Strain_Endo_seg001_a2,Radial_Strain_Endo_seg001_a3,Radial_Strain_Endo_seg001_a4))

Radial_Strain_Endo_seg002_point1 = as.numeric(Strain_data[9,col1])

Radial_Strain_Endo_seg002_point2 = as.numeric(Strain_data[9,col2])

Radial_Strain_Endo_seg002_point3 = as.numeric(Strain_data[9,col3])

Radial_Strain_Endo_seg002_point4 = as.numeric(Strain_data[9,col4])

Radial_Strain_Endo_seg002_point5 = as.numeric(Strain_data[9,col5])

Radial_Strain_Endo_seg002_a1 = (Radial_Strain_Endo_seg002_point2 - Radial_Strain_Endo_seg002_point1)/Time_duration1

Radial_Strain_Endo_seg002_a2 = (Radial_Strain_Endo_seg002_point3 - Radial_Strain_Endo_seg002_point2)/Time_duration2

Radial_Strain_Endo_seg002_a3 = (Radial_Strain_Endo_seg002_point4 - Radial_Strain_Endo_seg002_point3)/Time_duration3

Radial_Strain_Endo_seg002_a4 = (Radial_Strain_Endo_seg002_point5 - Radial_Strain_Endo_seg002_point4)/Time_duration4

Radial_Strain_Endo_seg002_a_min = min(c(Radial_Strain_Endo_seg002_a1,Radial_Strain_Endo_seg002_a2,Radial_Strain_Endo_seg002_a3,Radial_Strain_Endo_seg002_a4))

Radial_Strain_Endo_seg002_a_max = max(c(Radial_Strain_Endo_seg002_a1,Radial_Strain_Endo_seg002_a2,Radial_Strain_Endo_seg002_a3,Radial_Strain_Endo_seg002_a4))

Radial_Strain_Endo_seg003_point1 = as.numeric(Strain_data[10,col1])

Radial_Strain_Endo_seg003_point2 = as.numeric(Strain_data[10,col2])

Radial_Strain_Endo_seg003_point3 = as.numeric(Strain_data[10,col3])

Radial_Strain_Endo_seg003_point4 = as.numeric(Strain_data[10,col4])

Radial_Strain_Endo_seg003_point5 = as.numeric(Strain_data[10,col5])

Radial_Strain_Endo_seg003_a1 = (Radial_Strain_Endo_seg003_point2 - Radial_Strain_Endo_seg003_point1)/Time_duration1

Radial_Strain_Endo_seg003_a2 = (Radial_Strain_Endo_seg003_point3 - Radial_Strain_Endo_seg003_point2)/Time_duration2

Radial_Strain_Endo_seg003_a3 = (Radial_Strain_Endo_seg003_point4 - Radial_Strain_Endo_seg003_point3)/Time_duration3

Radial_Strain_Endo_seg003_a4 = (Radial_Strain_Endo_seg003_point5 - Radial_Strain_Endo_seg003_point4)/Time_duration4

Radial_Strain_Endo_seg003_a_min = min(c(Radial_Strain_Endo_seg003_a1,Radial_Strain_Endo_seg003_a2,Radial_Strain_Endo_seg003_a3,Radial_Strain_Endo_seg003_a4))

Radial_Strain_Endo_seg003_a_max = max(c(Radial_Strain_Endo_seg003_a1,Radial_Strain_Endo_seg003_a2,Radial_Strain_Endo_seg003_a3,Radial_Strain_Endo_seg003_a4))

Radial_Strain_Endo_seg004_point1 = as.numeric(Strain_data[11,col1])

Radial_Strain_Endo_seg004_point2 = as.numeric(Strain_data[11,col2])

Radial_Strain_Endo_seg004_point3 = as.numeric(Strain_data[11,col3])

Radial_Strain_Endo_seg004_point4 = as.numeric(Strain_data[11,col4])

Radial_Strain_Endo_seg004_point5 = as.numeric(Strain_data[11,col5])

Radial_Strain_Endo_seg004_a1 = (Radial_Strain_Endo_seg004_point2 - Radial_Strain_Endo_seg004_point1)/Time_duration1

Radial_Strain_Endo_seg004_a2 = (Radial_Strain_Endo_seg004_point3 - Radial_Strain_Endo_seg004_point2)/Time_duration2

Radial_Strain_Endo_seg004_a3 = (Radial_Strain_Endo_seg004_point4 - Radial_Strain_Endo_seg004_point3)/Time_duration3

Radial_Strain_Endo_seg004_a4 = (Radial_Strain_Endo_seg004_point5 - Radial_Strain_Endo_seg004_point4)/Time_duration4

Radial_Strain_Endo_seg004_a_min = min(c(Radial_Strain_Endo_seg004_a1,Radial_Strain_Endo_seg004_a2,Radial_Strain_Endo_seg004_a3,Radial_Strain_Endo_seg004_a4))

Radial_Strain_Endo_seg004_a_max = max(c(Radial_Strain_Endo_seg004_a1,Radial_Strain_Endo_seg004_a2,Radial_Strain_Endo_seg004_a3,Radial_Strain_Endo_seg004_a4))

Radial_Strain_Endo_seg005_point1 = as.numeric(Strain_data[12,col1])

Radial_Strain_Endo_seg005_point2 = as.numeric(Strain_data[12,col2])

Radial_Strain_Endo_seg005_point3 = as.numeric(Strain_data[12,col3])

Radial_Strain_Endo_seg005_point4 = as.numeric(Strain_data[12,col4])

Radial_Strain_Endo_seg005_point5 = as.numeric(Strain_data[12,col5])

Radial_Strain_Endo_seg005_a1 = (Radial_Strain_Endo_seg005_point2 - Radial_Strain_Endo_seg005_point1)/Time_duration1

Radial_Strain_Endo_seg005_a2 = (Radial_Strain_Endo_seg005_point3 - Radial_Strain_Endo_seg005_point2)/Time_duration2

Radial_Strain_Endo_seg005_a3 = (Radial_Strain_Endo_seg005_point4 - Radial_Strain_Endo_seg005_point3)/Time_duration3

Radial_Strain_Endo_seg005_a4 = (Radial_Strain_Endo_seg005_point5 - Radial_Strain_Endo_seg005_point4)/Time_duration4

Radial_Strain_Endo_seg005_a_min = min(c(Radial_Strain_Endo_seg005_a1,Radial_Strain_Endo_seg005_a2,Radial_Strain_Endo_seg005_a3,Radial_Strain_Endo_seg005_a4))

Radial_Strain_Endo_seg005_a_max = max(c(Radial_Strain_Endo_seg005_a1,Radial_Strain_Endo_seg005_a2,Radial_Strain_Endo_seg005_a3,Radial_Strain_Endo_seg005_a4))

Radial_Strain_Endo_seg006_point1 = as.numeric(Strain_data[13,col1])

Radial_Strain_Endo_seg006_point2 = as.numeric(Strain_data[13,col2])

Radial_Strain_Endo_seg006_point3 = as.numeric(Strain_data[13,col3])

Radial_Strain_Endo_seg006_point4 = as.numeric(Strain_data[13,col4])

Radial_Strain_Endo_seg006_point5 = as.numeric(Strain_data[13,col5])

Radial_Strain_Endo_seg006_a1 = (Radial_Strain_Endo_seg006_point2 - Radial_Strain_Endo_seg006_point1)/Time_duration1

Radial_Strain_Endo_seg006_a2 = (Radial_Strain_Endo_seg006_point3 - Radial_Strain_Endo_seg006_point2)/Time_duration2

Radial_Strain_Endo_seg006_a3 = (Radial_Strain_Endo_seg006_point4 - Radial_Strain_Endo_seg006_point3)/Time_duration3

Radial_Strain_Endo_seg006_a4 = (Radial_Strain_Endo_seg006_point5 - Radial_Strain_Endo_seg006_point4)/Time_duration4

Radial_Strain_Endo_seg006_a_min = min(c(Radial_Strain_Endo_seg006_a1,Radial_Strain_Endo_seg006_a2,Radial_Strain_Endo_seg006_a3,Radial_Strain_Endo_seg006_a4))

Radial_Strain_Endo_seg006_a_max = max(c(Radial_Strain_Endo_seg006_a1,Radial_Strain_Endo_seg006_a2,Radial_Strain_Endo_seg006_a3,Radial_Strain_Endo_seg006_a4))

##Average_values_per_frame_Radial_Strain_Endo

Average_values_per_frame_Radial_Strain_Endo_point1 = as.numeric(Strain_data[17,col1])

Average_values_per_frame_Radial_Strain_Endo_point2 = as.numeric(Strain_data[17,col2])

Average_values_per_frame_Radial_Strain_Endo_point3 = as.numeric(Strain_data[17,col3])

Average_values_per_frame_Radial_Strain_Endo_point4 = as.numeric(Strain_data[17,col4])

Average_values_per_frame_Radial_Strain_Endo_point5 = as.numeric(Strain_data[17,col5])

Average_values_per_frame_Radial_Strain_Endo_a1 = (Average_values_per_frame_Radial_Strain_Endo_point2 - Average_values_per_frame_Radial_Strain_Endo_point1)/Time_duration1

Average_values_per_frame_Radial_Strain_Endo_a2 = (Average_values_per_frame_Radial_Strain_Endo_point3 - Average_values_per_frame_Radial_Strain_Endo_point2)/Time_duration2

Average_values_per_frame_Radial_Strain_Endo_a3 = (Average_values_per_frame_Radial_Strain_Endo_point4 - Average_values_per_frame_Radial_Strain_Endo_point3)/Time_duration3

Average_values_per_frame_Radial_Strain_Endo_a4 = (Average_values_per_frame_Radial_Strain_Endo_point5 - Average_values_per_frame_Radial_Strain_Endo_point4)/Time_duration4

Average_values_per_frame_Radial_Strain_Endo_a_min = min(c(Average_values_per_frame_Radial_Strain_Endo_a1,Average_values_per_frame_Radial_Strain_Endo_a2,Average_values_per_frame_Radial_Strain_Endo_a3,Average_values_per_frame_Radial_Strain_Endo_a4))

Average_values_per_frame_Radial_Strain_Endo_a_max = max(c(Average_values_per_frame_Radial_Strain_Endo_a1,Average_values_per_frame_Radial_Strain_Endo_a2,Average_values_per_frame_Radial_Strain_Endo_a3,Average_values_per_frame_Radial_Strain_Endo_a4))

##Longitudinal_Strain_Endo

Longitudinal_Strain_Endo_seg001_point1 = as.numeric(Strain_data[21,col1])

Longitudinal_Strain_Endo_seg001_point2 = as.numeric(Strain_data[21,col2])

Longitudinal_Strain_Endo_seg001_point3 = as.numeric(Strain_data[21,col3])

Longitudinal_Strain_Endo_seg001_point4 = as.numeric(Strain_data[21,col4])

Longitudinal_Strain_Endo_seg001_point5 = as.numeric(Strain_data[21,col5])

Longitudinal_Strain_Endo_seg001_a1 = (Longitudinal_Strain_Endo_seg001_point2 - Longitudinal_Strain_Endo_seg001_point1)/Time_duration1

Longitudinal_Strain_Endo_seg001_a2 = (Longitudinal_Strain_Endo_seg001_point3 - Longitudinal_Strain_Endo_seg001_point2)/Time_duration2

Longitudinal_Strain_Endo_seg001_a3 = (Longitudinal_Strain_Endo_seg001_point4 - Longitudinal_Strain_Endo_seg001_point3)/Time_duration3

Longitudinal_Strain_Endo_seg001_a4 = (Longitudinal_Strain_Endo_seg001_point5 - Longitudinal_Strain_Endo_seg001_point4)/Time_duration4

Longitudinal_Strain_Endo_seg001_a_min = min(c(Longitudinal_Strain_Endo_seg001_a1,Longitudinal_Strain_Endo_seg001_a2,Longitudinal_Strain_Endo_seg001_a3,Longitudinal_Strain_Endo_seg001_a4))

Longitudinal_Strain_Endo_seg001_a_max = max(c(Longitudinal_Strain_Endo_seg001_a1,Longitudinal_Strain_Endo_seg001_a2,Longitudinal_Strain_Endo_seg001_a3,Longitudinal_Strain_Endo_seg001_a4))

Longitudinal_Strain_Endo_seg002_point1 = as.numeric(Strain_data[22,col1])

Longitudinal_Strain_Endo_seg002_point2 = as.numeric(Strain_data[22,col2])

Longitudinal_Strain_Endo_seg002_point3 = as.numeric(Strain_data[22,col3])

Longitudinal_Strain_Endo_seg002_point4 = as.numeric(Strain_data[22,col4])

Longitudinal_Strain_Endo_seg002_point5 = as.numeric(Strain_data[22,col5])

Longitudinal_Strain_Endo_seg002_a1 = (Longitudinal_Strain_Endo_seg002_point2 - Longitudinal_Strain_Endo_seg002_point1)/Time_duration1

Longitudinal_Strain_Endo_seg002_a2 = (Longitudinal_Strain_Endo_seg002_point3 - Longitudinal_Strain_Endo_seg002_point2)/Time_duration2

Longitudinal_Strain_Endo_seg002_a3 = (Longitudinal_Strain_Endo_seg002_point4 - Longitudinal_Strain_Endo_seg002_point3)/Time_duration3

Longitudinal_Strain_Endo_seg002_a4 = (Longitudinal_Strain_Endo_seg002_point5 - Longitudinal_Strain_Endo_seg002_point4)/Time_duration4

Longitudinal_Strain_Endo_seg002_a_min = min(c(Longitudinal_Strain_Endo_seg002_a1,Longitudinal_Strain_Endo_seg002_a2,Longitudinal_Strain_Endo_seg002_a3,Longitudinal_Strain_Endo_seg002_a4))

Longitudinal_Strain_Endo_seg002_a_max = max(c(Longitudinal_Strain_Endo_seg002_a1,Longitudinal_Strain_Endo_seg002_a2,Longitudinal_Strain_Endo_seg002_a3,Longitudinal_Strain_Endo_seg002_a4))

Longitudinal_Strain_Endo_seg003_point1 = as.numeric(Strain_data[23,col1])

Longitudinal_Strain_Endo_seg003_point2 = as.numeric(Strain_data[23,col2])

Longitudinal_Strain_Endo_seg003_point3 = as.numeric(Strain_data[23,col3])

Longitudinal_Strain_Endo_seg003_point4 = as.numeric(Strain_data[23,col4])

Longitudinal_Strain_Endo_seg003_point5 = as.numeric(Strain_data[23,col5])

Longitudinal_Strain_Endo_seg003_a1 = (Longitudinal_Strain_Endo_seg003_point2 - Longitudinal_Strain_Endo_seg003_point1)/Time_duration1

Longitudinal_Strain_Endo_seg003_a2 = (Longitudinal_Strain_Endo_seg003_point3 - Longitudinal_Strain_Endo_seg003_point2)/Time_duration2

Longitudinal_Strain_Endo_seg003_a3 = (Longitudinal_Strain_Endo_seg003_point4 - Longitudinal_Strain_Endo_seg003_point3)/Time_duration3

Longitudinal_Strain_Endo_seg003_a4 = (Longitudinal_Strain_Endo_seg003_point5 - Longitudinal_Strain_Endo_seg003_point4)/Time_duration4

Longitudinal_Strain_Endo_seg003_a_min = min(c(Longitudinal_Strain_Endo_seg003_a1,Longitudinal_Strain_Endo_seg003_a2,Longitudinal_Strain_Endo_seg003_a3,Longitudinal_Strain_Endo_seg003_a4))

Longitudinal_Strain_Endo_seg003_a_max = max(c(Longitudinal_Strain_Endo_seg003_a1,Longitudinal_Strain_Endo_seg003_a2,Longitudinal_Strain_Endo_seg003_a3,Longitudinal_Strain_Endo_seg003_a4))

Longitudinal_Strain_Endo_seg004_point1 = as.numeric(Strain_data[24,col1])

Longitudinal_Strain_Endo_seg004_point2 = as.numeric(Strain_data[24,col2])

Longitudinal_Strain_Endo_seg004_point3 = as.numeric(Strain_data[24,col3])

Longitudinal_Strain_Endo_seg004_point4 = as.numeric(Strain_data[24,col4])

Longitudinal_Strain_Endo_seg004_point5 = as.numeric(Strain_data[24,col5])

Longitudinal_Strain_Endo_seg004_a1 = (Longitudinal_Strain_Endo_seg004_point2 - Longitudinal_Strain_Endo_seg004_point1)/Time_duration1

Longitudinal_Strain_Endo_seg004_a2 = (Longitudinal_Strain_Endo_seg004_point3 - Longitudinal_Strain_Endo_seg004_point2)/Time_duration2

Longitudinal_Strain_Endo_seg004_a3 = (Longitudinal_Strain_Endo_seg004_point4 - Longitudinal_Strain_Endo_seg004_point3)/Time_duration3

Longitudinal_Strain_Endo_seg004_a4 = (Longitudinal_Strain_Endo_seg004_point5 - Longitudinal_Strain_Endo_seg004_point4)/Time_duration4

Longitudinal_Strain_Endo_seg004_a_min = min(c(Longitudinal_Strain_Endo_seg004_a1,Longitudinal_Strain_Endo_seg004_a2,Longitudinal_Strain_Endo_seg004_a3,Longitudinal_Strain_Endo_seg004_a4))

Longitudinal_Strain_Endo_seg004_a_max = max(c(Longitudinal_Strain_Endo_seg004_a1,Longitudinal_Strain_Endo_seg004_a2,Longitudinal_Strain_Endo_seg004_a3,Longitudinal_Strain_Endo_seg004_a4))

Longitudinal_Strain_Endo_seg005_point1 = as.numeric(Strain_data[25,col1])

Longitudinal_Strain_Endo_seg005_point2 = as.numeric(Strain_data[25,col2])

Longitudinal_Strain_Endo_seg005_point3 = as.numeric(Strain_data[25,col3])

Longitudinal_Strain_Endo_seg005_point4 = as.numeric(Strain_data[25,col4])

Longitudinal_Strain_Endo_seg005_point5 = as.numeric(Strain_data[25,col5])

Longitudinal_Strain_Endo_seg005_a1 = (Longitudinal_Strain_Endo_seg005_point2 - Longitudinal_Strain_Endo_seg005_point1)/Time_duration1

Longitudinal_Strain_Endo_seg005_a2 = (Longitudinal_Strain_Endo_seg005_point3 - Longitudinal_Strain_Endo_seg005_point2)/Time_duration2

Longitudinal_Strain_Endo_seg005_a3 = (Longitudinal_Strain_Endo_seg005_point4 - Longitudinal_Strain_Endo_seg005_point3)/Time_duration3

Longitudinal_Strain_Endo_seg005_a4 = (Longitudinal_Strain_Endo_seg005_point5 - Longitudinal_Strain_Endo_seg005_point4)/Time_duration4

Longitudinal_Strain_Endo_seg005_a_min = min(c(Longitudinal_Strain_Endo_seg005_a1,Longitudinal_Strain_Endo_seg005_a2,Longitudinal_Strain_Endo_seg005_a3,Longitudinal_Strain_Endo_seg005_a4))

Longitudinal_Strain_Endo_seg005_a_max = max(c(Longitudinal_Strain_Endo_seg005_a1,Longitudinal_Strain_Endo_seg005_a2,Longitudinal_Strain_Endo_seg005_a3,Longitudinal_Strain_Endo_seg005_a4))

Longitudinal_Strain_Endo_seg006_point1 = as.numeric(Strain_data[26,col1])

Longitudinal_Strain_Endo_seg006_point2 = as.numeric(Strain_data[26,col2])

Longitudinal_Strain_Endo_seg006_point3 = as.numeric(Strain_data[26,col3])

Longitudinal_Strain_Endo_seg006_point4 = as.numeric(Strain_data[26,col4])

Longitudinal_Strain_Endo_seg006_point5 = as.numeric(Strain_data[26,col5])

Longitudinal_Strain_Endo_seg006_a1 = (Longitudinal_Strain_Endo_seg006_point2 - Longitudinal_Strain_Endo_seg006_point1)/Time_duration1

Longitudinal_Strain_Endo_seg006_a2 = (Longitudinal_Strain_Endo_seg006_point3 - Longitudinal_Strain_Endo_seg006_point2)/Time_duration2

Longitudinal_Strain_Endo_seg006_a3 = (Longitudinal_Strain_Endo_seg006_point4 - Longitudinal_Strain_Endo_seg006_point3)/Time_duration3

Longitudinal_Strain_Endo_seg006_a4 = (Longitudinal_Strain_Endo_seg006_point5 - Longitudinal_Strain_Endo_seg006_point4)/Time_duration4

Longitudinal_Strain_Endo_seg006_a_min = min(c(Longitudinal_Strain_Endo_seg006_a1,Longitudinal_Strain_Endo_seg006_a2,Longitudinal_Strain_Endo_seg006_a3,Longitudinal_Strain_Endo_seg006_a4))

Longitudinal_Strain_Endo_seg006_a_max = max(c(Longitudinal_Strain_Endo_seg006_a1,Longitudinal_Strain_Endo_seg006_a2,Longitudinal_Strain_Endo_seg006_a3,Longitudinal_Strain_Endo_seg006_a4))

##Average_values_per_frame_Longitudinal_Strain_Endo

Average_values_per_frame_Longitudinal_Strain_Endo_point1 = as.numeric(Strain_data[30,col1])

Average_values_per_frame_Longitudinal_Strain_Endo_point2 = as.numeric(Strain_data[30,col2])

Average_values_per_frame_Longitudinal_Strain_Endo_point3 = as.numeric(Strain_data[30,col3])

Average_values_per_frame_Longitudinal_Strain_Endo_point4 = as.numeric(Strain_data[30,col4])

Average_values_per_frame_Longitudinal_Strain_Endo_point5 = as.numeric(Strain_data[30,col5])

Average_values_per_frame_Longitudinal_Strain_Endo_a1 = (Average_values_per_frame_Longitudinal_Strain_Endo_point2 - Average_values_per_frame_Longitudinal_Strain_Endo_point1)/Time_duration1

Average_values_per_frame_Longitudinal_Strain_Endo_a2 = (Average_values_per_frame_Longitudinal_Strain_Endo_point3 - Average_values_per_frame_Longitudinal_Strain_Endo_point2)/Time_duration2

Average_values_per_frame_Longitudinal_Strain_Endo_a3 = (Average_values_per_frame_Longitudinal_Strain_Endo_point4 - Average_values_per_frame_Longitudinal_Strain_Endo_point3)/Time_duration3

Average_values_per_frame_Longitudinal_Strain_Endo_a4 = (Average_values_per_frame_Longitudinal_Strain_Endo_point5 - Average_values_per_frame_Longitudinal_Strain_Endo_point4)/Time_duration4

Average_values_per_frame_Longitudinal_Strain_Endo_a_min = min(c(Average_values_per_frame_Longitudinal_Strain_Endo_a1,Average_values_per_frame_Longitudinal_Strain_Endo_a2,Average_values_per_frame_Longitudinal_Strain_Endo_a3,Average_values_per_frame_Longitudinal_Strain_Endo_a4))

Average_values_per_frame_Longitudinal_Strain_Endo_a_max = max(c(Average_values_per_frame_Longitudinal_Strain_Endo_a1,Average_values_per_frame_Longitudinal_Strain_Endo_a2,Average_values_per_frame_Longitudinal_Strain_Endo_a3,Average_values_per_frame_Longitudinal_Strain_Endo_a4))

##Radial_Strain_Epi

Radial_Strain_Epi_seg001_point1 = as.numeric(Strain_data[34,col1])

Radial_Strain_Epi_seg001_point2 = as.numeric(Strain_data[34,col2])

Radial_Strain_Epi_seg001_point3 = as.numeric(Strain_data[34,col3])

Radial_Strain_Epi_seg001_point4 = as.numeric(Strain_data[34,col4])

Radial_Strain_Epi_seg001_point5 = as.numeric(Strain_data[34,col5])

Radial_Strain_Epi_seg001_a1 = (Radial_Strain_Epi_seg001_point2 - Radial_Strain_Epi_seg001_point1)/Time_duration1

Radial_Strain_Epi_seg001_a2 = (Radial_Strain_Epi_seg001_point3 - Radial_Strain_Epi_seg001_point2)/Time_duration2

Radial_Strain_Epi_seg001_a3 = (Radial_Strain_Epi_seg001_point4 - Radial_Strain_Epi_seg001_point3)/Time_duration3

Radial_Strain_Epi_seg001_a4 = (Radial_Strain_Epi_seg001_point5 - Radial_Strain_Epi_seg001_point4)/Time_duration4

Radial_Strain_Epi_seg001_a_min = min(c(Radial_Strain_Epi_seg001_a1,Radial_Strain_Epi_seg001_a2,Radial_Strain_Epi_seg001_a3,Radial_Strain_Epi_seg001_a4))

Radial_Strain_Epi_seg001_a_max = max(c(Radial_Strain_Epi_seg001_a1,Radial_Strain_Epi_seg001_a2,Radial_Strain_Epi_seg001_a3,Radial_Strain_Epi_seg001_a4))

Radial_Strain_Epi_seg002_point1 = as.numeric(Strain_data[35,col1])

Radial_Strain_Epi_seg002_point2 = as.numeric(Strain_data[35,col2])

Radial_Strain_Epi_seg002_point3 = as.numeric(Strain_data[35,col3])

Radial_Strain_Epi_seg002_point4 = as.numeric(Strain_data[35,col4])

Radial_Strain_Epi_seg002_point5 = as.numeric(Strain_data[35,col5])

Radial_Strain_Epi_seg002_a1 = (Radial_Strain_Epi_seg002_point2 - Radial_Strain_Epi_seg002_point1)/Time_duration1

Radial_Strain_Epi_seg002_a2 = (Radial_Strain_Epi_seg002_point3 - Radial_Strain_Epi_seg002_point2)/Time_duration2

Radial_Strain_Epi_seg002_a3 = (Radial_Strain_Epi_seg002_point4 - Radial_Strain_Epi_seg002_point3)/Time_duration3

Radial_Strain_Epi_seg002_a4 = (Radial_Strain_Epi_seg002_point5 - Radial_Strain_Epi_seg002_point4)/Time_duration4

Radial_Strain_Epi_seg002_a_min = min(c(Radial_Strain_Epi_seg002_a1,Radial_Strain_Epi_seg002_a2,Radial_Strain_Epi_seg002_a3,Radial_Strain_Epi_seg002_a4))

Radial_Strain_Epi_seg002_a_max = max(c(Radial_Strain_Epi_seg002_a1,Radial_Strain_Epi_seg002_a2,Radial_Strain_Epi_seg002_a3,Radial_Strain_Epi_seg002_a4))

Radial_Strain_Epi_seg003_point1 = as.numeric(Strain_data[36,col1])

Radial_Strain_Epi_seg003_point2 = as.numeric(Strain_data[36,col2])

Radial_Strain_Epi_seg003_point3 = as.numeric(Strain_data[36,col3])

Radial_Strain_Epi_seg003_point4 = as.numeric(Strain_data[36,col4])

Radial_Strain_Epi_seg003_point5 = as.numeric(Strain_data[36,col5])

Radial_Strain_Epi_seg003_a1 = (Radial_Strain_Epi_seg003_point2 - Radial_Strain_Epi_seg003_point1)/Time_duration1

Radial_Strain_Epi_seg003_a2 = (Radial_Strain_Epi_seg003_point3 - Radial_Strain_Epi_seg003_point2)/Time_duration2

Radial_Strain_Epi_seg003_a3 = (Radial_Strain_Epi_seg003_point4 - Radial_Strain_Epi_seg003_point3)/Time_duration3

Radial_Strain_Epi_seg003_a4 = (Radial_Strain_Epi_seg003_point5 - Radial_Strain_Epi_seg003_point4)/Time_duration4

Radial_Strain_Epi_seg003_a_min = min(c(Radial_Strain_Epi_seg003_a1,Radial_Strain_Epi_seg003_a2,Radial_Strain_Epi_seg003_a3,Radial_Strain_Epi_seg003_a4))

Radial_Strain_Epi_seg003_a_max = max(c(Radial_Strain_Epi_seg003_a1,Radial_Strain_Epi_seg003_a2,Radial_Strain_Epi_seg003_a3,Radial_Strain_Epi_seg003_a4))

Radial_Strain_Epi_seg004_point1 = as.numeric(Strain_data[37,col1])

Radial_Strain_Epi_seg004_point2 = as.numeric(Strain_data[37,col2])

Radial_Strain_Epi_seg004_point3 = as.numeric(Strain_data[37,col3])

Radial_Strain_Epi_seg004_point4 = as.numeric(Strain_data[37,col4])

Radial_Strain_Epi_seg004_point5 = as.numeric(Strain_data[37,col5])

Radial_Strain_Epi_seg004_a1 = (Radial_Strain_Epi_seg004_point2 - Radial_Strain_Epi_seg004_point1)/Time_duration1

Radial_Strain_Epi_seg004_a2 = (Radial_Strain_Epi_seg004_point3 - Radial_Strain_Epi_seg004_point2)/Time_duration2

Radial_Strain_Epi_seg004_a3 = (Radial_Strain_Epi_seg004_point4 - Radial_Strain_Epi_seg004_point3)/Time_duration3

Radial_Strain_Epi_seg004_a4 = (Radial_Strain_Epi_seg004_point5 - Radial_Strain_Epi_seg004_point4)/Time_duration4

Radial_Strain_Epi_seg004_a_min = min(c(Radial_Strain_Epi_seg004_a1,Radial_Strain_Epi_seg004_a2,Radial_Strain_Epi_seg004_a3,Radial_Strain_Epi_seg004_a4))

Radial_Strain_Epi_seg004_a_max = max(c(Radial_Strain_Epi_seg004_a1,Radial_Strain_Epi_seg004_a2,Radial_Strain_Epi_seg004_a3,Radial_Strain_Epi_seg004_a4))

Radial_Strain_Epi_seg005_point1 = as.numeric(Strain_data[38,col1])

Radial_Strain_Epi_seg005_point2 = as.numeric(Strain_data[38,col2])

Radial_Strain_Epi_seg005_point3 = as.numeric(Strain_data[38,col3])

Radial_Strain_Epi_seg005_point4 = as.numeric(Strain_data[38,col4])

Radial_Strain_Epi_seg005_point5 = as.numeric(Strain_data[38,col5])

Radial_Strain_Epi_seg005_a1 = (Radial_Strain_Epi_seg005_point2 - Radial_Strain_Epi_seg005_point1)/Time_duration1

Radial_Strain_Epi_seg005_a2 = (Radial_Strain_Epi_seg005_point3 - Radial_Strain_Epi_seg005_point2)/Time_duration2

Radial_Strain_Epi_seg005_a3 = (Radial_Strain_Epi_seg005_point4 - Radial_Strain_Epi_seg005_point3)/Time_duration3

Radial_Strain_Epi_seg005_a4 = (Radial_Strain_Epi_seg005_point5 - Radial_Strain_Epi_seg005_point4)/Time_duration4

Radial_Strain_Epi_seg005_a_min = min(c(Radial_Strain_Epi_seg005_a1,Radial_Strain_Epi_seg005_a2,Radial_Strain_Epi_seg005_a3,Radial_Strain_Epi_seg005_a4))

Radial_Strain_Epi_seg005_a_max = max(c(Radial_Strain_Epi_seg005_a1,Radial_Strain_Epi_seg005_a2,Radial_Strain_Epi_seg005_a3,Radial_Strain_Epi_seg005_a4))

Radial_Strain_Epi_seg006_point1 = as.numeric(Strain_data[39,col1])

Radial_Strain_Epi_seg006_point2 = as.numeric(Strain_data[39,col2])

Radial_Strain_Epi_seg006_point3 = as.numeric(Strain_data[39,col3])

Radial_Strain_Epi_seg006_point4 = as.numeric(Strain_data[39,col4])

Radial_Strain_Epi_seg006_point5 = as.numeric(Strain_data[39,col5])

Radial_Strain_Epi_seg006_a1 = (Radial_Strain_Epi_seg006_point2 - Radial_Strain_Epi_seg006_point1)/Time_duration1

Radial_Strain_Epi_seg006_a2 = (Radial_Strain_Epi_seg006_point3 - Radial_Strain_Epi_seg006_point2)/Time_duration2

Radial_Strain_Epi_seg006_a3 = (Radial_Strain_Epi_seg006_point4 - Radial_Strain_Epi_seg006_point3)/Time_duration3

Radial_Strain_Epi_seg006_a4 = (Radial_Strain_Epi_seg006_point5 - Radial_Strain_Epi_seg006_point4)/Time_duration4

Radial_Strain_Epi_seg006_a_min = min(c(Radial_Strain_Epi_seg006_a1,Radial_Strain_Epi_seg006_a2,Radial_Strain_Epi_seg006_a3,Radial_Strain_Epi_seg006_a4))

Radial_Strain_Epi_seg006_a_max = max(c(Radial_Strain_Epi_seg006_a1,Radial_Strain_Epi_seg006_a2,Radial_Strain_Epi_seg006_a3,Radial_Strain_Epi_seg006_a4))

##Average_values_per_frame_Radial_Strain_Epi

Average_values_per_frame_Radial_Strain_Epi_point1 = as.numeric(Strain_data[43,col1])

Average_values_per_frame_Radial_Strain_Epi_point2 = as.numeric(Strain_data[43,col2])

Average_values_per_frame_Radial_Strain_Epi_point3 = as.numeric(Strain_data[43,col3])

Average_values_per_frame_Radial_Strain_Epi_point4 = as.numeric(Strain_data[43,col4])

Average_values_per_frame_Radial_Strain_Epi_point5 = as.numeric(Strain_data[43,col5])

Average_values_per_frame_Radial_Strain_Epi_a1 = (Average_values_per_frame_Radial_Strain_Epi_point2 - Average_values_per_frame_Radial_Strain_Epi_point1)/Time_duration1

Average_values_per_frame_Radial_Strain_Epi_a2 = (Average_values_per_frame_Radial_Strain_Epi_point3 - Average_values_per_frame_Radial_Strain_Epi_point2)/Time_duration2

Average_values_per_frame_Radial_Strain_Epi_a3 = (Average_values_per_frame_Radial_Strain_Epi_point4 - Average_values_per_frame_Radial_Strain_Epi_point3)/Time_duration3

Average_values_per_frame_Radial_Strain_Epi_a4 = (Average_values_per_frame_Radial_Strain_Epi_point5 - Average_values_per_frame_Radial_Strain_Epi_point4)/Time_duration4

Average_values_per_frame_Radial_Strain_Epi_a_min = min(c(Average_values_per_frame_Radial_Strain_Epi_a1,Average_values_per_frame_Radial_Strain_Epi_a2,Average_values_per_frame_Radial_Strain_Epi_a3,Average_values_per_frame_Radial_Strain_Epi_a4))

Average_values_per_frame_Radial_Strain_Epi_a_max = max(c(Average_values_per_frame_Radial_Strain_Epi_a1,Average_values_per_frame_Radial_Strain_Epi_a2,Average_values_per_frame_Radial_Strain_Epi_a3,Average_values_per_frame_Radial_Strain_Epi_a4))

##Longitudinal_Strain_Epi

Longitudinal_Strain_Epi_seg001_point1 = as.numeric(Strain_data[47,col1])

Longitudinal_Strain_Epi_seg001_point2 = as.numeric(Strain_data[47,col2])

Longitudinal_Strain_Epi_seg001_point3 = as.numeric(Strain_data[47,col3])

Longitudinal_Strain_Epi_seg001_point4 = as.numeric(Strain_data[47,col4])

Longitudinal_Strain_Epi_seg001_point5 = as.numeric(Strain_data[47,col5])

Longitudinal_Strain_Epi_seg001_a1 = (Longitudinal_Strain_Epi_seg001_point2 - Longitudinal_Strain_Epi_seg001_point1)/Time_duration1

Longitudinal_Strain_Epi_seg001_a2 = (Longitudinal_Strain_Epi_seg001_point3 - Longitudinal_Strain_Epi_seg001_point2)/Time_duration2

Longitudinal_Strain_Epi_seg001_a3 = (Longitudinal_Strain_Epi_seg001_point4 - Longitudinal_Strain_Epi_seg001_point3)/Time_duration3

Longitudinal_Strain_Epi_seg001_a4 = (Longitudinal_Strain_Epi_seg001_point5 - Longitudinal_Strain_Epi_seg001_point4)/Time_duration4

Longitudinal_Strain_Epi_seg001_a_min = min(c(Longitudinal_Strain_Epi_seg001_a1,Longitudinal_Strain_Epi_seg001_a2,Longitudinal_Strain_Epi_seg001_a3,Longitudinal_Strain_Epi_seg001_a4))

Longitudinal_Strain_Epi_seg001_a_max = max(c(Longitudinal_Strain_Epi_seg001_a1,Longitudinal_Strain_Epi_seg001_a2,Longitudinal_Strain_Epi_seg001_a3,Longitudinal_Strain_Epi_seg001_a4))

Longitudinal_Strain_Epi_seg002_point1 = as.numeric(Strain_data[48,col1])

Longitudinal_Strain_Epi_seg002_point2 = as.numeric(Strain_data[48,col2])

Longitudinal_Strain_Epi_seg002_point3 = as.numeric(Strain_data[48,col3])

Longitudinal_Strain_Epi_seg002_point4 = as.numeric(Strain_data[48,col4])

Longitudinal_Strain_Epi_seg002_point5 = as.numeric(Strain_data[48,col5])

Longitudinal_Strain_Epi_seg002_a1 = (Longitudinal_Strain_Epi_seg002_point2 - Longitudinal_Strain_Epi_seg002_point1)/Time_duration1

Longitudinal_Strain_Epi_seg002_a2 = (Longitudinal_Strain_Epi_seg002_point3 - Longitudinal_Strain_Epi_seg002_point2)/Time_duration2

Longitudinal_Strain_Epi_seg002_a3 = (Longitudinal_Strain_Epi_seg002_point4 - Longitudinal_Strain_Epi_seg002_point3)/Time_duration3

Longitudinal_Strain_Epi_seg002_a4 = (Longitudinal_Strain_Epi_seg002_point5 - Longitudinal_Strain_Epi_seg002_point4)/Time_duration4

Longitudinal_Strain_Epi_seg002_a_min = min(c(Longitudinal_Strain_Epi_seg002_a1,Longitudinal_Strain_Epi_seg002_a2,Longitudinal_Strain_Epi_seg002_a3,Longitudinal_Strain_Epi_seg002_a4))

Longitudinal_Strain_Epi_seg002_a_max = max(c(Longitudinal_Strain_Epi_seg002_a1,Longitudinal_Strain_Epi_seg002_a2,Longitudinal_Strain_Epi_seg002_a3,Longitudinal_Strain_Epi_seg002_a4))

Longitudinal_Strain_Epi_seg003_point1 = as.numeric(Strain_data[49,col1])

Longitudinal_Strain_Epi_seg003_point2 = as.numeric(Strain_data[49,col2])

Longitudinal_Strain_Epi_seg003_point3 = as.numeric(Strain_data[49,col3])

Longitudinal_Strain_Epi_seg003_point4 = as.numeric(Strain_data[49,col4])

Longitudinal_Strain_Epi_seg003_point5 = as.numeric(Strain_data[49,col5])

Longitudinal_Strain_Epi_seg003_a1 = (Longitudinal_Strain_Epi_seg003_point2 - Longitudinal_Strain_Epi_seg003_point1)/Time_duration1

Longitudinal_Strain_Epi_seg003_a2 = (Longitudinal_Strain_Epi_seg003_point3 - Longitudinal_Strain_Epi_seg003_point2)/Time_duration2

Longitudinal_Strain_Epi_seg003_a3 = (Longitudinal_Strain_Epi_seg003_point4 - Longitudinal_Strain_Epi_seg003_point3)/Time_duration3

Longitudinal_Strain_Epi_seg003_a4 = (Longitudinal_Strain_Epi_seg003_point5 - Longitudinal_Strain_Epi_seg003_point4)/Time_duration4

Longitudinal_Strain_Epi_seg003_a_min = min(c(Longitudinal_Strain_Epi_seg003_a1,Longitudinal_Strain_Epi_seg003_a2,Longitudinal_Strain_Epi_seg003_a3,Longitudinal_Strain_Epi_seg003_a4))

Longitudinal_Strain_Epi_seg003_a_max = max(c(Longitudinal_Strain_Epi_seg003_a1,Longitudinal_Strain_Epi_seg003_a2,Longitudinal_Strain_Epi_seg003_a3,Longitudinal_Strain_Epi_seg003_a4))

Longitudinal_Strain_Epi_seg004_point1 = as.numeric(Strain_data[50,col1])

Longitudinal_Strain_Epi_seg004_point2 = as.numeric(Strain_data[50,col2])

Longitudinal_Strain_Epi_seg004_point3 = as.numeric(Strain_data[50,col3])

Longitudinal_Strain_Epi_seg004_point4 = as.numeric(Strain_data[50,col4])

Longitudinal_Strain_Epi_seg004_point5 = as.numeric(Strain_data[50,col5])

Longitudinal_Strain_Epi_seg004_a1 = (Longitudinal_Strain_Epi_seg004_point2 - Longitudinal_Strain_Epi_seg004_point1)/Time_duration1

Longitudinal_Strain_Epi_seg004_a2 = (Longitudinal_Strain_Epi_seg004_point3 - Longitudinal_Strain_Epi_seg004_point2)/Time_duration2

Longitudinal_Strain_Epi_seg004_a3 = (Longitudinal_Strain_Epi_seg004_point4 - Longitudinal_Strain_Epi_seg004_point3)/Time_duration3

Longitudinal_Strain_Epi_seg004_a4 = (Longitudinal_Strain_Epi_seg004_point5 - Longitudinal_Strain_Epi_seg004_point4)/Time_duration4

Longitudinal_Strain_Epi_seg004_a_min = min(c(Longitudinal_Strain_Epi_seg004_a1,Longitudinal_Strain_Epi_seg004_a2,Longitudinal_Strain_Epi_seg004_a3,Longitudinal_Strain_Epi_seg004_a4))

Longitudinal_Strain_Epi_seg004_a_max = max(c(Longitudinal_Strain_Epi_seg004_a1,Longitudinal_Strain_Epi_seg004_a2,Longitudinal_Strain_Epi_seg004_a3,Longitudinal_Strain_Epi_seg004_a4))

Longitudinal_Strain_Epi_seg005_point1 = as.numeric(Strain_data[51,col1])

Longitudinal_Strain_Epi_seg005_point2 = as.numeric(Strain_data[51,col2])

Longitudinal_Strain_Epi_seg005_point3 = as.numeric(Strain_data[51,col3])

Longitudinal_Strain_Epi_seg005_point4 = as.numeric(Strain_data[51,col4])

Longitudinal_Strain_Epi_seg005_point5 = as.numeric(Strain_data[51,col5])

Longitudinal_Strain_Epi_seg005_a1 = (Longitudinal_Strain_Epi_seg005_point2 - Longitudinal_Strain_Epi_seg005_point1)/Time_duration1

Longitudinal_Strain_Epi_seg005_a2 = (Longitudinal_Strain_Epi_seg005_point3 - Longitudinal_Strain_Epi_seg005_point2)/Time_duration2

Longitudinal_Strain_Epi_seg005_a3 = (Longitudinal_Strain_Epi_seg005_point4 - Longitudinal_Strain_Epi_seg005_point3)/Time_duration3

Longitudinal_Strain_Epi_seg005_a4 = (Longitudinal_Strain_Epi_seg005_point5 - Longitudinal_Strain_Epi_seg005_point4)/Time_duration4

Longitudinal_Strain_Epi_seg005_a_min = min(c(Longitudinal_Strain_Epi_seg005_a1,Longitudinal_Strain_Epi_seg005_a2,Longitudinal_Strain_Epi_seg005_a3,Longitudinal_Strain_Epi_seg005_a4))

Longitudinal_Strain_Epi_seg005_a_max = max(c(Longitudinal_Strain_Epi_seg005_a1,Longitudinal_Strain_Epi_seg005_a2,Longitudinal_Strain_Epi_seg005_a3,Longitudinal_Strain_Epi_seg005_a4))

Longitudinal_Strain_Epi_seg006_point1 = as.numeric(Strain_data[52,col1])

Longitudinal_Strain_Epi_seg006_point2 = as.numeric(Strain_data[52,col2])

Longitudinal_Strain_Epi_seg006_point3 = as.numeric(Strain_data[52,col3])

Longitudinal_Strain_Epi_seg006_point4 = as.numeric(Strain_data[52,col4])

Longitudinal_Strain_Epi_seg006_point5 = as.numeric(Strain_data[52,col5])

Longitudinal_Strain_Epi_seg006_a1 = (Longitudinal_Strain_Epi_seg006_point2 - Longitudinal_Strain_Epi_seg006_point1)/Time_duration1

Longitudinal_Strain_Epi_seg006_a2 = (Longitudinal_Strain_Epi_seg006_point3 - Longitudinal_Strain_Epi_seg006_point2)/Time_duration2

Longitudinal_Strain_Epi_seg006_a3 = (Longitudinal_Strain_Epi_seg006_point4 - Longitudinal_Strain_Epi_seg006_point3)/Time_duration3

Longitudinal_Strain_Epi_seg006_a4 = (Longitudinal_Strain_Epi_seg006_point5 - Longitudinal_Strain_Epi_seg006_point4)/Time_duration4

Longitudinal_Strain_Epi_seg006_a_min = min(c(Longitudinal_Strain_Epi_seg006_a1,Longitudinal_Strain_Epi_seg006_a2,Longitudinal_Strain_Epi_seg006_a3,Longitudinal_Strain_Epi_seg006_a4))

Longitudinal_Strain_Epi_seg006_a_max = max(c(Longitudinal_Strain_Epi_seg006_a1,Longitudinal_Strain_Epi_seg006_a2,Longitudinal_Strain_Epi_seg006_a3,Longitudinal_Strain_Epi_seg006_a4))

##Average_values_per_frame_Longitudinal_Strain_Epi

Average_values_per_frame_Longitudinal_Strain_Epi_point1 = as.numeric(Strain_data[56,col1])

Average_values_per_frame_Longitudinal_Strain_Epi_point2 = as.numeric(Strain_data[56,col2])

Average_values_per_frame_Longitudinal_Strain_Epi_point3 = as.numeric(Strain_data[56,col3])

Average_values_per_frame_Longitudinal_Strain_Epi_point4 = as.numeric(Strain_data[56,col4])

Average_values_per_frame_Longitudinal_Strain_Epi_point5 = as.numeric(Strain_data[56,col5])

Average_values_per_frame_Longitudinal_Strain_Epi_a1 = (Average_values_per_frame_Longitudinal_Strain_Epi_point2 - Average_values_per_frame_Longitudinal_Strain_Epi_point1)/Time_duration1

Average_values_per_frame_Longitudinal_Strain_Epi_a2 = (Average_values_per_frame_Longitudinal_Strain_Epi_point3 - Average_values_per_frame_Longitudinal_Strain_Epi_point2)/Time_duration2

Average_values_per_frame_Longitudinal_Strain_Epi_a3 = (Average_values_per_frame_Longitudinal_Strain_Epi_point4 - Average_values_per_frame_Longitudinal_Strain_Epi_point3)/Time_duration3

Average_values_per_frame_Longitudinal_Strain_Epi_a4 = (Average_values_per_frame_Longitudinal_Strain_Epi_point5 - Average_values_per_frame_Longitudinal_Strain_Epi_point4)/Time_duration4

Average_values_per_frame_Longitudinal_Strain_Epi_a_min = min(c(Average_values_per_frame_Longitudinal_Strain_Epi_a1,Average_values_per_frame_Longitudinal_Strain_Epi_a2,Average_values_per_frame_Longitudinal_Strain_Epi_a3,Average_values_per_frame_Longitudinal_Strain_Epi_a4))

Average_values_per_frame_Longitudinal_Strain_Epi_a_max = max(c(Average_values_per_frame_Longitudinal_Strain_Epi_a1,Average_values_per_frame_Longitudinal_Strain_Epi_a2,Average_values_per_frame_Longitudinal_Strain_Epi_a3,Average_values_per_frame_Longitudinal_Strain_Epi_a4))

#Strain_rate

Strain_Rate_data <- read.xlsx(i,sheetIndex = 5, header = F)

colnames(Strain_Rate_data) <- Strain_Rate_data[3,]

name1 = paste("fr0",time3,sep = "")

col5 = grep(name1,colnames(Strain_Rate_data))

col4 = col5-1

col3 = col5-2

col2 = col5-3

col1 = col5-4

Timepoint1 = as.numeric(Strain_Rate_data[4,col1])

Timepoint2 = as.numeric(Strain_Rate_data[4,col2])

Timepoint3 = as.numeric(Strain_Rate_data[4,col3])

Timepoint4 = as.numeric(Strain_Rate_data[4,col4])

Timepoint5 = as.numeric(Strain_Rate_data[4,col5])

Time_duration1 = Timepoint2 - Timepoint1

Time_duration2 = Timepoint3 - Timepoint2

Time_duration3 = Timepoint4 - Timepoint3

Time_duration4 = Timepoint5 - Timepoint4

##Radial_Strain_Rate_Endo

Radial_Strain_Rate_Endo_seg001_point1 = as.numeric(Strain_Rate_data[8,col1])

Radial_Strain_Rate_Endo_seg001_point2 = as.numeric(Strain_Rate_data[8,col2])

Radial_Strain_Rate_Endo_seg001_point3 = as.numeric(Strain_Rate_data[8,col3])

Radial_Strain_Rate_Endo_seg001_point4 = as.numeric(Strain_Rate_data[8,col4])

Radial_Strain_Rate_Endo_seg001_point5 = as.numeric(Strain_Rate_data[8,col5])

Radial_Strain_Rate_Endo_seg001_a1 = (Radial_Strain_Rate_Endo_seg001_point2 - Radial_Strain_Rate_Endo_seg001_point1)/Time_duration1

Radial_Strain_Rate_Endo_seg001_a2 = (Radial_Strain_Rate_Endo_seg001_point3 - Radial_Strain_Rate_Endo_seg001_point2)/Time_duration2

Radial_Strain_Rate_Endo_seg001_a3 = (Radial_Strain_Rate_Endo_seg001_point4 - Radial_Strain_Rate_Endo_seg001_point3)/Time_duration3

Radial_Strain_Rate_Endo_seg001_a4 = (Radial_Strain_Rate_Endo_seg001_point5 - Radial_Strain_Rate_Endo_seg001_point4)/Time_duration4

Radial_Strain_Rate_Endo_seg001_a_min = min(c(Radial_Strain_Rate_Endo_seg001_a1,Radial_Strain_Rate_Endo_seg001_a2,Radial_Strain_Rate_Endo_seg001_a3,Radial_Strain_Rate_Endo_seg001_a4))

Radial_Strain_Rate_Endo_seg001_a_max = max(c(Radial_Strain_Rate_Endo_seg001_a1,Radial_Strain_Rate_Endo_seg001_a2,Radial_Strain_Rate_Endo_seg001_a3,Radial_Strain_Rate_Endo_seg001_a4))

Radial_Strain_Rate_Endo_seg002_point1 = as.numeric(Strain_Rate_data[9,col1])

Radial_Strain_Rate_Endo_seg002_point2 = as.numeric(Strain_Rate_data[9,col2])

Radial_Strain_Rate_Endo_seg002_point3 = as.numeric(Strain_Rate_data[9,col3])

Radial_Strain_Rate_Endo_seg002_point4 = as.numeric(Strain_Rate_data[9,col4])

Radial_Strain_Rate_Endo_seg002_point5 = as.numeric(Strain_Rate_data[9,col5])

Radial_Strain_Rate_Endo_seg002_a1 = (Radial_Strain_Rate_Endo_seg002_point2 - Radial_Strain_Rate_Endo_seg002_point1)/Time_duration1

Radial_Strain_Rate_Endo_seg002_a2 = (Radial_Strain_Rate_Endo_seg002_point3 - Radial_Strain_Rate_Endo_seg002_point2)/Time_duration2

Radial_Strain_Rate_Endo_seg002_a3 = (Radial_Strain_Rate_Endo_seg002_point4 - Radial_Strain_Rate_Endo_seg002_point3)/Time_duration3

Radial_Strain_Rate_Endo_seg002_a4 = (Radial_Strain_Rate_Endo_seg002_point5 - Radial_Strain_Rate_Endo_seg002_point4)/Time_duration4

Radial_Strain_Rate_Endo_seg002_a_min = min(c(Radial_Strain_Rate_Endo_seg002_a1,Radial_Strain_Rate_Endo_seg002_a2,Radial_Strain_Rate_Endo_seg002_a3,Radial_Strain_Rate_Endo_seg002_a4))

Radial_Strain_Rate_Endo_seg002_a_max = max(c(Radial_Strain_Rate_Endo_seg002_a1,Radial_Strain_Rate_Endo_seg002_a2,Radial_Strain_Rate_Endo_seg002_a3,Radial_Strain_Rate_Endo_seg002_a4))

Radial_Strain_Rate_Endo_seg003_point1 = as.numeric(Strain_Rate_data[10,col1])

Radial_Strain_Rate_Endo_seg003_point2 = as.numeric(Strain_Rate_data[10,col2])

Radial_Strain_Rate_Endo_seg003_point3 = as.numeric(Strain_Rate_data[10,col3])

Radial_Strain_Rate_Endo_seg003_point4 = as.numeric(Strain_Rate_data[10,col4])

Radial_Strain_Rate_Endo_seg003_point5 = as.numeric(Strain_Rate_data[10,col5])

Radial_Strain_Rate_Endo_seg003_a1 = (Radial_Strain_Rate_Endo_seg003_point2 - Radial_Strain_Rate_Endo_seg003_point1)/Time_duration1

Radial_Strain_Rate_Endo_seg003_a2 = (Radial_Strain_Rate_Endo_seg003_point3 - Radial_Strain_Rate_Endo_seg003_point2)/Time_duration2

Radial_Strain_Rate_Endo_seg003_a3 = (Radial_Strain_Rate_Endo_seg003_point4 - Radial_Strain_Rate_Endo_seg003_point3)/Time_duration3

Radial_Strain_Rate_Endo_seg003_a4 = (Radial_Strain_Rate_Endo_seg003_point5 - Radial_Strain_Rate_Endo_seg003_point4)/Time_duration4

Radial_Strain_Rate_Endo_seg003_a_min = min(c(Radial_Strain_Rate_Endo_seg003_a1,Radial_Strain_Rate_Endo_seg003_a2,Radial_Strain_Rate_Endo_seg003_a3,Radial_Strain_Rate_Endo_seg003_a4))

Radial_Strain_Rate_Endo_seg003_a_max = max(c(Radial_Strain_Rate_Endo_seg003_a1,Radial_Strain_Rate_Endo_seg003_a2,Radial_Strain_Rate_Endo_seg003_a3,Radial_Strain_Rate_Endo_seg003_a4))

Radial_Strain_Rate_Endo_seg004_point1 = as.numeric(Strain_Rate_data[11,col1])

Radial_Strain_Rate_Endo_seg004_point2 = as.numeric(Strain_Rate_data[11,col2])

Radial_Strain_Rate_Endo_seg004_point3 = as.numeric(Strain_Rate_data[11,col3])

Radial_Strain_Rate_Endo_seg004_point4 = as.numeric(Strain_Rate_data[11,col4])

Radial_Strain_Rate_Endo_seg004_point5 = as.numeric(Strain_Rate_data[11,col5])

Radial_Strain_Rate_Endo_seg004_a1 = (Radial_Strain_Rate_Endo_seg004_point2 - Radial_Strain_Rate_Endo_seg004_point1)/Time_duration1

Radial_Strain_Rate_Endo_seg004_a2 = (Radial_Strain_Rate_Endo_seg004_point3 - Radial_Strain_Rate_Endo_seg004_point2)/Time_duration2

Radial_Strain_Rate_Endo_seg004_a3 = (Radial_Strain_Rate_Endo_seg004_point4 - Radial_Strain_Rate_Endo_seg004_point3)/Time_duration3

Radial_Strain_Rate_Endo_seg004_a4 = (Radial_Strain_Rate_Endo_seg004_point5 - Radial_Strain_Rate_Endo_seg004_point4)/Time_duration4

Radial_Strain_Rate_Endo_seg004_a_min = min(c(Radial_Strain_Rate_Endo_seg004_a1,Radial_Strain_Rate_Endo_seg004_a2,Radial_Strain_Rate_Endo_seg004_a3,Radial_Strain_Rate_Endo_seg004_a4))

Radial_Strain_Rate_Endo_seg004_a_max = max(c(Radial_Strain_Rate_Endo_seg004_a1,Radial_Strain_Rate_Endo_seg004_a2,Radial_Strain_Rate_Endo_seg004_a3,Radial_Strain_Rate_Endo_seg004_a4))

Radial_Strain_Rate_Endo_seg005_point1 = as.numeric(Strain_Rate_data[12,col1])

Radial_Strain_Rate_Endo_seg005_point2 = as.numeric(Strain_Rate_data[12,col2])

Radial_Strain_Rate_Endo_seg005_point3 = as.numeric(Strain_Rate_data[12,col3])

Radial_Strain_Rate_Endo_seg005_point4 = as.numeric(Strain_Rate_data[12,col4])

Radial_Strain_Rate_Endo_seg005_point5 = as.numeric(Strain_Rate_data[12,col5])

Radial_Strain_Rate_Endo_seg005_a1 = (Radial_Strain_Rate_Endo_seg005_point2 - Radial_Strain_Rate_Endo_seg005_point1)/Time_duration1

Radial_Strain_Rate_Endo_seg005_a2 = (Radial_Strain_Rate_Endo_seg005_point3 - Radial_Strain_Rate_Endo_seg005_point2)/Time_duration2

Radial_Strain_Rate_Endo_seg005_a3 = (Radial_Strain_Rate_Endo_seg005_point4 - Radial_Strain_Rate_Endo_seg005_point3)/Time_duration3

Radial_Strain_Rate_Endo_seg005_a4 = (Radial_Strain_Rate_Endo_seg005_point5 - Radial_Strain_Rate_Endo_seg005_point4)/Time_duration4

Radial_Strain_Rate_Endo_seg005_a_min = min(c(Radial_Strain_Rate_Endo_seg005_a1,Radial_Strain_Rate_Endo_seg005_a2,Radial_Strain_Rate_Endo_seg005_a3,Radial_Strain_Rate_Endo_seg005_a4))

Radial_Strain_Rate_Endo_seg005_a_max = max(c(Radial_Strain_Rate_Endo_seg005_a1,Radial_Strain_Rate_Endo_seg005_a2,Radial_Strain_Rate_Endo_seg005_a3,Radial_Strain_Rate_Endo_seg005_a4))

Radial_Strain_Rate_Endo_seg006_point1 = as.numeric(Strain_Rate_data[13,col1])

Radial_Strain_Rate_Endo_seg006_point2 = as.numeric(Strain_Rate_data[13,col2])

Radial_Strain_Rate_Endo_seg006_point3 = as.numeric(Strain_Rate_data[13,col3])

Radial_Strain_Rate_Endo_seg006_point4 = as.numeric(Strain_Rate_data[13,col4])

Radial_Strain_Rate_Endo_seg006_point5 = as.numeric(Strain_Rate_data[13,col5])

Radial_Strain_Rate_Endo_seg006_a1 = (Radial_Strain_Rate_Endo_seg006_point2 - Radial_Strain_Rate_Endo_seg006_point1)/Time_duration1

Radial_Strain_Rate_Endo_seg006_a2 = (Radial_Strain_Rate_Endo_seg006_point3 - Radial_Strain_Rate_Endo_seg006_point2)/Time_duration2

Radial_Strain_Rate_Endo_seg006_a3 = (Radial_Strain_Rate_Endo_seg006_point4 - Radial_Strain_Rate_Endo_seg006_point3)/Time_duration3

Radial_Strain_Rate_Endo_seg006_a4 = (Radial_Strain_Rate_Endo_seg006_point5 - Radial_Strain_Rate_Endo_seg006_point4)/Time_duration4

Radial_Strain_Rate_Endo_seg006_a_min = min(c(Radial_Strain_Rate_Endo_seg006_a1,Radial_Strain_Rate_Endo_seg006_a2,Radial_Strain_Rate_Endo_seg006_a3,Radial_Strain_Rate_Endo_seg006_a4))

Radial_Strain_Rate_Endo_seg006_a_max = max(c(Radial_Strain_Rate_Endo_seg006_a1,Radial_Strain_Rate_Endo_seg006_a2,Radial_Strain_Rate_Endo_seg006_a3,Radial_Strain_Rate_Endo_seg006_a4))

##Average_values_per_frame_Radial_Strain_Rate_Endo

Average_values_per_frame_Radial_Strain_Rate_Endo_point1 = as.numeric(Strain_Rate_data[17,col1])

Average_values_per_frame_Radial_Strain_Rate_Endo_point2 = as.numeric(Strain_Rate_data[17,col2])

Average_values_per_frame_Radial_Strain_Rate_Endo_point3 = as.numeric(Strain_Rate_data[17,col3])

Average_values_per_frame_Radial_Strain_Rate_Endo_point4 = as.numeric(Strain_Rate_data[17,col4])

Average_values_per_frame_Radial_Strain_Rate_Endo_point5 = as.numeric(Strain_Rate_data[17,col5])

Average_values_per_frame_Radial_Strain_Rate_Endo_a1 = (Average_values_per_frame_Radial_Strain_Rate_Endo_point2 - Average_values_per_frame_Radial_Strain_Rate_Endo_point1)/Time_duration1

Average_values_per_frame_Radial_Strain_Rate_Endo_a2 = (Average_values_per_frame_Radial_Strain_Rate_Endo_point3 - Average_values_per_frame_Radial_Strain_Rate_Endo_point2)/Time_duration2

Average_values_per_frame_Radial_Strain_Rate_Endo_a3 = (Average_values_per_frame_Radial_Strain_Rate_Endo_point4 - Average_values_per_frame_Radial_Strain_Rate_Endo_point3)/Time_duration3

Average_values_per_frame_Radial_Strain_Rate_Endo_a4 = (Average_values_per_frame_Radial_Strain_Rate_Endo_point5 - Average_values_per_frame_Radial_Strain_Rate_Endo_point4)/Time_duration4

Average_values_per_frame_Radial_Strain_Rate_Endo_a_min = min(c(Average_values_per_frame_Radial_Strain_Rate_Endo_a1,Average_values_per_frame_Radial_Strain_Rate_Endo_a2,Average_values_per_frame_Radial_Strain_Rate_Endo_a3,Average_values_per_frame_Radial_Strain_Rate_Endo_a4))

Average_values_per_frame_Radial_Strain_Rate_Endo_a_max = max(c(Average_values_per_frame_Radial_Strain_Rate_Endo_a1,Average_values_per_frame_Radial_Strain_Rate_Endo_a2,Average_values_per_frame_Radial_Strain_Rate_Endo_a3,Average_values_per_frame_Radial_Strain_Rate_Endo_a4))

##Longitudinal_Strain_Rate_Endo

Longitudinal_Strain_Rate_Endo_seg001_point1 = as.numeric(Strain_Rate_data[21,col1])

Longitudinal_Strain_Rate_Endo_seg001_point2 = as.numeric(Strain_Rate_data[21,col2])

Longitudinal_Strain_Rate_Endo_seg001_point3 = as.numeric(Strain_Rate_data[21,col3])

Longitudinal_Strain_Rate_Endo_seg001_point4 = as.numeric(Strain_Rate_data[21,col4])

Longitudinal_Strain_Rate_Endo_seg001_point5 = as.numeric(Strain_Rate_data[21,col5])

Longitudinal_Strain_Rate_Endo_seg001_a1 = (Longitudinal_Strain_Rate_Endo_seg001_point2 - Longitudinal_Strain_Rate_Endo_seg001_point1)/Time_duration1

Longitudinal_Strain_Rate_Endo_seg001_a2 = (Longitudinal_Strain_Rate_Endo_seg001_point3 - Longitudinal_Strain_Rate_Endo_seg001_point2)/Time_duration2

Longitudinal_Strain_Rate_Endo_seg001_a3 = (Longitudinal_Strain_Rate_Endo_seg001_point4 - Longitudinal_Strain_Rate_Endo_seg001_point3)/Time_duration3

Longitudinal_Strain_Rate_Endo_seg001_a4 = (Longitudinal_Strain_Rate_Endo_seg001_point5 - Longitudinal_Strain_Rate_Endo_seg001_point4)/Time_duration4

Longitudinal_Strain_Rate_Endo_seg001_a_min = min(c(Longitudinal_Strain_Rate_Endo_seg001_a1,Longitudinal_Strain_Rate_Endo_seg001_a2,Longitudinal_Strain_Rate_Endo_seg001_a3,Longitudinal_Strain_Rate_Endo_seg001_a4))

Longitudinal_Strain_Rate_Endo_seg001_a_max = max(c(Longitudinal_Strain_Rate_Endo_seg001_a1,Longitudinal_Strain_Rate_Endo_seg001_a2,Longitudinal_Strain_Rate_Endo_seg001_a3,Longitudinal_Strain_Rate_Endo_seg001_a4))

Longitudinal_Strain_Rate_Endo_seg002_point1 = as.numeric(Strain_Rate_data[22,col1])

Longitudinal_Strain_Rate_Endo_seg002_point2 = as.numeric(Strain_Rate_data[22,col2])

Longitudinal_Strain_Rate_Endo_seg002_point3 = as.numeric(Strain_Rate_data[22,col3])

Longitudinal_Strain_Rate_Endo_seg002_point4 = as.numeric(Strain_Rate_data[22,col4])

Longitudinal_Strain_Rate_Endo_seg002_point5 = as.numeric(Strain_Rate_data[22,col5])

Longitudinal_Strain_Rate_Endo_seg002_a1 = (Longitudinal_Strain_Rate_Endo_seg002_point2 - Longitudinal_Strain_Rate_Endo_seg002_point1)/Time_duration1

Longitudinal_Strain_Rate_Endo_seg002_a2 = (Longitudinal_Strain_Rate_Endo_seg002_point3 - Longitudinal_Strain_Rate_Endo_seg002_point2)/Time_duration2

Longitudinal_Strain_Rate_Endo_seg002_a3 = (Longitudinal_Strain_Rate_Endo_seg002_point4 - Longitudinal_Strain_Rate_Endo_seg002_point3)/Time_duration3

Longitudinal_Strain_Rate_Endo_seg002_a4 = (Longitudinal_Strain_Rate_Endo_seg002_point5 - Longitudinal_Strain_Rate_Endo_seg002_point4)/Time_duration4

Longitudinal_Strain_Rate_Endo_seg002_a_min = min(c(Longitudinal_Strain_Rate_Endo_seg002_a1,Longitudinal_Strain_Rate_Endo_seg002_a2,Longitudinal_Strain_Rate_Endo_seg002_a3,Longitudinal_Strain_Rate_Endo_seg002_a4))

Longitudinal_Strain_Rate_Endo_seg002_a_max = max(c(Longitudinal_Strain_Rate_Endo_seg002_a1,Longitudinal_Strain_Rate_Endo_seg002_a2,Longitudinal_Strain_Rate_Endo_seg002_a3,Longitudinal_Strain_Rate_Endo_seg002_a4))

Longitudinal_Strain_Rate_Endo_seg003_point1 = as.numeric(Strain_Rate_data[23,col1])

Longitudinal_Strain_Rate_Endo_seg003_point2 = as.numeric(Strain_Rate_data[23,col2])

Longitudinal_Strain_Rate_Endo_seg003_point3 = as.numeric(Strain_Rate_data[23,col3])

Longitudinal_Strain_Rate_Endo_seg003_point4 = as.numeric(Strain_Rate_data[23,col4])

Longitudinal_Strain_Rate_Endo_seg003_point5 = as.numeric(Strain_Rate_data[23,col5])

Longitudinal_Strain_Rate_Endo_seg003_a1 = (Longitudinal_Strain_Rate_Endo_seg003_point2 - Longitudinal_Strain_Rate_Endo_seg003_point1)/Time_duration1

Longitudinal_Strain_Rate_Endo_seg003_a2 = (Longitudinal_Strain_Rate_Endo_seg003_point3 - Longitudinal_Strain_Rate_Endo_seg003_point2)/Time_duration2

Longitudinal_Strain_Rate_Endo_seg003_a3 = (Longitudinal_Strain_Rate_Endo_seg003_point4 - Longitudinal_Strain_Rate_Endo_seg003_point3)/Time_duration3

Longitudinal_Strain_Rate_Endo_seg003_a4 = (Longitudinal_Strain_Rate_Endo_seg003_point5 - Longitudinal_Strain_Rate_Endo_seg003_point4)/Time_duration4

Longitudinal_Strain_Rate_Endo_seg003_a_min = min(c(Longitudinal_Strain_Rate_Endo_seg003_a1,Longitudinal_Strain_Rate_Endo_seg003_a2,Longitudinal_Strain_Rate_Endo_seg003_a3,Longitudinal_Strain_Rate_Endo_seg003_a4))

Longitudinal_Strain_Rate_Endo_seg003_a_max = max(c(Longitudinal_Strain_Rate_Endo_seg003_a1,Longitudinal_Strain_Rate_Endo_seg003_a2,Longitudinal_Strain_Rate_Endo_seg003_a3,Longitudinal_Strain_Rate_Endo_seg003_a4))

Longitudinal_Strain_Rate_Endo_seg004_point1 = as.numeric(Strain_Rate_data[24,col1])

Longitudinal_Strain_Rate_Endo_seg004_point2 = as.numeric(Strain_Rate_data[24,col2])

Longitudinal_Strain_Rate_Endo_seg004_point3 = as.numeric(Strain_Rate_data[24,col3])

Longitudinal_Strain_Rate_Endo_seg004_point4 = as.numeric(Strain_Rate_data[24,col4])

Longitudinal_Strain_Rate_Endo_seg004_point5 = as.numeric(Strain_Rate_data[24,col5])

Longitudinal_Strain_Rate_Endo_seg004_a1 = (Longitudinal_Strain_Rate_Endo_seg004_point2 - Longitudinal_Strain_Rate_Endo_seg004_point1)/Time_duration1

Longitudinal_Strain_Rate_Endo_seg004_a2 = (Longitudinal_Strain_Rate_Endo_seg004_point3 - Longitudinal_Strain_Rate_Endo_seg004_point2)/Time_duration2

Longitudinal_Strain_Rate_Endo_seg004_a3 = (Longitudinal_Strain_Rate_Endo_seg004_point4 - Longitudinal_Strain_Rate_Endo_seg004_point3)/Time_duration3

Longitudinal_Strain_Rate_Endo_seg004_a4 = (Longitudinal_Strain_Rate_Endo_seg004_point5 - Longitudinal_Strain_Rate_Endo_seg004_point4)/Time_duration4

Longitudinal_Strain_Rate_Endo_seg004_a_min = min(c(Longitudinal_Strain_Rate_Endo_seg004_a1,Longitudinal_Strain_Rate_Endo_seg004_a2,Longitudinal_Strain_Rate_Endo_seg004_a3,Longitudinal_Strain_Rate_Endo_seg004_a4))

Longitudinal_Strain_Rate_Endo_seg004_a_max = max(c(Longitudinal_Strain_Rate_Endo_seg004_a1,Longitudinal_Strain_Rate_Endo_seg004_a2,Longitudinal_Strain_Rate_Endo_seg004_a3,Longitudinal_Strain_Rate_Endo_seg004_a4))

Longitudinal_Strain_Rate_Endo_seg005_point1 = as.numeric(Strain_Rate_data[25,col1])

Longitudinal_Strain_Rate_Endo_seg005_point2 = as.numeric(Strain_Rate_data[25,col2])

Longitudinal_Strain_Rate_Endo_seg005_point3 = as.numeric(Strain_Rate_data[25,col3])

Longitudinal_Strain_Rate_Endo_seg005_point4 = as.numeric(Strain_Rate_data[25,col4])

Longitudinal_Strain_Rate_Endo_seg005_point5 = as.numeric(Strain_Rate_data[25,col5])

Longitudinal_Strain_Rate_Endo_seg005_a1 = (Longitudinal_Strain_Rate_Endo_seg005_point2 - Longitudinal_Strain_Rate_Endo_seg005_point1)/Time_duration1

Longitudinal_Strain_Rate_Endo_seg005_a2 = (Longitudinal_Strain_Rate_Endo_seg005_point3 - Longitudinal_Strain_Rate_Endo_seg005_point2)/Time_duration2

Longitudinal_Strain_Rate_Endo_seg005_a3 = (Longitudinal_Strain_Rate_Endo_seg005_point4 - Longitudinal_Strain_Rate_Endo_seg005_point3)/Time_duration3

Longitudinal_Strain_Rate_Endo_seg005_a4 = (Longitudinal_Strain_Rate_Endo_seg005_point5 - Longitudinal_Strain_Rate_Endo_seg005_point4)/Time_duration4

Longitudinal_Strain_Rate_Endo_seg005_a_min = min(c(Longitudinal_Strain_Rate_Endo_seg005_a1,Longitudinal_Strain_Rate_Endo_seg005_a2,Longitudinal_Strain_Rate_Endo_seg005_a3,Longitudinal_Strain_Rate_Endo_seg005_a4))

Longitudinal_Strain_Rate_Endo_seg005_a_max = max(c(Longitudinal_Strain_Rate_Endo_seg005_a1,Longitudinal_Strain_Rate_Endo_seg005_a2,Longitudinal_Strain_Rate_Endo_seg005_a3,Longitudinal_Strain_Rate_Endo_seg005_a4))

Longitudinal_Strain_Rate_Endo_seg006_point1 = as.numeric(Strain_Rate_data[26,col1])

Longitudinal_Strain_Rate_Endo_seg006_point2 = as.numeric(Strain_Rate_data[26,col2])

Longitudinal_Strain_Rate_Endo_seg006_point3 = as.numeric(Strain_Rate_data[26,col3])

Longitudinal_Strain_Rate_Endo_seg006_point4 = as.numeric(Strain_Rate_data[26,col4])

Longitudinal_Strain_Rate_Endo_seg006_point5 = as.numeric(Strain_Rate_data[26,col5])

Longitudinal_Strain_Rate_Endo_seg006_a1 = (Longitudinal_Strain_Rate_Endo_seg006_point2 - Longitudinal_Strain_Rate_Endo_seg006_point1)/Time_duration1

Longitudinal_Strain_Rate_Endo_seg006_a2 = (Longitudinal_Strain_Rate_Endo_seg006_point3 - Longitudinal_Strain_Rate_Endo_seg006_point2)/Time_duration2

Longitudinal_Strain_Rate_Endo_seg006_a3 = (Longitudinal_Strain_Rate_Endo_seg006_point4 - Longitudinal_Strain_Rate_Endo_seg006_point3)/Time_duration3

Longitudinal_Strain_Rate_Endo_seg006_a4 = (Longitudinal_Strain_Rate_Endo_seg006_point5 - Longitudinal_Strain_Rate_Endo_seg006_point4)/Time_duration4

Longitudinal_Strain_Rate_Endo_seg006_a_min = min(c(Longitudinal_Strain_Rate_Endo_seg006_a1,Longitudinal_Strain_Rate_Endo_seg006_a2,Longitudinal_Strain_Rate_Endo_seg006_a3,Longitudinal_Strain_Rate_Endo_seg006_a4))

Longitudinal_Strain_Rate_Endo_seg006_a_max = max(c(Longitudinal_Strain_Rate_Endo_seg006_a1,Longitudinal_Strain_Rate_Endo_seg006_a2,Longitudinal_Strain_Rate_Endo_seg006_a3,Longitudinal_Strain_Rate_Endo_seg006_a4))

##Average_values_per_frame_Longitudinal_Strain_Rate_Endo

Average_values_per_frame_Longitudinal_Strain_Rate_Endo_point1 = as.numeric(Strain_Rate_data[30,col1])

Average_values_per_frame_Longitudinal_Strain_Rate_Endo_point2 = as.numeric(Strain_Rate_data[30,col2])

Average_values_per_frame_Longitudinal_Strain_Rate_Endo_point3 = as.numeric(Strain_Rate_data[30,col3])

Average_values_per_frame_Longitudinal_Strain_Rate_Endo_point4 = as.numeric(Strain_Rate_data[30,col4])

Average_values_per_frame_Longitudinal_Strain_Rate_Endo_point5 = as.numeric(Strain_Rate_data[30,col5])

Average_values_per_frame_Longitudinal_Strain_Rate_Endo_a1 = (Average_values_per_frame_Longitudinal_Strain_Rate_Endo_point2 - Average_values_per_frame_Longitudinal_Strain_Rate_Endo_point1)/Time_duration1

Average_values_per_frame_Longitudinal_Strain_Rate_Endo_a2 = (Average_values_per_frame_Longitudinal_Strain_Rate_Endo_point3 - Average_values_per_frame_Longitudinal_Strain_Rate_Endo_point2)/Time_duration2

Average_values_per_frame_Longitudinal_Strain_Rate_Endo_a3 = (Average_values_per_frame_Longitudinal_Strain_Rate_Endo_point4 - Average_values_per_frame_Longitudinal_Strain_Rate_Endo_point3)/Time_duration3

Average_values_per_frame_Longitudinal_Strain_Rate_Endo_a4 = (Average_values_per_frame_Longitudinal_Strain_Rate_Endo_point5 - Average_values_per_frame_Longitudinal_Strain_Rate_Endo_point4)/Time_duration4

Average_values_per_frame_Longitudinal_Strain_Rate_Endo_a_min = min(c(Average_values_per_frame_Longitudinal_Strain_Rate_Endo_a1,Average_values_per_frame_Longitudinal_Strain_Rate_Endo_a2,Average_values_per_frame_Longitudinal_Strain_Rate_Endo_a3,Average_values_per_frame_Longitudinal_Strain_Rate_Endo_a4))

Average_values_per_frame_Longitudinal_Strain_Rate_Endo_a_max = max(c(Average_values_per_frame_Longitudinal_Strain_Rate_Endo_a1,Average_values_per_frame_Longitudinal_Strain_Rate_Endo_a2,Average_values_per_frame_Longitudinal_Strain_Rate_Endo_a3,Average_values_per_frame_Longitudinal_Strain_Rate_Endo_a4))

##Radial_Strain_Rate_Epi

Radial_Strain_Rate_Epi_seg001_point1 = as.numeric(Strain_Rate_data[34,col1])

Radial_Strain_Rate_Epi_seg001_point2 = as.numeric(Strain_Rate_data[34,col2])

Radial_Strain_Rate_Epi_seg001_point3 = as.numeric(Strain_Rate_data[34,col3])

Radial_Strain_Rate_Epi_seg001_point4 = as.numeric(Strain_Rate_data[34,col4])

Radial_Strain_Rate_Epi_seg001_point5 = as.numeric(Strain_Rate_data[34,col5])

Radial_Strain_Rate_Epi_seg001_a1 = (Radial_Strain_Rate_Epi_seg001_point2 - Radial_Strain_Rate_Epi_seg001_point1)/Time_duration1

Radial_Strain_Rate_Epi_seg001_a2 = (Radial_Strain_Rate_Epi_seg001_point3 - Radial_Strain_Rate_Epi_seg001_point2)/Time_duration2

Radial_Strain_Rate_Epi_seg001_a3 = (Radial_Strain_Rate_Epi_seg001_point4 - Radial_Strain_Rate_Epi_seg001_point3)/Time_duration3

Radial_Strain_Rate_Epi_seg001_a4 = (Radial_Strain_Rate_Epi_seg001_point5 - Radial_Strain_Rate_Epi_seg001_point4)/Time_duration4

Radial_Strain_Rate_Epi_seg001_a_min = min(c(Radial_Strain_Rate_Epi_seg001_a1,Radial_Strain_Rate_Epi_seg001_a2,Radial_Strain_Rate_Epi_seg001_a3,Radial_Strain_Rate_Epi_seg001_a4))

Radial_Strain_Rate_Epi_seg001_a_max = max(c(Radial_Strain_Rate_Epi_seg001_a1,Radial_Strain_Rate_Epi_seg001_a2,Radial_Strain_Rate_Epi_seg001_a3,Radial_Strain_Rate_Epi_seg001_a4))

Radial_Strain_Rate_Epi_seg002_point1 = as.numeric(Strain_Rate_data[35,col1])

Radial_Strain_Rate_Epi_seg002_point2 = as.numeric(Strain_Rate_data[35,col2])

Radial_Strain_Rate_Epi_seg002_point3 = as.numeric(Strain_Rate_data[35,col3])

Radial_Strain_Rate_Epi_seg002_point4 = as.numeric(Strain_Rate_data[35,col4])

Radial_Strain_Rate_Epi_seg002_point5 = as.numeric(Strain_Rate_data[35,col5])

Radial_Strain_Rate_Epi_seg002_a1 = (Radial_Strain_Rate_Epi_seg002_point2 - Radial_Strain_Rate_Epi_seg002_point1)/Time_duration1

Radial_Strain_Rate_Epi_seg002_a2 = (Radial_Strain_Rate_Epi_seg002_point3 - Radial_Strain_Rate_Epi_seg002_point2)/Time_duration2

Radial_Strain_Rate_Epi_seg002_a3 = (Radial_Strain_Rate_Epi_seg002_point4 - Radial_Strain_Rate_Epi_seg002_point3)/Time_duration3

Radial_Strain_Rate_Epi_seg002_a4 = (Radial_Strain_Rate_Epi_seg002_point5 - Radial_Strain_Rate_Epi_seg002_point4)/Time_duration4

Radial_Strain_Rate_Epi_seg002_a_min = min(c(Radial_Strain_Rate_Epi_seg002_a1,Radial_Strain_Rate_Epi_seg002_a2,Radial_Strain_Rate_Epi_seg002_a3,Radial_Strain_Rate_Epi_seg002_a4))

Radial_Strain_Rate_Epi_seg002_a_max = max(c(Radial_Strain_Rate_Epi_seg002_a1,Radial_Strain_Rate_Epi_seg002_a2,Radial_Strain_Rate_Epi_seg002_a3,Radial_Strain_Rate_Epi_seg002_a4))

Radial_Strain_Rate_Epi_seg003_point1 = as.numeric(Strain_Rate_data[36,col1])

Radial_Strain_Rate_Epi_seg003_point2 = as.numeric(Strain_Rate_data[36,col2])

Radial_Strain_Rate_Epi_seg003_point3 = as.numeric(Strain_Rate_data[36,col3])

Radial_Strain_Rate_Epi_seg003_point4 = as.numeric(Strain_Rate_data[36,col4])

Radial_Strain_Rate_Epi_seg003_point5 = as.numeric(Strain_Rate_data[36,col5])

Radial_Strain_Rate_Epi_seg003_a1 = (Radial_Strain_Rate_Epi_seg003_point2 - Radial_Strain_Rate_Epi_seg003_point1)/Time_duration1

Radial_Strain_Rate_Epi_seg003_a2 = (Radial_Strain_Rate_Epi_seg003_point3 - Radial_Strain_Rate_Epi_seg003_point2)/Time_duration2

Radial_Strain_Rate_Epi_seg003_a3 = (Radial_Strain_Rate_Epi_seg003_point4 - Radial_Strain_Rate_Epi_seg003_point3)/Time_duration3

Radial_Strain_Rate_Epi_seg003_a4 = (Radial_Strain_Rate_Epi_seg003_point5 - Radial_Strain_Rate_Epi_seg003_point4)/Time_duration4

Radial_Strain_Rate_Epi_seg003_a_min = min(c(Radial_Strain_Rate_Epi_seg003_a1,Radial_Strain_Rate_Epi_seg003_a2,Radial_Strain_Rate_Epi_seg003_a3,Radial_Strain_Rate_Epi_seg003_a4))

Radial_Strain_Rate_Epi_seg003_a_max = max(c(Radial_Strain_Rate_Epi_seg003_a1,Radial_Strain_Rate_Epi_seg003_a2,Radial_Strain_Rate_Epi_seg003_a3,Radial_Strain_Rate_Epi_seg003_a4))

Radial_Strain_Rate_Epi_seg004_point1 = as.numeric(Strain_Rate_data[37,col1])

Radial_Strain_Rate_Epi_seg004_point2 = as.numeric(Strain_Rate_data[37,col2])

Radial_Strain_Rate_Epi_seg004_point3 = as.numeric(Strain_Rate_data[37,col3])

Radial_Strain_Rate_Epi_seg004_point4 = as.numeric(Strain_Rate_data[37,col4])

Radial_Strain_Rate_Epi_seg004_point5 = as.numeric(Strain_Rate_data[37,col5])

Radial_Strain_Rate_Epi_seg004_a1 = (Radial_Strain_Rate_Epi_seg004_point2 - Radial_Strain_Rate_Epi_seg004_point1)/Time_duration1

Radial_Strain_Rate_Epi_seg004_a2 = (Radial_Strain_Rate_Epi_seg004_point3 - Radial_Strain_Rate_Epi_seg004_point2)/Time_duration2

Radial_Strain_Rate_Epi_seg004_a3 = (Radial_Strain_Rate_Epi_seg004_point4 - Radial_Strain_Rate_Epi_seg004_point3)/Time_duration3

Radial_Strain_Rate_Epi_seg004_a4 = (Radial_Strain_Rate_Epi_seg004_point5 - Radial_Strain_Rate_Epi_seg004_point4)/Time_duration4

Radial_Strain_Rate_Epi_seg004_a_min = min(c(Radial_Strain_Rate_Epi_seg004_a1,Radial_Strain_Rate_Epi_seg004_a2,Radial_Strain_Rate_Epi_seg004_a3,Radial_Strain_Rate_Epi_seg004_a4))

Radial_Strain_Rate_Epi_seg004_a_max = max(c(Radial_Strain_Rate_Epi_seg004_a1,Radial_Strain_Rate_Epi_seg004_a2,Radial_Strain_Rate_Epi_seg004_a3,Radial_Strain_Rate_Epi_seg004_a4))

Radial_Strain_Rate_Epi_seg005_point1 = as.numeric(Strain_Rate_data[38,col1])

Radial_Strain_Rate_Epi_seg005_point2 = as.numeric(Strain_Rate_data[38,col2])

Radial_Strain_Rate_Epi_seg005_point3 = as.numeric(Strain_Rate_data[38,col3])

Radial_Strain_Rate_Epi_seg005_point4 = as.numeric(Strain_Rate_data[38,col4])

Radial_Strain_Rate_Epi_seg005_point5 = as.numeric(Strain_Rate_data[38,col5])

Radial_Strain_Rate_Epi_seg005_a1 = (Radial_Strain_Rate_Epi_seg005_point2 - Radial_Strain_Rate_Epi_seg005_point1)/Time_duration1

Radial_Strain_Rate_Epi_seg005_a2 = (Radial_Strain_Rate_Epi_seg005_point3 - Radial_Strain_Rate_Epi_seg005_point2)/Time_duration2

Radial_Strain_Rate_Epi_seg005_a3 = (Radial_Strain_Rate_Epi_seg005_point4 - Radial_Strain_Rate_Epi_seg005_point3)/Time_duration3

Radial_Strain_Rate_Epi_seg005_a4 = (Radial_Strain_Rate_Epi_seg005_point5 - Radial_Strain_Rate_Epi_seg005_point4)/Time_duration4

Radial_Strain_Rate_Epi_seg005_a_min = min(c(Radial_Strain_Rate_Epi_seg005_a1,Radial_Strain_Rate_Epi_seg005_a2,Radial_Strain_Rate_Epi_seg005_a3,Radial_Strain_Rate_Epi_seg005_a4))

Radial_Strain_Rate_Epi_seg005_a_max = max(c(Radial_Strain_Rate_Epi_seg005_a1,Radial_Strain_Rate_Epi_seg005_a2,Radial_Strain_Rate_Epi_seg005_a3,Radial_Strain_Rate_Epi_seg005_a4))

Radial_Strain_Rate_Epi_seg006_point1 = as.numeric(Strain_Rate_data[39,col1])

Radial_Strain_Rate_Epi_seg006_point2 = as.numeric(Strain_Rate_data[39,col2])

Radial_Strain_Rate_Epi_seg006_point3 = as.numeric(Strain_Rate_data[39,col3])

Radial_Strain_Rate_Epi_seg006_point4 = as.numeric(Strain_Rate_data[39,col4])

Radial_Strain_Rate_Epi_seg006_point5 = as.numeric(Strain_Rate_data[39,col5])

Radial_Strain_Rate_Epi_seg006_a1 = (Radial_Strain_Rate_Epi_seg006_point2 - Radial_Strain_Rate_Epi_seg006_point1)/Time_duration1

Radial_Strain_Rate_Epi_seg006_a2 = (Radial_Strain_Rate_Epi_seg006_point3 - Radial_Strain_Rate_Epi_seg006_point2)/Time_duration2

Radial_Strain_Rate_Epi_seg006_a3 = (Radial_Strain_Rate_Epi_seg006_point4 - Radial_Strain_Rate_Epi_seg006_point3)/Time_duration3

Radial_Strain_Rate_Epi_seg006_a4 = (Radial_Strain_Rate_Epi_seg006_point5 - Radial_Strain_Rate_Epi_seg006_point4)/Time_duration4

Radial_Strain_Rate_Epi_seg006_a_min = min(c(Radial_Strain_Rate_Epi_seg006_a1,Radial_Strain_Rate_Epi_seg006_a2,Radial_Strain_Rate_Epi_seg006_a3,Radial_Strain_Rate_Epi_seg006_a4))

Radial_Strain_Rate_Epi_seg006_a_max = max(c(Radial_Strain_Rate_Epi_seg006_a1,Radial_Strain_Rate_Epi_seg006_a2,Radial_Strain_Rate_Epi_seg006_a3,Radial_Strain_Rate_Epi_seg006_a4))

##Average_values_per_frame_Radial_Strain_Rate_Epi

Average_values_per_frame_Radial_Strain_Rate_Epi_point1 = as.numeric(Strain_Rate_data[43,col1])

Average_values_per_frame_Radial_Strain_Rate_Epi_point2 = as.numeric(Strain_Rate_data[43,col2])

Average_values_per_frame_Radial_Strain_Rate_Epi_point3 = as.numeric(Strain_Rate_data[43,col3])

Average_values_per_frame_Radial_Strain_Rate_Epi_point4 = as.numeric(Strain_Rate_data[43,col4])

Average_values_per_frame_Radial_Strain_Rate_Epi_point5 = as.numeric(Strain_Rate_data[43,col5])

Average_values_per_frame_Radial_Strain_Rate_Epi_a1 = (Average_values_per_frame_Radial_Strain_Rate_Epi_point2 - Average_values_per_frame_Radial_Strain_Rate_Epi_point1)/Time_duration1

Average_values_per_frame_Radial_Strain_Rate_Epi_a2 = (Average_values_per_frame_Radial_Strain_Rate_Epi_point3 - Average_values_per_frame_Radial_Strain_Rate_Epi_point2)/Time_duration2

Average_values_per_frame_Radial_Strain_Rate_Epi_a3 = (Average_values_per_frame_Radial_Strain_Rate_Epi_point4 - Average_values_per_frame_Radial_Strain_Rate_Epi_point3)/Time_duration3

Average_values_per_frame_Radial_Strain_Rate_Epi_a4 = (Average_values_per_frame_Radial_Strain_Rate_Epi_point5 - Average_values_per_frame_Radial_Strain_Rate_Epi_point4)/Time_duration4

Average_values_per_frame_Radial_Strain_Rate_Epi_a_min = min(c(Average_values_per_frame_Radial_Strain_Rate_Epi_a1,Average_values_per_frame_Radial_Strain_Rate_Epi_a2,Average_values_per_frame_Radial_Strain_Rate_Epi_a3,Average_values_per_frame_Radial_Strain_Rate_Epi_a4))

Average_values_per_frame_Radial_Strain_Rate_Epi_a_max = max(c(Average_values_per_frame_Radial_Strain_Rate_Epi_a1,Average_values_per_frame_Radial_Strain_Rate_Epi_a2,Average_values_per_frame_Radial_Strain_Rate_Epi_a3,Average_values_per_frame_Radial_Strain_Rate_Epi_a4))

##Longitudinal_Strain_Rate_Epi

Longitudinal_Strain_Rate_Epi_seg001_point1 = as.numeric(Strain_Rate_data[47,col1])

Longitudinal_Strain_Rate_Epi_seg001_point2 = as.numeric(Strain_Rate_data[47,col2])

Longitudinal_Strain_Rate_Epi_seg001_point3 = as.numeric(Strain_Rate_data[47,col3])

Longitudinal_Strain_Rate_Epi_seg001_point4 = as.numeric(Strain_Rate_data[47,col4])

Longitudinal_Strain_Rate_Epi_seg001_point5 = as.numeric(Strain_Rate_data[47,col5])

Longitudinal_Strain_Rate_Epi_seg001_a1 = (Longitudinal_Strain_Rate_Epi_seg001_point2 - Longitudinal_Strain_Rate_Epi_seg001_point1)/Time_duration1

Longitudinal_Strain_Rate_Epi_seg001_a2 = (Longitudinal_Strain_Rate_Epi_seg001_point3 - Longitudinal_Strain_Rate_Epi_seg001_point2)/Time_duration2

Longitudinal_Strain_Rate_Epi_seg001_a3 = (Longitudinal_Strain_Rate_Epi_seg001_point4 - Longitudinal_Strain_Rate_Epi_seg001_point3)/Time_duration3

Longitudinal_Strain_Rate_Epi_seg001_a4 = (Longitudinal_Strain_Rate_Epi_seg001_point5 - Longitudinal_Strain_Rate_Epi_seg001_point4)/Time_duration4

Longitudinal_Strain_Rate_Epi_seg001_a_min = min(c(Longitudinal_Strain_Rate_Epi_seg001_a1,Longitudinal_Strain_Rate_Epi_seg001_a2,Longitudinal_Strain_Rate_Epi_seg001_a3,Longitudinal_Strain_Rate_Epi_seg001_a4))

Longitudinal_Strain_Rate_Epi_seg001_a_max = max(c(Longitudinal_Strain_Rate_Epi_seg001_a1,Longitudinal_Strain_Rate_Epi_seg001_a2,Longitudinal_Strain_Rate_Epi_seg001_a3,Longitudinal_Strain_Rate_Epi_seg001_a4))

Longitudinal_Strain_Rate_Epi_seg002_point1 = as.numeric(Strain_Rate_data[48,col1])

Longitudinal_Strain_Rate_Epi_seg002_point2 = as.numeric(Strain_Rate_data[48,col2])

Longitudinal_Strain_Rate_Epi_seg002_point3 = as.numeric(Strain_Rate_data[48,col3])

Longitudinal_Strain_Rate_Epi_seg002_point4 = as.numeric(Strain_Rate_data[48,col4])

Longitudinal_Strain_Rate_Epi_seg002_point5 = as.numeric(Strain_Rate_data[48,col5])

Longitudinal_Strain_Rate_Epi_seg002_a1 = (Longitudinal_Strain_Rate_Epi_seg002_point2 - Longitudinal_Strain_Rate_Epi_seg002_point1)/Time_duration1

Longitudinal_Strain_Rate_Epi_seg002_a2 = (Longitudinal_Strain_Rate_Epi_seg002_point3 - Longitudinal_Strain_Rate_Epi_seg002_point2)/Time_duration2

Longitudinal_Strain_Rate_Epi_seg002_a3 = (Longitudinal_Strain_Rate_Epi_seg002_point4 - Longitudinal_Strain_Rate_Epi_seg002_point3)/Time_duration3

Longitudinal_Strain_Rate_Epi_seg002_a4 = (Longitudinal_Strain_Rate_Epi_seg002_point5 - Longitudinal_Strain_Rate_Epi_seg002_point4)/Time_duration4

Longitudinal_Strain_Rate_Epi_seg002_a_min = min(c(Longitudinal_Strain_Rate_Epi_seg002_a1,Longitudinal_Strain_Rate_Epi_seg002_a2,Longitudinal_Strain_Rate_Epi_seg002_a3,Longitudinal_Strain_Rate_Epi_seg002_a4))

Longitudinal_Strain_Rate_Epi_seg002_a_max = max(c(Longitudinal_Strain_Rate_Epi_seg002_a1,Longitudinal_Strain_Rate_Epi_seg002_a2,Longitudinal_Strain_Rate_Epi_seg002_a3,Longitudinal_Strain_Rate_Epi_seg002_a4))

Longitudinal_Strain_Rate_Epi_seg003_point1 = as.numeric(Strain_Rate_data[49,col1])

Longitudinal_Strain_Rate_Epi_seg003_point2 = as.numeric(Strain_Rate_data[49,col2])

Longitudinal_Strain_Rate_Epi_seg003_point3 = as.numeric(Strain_Rate_data[49,col3])

Longitudinal_Strain_Rate_Epi_seg003_point4 = as.numeric(Strain_Rate_data[49,col4])

Longitudinal_Strain_Rate_Epi_seg003_point5 = as.numeric(Strain_Rate_data[49,col5])

Longitudinal_Strain_Rate_Epi_seg003_a1 = (Longitudinal_Strain_Rate_Epi_seg003_point2 - Longitudinal_Strain_Rate_Epi_seg003_point1)/Time_duration1

Longitudinal_Strain_Rate_Epi_seg003_a2 = (Longitudinal_Strain_Rate_Epi_seg003_point3 - Longitudinal_Strain_Rate_Epi_seg003_point2)/Time_duration2

Longitudinal_Strain_Rate_Epi_seg003_a3 = (Longitudinal_Strain_Rate_Epi_seg003_point4 - Longitudinal_Strain_Rate_Epi_seg003_point3)/Time_duration3

Longitudinal_Strain_Rate_Epi_seg003_a4 = (Longitudinal_Strain_Rate_Epi_seg003_point5 - Longitudinal_Strain_Rate_Epi_seg003_point4)/Time_duration4

Longitudinal_Strain_Rate_Epi_seg003_a_min = min(c(Longitudinal_Strain_Rate_Epi_seg003_a1,Longitudinal_Strain_Rate_Epi_seg003_a2,Longitudinal_Strain_Rate_Epi_seg003_a3,Longitudinal_Strain_Rate_Epi_seg003_a4))

Longitudinal_Strain_Rate_Epi_seg003_a_max = max(c(Longitudinal_Strain_Rate_Epi_seg003_a1,Longitudinal_Strain_Rate_Epi_seg003_a2,Longitudinal_Strain_Rate_Epi_seg003_a3,Longitudinal_Strain_Rate_Epi_seg003_a4))

Longitudinal_Strain_Rate_Epi_seg004_point1 = as.numeric(Strain_Rate_data[50,col1])

Longitudinal_Strain_Rate_Epi_seg004_point2 = as.numeric(Strain_Rate_data[50,col2])

Longitudinal_Strain_Rate_Epi_seg004_point3 = as.numeric(Strain_Rate_data[50,col3])

Longitudinal_Strain_Rate_Epi_seg004_point4 = as.numeric(Strain_Rate_data[50,col4])

Longitudinal_Strain_Rate_Epi_seg004_point5 = as.numeric(Strain_Rate_data[50,col5])

Longitudinal_Strain_Rate_Epi_seg004_a1 = (Longitudinal_Strain_Rate_Epi_seg004_point2 - Longitudinal_Strain_Rate_Epi_seg004_point1)/Time_duration1

Longitudinal_Strain_Rate_Epi_seg004_a2 = (Longitudinal_Strain_Rate_Epi_seg004_point3 - Longitudinal_Strain_Rate_Epi_seg004_point2)/Time_duration2

Longitudinal_Strain_Rate_Epi_seg004_a3 = (Longitudinal_Strain_Rate_Epi_seg004_point4 - Longitudinal_Strain_Rate_Epi_seg004_point3)/Time_duration3

Longitudinal_Strain_Rate_Epi_seg004_a4 = (Longitudinal_Strain_Rate_Epi_seg004_point5 - Longitudinal_Strain_Rate_Epi_seg004_point4)/Time_duration4

Longitudinal_Strain_Rate_Epi_seg004_a_min = min(c(Longitudinal_Strain_Rate_Epi_seg004_a1,Longitudinal_Strain_Rate_Epi_seg004_a2,Longitudinal_Strain_Rate_Epi_seg004_a3,Longitudinal_Strain_Rate_Epi_seg004_a4))

Longitudinal_Strain_Rate_Epi_seg004_a_max = max(c(Longitudinal_Strain_Rate_Epi_seg004_a1,Longitudinal_Strain_Rate_Epi_seg004_a2,Longitudinal_Strain_Rate_Epi_seg004_a3,Longitudinal_Strain_Rate_Epi_seg004_a4))

Longitudinal_Strain_Rate_Epi_seg005_point1 = as.numeric(Strain_Rate_data[51,col1])

Longitudinal_Strain_Rate_Epi_seg005_point2 = as.numeric(Strain_Rate_data[51,col2])

Longitudinal_Strain_Rate_Epi_seg005_point3 = as.numeric(Strain_Rate_data[51,col3])

Longitudinal_Strain_Rate_Epi_seg005_point4 = as.numeric(Strain_Rate_data[51,col4])

Longitudinal_Strain_Rate_Epi_seg005_point5 = as.numeric(Strain_Rate_data[51,col5])

Longitudinal_Strain_Rate_Epi_seg005_a1 = (Longitudinal_Strain_Rate_Epi_seg005_point2 - Longitudinal_Strain_Rate_Epi_seg005_point1)/Time_duration1

Longitudinal_Strain_Rate_Epi_seg005_a2 = (Longitudinal_Strain_Rate_Epi_seg005_point3 - Longitudinal_Strain_Rate_Epi_seg005_point2)/Time_duration2

Longitudinal_Strain_Rate_Epi_seg005_a3 = (Longitudinal_Strain_Rate_Epi_seg005_point4 - Longitudinal_Strain_Rate_Epi_seg005_point3)/Time_duration3

Longitudinal_Strain_Rate_Epi_seg005_a4 = (Longitudinal_Strain_Rate_Epi_seg005_point5 - Longitudinal_Strain_Rate_Epi_seg005_point4)/Time_duration4

Longitudinal_Strain_Rate_Epi_seg005_a_min = min(c(Longitudinal_Strain_Rate_Epi_seg005_a1,Longitudinal_Strain_Rate_Epi_seg005_a2,Longitudinal_Strain_Rate_Epi_seg005_a3,Longitudinal_Strain_Rate_Epi_seg005_a4))

Longitudinal_Strain_Rate_Epi_seg005_a_max = max(c(Longitudinal_Strain_Rate_Epi_seg005_a1,Longitudinal_Strain_Rate_Epi_seg005_a2,Longitudinal_Strain_Rate_Epi_seg005_a3,Longitudinal_Strain_Rate_Epi_seg005_a4))

Longitudinal_Strain_Rate_Epi_seg006_point1 = as.numeric(Strain_Rate_data[52,col1])

Longitudinal_Strain_Rate_Epi_seg006_point2 = as.numeric(Strain_Rate_data[52,col2])

Longitudinal_Strain_Rate_Epi_seg006_point3 = as.numeric(Strain_Rate_data[52,col3])

Longitudinal_Strain_Rate_Epi_seg006_point4 = as.numeric(Strain_Rate_data[52,col4])

Longitudinal_Strain_Rate_Epi_seg006_point5 = as.numeric(Strain_Rate_data[52,col5])

Longitudinal_Strain_Rate_Epi_seg006_a1 = (Longitudinal_Strain_Rate_Epi_seg006_point2 - Longitudinal_Strain_Rate_Epi_seg006_point1)/Time_duration1

Longitudinal_Strain_Rate_Epi_seg006_a2 = (Longitudinal_Strain_Rate_Epi_seg006_point3 - Longitudinal_Strain_Rate_Epi_seg006_point2)/Time_duration2

Longitudinal_Strain_Rate_Epi_seg006_a3 = (Longitudinal_Strain_Rate_Epi_seg006_point4 - Longitudinal_Strain_Rate_Epi_seg006_point3)/Time_duration3

Longitudinal_Strain_Rate_Epi_seg006_a4 = (Longitudinal_Strain_Rate_Epi_seg006_point5 - Longitudinal_Strain_Rate_Epi_seg006_point4)/Time_duration4

Longitudinal_Strain_Rate_Epi_seg006_a_min = min(c(Longitudinal_Strain_Rate_Epi_seg006_a1,Longitudinal_Strain_Rate_Epi_seg006_a2,Longitudinal_Strain_Rate_Epi_seg006_a3,Longitudinal_Strain_Rate_Epi_seg006_a4))

Longitudinal_Strain_Rate_Epi_seg006_a_max = max(c(Longitudinal_Strain_Rate_Epi_seg006_a1,Longitudinal_Strain_Rate_Epi_seg006_a2,Longitudinal_Strain_Rate_Epi_seg006_a3,Longitudinal_Strain_Rate_Epi_seg006_a4))

##Average_values_per_frame_Longitudinal_Strain_Rate_Epi

Average_values_per_frame_Longitudinal_Strain_Rate_Epi_point1 = as.numeric(Strain_Rate_data[56,col1])

Average_values_per_frame_Longitudinal_Strain_Rate_Epi_point2 = as.numeric(Strain_Rate_data[56,col2])

Average_values_per_frame_Longitudinal_Strain_Rate_Epi_point3 = as.numeric(Strain_Rate_data[56,col3])

Average_values_per_frame_Longitudinal_Strain_Rate_Epi_point4 = as.numeric(Strain_Rate_data[56,col4])

Average_values_per_frame_Longitudinal_Strain_Rate_Epi_point5 = as.numeric(Strain_Rate_data[56,col5])

Average_values_per_frame_Longitudinal_Strain_Rate_Epi_a1 = (Average_values_per_frame_Longitudinal_Strain_Rate_Epi_point2 - Average_values_per_frame_Longitudinal_Strain_Rate_Epi_point1)/Time_duration1

Average_values_per_frame_Longitudinal_Strain_Rate_Epi_a2 = (Average_values_per_frame_Longitudinal_Strain_Rate_Epi_point3 - Average_values_per_frame_Longitudinal_Strain_Rate_Epi_point2)/Time_duration2

Average_values_per_frame_Longitudinal_Strain_Rate_Epi_a3 = (Average_values_per_frame_Longitudinal_Strain_Rate_Epi_point4 - Average_values_per_frame_Longitudinal_Strain_Rate_Epi_point3)/Time_duration3

Average_values_per_frame_Longitudinal_Strain_Rate_Epi_a4 = (Average_values_per_frame_Longitudinal_Strain_Rate_Epi_point5 - Average_values_per_frame_Longitudinal_Strain_Rate_Epi_point4)/Time_duration4

Average_values_per_frame_Longitudinal_Strain_Rate_Epi_a_min = min(c(Average_values_per_frame_Longitudinal_Strain_Rate_Epi_a1,Average_values_per_frame_Longitudinal_Strain_Rate_Epi_a2,Average_values_per_frame_Longitudinal_Strain_Rate_Epi_a3,Average_values_per_frame_Longitudinal_Strain_Rate_Epi_a4))

Average_values_per_frame_Longitudinal_Strain_Rate_Epi_a_max = max(c(Average_values_per_frame_Longitudinal_Strain_Rate_Epi_a1,Average_values_per_frame_Longitudinal_Strain_Rate_Epi_a2,Average_values_per_frame_Longitudinal_Strain_Rate_Epi_a3,Average_values_per_frame_Longitudinal_Strain_Rate_Epi_a4))

#Shear

Shear_data <- read.xlsx(i,sheetIndex = 6, header = F)

colnames(Shear_data) <- Shear_data[3,]

name1 = paste("fr0",time3,sep = "")

col5 = grep(name1,colnames(Shear_data))

col4 = col5-1

col3 = col5-2

col2 = col5-3

col1 = col5-4

Timepoint1 = as.numeric(Shear_data[4,col1])

Timepoint2 = as.numeric(Shear_data[4,col2])

Timepoint3 = as.numeric(Shear_data[4,col3])

Timepoint4 = as.numeric(Shear_data[4,col4])

Timepoint5 = as.numeric(Shear_data[4,col5])

Time_duration1 = Timepoint2 - Timepoint1

Time_duration2 = Timepoint3 - Timepoint2

Time_duration3 = Timepoint4 - Timepoint3

Time_duration4 = Timepoint5 - Timepoint4

##Shear

Shear_seg001_point1 = as.numeric(Shear_data[8,col1])

Shear_seg001_point2 = as.numeric(Shear_data[8,col2])

Shear_seg001_point3 = as.numeric(Shear_data[8,col3])

Shear_seg001_point4 = as.numeric(Shear_data[8,col4])

Shear_seg001_point5 = as.numeric(Shear_data[8,col5])

Shear_seg001_a1 = (Shear_seg001_point2 - Shear_seg001_point1)/Time_duration1

Shear_seg001_a2 = (Shear_seg001_point3 - Shear_seg001_point2)/Time_duration2

Shear_seg001_a3 = (Shear_seg001_point4 - Shear_seg001_point3)/Time_duration3

Shear_seg001_a4 = (Shear_seg001_point5 - Shear_seg001_point4)/Time_duration4

Shear_seg001_a_min = min(c(Shear_seg001_a1,Shear_seg001_a2,Shear_seg001_a3,Shear_seg001_a4))

Shear_seg001_a_max = max(c(Shear_seg001_a1,Shear_seg001_a2,Shear_seg001_a3,Shear_seg001_a4))

Shear_seg002_point1 = as.numeric(Shear_data[9,col1])

Shear_seg002_point2 = as.numeric(Shear_data[9,col2])

Shear_seg002_point3 = as.numeric(Shear_data[9,col3])

Shear_seg002_point4 = as.numeric(Shear_data[9,col4])

Shear_seg002_point5 = as.numeric(Shear_data[9,col5])

Shear_seg002_a1 = (Shear_seg002_point2 - Shear_seg002_point1)/Time_duration1

Shear_seg002_a2 = (Shear_seg002_point3 - Shear_seg002_point2)/Time_duration2

Shear_seg002_a3 = (Shear_seg002_point4 - Shear_seg002_point3)/Time_duration3

Shear_seg002_a4 = (Shear_seg002_point5 - Shear_seg002_point4)/Time_duration4

Shear_seg002_a_min = min(c(Shear_seg002_a1,Shear_seg002_a2,Shear_seg002_a3,Shear_seg002_a4))

Shear_seg002_a_max = max(c(Shear_seg002_a1,Shear_seg002_a2,Shear_seg002_a3,Shear_seg002_a4))

Shear_seg003_point1 = as.numeric(Shear_data[10,col1])

Shear_seg003_point2 = as.numeric(Shear_data[10,col2])

Shear_seg003_point3 = as.numeric(Shear_data[10,col3])

Shear_seg003_point4 = as.numeric(Shear_data[10,col4])

Shear_seg003_point5 = as.numeric(Shear_data[10,col5])

Shear_seg003_a1 = (Shear_seg003_point2 - Shear_seg003_point1)/Time_duration1

Shear_seg003_a2 = (Shear_seg003_point3 - Shear_seg003_point2)/Time_duration2

Shear_seg003_a3 = (Shear_seg003_point4 - Shear_seg003_point3)/Time_duration3

Shear_seg003_a4 = (Shear_seg003_point5 - Shear_seg003_point4)/Time_duration4

Shear_seg003_a_min = min(c(Shear_seg003_a1,Shear_seg003_a2,Shear_seg003_a3,Shear_seg003_a4))

Shear_seg003_a_max = max(c(Shear_seg003_a1,Shear_seg003_a2,Shear_seg003_a3,Shear_seg003_a4))

Shear_seg004_point1 = as.numeric(Shear_data[11,col1])

Shear_seg004_point2 = as.numeric(Shear_data[11,col2])

Shear_seg004_point3 = as.numeric(Shear_data[11,col3])

Shear_seg004_point4 = as.numeric(Shear_data[11,col4])

Shear_seg004_point5 = as.numeric(Shear_data[11,col5])

Shear_seg004_a1 = (Shear_seg004_point2 - Shear_seg004_point1)/Time_duration1

Shear_seg004_a2 = (Shear_seg004_point3 - Shear_seg004_point2)/Time_duration2

Shear_seg004_a3 = (Shear_seg004_point4 - Shear_seg004_point3)/Time_duration3

Shear_seg004_a4 = (Shear_seg004_point5 - Shear_seg004_point4)/Time_duration4

Shear_seg004_a_min = min(c(Shear_seg004_a1,Shear_seg004_a2,Shear_seg004_a3,Shear_seg004_a4))

Shear_seg004_a_max = max(c(Shear_seg004_a1,Shear_seg004_a2,Shear_seg004_a3,Shear_seg004_a4))

Shear_seg005_point1 = as.numeric(Shear_data[12,col1])

Shear_seg005_point2 = as.numeric(Shear_data[12,col2])

Shear_seg005_point3 = as.numeric(Shear_data[12,col3])

Shear_seg005_point4 = as.numeric(Shear_data[12,col4])

Shear_seg005_point5 = as.numeric(Shear_data[12,col5])

Shear_seg005_a1 = (Shear_seg005_point2 - Shear_seg005_point1)/Time_duration1

Shear_seg005_a2 = (Shear_seg005_point3 - Shear_seg005_point2)/Time_duration2

Shear_seg005_a3 = (Shear_seg005_point4 - Shear_seg005_point3)/Time_duration3

Shear_seg005_a4 = (Shear_seg005_point5 - Shear_seg005_point4)/Time_duration4

Shear_seg005_a_min = min(c(Shear_seg005_a1,Shear_seg005_a2,Shear_seg005_a3,Shear_seg005_a4))

Shear_seg005_a_max = max(c(Shear_seg005_a1,Shear_seg005_a2,Shear_seg005_a3,Shear_seg005_a4))

Shear_seg006_point1 = as.numeric(Shear_data[13,col1])

Shear_seg006_point2 = as.numeric(Shear_data[13,col2])

Shear_seg006_point3 = as.numeric(Shear_data[13,col3])

Shear_seg006_point4 = as.numeric(Shear_data[13,col4])

Shear_seg006_point5 = as.numeric(Shear_data[13,col5])

Shear_seg006_a1 = (Shear_seg006_point2 - Shear_seg006_point1)/Time_duration1

Shear_seg006_a2 = (Shear_seg006_point3 - Shear_seg006_point2)/Time_duration2

Shear_seg006_a3 = (Shear_seg006_point4 - Shear_seg006_point3)/Time_duration3

Shear_seg006_a4 = (Shear_seg006_point5 - Shear_seg006_point4)/Time_duration4

Shear_seg006_a_min = min(c(Shear_seg006_a1,Shear_seg006_a2,Shear_seg006_a3,Shear_seg006_a4))

Shear_seg006_a_max = max(c(Shear_seg006_a1,Shear_seg006_a2,Shear_seg006_a3,Shear_seg006_a4))

##Average_values_per_frame_Shear

Average_values_per_frame_Shear_point1 = as.numeric(Shear_data[17,col1])

Average_values_per_frame_Shear_point2 = as.numeric(Shear_data[17,col2])

Average_values_per_frame_Shear_point3 = as.numeric(Shear_data[17,col3])

Average_values_per_frame_Shear_point4 = as.numeric(Shear_data[17,col4])

Average_values_per_frame_Shear_point5 = as.numeric(Shear_data[17,col5])

Average_values_per_frame_Shear_a1 = (Average_values_per_frame_Shear_point2 - Average_values_per_frame_Shear_point1)/Time_duration1

Average_values_per_frame_Shear_a2 = (Average_values_per_frame_Shear_point3 - Average_values_per_frame_Shear_point2)/Time_duration2

Average_values_per_frame_Shear_a3 = (Average_values_per_frame_Shear_point4 - Average_values_per_frame_Shear_point3)/Time_duration3

Average_values_per_frame_Shear_a4 = (Average_values_per_frame_Shear_point5 - Average_values_per_frame_Shear_point4)/Time_duration4

Average_values_per_frame_Shear_a_min = min(c(Average_values_per_frame_Shear_a1,Average_values_per_frame_Shear_a2,Average_values_per_frame_Shear_a3,Average_values_per_frame_Shear_a4))

Average_values_per_frame_Shear_a_max = max(c(Average_values_per_frame_Shear_a1,Average_values_per_frame_Shear_a2,Average_values_per_frame_Shear_a3,Average_values_per_frame_Shear_a4))

##Shear_Rate

Shear_Rate_seg001_point1 = as.numeric(Shear_data[21,col1])

Shear_Rate_seg001_point2 = as.numeric(Shear_data[21,col2])

Shear_Rate_seg001_point3 = as.numeric(Shear_data[21,col3])

Shear_Rate_seg001_point4 = as.numeric(Shear_data[21,col4])

Shear_Rate_seg001_point5 = as.numeric(Shear_data[21,col5])

Shear_Rate_seg001_a1 = (Shear_Rate_seg001_point2 - Shear_Rate_seg001_point1)/Time_duration1

Shear_Rate_seg001_a2 = (Shear_Rate_seg001_point3 - Shear_Rate_seg001_point2)/Time_duration2

Shear_Rate_seg001_a3 = (Shear_Rate_seg001_point4 - Shear_Rate_seg001_point3)/Time_duration3

Shear_Rate_seg001_a4 = (Shear_Rate_seg001_point5 - Shear_Rate_seg001_point4)/Time_duration4

Shear_Rate_seg001_a_min = min(c(Shear_Rate_seg001_a1,Shear_Rate_seg001_a2,Shear_Rate_seg001_a3,Shear_Rate_seg001_a4))

Shear_Rate_seg001_a_max = max(c(Shear_Rate_seg001_a1,Shear_Rate_seg001_a2,Shear_Rate_seg001_a3,Shear_Rate_seg001_a4))

Shear_Rate_seg002_point1 = as.numeric(Shear_data[22,col1])

Shear_Rate_seg002_point2 = as.numeric(Shear_data[22,col2])

Shear_Rate_seg002_point3 = as.numeric(Shear_data[22,col3])

Shear_Rate_seg002_point4 = as.numeric(Shear_data[22,col4])

Shear_Rate_seg002_point5 = as.numeric(Shear_data[22,col5])

Shear_Rate_seg002_a1 = (Shear_Rate_seg002_point2 - Shear_Rate_seg002_point1)/Time_duration1

Shear_Rate_seg002_a2 = (Shear_Rate_seg002_point3 - Shear_Rate_seg002_point2)/Time_duration2

Shear_Rate_seg002_a3 = (Shear_Rate_seg002_point4 - Shear_Rate_seg002_point3)/Time_duration3

Shear_Rate_seg002_a4 = (Shear_Rate_seg002_point5 - Shear_Rate_seg002_point4)/Time_duration4

Shear_Rate_seg002_a_min = min(c(Shear_Rate_seg002_a1,Shear_Rate_seg002_a2,Shear_Rate_seg002_a3,Shear_Rate_seg002_a4))

Shear_Rate_seg002_a_max = max(c(Shear_Rate_seg002_a1,Shear_Rate_seg002_a2,Shear_Rate_seg002_a3,Shear_Rate_seg002_a4))

Shear_Rate_seg003_point1 = as.numeric(Shear_data[23,col1])

Shear_Rate_seg003_point2 = as.numeric(Shear_data[23,col2])

Shear_Rate_seg003_point3 = as.numeric(Shear_data[23,col3])

Shear_Rate_seg003_point4 = as.numeric(Shear_data[23,col4])

Shear_Rate_seg003_point5 = as.numeric(Shear_data[23,col5])

Shear_Rate_seg003_a1 = (Shear_Rate_seg003_point2 - Shear_Rate_seg003_point1)/Time_duration1

Shear_Rate_seg003_a2 = (Shear_Rate_seg003_point3 - Shear_Rate_seg003_point2)/Time_duration2

Shear_Rate_seg003_a3 = (Shear_Rate_seg003_point4 - Shear_Rate_seg003_point3)/Time_duration3

Shear_Rate_seg003_a4 = (Shear_Rate_seg003_point5 - Shear_Rate_seg003_point4)/Time_duration4

Shear_Rate_seg003_a_min = min(c(Shear_Rate_seg003_a1,Shear_Rate_seg003_a2,Shear_Rate_seg003_a3,Shear_Rate_seg003_a4))

Shear_Rate_seg003_a_max = max(c(Shear_Rate_seg003_a1,Shear_Rate_seg003_a2,Shear_Rate_seg003_a3,Shear_Rate_seg003_a4))

Shear_Rate_seg004_point1 = as.numeric(Shear_data[24,col1])

Shear_Rate_seg004_point2 = as.numeric(Shear_data[24,col2])

Shear_Rate_seg004_point3 = as.numeric(Shear_data[24,col3])

Shear_Rate_seg004_point4 = as.numeric(Shear_data[24,col4])

Shear_Rate_seg004_point5 = as.numeric(Shear_data[24,col5])

Shear_Rate_seg004_a1 = (Shear_Rate_seg004_point2 - Shear_Rate_seg004_point1)/Time_duration1

Shear_Rate_seg004_a2 = (Shear_Rate_seg004_point3 - Shear_Rate_seg004_point2)/Time_duration2

Shear_Rate_seg004_a3 = (Shear_Rate_seg004_point4 - Shear_Rate_seg004_point3)/Time_duration3

Shear_Rate_seg004_a4 = (Shear_Rate_seg004_point5 - Shear_Rate_seg004_point4)/Time_duration4

Shear_Rate_seg004_a_min = min(c(Shear_Rate_seg004_a1,Shear_Rate_seg004_a2,Shear_Rate_seg004_a3,Shear_Rate_seg004_a4))

Shear_Rate_seg004_a_max = max(c(Shear_Rate_seg004_a1,Shear_Rate_seg004_a2,Shear_Rate_seg004_a3,Shear_Rate_seg004_a4))

Shear_Rate_seg005_point1 = as.numeric(Shear_data[25,col1])

Shear_Rate_seg005_point2 = as.numeric(Shear_data[25,col2])

Shear_Rate_seg005_point3 = as.numeric(Shear_data[25,col3])

Shear_Rate_seg005_point4 = as.numeric(Shear_data[25,col4])

Shear_Rate_seg005_point5 = as.numeric(Shear_data[25,col5])

Shear_Rate_seg005_a1 = (Shear_Rate_seg005_point2 - Shear_Rate_seg005_point1)/Time_duration1

Shear_Rate_seg005_a2 = (Shear_Rate_seg005_point3 - Shear_Rate_seg005_point2)/Time_duration2

Shear_Rate_seg005_a3 = (Shear_Rate_seg005_point4 - Shear_Rate_seg005_point3)/Time_duration3

Shear_Rate_seg005_a4 = (Shear_Rate_seg005_point5 - Shear_Rate_seg005_point4)/Time_duration4

Shear_Rate_seg005_a_min = min(c(Shear_Rate_seg005_a1,Shear_Rate_seg005_a2,Shear_Rate_seg005_a3,Shear_Rate_seg005_a4))

Shear_Rate_seg005_a_max = max(c(Shear_Rate_seg005_a1,Shear_Rate_seg005_a2,Shear_Rate_seg005_a3,Shear_Rate_seg005_a4))

Shear_Rate_seg006_point1 = as.numeric(Shear_data[26,col1])

Shear_Rate_seg006_point2 = as.numeric(Shear_data[26,col2])

Shear_Rate_seg006_point3 = as.numeric(Shear_data[26,col3])

Shear_Rate_seg006_point4 = as.numeric(Shear_data[26,col4])

Shear_Rate_seg006_point5 = as.numeric(Shear_data[26,col5])

Shear_Rate_seg006_a1 = (Shear_Rate_seg006_point2 - Shear_Rate_seg006_point1)/Time_duration1

Shear_Rate_seg006_a2 = (Shear_Rate_seg006_point3 - Shear_Rate_seg006_point2)/Time_duration2

Shear_Rate_seg006_a3 = (Shear_Rate_seg006_point4 - Shear_Rate_seg006_point3)/Time_duration3

Shear_Rate_seg006_a4 = (Shear_Rate_seg006_point5 - Shear_Rate_seg006_point4)/Time_duration4

Shear_Rate_seg006_a_min = min(c(Shear_Rate_seg006_a1,Shear_Rate_seg006_a2,Shear_Rate_seg006_a3,Shear_Rate_seg006_a4))

Shear_Rate_seg006_a_max = max(c(Shear_Rate_seg006_a1,Shear_Rate_seg006_a2,Shear_Rate_seg006_a3,Shear_Rate_seg006_a4))

##Average_values_per_frame_Shear_Rate

Average_values_per_frame_Shear_Rate_point1 = as.numeric(Shear_data[30,col1])

Average_values_per_frame_Shear_Rate_point2 = as.numeric(Shear_data[30,col2])

Average_values_per_frame_Shear_Rate_point3 = as.numeric(Shear_data[30,col3])

Average_values_per_frame_Shear_Rate_point4 = as.numeric(Shear_data[30,col4])

Average_values_per_frame_Shear_Rate_point5 = as.numeric(Shear_data[30,col5])

Average_values_per_frame_Shear_Rate_a1 = (Average_values_per_frame_Shear_Rate_point2 - Average_values_per_frame_Shear_Rate_point1)/Time_duration1

Average_values_per_frame_Shear_Rate_a2 = (Average_values_per_frame_Shear_Rate_point3 - Average_values_per_frame_Shear_Rate_point2)/Time_duration2

Average_values_per_frame_Shear_Rate_a3 = (Average_values_per_frame_Shear_Rate_point4 - Average_values_per_frame_Shear_Rate_point3)/Time_duration3

Average_values_per_frame_Shear_Rate_a4 = (Average_values_per_frame_Shear_Rate_point5 - Average_values_per_frame_Shear_Rate_point4)/Time_duration4

Average_values_per_frame_Shear_Rate_a_min = min(c(Average_values_per_frame_Shear_Rate_a1,Average_values_per_frame_Shear_Rate_a2,Average_values_per_frame_Shear_Rate_a3,Average_values_per_frame_Shear_Rate_a4))

Average_values_per_frame_Shear_Rate_a_max = max(c(Average_values_per_frame_Shear_Rate_a1,Average_values_per_frame_Shear_Rate_a2,Average_values_per_frame_Shear_Rate_a3,Average_values_per_frame_Shear_Rate_a4))

#summary data

data_summary <- read.xlsx(i,sheetIndex = 1, header = F)

summary_type = data_summary[1,2]

summary_heart_rate = data_summary[5,2]

summary_RADIAL_VelocityEndo_T2P_seg1 = data_summary[23,2]

summary_RADIAL_VelocityEndo_T2P_seg2 = data_summary[23,3]

summary_RADIAL_VelocityEndo_T2P_seg3 = data_summary[23,4]

summary_RADIAL_VelocityEndo_T2P_seg4 = data_summary[23,5]

summary_RADIAL_VelocityEndo_T2P_seg5 = data_summary[23,6]

summary_RADIAL_VelocityEndo_T2P_seg6 = data_summary[23,7]

summary_RADIAL_VelocityEndo_T2P_ave = data_summary[23,8]

summary_RADIAL_VelocityEndo_Pk_seg1 = data_summary[24,2]

summary_RADIAL_VelocityEndo_Pk_seg2 = data_summary[24,3]

summary_RADIAL_VelocityEndo_Pk_seg3 = data_summary[24,4]

summary_RADIAL_VelocityEndo_Pk_seg4 = data_summary[24,5]

summary_RADIAL_VelocityEndo_Pk_seg5 = data_summary[24,6]

summary_RADIAL_VelocityEndo_Pk_seg6 = data_summary[24,7]

summary_RADIAL_VelocityEndo_Pk_ave = data_summary[24,8]

summary_RADIAL_VelocityEndo_PhasePercent_seg1 = data_summary[25,2]

summary_RADIAL_VelocityEndo_PhasePercent_seg2 = data_summary[25,3]

summary_RADIAL_VelocityEndo_PhasePercent_seg3 = data_summary[25,4]

summary_RADIAL_VelocityEndo_PhasePercent_seg4 = data_summary[25,5]

summary_RADIAL_VelocityEndo_PhasePercent_seg5 = data_summary[25,6]

summary_RADIAL_VelocityEndo_PhasePercent_seg6 = data_summary[25,7]

summary_RADIAL_VelocityEndo_PhasePercent_ave = data_summary[25,8]

summary_RADIAL_VelocityEndo_Phase_seg1 = data_summary[26,2]

summary_RADIAL_VelocityEndo_Phase_seg2 = data_summary[26,3]

summary_RADIAL_VelocityEndo_Phase_seg3 = data_summary[26,4]

summary_RADIAL_VelocityEndo_Phase_seg4 = data_summary[26,5]

summary_RADIAL_VelocityEndo_Phase_seg5 = data_summary[26,6]

summary_RADIAL_VelocityEndo_Phase_seg6 = data_summary[26,7]

summary_RADIAL_VelocityEndo_Phase_ave = data_summary[26,8]

summary_RADIAL_VelocityEndo_MaximumOpposingWallDelay = data_summary[27,2]

summary_RADIAL_VelocityEpi_T2P_seg1 = data_summary[23,12]

summary_RADIAL_VelocityEpi_T2P_seg2 = data_summary[23,13]

summary_RADIAL_VelocityEpi_T2P_seg3 = data_summary[23,14]

summary_RADIAL_VelocityEpi_T2P_seg4 = data_summary[23,15]

summary_RADIAL_VelocityEpi_T2P_seg5 = data_summary[23,16]

summary_RADIAL_VelocityEpi_T2P_seg6 = data_summary[23,17]

summary_RADIAL_VelocityEpi_T2P_ave = data_summary[23,18]

summary_RADIAL_VelocityEpi_Pk_seg1 = data_summary[24,12]

summary_RADIAL_VelocityEpi_Pk_seg2 = data_summary[24,13]

summary_RADIAL_VelocityEpi_Pk_seg3 = data_summary[24,14]

summary_RADIAL_VelocityEpi_Pk_seg4 = data_summary[24,15]

summary_RADIAL_VelocityEpi_Pk_seg5 = data_summary[24,16]

summary_RADIAL_VelocityEpi_Pk_seg6 = data_summary[24,17]

summary_RADIAL_VelocityEpi_Pk_ave = data_summary[24,18]

summary_RADIAL_VelocityEpi_PhasePercent_seg1 = data_summary[25,12]

summary_RADIAL_VelocityEpi_PhasePercent_seg2 = data_summary[25,13]

summary_RADIAL_VelocityEpi_PhasePercent_seg3 = data_summary[25,14]

summary_RADIAL_VelocityEpi_PhasePercent_seg4 = data_summary[25,15]

summary_RADIAL_VelocityEpi_PhasePercent_seg5 = data_summary[25,16]

summary_RADIAL_VelocityEpi_PhasePercent_seg6 = data_summary[25,17]

summary_RADIAL_VelocityEpi_PhasePercent_ave = data_summary[25,18]

summary_RADIAL_VelocityEpi_Phase_seg1 = data_summary[26,12]

summary_RADIAL_VelocityEpi_Phase_seg2 = data_summary[26,13]

summary_RADIAL_VelocityEpi_Phase_seg3 = data_summary[26,14]

summary_RADIAL_VelocityEpi_Phase_seg4 = data_summary[26,15]

summary_RADIAL_VelocityEpi_Phase_seg5 = data_summary[26,16]

summary_RADIAL_VelocityEpi_Phase_seg6 = data_summary[26,17]

summary_RADIAL_VelocityEpi_Phase_ave = data_summary[26,18]

summary_RADIAL_VelocityEpi_MaximumOpposingWallDelay = data_summary[27,12]

summary_RADIAL_DisplacementEndo_T2P_seg1 = data_summary[31,2]

summary_RADIAL_DisplacementEndo_T2P_seg2 = data_summary[31,3]

summary_RADIAL_DisplacementEndo_T2P_seg3 = data_summary[31,4]

summary_RADIAL_DisplacementEndo_T2P_seg4 = data_summary[31,5]

summary_RADIAL_DisplacementEndo_T2P_seg5 = data_summary[31,6]

summary_RADIAL_DisplacementEndo_T2P_seg6 = data_summary[31,7]

summary_RADIAL_DisplacementEndo_T2P_ave = data_summary[31,8]

summary_RADIAL_DisplacementEndo_Pk_seg1 = data_summary[32,2]

summary_RADIAL_DisplacementEndo_Pk_seg2 = data_summary[32,3]

summary_RADIAL_DisplacementEndo_Pk_seg3 = data_summary[32,4]

summary_RADIAL_DisplacementEndo_Pk_seg4 = data_summary[32,5]

summary_RADIAL_DisplacementEndo_Pk_seg5 = data_summary[32,6]

summary_RADIAL_DisplacementEndo_Pk_seg6 = data_summary[32,7]

summary_RADIAL_DisplacementEndo_Pk_ave = data_summary[32,8]

summary_RADIAL_DisplacementEndo_PhasePercent_seg1 = data_summary[33,2]

summary_RADIAL_DisplacementEndo_PhasePercent_seg2 = data_summary[33,3]

summary_RADIAL_DisplacementEndo_PhasePercent_seg3 = data_summary[33,4]

summary_RADIAL_DisplacementEndo_PhasePercent_seg4 = data_summary[33,5]

summary_RADIAL_DisplacementEndo_PhasePercent_seg5 = data_summary[33,6]

summary_RADIAL_DisplacementEndo_PhasePercent_seg6 = data_summary[33,7]

summary_RADIAL_DisplacementEndo_PhasePercent_ave = data_summary[33,8]

summary_RADIAL_DisplacementEndo_Phase_seg1 = data_summary[34,2]

summary_RADIAL_DisplacementEndo_Phase_seg2 = data_summary[34,3]

summary_RADIAL_DisplacementEndo_Phase_seg3 = data_summary[34,4]

summary_RADIAL_DisplacementEndo_Phase_seg4 = data_summary[34,5]

summary_RADIAL_DisplacementEndo_Phase_seg5 = data_summary[34,6]

summary_RADIAL_DisplacementEndo_Phase_seg6 = data_summary[34,7]

summary_RADIAL_DisplacementEndo_Phase_ave = data_summary[34,8]

summary_RADIAL_DisplacementEndo_MaximumOpposingWallDelay = data_summary[35,2]

summary_RADIAL_DisplacementEpi_T2P_seg1 = data_summary[31,12]

summary_RADIAL_DisplacementEpi_T2P_seg2 = data_summary[31,13]

summary_RADIAL_DisplacementEpi_T2P_seg3 = data_summary[31,14]

summary_RADIAL_DisplacementEpi_T2P_seg4 = data_summary[31,15]

summary_RADIAL_DisplacementEpi_T2P_seg5 = data_summary[31,16]

summary_RADIAL_DisplacementEpi_T2P_seg6 = data_summary[31,17]

summary_RADIAL_DisplacementEpi_T2P_ave = data_summary[31,18]

summary_RADIAL_DisplacementEpi_Pk_seg1 = data_summary[32,12]

summary_RADIAL_DisplacementEpi_Pk_seg2 = data_summary[32,13]

summary_RADIAL_DisplacementEpi_Pk_seg3 = data_summary[32,14]

summary_RADIAL_DisplacementEpi_Pk_seg4 = data_summary[32,15]

summary_RADIAL_DisplacementEpi_Pk_seg5 = data_summary[32,16]

summary_RADIAL_DisplacementEpi_Pk_seg6 = data_summary[32,17]

summary_RADIAL_DisplacementEpi_Pk_ave = data_summary[32,18]

summary_RADIAL_DisplacementEpi_PhasePercent_seg1 = data_summary[33,12]

summary_RADIAL_DisplacementEpi_PhasePercent_seg2 = data_summary[33,13]

summary_RADIAL_DisplacementEpi_PhasePercent_seg3 = data_summary[33,14]

summary_RADIAL_DisplacementEpi_PhasePercent_seg4 = data_summary[33,15]

summary_RADIAL_DisplacementEpi_PhasePercent_seg5 = data_summary[33,16]

summary_RADIAL_DisplacementEpi_PhasePercent_seg6 = data_summary[33,17]

summary_RADIAL_DisplacementEpi_PhasePercent_ave = data_summary[33,18]

summary_RADIAL_DisplacementEpi_Phase_seg1 = data_summary[34,12]

summary_RADIAL_DisplacementEpi_Phase_seg2 = data_summary[34,13]

summary_RADIAL_DisplacementEpi_Phase_seg3 = data_summary[34,14]

summary_RADIAL_DisplacementEpi_Phase_seg4 = data_summary[34,15]

summary_RADIAL_DisplacementEpi_Phase_seg5 = data_summary[34,16]

summary_RADIAL_DisplacementEpi_Phase_seg6 = data_summary[34,17]

summary_RADIAL_DisplacementEpi_Phase_ave = data_summary[34,18]

summary_RADIAL_DisplacementEpi_MaximumOpposingWallDelay = data_summary[35,12]

summary_RADIAL_StrainEndo_T2P_seg1 = data_summary[39,2]

summary_RADIAL_StrainEndo_T2P_seg2 = data_summary[39,3]

summary_RADIAL_StrainEndo_T2P_seg3 = data_summary[39,4]

summary_RADIAL_StrainEndo_T2P_seg4 = data_summary[39,5]

summary_RADIAL_StrainEndo_T2P_seg5 = data_summary[39,6]

summary_RADIAL_StrainEndo_T2P_seg6 = data_summary[39,7]

summary_RADIAL_StrainEndo_T2P_ave = data_summary[39,8]

summary_RADIAL_StrainEndo_Pk_seg1 = data_summary[40,2]

summary_RADIAL_StrainEndo_Pk_seg2 = data_summary[40,3]

summary_RADIAL_StrainEndo_Pk_seg3 = data_summary[40,4]

summary_RADIAL_StrainEndo_Pk_seg4 = data_summary[40,5]

summary_RADIAL_StrainEndo_Pk_seg5 = data_summary[40,6]

summary_RADIAL_StrainEndo_Pk_seg6 = data_summary[40,7]

summary_RADIAL_StrainEndo_Pk_ave = data_summary[40,8]

summary_RADIAL_StrainEndo_PhasePercent_seg1 = data_summary[41,2]

summary_RADIAL_StrainEndo_PhasePercent_seg2 = data_summary[41,3]

summary_RADIAL_StrainEndo_PhasePercent_seg3 = data_summary[41,4]

summary_RADIAL_StrainEndo_PhasePercent_seg4 = data_summary[41,5]

summary_RADIAL_StrainEndo_PhasePercent_seg5 = data_summary[41,6]

summary_RADIAL_StrainEndo_PhasePercent_seg6 = data_summary[41,7]

summary_RADIAL_StrainEndo_PhasePercent_ave = data_summary[41,8]

summary_RADIAL_StrainEndo_Phase_seg1 = data_summary[42,2]

summary_RADIAL_StrainEndo_Phase_seg2 = data_summary[42,3]

summary_RADIAL_StrainEndo_Phase_seg3 = data_summary[42,4]

summary_RADIAL_StrainEndo_Phase_seg4 = data_summary[42,5]

summary_RADIAL_StrainEndo_Phase_seg5 = data_summary[42,6]

summary_RADIAL_StrainEndo_Phase_seg6 = data_summary[42,7]

summary_RADIAL_StrainEndo_Phase_ave = data_summary[42,8]

summary_RADIAL_StrainEndo_MaximumOpposingWallDelay = data_summary[43,2]

summary_RADIAL_StrainEpi_T2P_seg1 = data_summary[39,12]

summary_RADIAL_StrainEpi_T2P_seg2 = data_summary[39,13]

summary_RADIAL_StrainEpi_T2P_seg3 = data_summary[39,14]

summary_RADIAL_StrainEpi_T2P_seg4 = data_summary[39,15]

summary_RADIAL_StrainEpi_T2P_seg5 = data_summary[39,16]

summary_RADIAL_StrainEpi_T2P_seg6 = data_summary[39,17]

summary_RADIAL_StrainEpi_T2P_ave = data_summary[39,18]

summary_RADIAL_StrainEpi_Pk_seg1 = data_summary[40,12]

summary_RADIAL_StrainEpi_Pk_seg2 = data_summary[40,13]

summary_RADIAL_StrainEpi_Pk_seg3 = data_summary[40,14]

summary_RADIAL_StrainEpi_Pk_seg4 = data_summary[40,15]

summary_RADIAL_StrainEpi_Pk_seg5 = data_summary[40,16]

summary_RADIAL_StrainEpi_Pk_seg6 = data_summary[40,17]

summary_RADIAL_StrainEpi_Pk_ave = data_summary[40,18]

summary_RADIAL_StrainEpi_PhasePercent_seg1 = data_summary[41,12]

summary_RADIAL_StrainEpi_PhasePercent_seg2 = data_summary[41,13]

summary_RADIAL_StrainEpi_PhasePercent_seg3 = data_summary[41,14]

summary_RADIAL_StrainEpi_PhasePercent_seg4 = data_summary[41,15]

summary_RADIAL_StrainEpi_PhasePercent_seg5 = data_summary[41,16]

summary_RADIAL_StrainEpi_PhasePercent_seg6 = data_summary[41,17]

summary_RADIAL_StrainEpi_PhasePercent_ave = data_summary[41,18]

summary_RADIAL_StrainEpi_Phase_seg1 = data_summary[42,12]

summary_RADIAL_StrainEpi_Phase_seg2 = data_summary[42,13]

summary_RADIAL_StrainEpi_Phase_seg3 = data_summary[42,14]

summary_RADIAL_StrainEpi_Phase_seg4 = data_summary[42,15]

summary_RADIAL_StrainEpi_Phase_seg5 = data_summary[42,16]

summary_RADIAL_StrainEpi_Phase_seg6 = data_summary[42,17]

summary_RADIAL_StrainEpi_Phase_ave = data_summary[42,18]

summary_RADIAL_StrainEpi_MaximumOpposingWallDelay = data_summary[43,12]

summary_RADIAL_StrainRateEndo_T2P_seg1 = data_summary[47,2]

summary_RADIAL_StrainRateEndo_T2P_seg2 = data_summary[47,3]

summary_RADIAL_StrainRateEndo_T2P_seg3 = data_summary[47,4]

summary_RADIAL_StrainRateEndo_T2P_seg4 = data_summary[47,5]

summary_RADIAL_StrainRateEndo_T2P_seg5 = data_summary[47,6]

summary_RADIAL_StrainRateEndo_T2P_seg6 = data_summary[47,7]

summary_RADIAL_StrainRateEndo_T2P_ave = data_summary[47,8]

summary_RADIAL_StrainRateEndo_Pk_seg1 = data_summary[48,2]

summary_RADIAL_StrainRateEndo_Pk_seg2 = data_summary[48,3]

summary_RADIAL_StrainRateEndo_Pk_seg3 = data_summary[48,4]

summary_RADIAL_StrainRateEndo_Pk_seg4 = data_summary[48,5]

summary_RADIAL_StrainRateEndo_Pk_seg5 = data_summary[48,6]

summary_RADIAL_StrainRateEndo_Pk_seg6 = data_summary[48,7]

summary_RADIAL_StrainRateEndo_Pk_ave = data_summary[48,8]

summary_RADIAL_StrainRateEndo_PhasePercent_seg1 = data_summary[49,2]

summary_RADIAL_StrainRateEndo_PhasePercent_seg2 = data_summary[49,3]

summary_RADIAL_StrainRateEndo_PhasePercent_seg3 = data_summary[49,4]

summary_RADIAL_StrainRateEndo_PhasePercent_seg4 = data_summary[49,5]

summary_RADIAL_StrainRateEndo_PhasePercent_seg5 = data_summary[49,6]

summary_RADIAL_StrainRateEndo_PhasePercent_seg6 = data_summary[49,7]

summary_RADIAL_StrainRateEndo_PhasePercent_ave = data_summary[49,8]

summary_RADIAL_StrainRateEndo_Phase_seg1 = data_summary[50,2]

summary_RADIAL_StrainRateEndo_Phase_seg2 = data_summary[50,3]

summary_RADIAL_StrainRateEndo_Phase_seg3 = data_summary[50,4]

summary_RADIAL_StrainRateEndo_PhaseP_seg4 = data_summary[50,5]

summary_RADIAL_StrainRateEndo_Phase_seg5 = data_summary[50,6]

summary_RADIAL_StrainRateEndo_Phase_seg6 = data_summary[50,7]

summary_RADIAL_StrainRateEndo_Phase_ave = data_summary[50,8]

summary_RADIAL_StrainRateEndo_MaximumOpposingWallDelay = data_summary[51,2]

summary_RADIAL_StrainRateEpi_T2P_seg1 = data_summary[47,12]

summary_RADIAL_StrainRateEpi_T2P_seg2 = data_summary[47,13]

summary_RADIAL_StrainRateEpi_T2P_seg3 = data_summary[47,14]

summary_RADIAL_StrainRateEpi_T2P_seg4 = data_summary[47,15]

summary_RADIAL_StrainRateEpi_T2P_seg5 = data_summary[47,16]

summary_RADIAL_StrainRateEpi_T2P_seg6 = data_summary[47,17]

summary_RADIAL_StrainRateEpi_T2P_ave = data_summary[47,18]

summary_RADIAL_StrainRateEpi_Pk_seg1 = data_summary[48,12]

summary_RADIAL_StrainRateEpi_Pk_seg2 = data_summary[48,13]

summary_RADIAL_StrainRateEpi_Pk_seg3 = data_summary[48,14]

summary_RADIAL_StrainRateEpi_Pk_seg4 = data_summary[48,15]

summary_RADIAL_StrainRateEpi_Pk_seg5 = data_summary[48,16]

summary_RADIAL_StrainRateEpi_Pk_seg6 = data_summary[48,17]

summary_RADIAL_StrainRateEpi_Pk_ave = data_summary[48,18]

summary_RADIAL_StrainRateEpi_PhasePercent_seg1 = data_summary[49,12]

summary_RADIAL_StrainRateEpi_PhasePercent_seg2 = data_summary[49,13]

summary_RADIAL_StrainRateEpi_PhasePercent_seg3 = data_summary[49,14]

summary_RADIAL_StrainRateEpi_PhasePercent_seg4 = data_summary[49,15]

summary_RADIAL_StrainRateEpi_PhasePercent_seg5 = data_summary[49,16]

summary_RADIAL_StrainRateEpi_PhasePercent_seg6 = data_summary[49,17]

summary_RADIAL_StrainRateEpi_PhasePercent_ave = data_summary[49,18]

summary_RADIAL_StrainRateEpi_Phase_seg1 = data_summary[50,12]

summary_RADIAL_StrainRateEpi_Phase_seg2 = data_summary[50,13]

summary_RADIAL_StrainRateEpi_Phase_seg3 = data_summary[50,14]

summary_RADIAL_StrainRateEpi_Phase_seg4 = data_summary[50,15]

summary_RADIAL_StrainRateEpi_Phase_seg5 = data_summary[50,16]

summary_RADIAL_StrainRateEpi_Phase_seg6 = data_summary[50,17]

summary_RADIAL_StrainRateEpi_Phase_ave = data_summary[50,18]

summary_RADIAL_StrainRateEpi_MaximumOpposingWallDelay = data_summary[51,12]

summary_LONGITUDINAL_VelocityEndo_T2P_seg1 = data_summary[56,2]

summary_LONGITUDINAL_VelocityEndo_T2P_seg2 = data_summary[56,3]

summary_LONGITUDINAL_VelocityEndo_T2P_seg3 = data_summary[56,4]

summary_LONGITUDINAL_VelocityEndo_T2P_seg4 = data_summary[56,5]

summary_LONGITUDINAL_VelocityEndo_T2P_seg5 = data_summary[56,6]

summary_LONGITUDINAL_VelocityEndo_T2P_seg6 = data_summary[56,7]

summary_LONGITUDINAL_VelocityEndo_T2P_ave = data_summary[56,8]

summary_LONGITUDINAL_VelocityEndo_Pk_seg1 = data_summary[57,2]

summary_LONGITUDINAL_VelocityEndo_Pk_seg2 = data_summary[57,3]

summary_LONGITUDINAL_VelocityEndo_Pk_seg3 = data_summary[57,4]

summary_LONGITUDINAL_VelocityEndo_Pk_seg4 = data_summary[57,5]

summary_LONGITUDINAL_VelocityEndo_Pk_seg5 = data_summary[57,6]

summary_LONGITUDINAL_VelocityEndo_Pk_seg6 = data_summary[57,7]

summary_LONGITUDINAL_VelocityEndo_Pk_ave = data_summary[57,8]

summary_LONGITUDINAL_VelocityEndo_PhasePercent_seg1 = data_summary[58,2]

summary_LONGITUDINAL_VelocityEndo_PhasePercent_seg2 = data_summary[58,3]

summary_LONGITUDINAL_VelocityEndo_PhasePercent_seg3 = data_summary[58,4]

summary_LONGITUDINAL_VelocityEndo_PhasePercent_seg4 = data_summary[58,5]

summary_LONGITUDINAL_VelocityEndo_PhasePercent_seg5 = data_summary[58,6]

summary_LONGITUDINAL_VelocityEndo_PhasePercent_seg6 = data_summary[58,7]

summary_LONGITUDINAL_VelocityEndo_PhasePercent_ave = data_summary[58,8]

summary_LONGITUDINAL_VelocityEndo_Phase_seg1 = data_summary[59,2]

summary_LONGITUDINAL_VelocityEndo_Phase_seg2 = data_summary[59,3]

summary_LONGITUDINAL_VelocityEndo_Phase_seg3 = data_summary[59,4]

summary_LONGITUDINAL_VelocityEndo_Phase_seg4 = data_summary[59,5]

summary_LONGITUDINAL_VelocityEndo_Phase_seg5 = data_summary[59,6]

summary_LONGITUDINAL_VelocityEndo_Phase_seg6 = data_summary[59,7]

summary_LONGITUDINAL_VelocityEndo_Phase_ave = data_summary[59,8]

summary_LONGITUDINAL_VelocityEndo_MaximumOpposingWallDelay = data_summary[60,2]

summary_LONGITUDINAL_VelocityEpi_T2P_seg1 = data_summary[56,12]

summary_LONGITUDINAL_VelocityEpi_T2P_seg2 = data_summary[56,13]

summary_LONGITUDINAL_VelocityEpi_T2P_seg3 = data_summary[56,14]

summary_LONGITUDINAL_VelocityEpi_T2P_seg4 = data_summary[56,15]

summary_LONGITUDINAL_VelocityEpi_T2P_seg5 = data_summary[56,16]

summary_LONGITUDINAL_VelocityEpi_T2P_seg6 = data_summary[56,17]

summary_LONGITUDINAL_VelocityEpi_T2P_ave = data_summary[56,18]

summary_LONGITUDINAL_VelocityEpi_Pk_seg1 = data_summary[57,12]

summary_LONGITUDINAL_VelocityEpi_Pk_seg2 = data_summary[57,13]

summary_LONGITUDINAL_VelocityEpi_Pk_seg3 = data_summary[57,14]

summary_LONGITUDINAL_VelocityEpi_Pk_seg4 = data_summary[57,15]

summary_LONGITUDINAL_VelocityEpi_Pk_seg5 = data_summary[57,16]

summary_LONGITUDINAL_VelocityEpi_Pk_seg6 = data_summary[57,17]

summary_LONGITUDINAL_VelocityEpi_Pk_ave = data_summary[57,18]

summary_LONGITUDINAL_VelocityEpi_PhasePercent_seg1 = data_summary[58,12]

summary_LONGITUDINAL_VelocityEpi_PhasePercent_seg2 = data_summary[58,13]

summary_LONGITUDINAL_VelocityEpi_PhasePercent_seg3 = data_summary[58,14]

summary_LONGITUDINAL_VelocityEpi_PhasePercent_seg4 = data_summary[58,15]

summary_LONGITUDINAL_VelocityEpi_PhasePercent_seg5 = data_summary[58,16]

summary_LONGITUDINAL_VelocityEpi_PhasePercent_seg6 = data_summary[58,17]

summary_LONGITUDINAL_VelocityEpi_PhasePercent_ave = data_summary[58,18]

summary_LONGITUDINAL_VelocityEpi_Phase_seg1 = data_summary[59,12]

summary_LONGITUDINAL_VelocityEpi_Phase_seg2 = data_summary[59,13]

summary_LONGITUDINAL_VelocityEpi_Phase_seg3 = data_summary[59,14]

summary_LONGITUDINAL_VelocityEpi_Phase_seg4 = data_summary[59,15]

summary_LONGITUDINAL_VelocityEpi_Phase_seg5 = data_summary[59,16]

summary_LONGITUDINAL_VelocityEpi_Phase_seg6 = data_summary[59,17]

summary_LONGITUDINAL_VelocityEpi_Phase_ave = data_summary[59,18]

summary_LONGITUDINAL_VelocityEpi_MaximumOpposingWallDelay = data_summary[60,12]

summary_LONGITUDINAL_DisplacementEndo_T2P_seg1 = data_summary[64,2]

summary_LONGITUDINAL_DisplacementEndo_T2P_seg2 = data_summary[64,3]

summary_LONGITUDINAL_DisplacementEndo_T2P_seg3 = data_summary[64,4]

summary_LONGITUDINAL_DisplacementEndo_T2P_seg4 = data_summary[64,5]

summary_LONGITUDINAL_DisplacementEndo_T2P_seg5 = data_summary[64,6]

summary_LONGITUDINAL_DisplacementEndo_T2P_seg6 = data_summary[64,7]

summary_LONGITUDINAL_DisplacementEndo_T2P_ave = data_summary[64,8]

summary_LONGITUDINAL_DisplacementEndo_Pk_seg1 = data_summary[65,2]

summary_LONGITUDINAL_DisplacementEndo_Pk_seg2 = data_summary[65,3]

summary_LONGITUDINAL_DisplacementEndo_Pk_seg3 = data_summary[65,4]

summary_LONGITUDINAL_DisplacementEndo_Pk_seg4 = data_summary[65,5]

summary_LONGITUDINAL_DisplacementEndo_Pk_seg5 = data_summary[65,6]

summary_LONGITUDINAL_DisplacementEndo_Pk_seg6 = data_summary[65,7]

summary_LONGITUDINAL_DisplacementEndo_Pk_ave = data_summary[65,8]

summary_LONGITUDINAL_DisplacementEndo_PhasePercent_seg1 = data_summary[66,2]

summary_LONGITUDINAL_DisplacementEndo_PhasePercent_seg2 = data_summary[66,3]

summary_LONGITUDINAL_DisplacementEndo_PhasePercent_seg3 = data_summary[66,4]

summary_LONGITUDINAL_DisplacementEndo_PhasePercent_seg4 = data_summary[66,5]

summary_LONGITUDINAL_DisplacementEndo_PhasePercent_seg5 = data_summary[66,6]

summary_LONGITUDINAL_DisplacementEndo_PhasePercent_seg6 = data_summary[66,7]

summary_LONGITUDINAL_DisplacementEndo_PhasePercent_ave = data_summary[66,8]

summary_LONGITUDINAL_DisplacementEndo_Phase_seg1 = data_summary[67,2]

summary_LONGITUDINAL_DisplacementEndo_Phase_seg2 = data_summary[67,3]

summary_LONGITUDINAL_DisplacementEndo_Phase_seg3 = data_summary[67,4]

summary_LONGITUDINAL_DisplacementEndo_Phase_seg4 = data_summary[67,5]

summary_LONGITUDINAL_DisplacementEndo_Phase_seg5 = data_summary[67,6]

summary_LONGITUDINAL_DisplacementEndo_Phase_seg6 = data_summary[67,7]

summary_LONGITUDINAL_DisplacementEndo_Phase_ave = data_summary[67,8]

summary_LONGITUDINAL_DisplacementEndo_MaximumOpposingWallDelay = data_summary[68,2]

summary_LONGITUDINAL_DisplacementEpi_T2P_seg1 = data_summary[64,12]

summary_LONGITUDINAL_DisplacementEpi_T2P_seg2 = data_summary[64,13]

summary_LONGITUDINAL_DisplacementEpi_T2P_seg3 = data_summary[64,14]

summary_LONGITUDINAL_DisplacementEpi_T2P_seg4 = data_summary[64,15]

summary_LONGITUDINAL_DisplacementEpi_T2P_seg5 = data_summary[64,16]

summary_LONGITUDINAL_DisplacementEpi_T2P_seg6 = data_summary[64,17]

summary_LONGITUDINAL_DisplacementEpi_T2P_ave = data_summary[64,18]

summary_LONGITUDINAL_DisplacementEpi_Pk_seg1 = data_summary[65,12]

summary_LONGITUDINAL_DisplacementEpi_Pk_seg2 = data_summary[65,13]

summary_LONGITUDINAL_DisplacementEpi_Pk_seg3 = data_summary[65,14]

summary_LONGITUDINAL_DisplacementEpi_Pk_seg4 = data_summary[65,15]

summary_LONGITUDINAL_DisplacementEpi_Pk_seg5 = data_summary[65,16]

summary_LONGITUDINAL_DisplacementEpi_Pk_seg6 = data_summary[65,17]

summary_LONGITUDINAL_DisplacementEpi_Pk_ave = data_summary[65,18]

summary_LONGITUDINAL_DisplacementEpi_PhasePercent_seg1 = data_summary[66,12]

summary_LONGITUDINAL_DisplacementEpi_PhasePercent_seg2 = data_summary[66,13]

summary_LONGITUDINAL_DisplacementEpi_PhasePercent_seg3 = data_summary[66,14]

summary_LONGITUDINAL_DisplacementEpi_PhasePercent_seg4 = data_summary[66,15]

summary_LONGITUDINAL_DisplacementEpi_PhasePercent_seg5 = data_summary[66,16]

summary_LONGITUDINAL_DisplacementEpi_PhasePercent_seg6 = data_summary[66,17]

summary_LONGITUDINAL_DisplacementEpi_PhasePercent_ave = data_summary[66,18]

summary_LONGITUDINAL_DisplacementEpi_Phase_seg1 = data_summary[67,12]

summary_LONGITUDINAL_DisplacementEpi_Phase_seg2 = data_summary[67,13]

summary_LONGITUDINAL_DisplacementEpi_Phase_seg3 = data_summary[67,14]

summary_LONGITUDINAL_DisplacementEpi_Phase_seg4 = data_summary[67,15]

summary_LONGITUDINAL_DisplacementEpi_Phase_seg5 = data_summary[67,16]

summary_LONGITUDINAL_DisplacementEpi_Phase_seg6 = data_summary[67,17]

summary_LONGITUDINAL_DisplacementEpi_Phase_ave = data_summary[67,18]

summary_LONGITUDINAL_DisplacementEpi_MaximumOpposingWallDelay = data_summary[68,12]

summary_LONGITUDINAL_StrainEndo_T2P_seg1 = data_summary[72,2]

summary_LONGITUDINAL_StrainEndo_T2P_seg2 = data_summary[72,3]

summary_LONGITUDINAL_StrainEndo_T2P_seg3 = data_summary[72,4]

summary_LONGITUDINAL_StrainEndo_T2P_seg4 = data_summary[72,5]

summary_LONGITUDINAL_StrainEndo_T2P_seg5 = data_summary[72,6]

summary_LONGITUDINAL_StrainEndo_T2P_seg6 = data_summary[72,7]

summary_LONGITUDINAL_StrainEndo_T2P_ave = data_summary[72,8]

summary_LONGITUDINAL_StrainEndo_Pk_seg1 = data_summary[73,2]

summary_LONGITUDINAL_StrainEndo_Pk_seg2 = data_summary[73,3]

summary_LONGITUDINAL_StrainEndo_Pk_seg3 = data_summary[73,4]

summary_LONGITUDINAL_StrainEndo_Pk_seg4 = data_summary[73,5]

summary_LONGITUDINAL_StrainEndo_Pk_seg5 = data_summary[73,6]

summary_LONGITUDINAL_StrainEndo_Pk_seg6 = data_summary[73,7]

summary_LONGITUDINAL_StrainEndo_Pk_ave = data_summary[73,8]

summary_LONGITUDINAL_StrainEndo_PhasePercent_seg1 = data_summary[74,2]

summary_LONGITUDINAL_StrainEndo_PhasePercent_seg2 = data_summary[74,3]

summary_LONGITUDINAL_StrainEndo_PhasePercent_seg3 = data_summary[74,4]

summary_LONGITUDINAL_StrainEndo_PhasePercent_seg4 = data_summary[74,5]

summary_LONGITUDINAL_StrainEndo_PhasePercent_seg5 = data_summary[74,6]

summary_LONGITUDINAL_StrainEndo_PhasePercent_seg6 = data_summary[74,7]

summary_LONGITUDINAL_StrainEndo_PhasePercent_ave = data_summary[74,8]

summary_LONGITUDINAL_StrainEndo_Phase_seg1 = data_summary[75,2]

summary_LONGITUDINAL_StrainEndo_Phase_seg2 = data_summary[75,3]

summary_LONGITUDINAL_StrainEndo_Phase_seg3 = data_summary[75,4]

summary_LONGITUDINAL_StrainEndo_Phase_seg4 = data_summary[75,5]

summary_LONGITUDINAL_StrainEndo_Phase_seg5 = data_summary[75,6]

summary_LONGITUDINAL_StrainEndo_Phase_seg6 = data_summary[75,7]

summary_LONGITUDINAL_StrainEndo_Phase_ave = data_summary[75,8]

summary_LONGITUDINAL_StrainEndo_MaximumOpposingWallDelay = data_summary[76,2]

summary_LONGITUDINAL_StrainEpi_T2P_seg1 = data_summary[72,12]

summary_LONGITUDINAL_StrainEpi_T2P_seg2 = data_summary[72,13]

summary_LONGITUDINAL_StrainEpi_T2P_seg3 = data_summary[72,14]

summary_LONGITUDINAL_StrainEpi_T2P_seg4 = data_summary[72,15]

summary_LONGITUDINAL_StrainEpi_T2P_seg5 = data_summary[72,16]

summary_LONGITUDINAL_StrainEpi_T2P_seg6 = data_summary[72,17]

summary_LONGITUDINAL_StrainEpi_T2P_ave = data_summary[72,18]

summary_LONGITUDINAL_StrainEpi_Pk_seg1 = data_summary[73,12]

summary_LONGITUDINAL_StrainEpi_Pk_seg2 = data_summary[73,13]

summary_LONGITUDINAL_StrainEpi_Pk_seg3 = data_summary[73,14]

summary_LONGITUDINAL_StrainEpi_Pk_seg4 = data_summary[73,15]

summary_LONGITUDINAL_StrainEpi_Pk_seg5 = data_summary[73,16]

summary_LONGITUDINAL_StrainEpi_Pk_seg6 = data_summary[73,17]

summary_LONGITUDINAL_StrainEpi_Pk_ave = data_summary[73,18]

summary_LONGITUDINAL_StrainEpi_PhasePercent_seg1 = data_summary[74,12]

summary_LONGITUDINAL_StrainEpi_PhasePercent_seg2 = data_summary[74,13]

summary_LONGITUDINAL_StrainEpi_PhasePercent_seg3 = data_summary[74,14]

summary_LONGITUDINAL_StrainEpi_PhasePercent_seg4 = data_summary[74,15]

summary_LONGITUDINAL_StrainEpi_PhasePercent_seg5 = data_summary[74,16]

summary_LONGITUDINAL_StrainEpi_PhasePercent_seg6 = data_summary[74,17]

summary_LONGITUDINAL_StrainEpi_PhasePercent_ave = data_summary[74,18]

summary_LONGITUDINAL_StrainEpi_Phase_seg1 = data_summary[75,12]

summary_LONGITUDINAL_StrainEpi_Phase_seg2 = data_summary[75,13]

summary_LONGITUDINAL_StrainEpi_Phase_seg3 = data_summary[75,14]

summary_LONGITUDINAL_StrainEpi_Phase_seg4 = data_summary[75,15]

summary_LONGITUDINAL_StrainEpi_Phase_seg5 = data_summary[75,16]

summary_LONGITUDINAL_StrainEpi_Phase_seg6 = data_summary[75,17]

summary_LONGITUDINAL_StrainEpi_Phase_ave = data_summary[75,18]

summary_LONGITUDINAL_StrainEpi_MaximumOpposingWallDelay = data_summary[76,12]

summary_LONGITUDINAL_StrainRateEndo_T2P_seg1 = data_summary[80,2]

summary_LONGITUDINAL_StrainRateEndo_T2P_seg2 = data_summary[80,3]

summary_LONGITUDINAL_StrainRateEndo_T2P_seg3 = data_summary[80,4]

summary_LONGITUDINAL_StrainRateEndo_T2P_seg4 = data_summary[80,5]

summary_LONGITUDINAL_StrainRateEndo_T2P_seg5 = data_summary[80,6]

summary_LONGITUDINAL_StrainRateEndo_T2P_seg6 = data_summary[80,7]

summary_LONGITUDINAL_StrainRateEndo_T2P_ave = data_summary[80,8]

summary_LONGITUDINAL_StrainRateEndo_Pk_seg1 = data_summary[81,2]

summary_LONGITUDINAL_StrainRateEndo_Pk_seg2 = data_summary[81,3]

summary_LONGITUDINAL_StrainRateEndo_Pk_seg3 = data_summary[81,4]

summary_LONGITUDINAL_StrainRateEndo_Pk_seg4 = data_summary[81,5]

summary_LONGITUDINAL_StrainRateEndo_Pk_seg5 = data_summary[81,6]

summary_LONGITUDINAL_StrainRateEndo_Pk_seg6 = data_summary[81,7]

summary_LONGITUDINAL_StrainRateEndo_Pk_ave = data_summary[81,8]

summary_LONGITUDINAL_StrainRateEndo_PhasePercent_seg1 = data_summary[82,2]

summary_LONGITUDINAL_StrainRateEndo_PhasePercent_seg2 = data_summary[82,3]

summary_LONGITUDINAL_StrainRateEndo_PhasePercent_seg3 = data_summary[82,4]

summary_LONGITUDINAL_StrainRateEndo_PhasePercent_seg4 = data_summary[82,5]

summary_LONGITUDINAL_StrainRateEndo_PhasePercent_seg5 = data_summary[82,6]

summary_LONGITUDINAL_StrainRateEndo_PhasePercent_seg6 = data_summary[82,7]

summary_LONGITUDINAL_StrainRateEndo_PhasePercent_ave = data_summary[82,8]

summary_LONGITUDINAL_StrainRateEndo_Phase_seg1 = data_summary[83,2]

summary_LONGITUDINAL_StrainRateEndo_Phase_seg2 = data_summary[83,3]

summary_LONGITUDINAL_StrainRateEndo_Phase_seg3 = data_summary[83,4]

summary_LONGITUDINAL_StrainRateEndo_Phase_seg4 = data_summary[83,5]

summary_LONGITUDINAL_StrainRateEndo_Phase_seg5 = data_summary[83,6]

summary_LONGITUDINAL_StrainRateEndo_Phase_seg6 = data_summary[83,7]

summary_LONGITUDINAL_StrainRateEndo_Phase_ave = data_summary[83,8]

summary_LONGITUDINAL_StrainRateEndo_MaximumOpposingWallDelay = data_summary[84,2]

summary_LONGITUDINAL_StrainRateEpi_T2P_seg1 = data_summary[80,12]

summary_LONGITUDINAL_StrainRateEpi_T2P_seg2 = data_summary[80,13]

summary_LONGITUDINAL_StrainRateEpi_T2P_seg3 = data_summary[80,14]

summary_LONGITUDINAL_StrainRateEpi_T2P_seg4 = data_summary[80,15]

summary_LONGITUDINAL_StrainRateEpi_T2P_seg5 = data_summary[80,16]

summary_LONGITUDINAL_StrainRateEpi_T2P_seg6 = data_summary[80,17]

summary_LONGITUDINAL_StrainRateEpi_T2P_ave = data_summary[80,18]

summary_LONGITUDINAL_StrainRateEpi_Pk_seg1 = data_summary[81,12]

summary_LONGITUDINAL_StrainRateEpi_Pk_seg2 = data_summary[81,13]

summary_LONGITUDINAL_StrainRateEpi_Pk_seg3 = data_summary[81,14]

summary_LONGITUDINAL_StrainRateEpi_Pk_seg4 = data_summary[81,15]

summary_LONGITUDINAL_StrainRateEpi_Pk_seg5 = data_summary[81,16]

summary_LONGITUDINAL_StrainRateEpi_Pk_seg6 = data_summary[81,17]

summary_LONGITUDINAL_StrainRateEpi_Pk_ave = data_summary[81,18]

summary_LONGITUDINAL_StrainRateEpi_PhasePercent_seg1 = data_summary[82,12]

summary_LONGITUDINAL_StrainRateEpi_PhasePercent_seg2 = data_summary[82,13]

summary_LONGITUDINAL_StrainRateEpi_PhasePercent_seg3 = data_summary[82,14]

summary_LONGITUDINAL_StrainRateEpi_PhasePercent_seg4 = data_summary[82,15]

summary_LONGITUDINAL_StrainRateEpi_PhasePercent_seg5 = data_summary[82,16]

summary_LONGITUDINAL_StrainRateEpi_PhasePercent_seg6 = data_summary[82,17]

summary_LONGITUDINAL_StrainRateEpi_PhasePercent_ave = data_summary[82,18]

summary_LONGITUDINAL_StrainRateEpi_Phase_seg1 = data_summary[83,12]

summary_LONGITUDINAL_StrainRateEpi_Phase_seg2 = data_summary[83,13]

summary_LONGITUDINAL_StrainRateEpi_Phase_seg3 = data_summary[83,14]

summary_LONGITUDINAL_StrainRateEpi_Phase_seg4 = data_summary[83,15]

summary_LONGITUDINAL_StrainRateEpi_Phase_seg5 = data_summary[83,16]

summary_LONGITUDINAL_StrainRateEpi_Phase_seg6 = data_summary[83,17]

summary_LONGITUDINAL_StrainRateEpi_Phase_ave = data_summary[83,18]

summary_LONGITUDINAL_StrainRateEpi_MaximumOpposingWallDelay = data_summary[84,12]

outTab=rbind(outTab,

cbind(i,

type,

summary_type,

summary_heart_rate,

summary_RADIAL_VelocityEndo_T2P_seg1,

summary_RADIAL_VelocityEndo_T2P_seg2,

summary_RADIAL_VelocityEndo_T2P_seg3,

summary_RADIAL_VelocityEndo_T2P_seg4,

summary_RADIAL_VelocityEndo_T2P_seg5,

summary_RADIAL_VelocityEndo_T2P_seg6,

summary_RADIAL_VelocityEndo_T2P_ave,

summary_RADIAL_VelocityEndo_Pk_seg1,

summary_RADIAL_VelocityEndo_Pk_seg2,

summary_RADIAL_VelocityEndo_Pk_seg3,

summary_RADIAL_VelocityEndo_Pk_seg4,

summary_RADIAL_VelocityEndo_Pk_seg5,

summary_RADIAL_VelocityEndo_Pk_seg6,

summary_RADIAL_VelocityEndo_Pk_ave,

summary_RADIAL_VelocityEndo_PhasePercent_seg1,

summary_RADIAL_VelocityEndo_PhasePercent_seg2,

summary_RADIAL_VelocityEndo_PhasePercent_seg3,

summary_RADIAL_VelocityEndo_PhasePercent_seg4,

summary_RADIAL_VelocityEndo_PhasePercent_seg5,

summary_RADIAL_VelocityEndo_PhasePercent_seg6,

summary_RADIAL_VelocityEndo_PhasePercent_ave,

summary_RADIAL_VelocityEndo_Phase_seg1,

summary_RADIAL_VelocityEndo_Phase_seg2,

summary_RADIAL_VelocityEndo_Phase_seg3,

summary_RADIAL_VelocityEndo_Phase_seg4,

summary_RADIAL_VelocityEndo_Phase_seg5,

summary_RADIAL_VelocityEndo_Phase_seg6,

summary_RADIAL_VelocityEndo_Phase_ave,

summary_RADIAL_VelocityEndo_MaximumOpposingWallDelay,

summary_RADIAL_VelocityEpi_T2P_seg1,

summary_RADIAL_VelocityEpi_T2P_seg2,

summary_RADIAL_VelocityEpi_T2P_seg3,

summary_RADIAL_VelocityEpi_T2P_seg4,

summary_RADIAL_VelocityEpi_T2P_seg5,

summary_RADIAL_VelocityEpi_T2P_seg6,

summary_RADIAL_VelocityEpi_T2P_ave,

summary_RADIAL_VelocityEpi_Pk_seg1,

summary_RADIAL_VelocityEpi_Pk_seg2,

summary_RADIAL_VelocityEpi_Pk_seg3,

summary_RADIAL_VelocityEpi_Pk_seg4,

summary_RADIAL_VelocityEpi_Pk_seg5,

summary_RADIAL_VelocityEpi_Pk_seg6,

summary_RADIAL_VelocityEpi_Pk_ave,

summary_RADIAL_VelocityEpi_PhasePercent_seg1,

summary_RADIAL_VelocityEpi_PhasePercent_seg2,

summary_RADIAL_VelocityEpi_PhasePercent_seg3,

summary_RADIAL_VelocityEpi_PhasePercent_seg4,

summary_RADIAL_VelocityEpi_PhasePercent_seg5,

summary_RADIAL_VelocityEpi_PhasePercent_seg6,

summary_RADIAL_VelocityEpi_PhasePercent_ave,

summary_RADIAL_VelocityEpi_Phase_seg1,

summary_RADIAL_VelocityEpi_Phase_seg2,

summary_RADIAL_VelocityEpi_Phase_seg3,

summary_RADIAL_VelocityEpi_Phase_seg4,

summary_RADIAL_VelocityEpi_Phase_seg5,

summary_RADIAL_VelocityEpi_Phase_seg6,

summary_RADIAL_VelocityEpi_Phase_ave,

summary_RADIAL_VelocityEpi_MaximumOpposingWallDelay,

summary_RADIAL_DisplacementEndo_T2P_seg1,

summary_RADIAL_DisplacementEndo_T2P_seg2,

summary_RADIAL_DisplacementEndo_T2P_seg3,

summary_RADIAL_DisplacementEndo_T2P_seg4,

summary_RADIAL_DisplacementEndo_T2P_seg5,

summary_RADIAL_DisplacementEndo_T2P_seg6,

summary_RADIAL_DisplacementEndo_T2P_ave,

summary_RADIAL_DisplacementEndo_Pk_seg1,

summary_RADIAL_DisplacementEndo_Pk_seg2,

summary_RADIAL_DisplacementEndo_Pk_seg3,

summary_RADIAL_DisplacementEndo_Pk_seg4,

summary_RADIAL_DisplacementEndo_Pk_seg5,

summary_RADIAL_DisplacementEndo_Pk_seg6,

summary_RADIAL_DisplacementEndo_Pk_ave,

summary_RADIAL_DisplacementEndo_PhasePercent_seg1,

summary_RADIAL_DisplacementEndo_PhasePercent_seg2,

summary_RADIAL_DisplacementEndo_PhasePercent_seg3,

summary_RADIAL_DisplacementEndo_PhasePercent_seg4,

summary_RADIAL_DisplacementEndo_PhasePercent_seg5,

summary_RADIAL_DisplacementEndo_PhasePercent_seg6,

summary_RADIAL_DisplacementEndo_PhasePercent_ave,

summary_RADIAL_DisplacementEndo_Phase_seg1,

summary_RADIAL_DisplacementEndo_Phase_seg2,

summary_RADIAL_DisplacementEndo_Phase_seg3,

summary_RADIAL_DisplacementEndo_Phase_seg4,

summary_RADIAL_DisplacementEndo_Phase_seg5,

summary_RADIAL_DisplacementEndo_Phase_seg6,

summary_RADIAL_DisplacementEndo_Phase_ave,

summary_RADIAL_DisplacementEndo_MaximumOpposingWallDelay,

summary_RADIAL_DisplacementEpi_T2P_seg1,

summary_RADIAL_DisplacementEpi_T2P_seg2,

summary_RADIAL_DisplacementEpi_T2P_seg3,

summary_RADIAL_DisplacementEpi_T2P_seg4,

summary_RADIAL_DisplacementEpi_T2P_seg5,

summary_RADIAL_DisplacementEpi_T2P_seg6,

summary_RADIAL_DisplacementEpi_T2P_ave,

summary_RADIAL_DisplacementEpi_Pk_seg1,

summary_RADIAL_DisplacementEpi_Pk_seg2,

summary_RADIAL_DisplacementEpi_Pk_seg3,

summary_RADIAL_DisplacementEpi_Pk_seg4,

summary_RADIAL_DisplacementEpi_Pk_seg5,

summary_RADIAL_DisplacementEpi_Pk_seg6,

summary_RADIAL_DisplacementEpi_Pk_ave,

summary_RADIAL_DisplacementEpi_PhasePercent_seg1,

summary_RADIAL_DisplacementEpi_PhasePercent_seg2,

summary_RADIAL_DisplacementEpi_PhasePercent_seg3,

summary_RADIAL_DisplacementEpi_PhasePercent_seg4,

summary_RADIAL_DisplacementEpi_PhasePercent_seg5,

summary_RADIAL_DisplacementEpi_PhasePercent_seg6,

summary_RADIAL_DisplacementEpi_PhasePercent_ave,

summary_RADIAL_DisplacementEpi_Phase_seg1,

summary_RADIAL_DisplacementEpi_Phase_seg2,

summary_RADIAL_DisplacementEpi_Phase_seg3,

summary_RADIAL_DisplacementEpi_Phase_seg4,

summary_RADIAL_DisplacementEpi_Phase_seg5,

summary_RADIAL_DisplacementEpi_Phase_seg6,

summary_RADIAL_DisplacementEpi_Phase_ave,

summary_RADIAL_DisplacementEpi_MaximumOpposingWallDelay,

summary_RADIAL_StrainEndo_T2P_seg1,

summary_RADIAL_StrainEndo_T2P_seg2,

summary_RADIAL_StrainEndo_T2P_seg3,

summary_RADIAL_StrainEndo_T2P_seg4,

summary_RADIAL_StrainEndo_T2P_seg5,

summary_RADIAL_StrainEndo_T2P_seg6,

summary_RADIAL_StrainEndo_T2P_ave,

summary_RADIAL_StrainEndo_Pk_seg1,

summary_RADIAL_StrainEndo_Pk_seg2,

summary_RADIAL_StrainEndo_Pk_seg3,

summary_RADIAL_StrainEndo_Pk_seg4,

summary_RADIAL_StrainEndo_Pk_seg5,

summary_RADIAL_StrainEndo_Pk_seg6,

summary_RADIAL_StrainEndo_Pk_ave,

summary_RADIAL_StrainEndo_PhasePercent_seg1,

summary_RADIAL_StrainEndo_PhasePercent_seg2,

summary_RADIAL_StrainEndo_PhasePercent_seg3,

summary_RADIAL_StrainEndo_PhasePercent_seg4,

summary_RADIAL_StrainEndo_PhasePercent_seg5,

summary_RADIAL_StrainEndo_PhasePercent_seg6,

summary_RADIAL_StrainEndo_PhasePercent_ave,

summary_RADIAL_StrainEndo_Phase_seg1,

summary_RADIAL_StrainEndo_Phase_seg2,

summary_RADIAL_StrainEndo_Phase_seg3,

summary_RADIAL_StrainEndo_Phase_seg4,

summary_RADIAL_StrainEndo_Phase_seg5,

summary_RADIAL_StrainEndo_Phase_seg6,

summary_RADIAL_StrainEndo_Phase_ave,

summary_RADIAL_StrainEndo_MaximumOpposingWallDelay,

summary_RADIAL_StrainEpi_T2P_seg1,

summary_RADIAL_StrainEpi_T2P_seg2,

summary_RADIAL_StrainEpi_T2P_seg3,

summary_RADIAL_StrainEpi_T2P_seg4,

summary_RADIAL_StrainEpi_T2P_seg5,

summary_RADIAL_StrainEpi_T2P_seg6,

summary_RADIAL_StrainEpi_T2P_ave,

summary_RADIAL_StrainEpi_Pk_seg1,

summary_RADIAL_StrainEpi_Pk_seg2,

summary_RADIAL_StrainEpi_Pk_seg3,

summary_RADIAL_StrainEpi_Pk_seg4,

summary_RADIAL_StrainEpi_Pk_seg5,

summary_RADIAL_StrainEpi_Pk_seg6,

summary_RADIAL_StrainEpi_Pk_ave,

summary_RADIAL_StrainEpi_PhasePercent_seg1,

summary_RADIAL_StrainEpi_PhasePercent_seg2,

summary_RADIAL_StrainEpi_PhasePercent_seg3,

summary_RADIAL_StrainEpi_PhasePercent_seg4,

summary_RADIAL_StrainEpi_PhasePercent_seg5,

summary_RADIAL_StrainEpi_PhasePercent_seg6,

summary_RADIAL_StrainEpi_PhasePercent_ave,

summary_RADIAL_StrainEpi_Phase_seg1,

summary_RADIAL_StrainEpi_Phase_seg2,

summary_RADIAL_StrainEpi_Phase_seg3,

summary_RADIAL_StrainEpi_Phase_seg4,

summary_RADIAL_StrainEpi_Phase_seg5,

summary_RADIAL_StrainEpi_Phase_seg6,

summary_RADIAL_StrainEpi_Phase_ave,

summary_RADIAL_StrainEpi_MaximumOpposingWallDelay,

summary_RADIAL_StrainRateEndo_T2P_seg1,

summary_RADIAL_StrainRateEndo_T2P_seg2,

summary_RADIAL_StrainRateEndo_T2P_seg3,

summary_RADIAL_StrainRateEndo_T2P_seg4,

summary_RADIAL_StrainRateEndo_T2P_seg5,

summary_RADIAL_StrainRateEndo_T2P_seg6,

summary_RADIAL_StrainRateEndo_T2P_ave,

summary_RADIAL_StrainRateEndo_Pk_seg1,

summary_RADIAL_StrainRateEndo_Pk_seg2,

summary_RADIAL_StrainRateEndo_Pk_seg3,

summary_RADIAL_StrainRateEndo_Pk_seg4,

summary_RADIAL_StrainRateEndo_Pk_seg5,

summary_RADIAL_StrainRateEndo_Pk_seg6,

summary_RADIAL_StrainRateEndo_Pk_ave,

summary_RADIAL_StrainRateEndo_PhasePercent_seg1,

summary_RADIAL_StrainRateEndo_PhasePercent_seg2,

summary_RADIAL_StrainRateEndo_PhasePercent_seg3,

summary_RADIAL_StrainRateEndo_PhasePercent_seg4,

summary_RADIAL_StrainRateEndo_PhasePercent_seg5,

summary_RADIAL_StrainRateEndo_PhasePercent_seg6,

summary_RADIAL_StrainRateEndo_PhasePercent_ave,

summary_RADIAL_StrainRateEndo_Phase_seg1,

summary_RADIAL_StrainRateEndo_Phase_seg2,

summary_RADIAL_StrainRateEndo_Phase_seg3,

summary_RADIAL_StrainRateEndo_PhaseP_seg4,

summary_RADIAL_StrainRateEndo_Phase_seg5,

summary_RADIAL_StrainRateEndo_Phase_seg6,

summary_RADIAL_StrainRateEndo_Phase_ave,

summary_RADIAL_StrainRateEndo_MaximumOpposingWallDelay,

summary_RADIAL_StrainRateEpi_T2P_seg1,

summary_RADIAL_StrainRateEpi_T2P_seg2,

summary_RADIAL_StrainRateEpi_T2P_seg3,

summary_RADIAL_StrainRateEpi_T2P_seg4,

summary_RADIAL_StrainRateEpi_T2P_seg5,

summary_RADIAL_StrainRateEpi_T2P_seg6,

summary_RADIAL_StrainRateEpi_T2P_ave,

summary_RADIAL_StrainRateEpi_Pk_seg1,

summary_RADIAL_StrainRateEpi_Pk_seg2,

summary_RADIAL_StrainRateEpi_Pk_seg3,

summary_RADIAL_StrainRateEpi_Pk_seg4,

summary_RADIAL_StrainRateEpi_Pk_seg5,

summary_RADIAL_StrainRateEpi_Pk_seg6,

summary_RADIAL_StrainRateEpi_Pk_ave,

summary_RADIAL_StrainRateEpi_PhasePercent_seg1,

summary_RADIAL_StrainRateEpi_PhasePercent_seg2,

summary_RADIAL_StrainRateEpi_PhasePercent_seg3,

summary_RADIAL_StrainRateEpi_PhasePercent_seg4,

summary_RADIAL_StrainRateEpi_PhasePercent_seg5,

summary_RADIAL_StrainRateEpi_PhasePercent_seg6,

summary_RADIAL_StrainRateEpi_PhasePercent_ave,

summary_RADIAL_StrainRateEpi_Phase_seg1,

summary_RADIAL_StrainRateEpi_Phase_seg2,

summary_RADIAL_StrainRateEpi_Phase_seg3,

summary_RADIAL_StrainRateEpi_Phase_seg4,

summary_RADIAL_StrainRateEpi_Phase_seg5,

summary_RADIAL_StrainRateEpi_Phase_seg6,

summary_RADIAL_StrainRateEpi_Phase_ave,

summary_RADIAL_StrainRateEpi_MaximumOpposingWallDelay,

summary_LONGITUDINAL_VelocityEndo_T2P_seg1,

summary_LONGITUDINAL_VelocityEndo_T2P_seg2,

summary_LONGITUDINAL_VelocityEndo_T2P_seg3,

summary_LONGITUDINAL_VelocityEndo_T2P_seg4,

summary_LONGITUDINAL_VelocityEndo_T2P_seg5,

summary_LONGITUDINAL_VelocityEndo_T2P_seg6,

summary_LONGITUDINAL_VelocityEndo_T2P_ave,

summary_LONGITUDINAL_VelocityEndo_Pk_seg1,

summary_LONGITUDINAL_VelocityEndo_Pk_seg2,

summary_LONGITUDINAL_VelocityEndo_Pk_seg3,

summary_LONGITUDINAL_VelocityEndo_Pk_seg4,

summary_LONGITUDINAL_VelocityEndo_Pk_seg5,

summary_LONGITUDINAL_VelocityEndo_Pk_seg6,

summary_LONGITUDINAL_VelocityEndo_Pk_ave,

summary_LONGITUDINAL_VelocityEndo_PhasePercent_seg1,

summary_LONGITUDINAL_VelocityEndo_PhasePercent_seg2,

summary_LONGITUDINAL_VelocityEndo_PhasePercent_seg3,

summary_LONGITUDINAL_VelocityEndo_PhasePercent_seg4,

summary_LONGITUDINAL_VelocityEndo_PhasePercent_seg5,

summary_LONGITUDINAL_VelocityEndo_PhasePercent_seg6,

summary_LONGITUDINAL_VelocityEndo_PhasePercent_ave,

summary_LONGITUDINAL_VelocityEndo_Phase_seg1,

summary_LONGITUDINAL_VelocityEndo_Phase_seg2,

summary_LONGITUDINAL_VelocityEndo_Phase_seg3,

summary_LONGITUDINAL_VelocityEndo_Phase_seg4,

summary_LONGITUDINAL_VelocityEndo_Phase_seg5,

summary_LONGITUDINAL_VelocityEndo_Phase_seg6,

summary_LONGITUDINAL_VelocityEndo_Phase_ave,

summary_LONGITUDINAL_VelocityEndo_MaximumOpposingWallDelay,

summary_LONGITUDINAL_VelocityEpi_T2P_seg1,

summary_LONGITUDINAL_VelocityEpi_T2P_seg2,

summary_LONGITUDINAL_VelocityEpi_T2P_seg3,

summary_LONGITUDINAL_VelocityEpi_T2P_seg4,

summary_LONGITUDINAL_VelocityEpi_T2P_seg5,

summary_LONGITUDINAL_VelocityEpi_T2P_seg6,

summary_LONGITUDINAL_VelocityEpi_T2P_ave,

summary_LONGITUDINAL_VelocityEpi_Pk_seg1,

summary_LONGITUDINAL_VelocityEpi_Pk_seg2,

summary_LONGITUDINAL_VelocityEpi_Pk_seg3,

summary_LONGITUDINAL_VelocityEpi_Pk_seg4,

summary_LONGITUDINAL_VelocityEpi_Pk_seg5,

summary_LONGITUDINAL_VelocityEpi_Pk_seg6,

summary_LONGITUDINAL_VelocityEpi_Pk_ave,

summary_LONGITUDINAL_VelocityEpi_PhasePercent_seg1,

summary_LONGITUDINAL_VelocityEpi_PhasePercent_seg2,

summary_LONGITUDINAL_VelocityEpi_PhasePercent_seg3,

summary_LONGITUDINAL_VelocityEpi_PhasePercent_seg4,

summary_LONGITUDINAL_VelocityEpi_PhasePercent_seg5,

summary_LONGITUDINAL_VelocityEpi_PhasePercent_seg6,

summary_LONGITUDINAL_VelocityEpi_PhasePercent_ave,

summary_LONGITUDINAL_VelocityEpi_Phase_seg1,

summary_LONGITUDINAL_VelocityEpi_Phase_seg2,

summary_LONGITUDINAL_VelocityEpi_Phase_seg3,

summary_LONGITUDINAL_VelocityEpi_Phase_seg4,

summary_LONGITUDINAL_VelocityEpi_Phase_seg5,

summary_LONGITUDINAL_VelocityEpi_Phase_seg6,

summary_LONGITUDINAL_VelocityEpi_Phase_ave,

summary_LONGITUDINAL_VelocityEpi_MaximumOpposingWallDelay,

summary_LONGITUDINAL_DisplacementEndo_T2P_seg1,

summary_LONGITUDINAL_DisplacementEndo_T2P_seg2,

summary_LONGITUDINAL_DisplacementEndo_T2P_seg3,

summary_LONGITUDINAL_DisplacementEndo_T2P_seg4,

summary_LONGITUDINAL_DisplacementEndo_T2P_seg5,

summary_LONGITUDINAL_DisplacementEndo_T2P_seg6,

summary_LONGITUDINAL_DisplacementEndo_T2P_ave,

summary_LONGITUDINAL_DisplacementEndo_Pk_seg1,

summary_LONGITUDINAL_DisplacementEndo_Pk_seg2,

summary_LONGITUDINAL_DisplacementEndo_Pk_seg3,

summary_LONGITUDINAL_DisplacementEndo_Pk_seg4,

summary_LONGITUDINAL_DisplacementEndo_Pk_seg5,

summary_LONGITUDINAL_DisplacementEndo_Pk_seg6,

summary_LONGITUDINAL_DisplacementEndo_Pk_ave,

summary_LONGITUDINAL_DisplacementEndo_PhasePercent_seg1,

summary_LONGITUDINAL_DisplacementEndo_PhasePercent_seg2,

summary_LONGITUDINAL_DisplacementEndo_PhasePercent_seg3,

summary_LONGITUDINAL_DisplacementEndo_PhasePercent_seg4,

summary_LONGITUDINAL_DisplacementEndo_PhasePercent_seg5,

summary_LONGITUDINAL_DisplacementEndo_PhasePercent_seg6,

summary_LONGITUDINAL_DisplacementEndo_PhasePercent_ave,

summary_LONGITUDINAL_DisplacementEndo_Phase_seg1,

summary_LONGITUDINAL_DisplacementEndo_Phase_seg2,

summary_LONGITUDINAL_DisplacementEndo_Phase_seg3,

summary_LONGITUDINAL_DisplacementEndo_Phase_seg4,

summary_LONGITUDINAL_DisplacementEndo_Phase_seg5,

summary_LONGITUDINAL_DisplacementEndo_Phase_seg6,

summary_LONGITUDINAL_DisplacementEndo_Phase_ave,

summary_LONGITUDINAL_DisplacementEndo_MaximumOpposingWallDelay,

summary_LONGITUDINAL_DisplacementEpi_T2P_seg1,

summary_LONGITUDINAL_DisplacementEpi_T2P_seg2,

summary_LONGITUDINAL_DisplacementEpi_T2P_seg3,

summary_LONGITUDINAL_DisplacementEpi_T2P_seg4,

summary_LONGITUDINAL_DisplacementEpi_T2P_seg5,

summary_LONGITUDINAL_DisplacementEpi_T2P_seg6,

summary_LONGITUDINAL_DisplacementEpi_T2P_ave,

summary_LONGITUDINAL_DisplacementEpi_Pk_seg1,

summary_LONGITUDINAL_DisplacementEpi_Pk_seg2,

summary_LONGITUDINAL_DisplacementEpi_Pk_seg3,

summary_LONGITUDINAL_DisplacementEpi_Pk_seg4,

summary_LONGITUDINAL_DisplacementEpi_Pk_seg5,

summary_LONGITUDINAL_DisplacementEpi_Pk_seg6,

summary_LONGITUDINAL_DisplacementEpi_Pk_ave,

summary_LONGITUDINAL_DisplacementEpi_PhasePercent_seg1,

summary_LONGITUDINAL_DisplacementEpi_PhasePercent_seg2,

summary_LONGITUDINAL_DisplacementEpi_PhasePercent_seg3,

summary_LONGITUDINAL_DisplacementEpi_PhasePercent_seg4,

summary_LONGITUDINAL_DisplacementEpi_PhasePercent_seg5,

summary_LONGITUDINAL_DisplacementEpi_PhasePercent_seg6,

summary_LONGITUDINAL_DisplacementEpi_PhasePercent_ave,

summary_LONGITUDINAL_DisplacementEpi_Phase_seg1,

summary_LONGITUDINAL_DisplacementEpi_Phase_seg2,

summary_LONGITUDINAL_DisplacementEpi_Phase_seg3,

summary_LONGITUDINAL_DisplacementEpi_Phase_seg4,

summary_LONGITUDINAL_DisplacementEpi_Phase_seg5,

summary_LONGITUDINAL_DisplacementEpi_Phase_seg6,

summary_LONGITUDINAL_DisplacementEpi_Phase_ave,

summary_LONGITUDINAL_DisplacementEpi_MaximumOpposingWallDelay,

summary_LONGITUDINAL_StrainEndo_T2P_seg1,

summary_LONGITUDINAL_StrainEndo_T2P_seg2,

summary_LONGITUDINAL_StrainEndo_T2P_seg3,

summary_LONGITUDINAL_StrainEndo_T2P_seg4,

summary_LONGITUDINAL_StrainEndo_T2P_seg5,

summary_LONGITUDINAL_StrainEndo_T2P_seg6,

summary_LONGITUDINAL_StrainEndo_T2P_ave,

summary_LONGITUDINAL_StrainEndo_Pk_seg1,

summary_LONGITUDINAL_StrainEndo_Pk_seg2,

summary_LONGITUDINAL_StrainEndo_Pk_seg3,

summary_LONGITUDINAL_StrainEndo_Pk_seg4,

summary_LONGITUDINAL_StrainEndo_Pk_seg5,

summary_LONGITUDINAL_StrainEndo_Pk_seg6,

summary_LONGITUDINAL_StrainEndo_Pk_ave,

summary_LONGITUDINAL_StrainEndo_PhasePercent_seg1,

summary_LONGITUDINAL_StrainEndo_PhasePercent_seg2,

summary_LONGITUDINAL_StrainEndo_PhasePercent_seg3,

summary_LONGITUDINAL_StrainEndo_PhasePercent_seg4,

summary_LONGITUDINAL_StrainEndo_PhasePercent_seg5,

summary_LONGITUDINAL_StrainEndo_PhasePercent_seg6,

summary_LONGITUDINAL_StrainEndo_PhasePercent_ave,

summary_LONGITUDINAL_StrainEndo_Phase_seg1,

summary_LONGITUDINAL_StrainEndo_Phase_seg2,

summary_LONGITUDINAL_StrainEndo_Phase_seg3,

summary_LONGITUDINAL_StrainEndo_Phase_seg4,

summary_LONGITUDINAL_StrainEndo_Phase_seg5,

summary_LONGITUDINAL_StrainEndo_Phase_seg6,

summary_LONGITUDINAL_StrainEndo_Phase_ave,

summary_LONGITUDINAL_StrainEndo_MaximumOpposingWallDelay,

summary_LONGITUDINAL_StrainEpi_T2P_seg1,

summary_LONGITUDINAL_StrainEpi_T2P_seg2,

summary_LONGITUDINAL_StrainEpi_T2P_seg3,

summary_LONGITUDINAL_StrainEpi_T2P_seg4,

summary_LONGITUDINAL_StrainEpi_T2P_seg5,

summary_LONGITUDINAL_StrainEpi_T2P_seg6,

summary_LONGITUDINAL_StrainEpi_T2P_ave,

summary_LONGITUDINAL_StrainEpi_Pk_seg1,

summary_LONGITUDINAL_StrainEpi_Pk_seg2,

summary_LONGITUDINAL_StrainEpi_Pk_seg3,

summary_LONGITUDINAL_StrainEpi_Pk_seg4,

summary_LONGITUDINAL_StrainEpi_Pk_seg5,

summary_LONGITUDINAL_StrainEpi_Pk_seg6,

summary_LONGITUDINAL_StrainEpi_Pk_ave,

summary_LONGITUDINAL_StrainEpi_PhasePercent_seg1,

summary_LONGITUDINAL_StrainEpi_PhasePercent_seg2,

summary_LONGITUDINAL_StrainEpi_PhasePercent_seg3,

summary_LONGITUDINAL_StrainEpi_PhasePercent_seg4,

summary_LONGITUDINAL_StrainEpi_PhasePercent_seg5,

summary_LONGITUDINAL_StrainEpi_PhasePercent_seg6,

summary_LONGITUDINAL_StrainEpi_PhasePercent_ave,

summary_LONGITUDINAL_StrainEpi_Phase_seg1,

summary_LONGITUDINAL_StrainEpi_Phase_seg2,

summary_LONGITUDINAL_StrainEpi_Phase_seg3,

summary_LONGITUDINAL_StrainEpi_Phase_seg4,

summary_LONGITUDINAL_StrainEpi_Phase_seg5,

summary_LONGITUDINAL_StrainEpi_Phase_seg6,

summary_LONGITUDINAL_StrainEpi_Phase_ave,

summary_LONGITUDINAL_StrainEpi_MaximumOpposingWallDelay,

summary_LONGITUDINAL_StrainRateEndo_T2P_seg1,

summary_LONGITUDINAL_StrainRateEndo_T2P_seg2,

summary_LONGITUDINAL_StrainRateEndo_T2P_seg3,

summary_LONGITUDINAL_StrainRateEndo_T2P_seg4,

summary_LONGITUDINAL_StrainRateEndo_T2P_seg5,

summary_LONGITUDINAL_StrainRateEndo_T2P_seg6,

summary_LONGITUDINAL_StrainRateEndo_T2P_ave,

summary_LONGITUDINAL_StrainRateEndo_Pk_seg1,

summary_LONGITUDINAL_StrainRateEndo_Pk_seg2,

summary_LONGITUDINAL_StrainRateEndo_Pk_seg3,

summary_LONGITUDINAL_StrainRateEndo_Pk_seg4,

summary_LONGITUDINAL_StrainRateEndo_Pk_seg5,

summary_LONGITUDINAL_StrainRateEndo_Pk_seg6,

summary_LONGITUDINAL_StrainRateEndo_Pk_ave,

summary_LONGITUDINAL_StrainRateEndo_PhasePercent_seg1,

summary_LONGITUDINAL_StrainRateEndo_PhasePercent_seg2,

summary_LONGITUDINAL_StrainRateEndo_PhasePercent_seg3,

summary_LONGITUDINAL_StrainRateEndo_PhasePercent_seg4,

summary_LONGITUDINAL_StrainRateEndo_PhasePercent_seg5,

summary_LONGITUDINAL_StrainRateEndo_PhasePercent_seg6,

summary_LONGITUDINAL_StrainRateEndo_PhasePercent_ave,

summary_LONGITUDINAL_StrainRateEndo_Phase_seg1,

summary_LONGITUDINAL_StrainRateEndo_Phase_seg2,

summary_LONGITUDINAL_StrainRateEndo_Phase_seg3,

summary_LONGITUDINAL_StrainRateEndo_Phase_seg4,

summary_LONGITUDINAL_StrainRateEndo_Phase_seg5,

summary_LONGITUDINAL_StrainRateEndo_Phase_seg6,

summary_LONGITUDINAL_StrainRateEndo_Phase_ave,

summary_LONGITUDINAL_StrainRateEndo_MaximumOpposingWallDelay,

summary_LONGITUDINAL_StrainRateEpi_T2P_seg1,

summary_LONGITUDINAL_StrainRateEpi_T2P_seg2,

summary_LONGITUDINAL_StrainRateEpi_T2P_seg3,

summary_LONGITUDINAL_StrainRateEpi_T2P_seg4,

summary_LONGITUDINAL_StrainRateEpi_T2P_seg5,

summary_LONGITUDINAL_StrainRateEpi_T2P_seg6,

summary_LONGITUDINAL_StrainRateEpi_T2P_ave,

summary_LONGITUDINAL_StrainRateEpi_Pk_seg1,

summary_LONGITUDINAL_StrainRateEpi_Pk_seg2,

summary_LONGITUDINAL_StrainRateEpi_Pk_seg3,

summary_LONGITUDINAL_StrainRateEpi_Pk_seg4,

summary_LONGITUDINAL_StrainRateEpi_Pk_seg5,

summary_LONGITUDINAL_StrainRateEpi_Pk_seg6,

summary_LONGITUDINAL_StrainRateEpi_Pk_ave,

summary_LONGITUDINAL_StrainRateEpi_PhasePercent_seg1,

summary_LONGITUDINAL_StrainRateEpi_PhasePercent_seg2,

summary_LONGITUDINAL_StrainRateEpi_PhasePercent_seg3,

summary_LONGITUDINAL_StrainRateEpi_PhasePercent_seg4,

summary_LONGITUDINAL_StrainRateEpi_PhasePercent_seg5,

summary_LONGITUDINAL_StrainRateEpi_PhasePercent_seg6,

summary_LONGITUDINAL_StrainRateEpi_PhasePercent_ave,

summary_LONGITUDINAL_StrainRateEpi_Phase_seg1,

summary_LONGITUDINAL_StrainRateEpi_Phase_seg2,

summary_LONGITUDINAL_StrainRateEpi_Phase_seg3,

summary_LONGITUDINAL_StrainRateEpi_Phase_seg4,

summary_LONGITUDINAL_StrainRateEpi_Phase_seg5,

summary_LONGITUDINAL_StrainRateEpi_Phase_seg6,

summary_LONGITUDINAL_StrainRateEpi_Phase_ave,

summary_LONGITUDINAL_StrainRateEpi_MaximumOpposingWallDelay,

Radial_Velocity_Endo_seg001_a_min,

Radial_Velocity_Endo_seg001_a_max,

Radial_Velocity_Endo_seg002_a_min,

Radial_Velocity_Endo_seg002_a_max,

Radial_Velocity_Endo_seg003_a_min,

Radial_Velocity_Endo_seg003_a_max,

Radial_Velocity_Endo_seg004_a_min,

Radial_Velocity_Endo_seg004_a_max,

Radial_Velocity_Endo_seg005_a_min,

Radial_Velocity_Endo_seg005_a_max,

Radial_Velocity_Endo_seg006_a_min,

Radial_Velocity_Endo_seg006_a_max,

Average_values_per_frame_Radial_Velocity_Endo_a_min,

Average_values_per_frame_Radial_Velocity_Endo_a_max,

Longitudinal_Velocity_Endo_seg001_a_min,

Longitudinal_Velocity_Endo_seg001_a_max,

Longitudinal_Velocity_Endo_seg002_a_min,

Longitudinal_Velocity_Endo_seg002_a_max,

Longitudinal_Velocity_Endo_seg003_a_min,

Longitudinal_Velocity_Endo_seg003_a_max,

Longitudinal_Velocity_Endo_seg004_a_min,

Longitudinal_Velocity_Endo_seg004_a_max,

Longitudinal_Velocity_Endo_seg005_a_min,

Longitudinal_Velocity_Endo_seg005_a_max,

Longitudinal_Velocity_Endo_seg006_a_min,

Longitudinal_Velocity_Endo_seg006_a_max,

Average_values_per_frame_Longitudinal_Velocity_Endo_a_min,

Average_values_per_frame_Longitudinal_Velocity_Endo_a_max,

Radial_Velocity_Epi_seg001_a_min,

Radial_Velocity_Epi_seg001_a_max,

Radial_Velocity_Epi_seg002_a_min,

Radial_Velocity_Epi_seg002_a_max,

Radial_Velocity_Epi_seg003_a_min,

Radial_Velocity_Epi_seg003_a_max,

Radial_Velocity_Epi_seg004_a_min,

Radial_Velocity_Epi_seg004_a_max,

Radial_Velocity_Epi_seg005_a_min,

Radial_Velocity_Epi_seg005_a_max,

Radial_Velocity_Epi_seg006_a_min,

Radial_Velocity_Epi_seg006_a_max,

Average_values_per_frame_Radial_Velocity_Epi_a_min,

Average_values_per_frame_Radial_Velocity_Epi_a_max,

Longitudinal_Velocity_Epi_seg001_a_min,

Longitudinal_Velocity_Epi_seg001_a_max,

Longitudinal_Velocity_Epi_seg002_a_min,

Longitudinal_Velocity_Epi_seg002_a_max,

Longitudinal_Velocity_Epi_seg003_a_min,

Longitudinal_Velocity_Epi_seg003_a_max,

Longitudinal_Velocity_Epi_seg004_a_min,

Longitudinal_Velocity_Epi_seg004_a_max,

Longitudinal_Velocity_Epi_seg005_a_min,

Longitudinal_Velocity_Epi_seg005_a_max,

Longitudinal_Velocity_Epi_seg006_a_min,

Longitudinal_Velocity_Epi_seg006_a_max,

Average_values_per_frame_Longitudinal_Velocity_Epi_a_min,

Average_values_per_frame_Longitudinal_Velocity_Epi_a_max,

Radial_Displacement_Endo_seg001_a_min,

Radial_Displacement_Endo_seg001_a_max,

Radial_Displacement_Endo_seg002_a_min,

Radial_Displacement_Endo_seg002_a_max,

Radial_Displacement_Endo_seg003_a_min,

Radial_Displacement_Endo_seg003_a_max,

Radial_Displacement_Endo_seg004_a_min,

Radial_Displacement_Endo_seg004_a_max,

Radial_Displacement_Endo_seg005_a_min,

Radial_Displacement_Endo_seg005_a_max,

Radial_Displacement_Endo_seg006_a_min,

Radial_Displacement_Endo_seg006_a_max,

Average_values_per_frame_Radial_Displacement_Endo_a_min,

Average_values_per_frame_Radial_Displacement_Endo_a_max,

Longitudinal_Displacement_Endo_seg001_a_min,

Longitudinal_Displacement_Endo_seg001_a_max,

Longitudinal_Displacement_Endo_seg002_a_min,

Longitudinal_Displacement_Endo_seg002_a_max,

Longitudinal_Displacement_Endo_seg003_a_min,

Longitudinal_Displacement_Endo_seg003_a_max,

Longitudinal_Displacement_Endo_seg004_a_min,

Longitudinal_Displacement_Endo_seg004_a_max,

Longitudinal_Displacement_Endo_seg005_a_min,

Longitudinal_Displacement_Endo_seg005_a_max,

Longitudinal_Displacement_Endo_seg006_a_min,

Longitudinal_Displacement_Endo_seg006_a_max,

Average_values_per_frame_Longitudinal_Displacement_Endo_a_min,

Average_values_per_frame_Longitudinal_Displacement_Endo_a_max,

Radial_Displacement_Epi_seg001_a_min,

Radial_Displacement_Epi_seg001_a_max,

Radial_Displacement_Epi_seg002_a_min,

Radial_Displacement_Epi_seg002_a_max,

Radial_Displacement_Epi_seg003_a_min,

Radial_Displacement_Epi_seg003_a_max,

Radial_Displacement_Epi_seg004_a_min,

Radial_Displacement_Epi_seg004_a_max,

Radial_Displacement_Epi_seg005_a_min,

Radial_Displacement_Epi_seg005_a_max,

Radial_Displacement_Epi_seg006_a_min,

Radial_Displacement_Epi_seg006_a_max,

Average_values_per_frame_Radial_Displacement_Epi_a_min,

Average_values_per_frame_Radial_Displacement_Epi_a_max,

Longitudinal_Displacement_Epi_seg001_a_min,

Longitudinal_Displacement_Epi_seg001_a_max,

Longitudinal_Displacement_Epi_seg002_a_min,

Longitudinal_Displacement_Epi_seg002_a_max,

Longitudinal_Displacement_Epi_seg003_a_min,

Longitudinal_Displacement_Epi_seg003_a_max,

Longitudinal_Displacement_Epi_seg004_a_min,

Longitudinal_Displacement_Epi_seg004_a_max,

Longitudinal_Displacement_Epi_seg005_a_min,

Longitudinal_Displacement_Epi_seg005_a_max,

Longitudinal_Displacement_Epi_seg006_a_min,

Longitudinal_Displacement_Epi_seg006_a_max,

Average_values_per_frame_Longitudinal_Displacement_Epi_a_min,

Average_values_per_frame_Longitudinal_Displacement_Epi_a_max,

Radial_Strain_Endo_seg001_a_min,

Radial_Strain_Endo_seg001_a_max,

Radial_Strain_Endo_seg002_a_min,

Radial_Strain_Endo_seg002_a_max,

Radial_Strain_Endo_seg003_a_min,

Radial_Strain_Endo_seg003_a_max,

Radial_Strain_Endo_seg004_a_min,

Radial_Strain_Endo_seg004_a_max,

Radial_Strain_Endo_seg005_a_min,

Radial_Strain_Endo_seg005_a_max,

Radial_Strain_Endo_seg006_a_min,

Radial_Strain_Endo_seg006_a_max,

Average_values_per_frame_Radial_Strain_Endo_a_min,

Average_values_per_frame_Radial_Strain_Endo_a_max,

Longitudinal_Strain_Endo_seg001_a_min,

Longitudinal_Strain_Endo_seg001_a_max,

Longitudinal_Strain_Endo_seg002_a_min,

Longitudinal_Strain_Endo_seg002_a_max,

Longitudinal_Strain_Endo_seg003_a_min,

Longitudinal_Strain_Endo_seg003_a_max,

Longitudinal_Strain_Endo_seg004_a_min,

Longitudinal_Strain_Endo_seg004_a_max,

Longitudinal_Strain_Endo_seg005_a_min,

Longitudinal_Strain_Endo_seg005_a_max,

Longitudinal_Strain_Endo_seg006_a_min,

Longitudinal_Strain_Endo_seg006_a_max,

Average_values_per_frame_Longitudinal_Strain_Endo_a_min,

Average_values_per_frame_Longitudinal_Strain_Endo_a_max,

Radial_Strain_Epi_seg001_a_min,

Radial_Strain_Epi_seg001_a_max,

Radial_Strain_Epi_seg002_a_min,

Radial_Strain_Epi_seg002_a_max,

Radial_Strain_Epi_seg003_a_min,

Radial_Strain_Epi_seg003_a_max,

Radial_Strain_Epi_seg004_a_min,

Radial_Strain_Epi_seg004_a_max,

Radial_Strain_Epi_seg005_a_min,

Radial_Strain_Epi_seg005_a_max,

Radial_Strain_Epi_seg006_a_min,

Radial_Strain_Epi_seg006_a_max,

Average_values_per_frame_Radial_Strain_Epi_a_min,

Average_values_per_frame_Radial_Strain_Epi_a_max,

Longitudinal_Strain_Epi_seg001_a_min,

Longitudinal_Strain_Epi_seg001_a_max,

Longitudinal_Strain_Epi_seg002_a_min,

Longitudinal_Strain_Epi_seg002_a_max,

Longitudinal_Strain_Epi_seg003_a_min,

Longitudinal_Strain_Epi_seg003_a_max,

Longitudinal_Strain_Epi_seg004_a_min,

Longitudinal_Strain_Epi_seg004_a_max,

Longitudinal_Strain_Epi_seg005_a_min,

Longitudinal_Strain_Epi_seg005_a_max,

Longitudinal_Strain_Epi_seg006_a_min,

Longitudinal_Strain_Epi_seg006_a_max,

Average_values_per_frame_Longitudinal_Strain_Epi_a_min,

Average_values_per_frame_Longitudinal_Strain_Epi_a_max,

Radial_Strain_Rate_Endo_seg001_a_min,

Radial_Strain_Rate_Endo_seg001_a_max,

Radial_Strain_Rate_Endo_seg002_a_min,

Radial_Strain_Rate_Endo_seg002_a_max,

Radial_Strain_Rate_Endo_seg003_a_min,

Radial_Strain_Rate_Endo_seg003_a_max,

Radial_Strain_Rate_Endo_seg004_a_min,

Radial_Strain_Rate_Endo_seg004_a_max,

Radial_Strain_Rate_Endo_seg005_a_min,

Radial_Strain_Rate_Endo_seg005_a_max,

Radial_Strain_Rate_Endo_seg006_a_min,

Radial_Strain_Rate_Endo_seg006_a_max,

Average_values_per_frame_Radial_Strain_Rate_Endo_a_min,

Average_values_per_frame_Radial_Strain_Rate_Endo_a_max,

Longitudinal_Strain_Rate_Endo_seg001_a_min,

Longitudinal_Strain_Rate_Endo_seg001_a_max,

Longitudinal_Strain_Rate_Endo_seg002_a_min,

Longitudinal_Strain_Rate_Endo_seg002_a_max,

Longitudinal_Strain_Rate_Endo_seg003_a_min,

Longitudinal_Strain_Rate_Endo_seg003_a_max,

Longitudinal_Strain_Rate_Endo_seg004_a_min,

Longitudinal_Strain_Rate_Endo_seg004_a_max,

Longitudinal_Strain_Rate_Endo_seg005_a_min,

Longitudinal_Strain_Rate_Endo_seg005_a_max,

Longitudinal_Strain_Rate_Endo_seg006_a_min,

Longitudinal_Strain_Rate_Endo_seg006_a_max,

Average_values_per_frame_Longitudinal_Strain_Rate_Endo_a_min,

Average_values_per_frame_Longitudinal_Strain_Rate_Endo_a_max,

Radial_Strain_Rate_Epi_seg001_a_min,

Radial_Strain_Rate_Epi_seg001_a_max,

Radial_Strain_Rate_Epi_seg002_a_min,

Radial_Strain_Rate_Epi_seg002_a_max,

Radial_Strain_Rate_Epi_seg003_a_min,

Radial_Strain_Rate_Epi_seg003_a_max,

Radial_Strain_Rate_Epi_seg004_a_min,

Radial_Strain_Rate_Epi_seg004_a_max,

Radial_Strain_Rate_Epi_seg005_a_min,

Radial_Strain_Rate_Epi_seg005_a_max,

Radial_Strain_Rate_Epi_seg006_a_min,

Radial_Strain_Rate_Epi_seg006_a_max,

Average_values_per_frame_Radial_Strain_Rate_Epi_a_min,

Average_values_per_frame_Radial_Strain_Rate_Epi_a_max,

Longitudinal_Strain_Rate_Epi_seg001_a_min,

Longitudinal_Strain_Rate_Epi_seg001_a_max,

Longitudinal_Strain_Rate_Epi_seg002_a_min,

Longitudinal_Strain_Rate_Epi_seg002_a_max,

Longitudinal_Strain_Rate_Epi_seg003_a_min,

Longitudinal_Strain_Rate_Epi_seg003_a_max,

Longitudinal_Strain_Rate_Epi_seg004_a_min,

Longitudinal_Strain_Rate_Epi_seg004_a_max,

Longitudinal_Strain_Rate_Epi_seg005_a_min,

Longitudinal_Strain_Rate_Epi_seg005_a_max,

Longitudinal_Strain_Rate_Epi_seg006_a_min,

Longitudinal_Strain_Rate_Epi_seg006_a_max,

Average_values_per_frame_Longitudinal_Strain_Rate_Epi_a_min,

Average_values_per_frame_Longitudinal_Strain_Rate_Epi_a_max,

Shear_seg001_a_min,

Shear_seg001_a_max,

Shear_seg002_a_min,

Shear_seg002_a_max,

Shear_seg003_a_min,

Shear_seg003_a_max,

Shear_seg004_a_min,

Shear_seg004_a_max,

Shear_seg005_a_min,

Shear_seg005_a_max,

Shear_seg006_a_min,

Shear_seg006_a_max,

Average_values_per_frame_Shear_a_min,

Average_values_per_frame_Shear_a_max,

Shear_Rate_seg001_a_min,

Shear_Rate_seg001_a_max,

Shear_Rate_seg002_a_min,

Shear_Rate_seg002_a_max,

Shear_Rate_seg003_a_min,

Shear_Rate_seg003_a_max,

Shear_Rate_seg004_a_min,

Shear_Rate_seg004_a_max,

Shear_Rate_seg005_a_min,

Shear_Rate_seg005_a_max,

Shear_Rate_seg006_a_min,

Shear_Rate_seg006_a_max,

Average_values_per_frame_Shear_Rate_a_min,

Average_values_per_frame_Shear_Rate_a_max))

}

write.csv(outTab, paste(type, ".csv",sep=""))

rm(list = ls())

# Part 2 Calculate maximum and minimum values as well as accelerations

library(xlsx)

dir="D:\\Data\\TAC_imaging\\strain-15day"

setwd(dir)

files=grep(".xlsx",dir(),value=T)

as.data.frame(files)

outTab=data.frame()

#利用循环语句提取数据

for (i in files[1:length(files)]){

time1 = unlist(strsplit(i,split = " "))

type=time1[1]

MouseID = time1[2]

time2=time1[3]

time3 = substr(time2,1,2)

#velocity

velocity_data <- read.xlsx(i,sheetIndex = 2, header = F)

colnames(velocity_data) <- velocity_data[3,]

name1 = paste("fr0",time3,sep = "")

col5 = grep(name1,colnames(velocity_data))

col4 = col5-1

col3 = col5-2

col2 = col5-3

col1 = col5-4

Timepoint1 = as.numeric(velocity_data[4,col1])

Timepoint2 = as.numeric(velocity_data[4,col2])

Timepoint3 = as.numeric(velocity_data[4,col3])

Timepoint4 = as.numeric(velocity_data[4,col4])

Timepoint5 = as.numeric(velocity_data[4,col5])

Time_duration1 = Timepoint2 - Timepoint1

Time_duration2 = Timepoint3 - Timepoint2

Time_duration3 = Timepoint4 - Timepoint3

Time_duration4 = Timepoint5 - Timepoint4

##Radial_Velocity_Endo

Radial_Velocity_Endo_seg001_point1 = as.numeric(velocity_data[8,col1])

Radial_Velocity_Endo_seg001_point2 = as.numeric(velocity_data[8,col2])

Radial_Velocity_Endo_seg001_point3 = as.numeric(velocity_data[8,col3])

Radial_Velocity_Endo_seg001_point4 = as.numeric(velocity_data[8,col4])

Radial_Velocity_Endo_seg001_point5 = as.numeric(velocity_data[8,col5])

Radial_Velocity_Endo_seg001_a1 = (Radial_Velocity_Endo_seg001_point2 - Radial_Velocity_Endo_seg001_point1)/Time_duration1

Radial_Velocity_Endo_seg001_a2 = (Radial_Velocity_Endo_seg001_point3 - Radial_Velocity_Endo_seg001_point2)/Time_duration2

Radial_Velocity_Endo_seg001_a3 = (Radial_Velocity_Endo_seg001_point4 - Radial_Velocity_Endo_seg001_point3)/Time_duration3

Radial_Velocity_Endo_seg001_a4 = (Radial_Velocity_Endo_seg001_point5 - Radial_Velocity_Endo_seg001_point4)/Time_duration4

Radial_Velocity_Endo_seg001_a_min = min(c(Radial_Velocity_Endo_seg001_a1,Radial_Velocity_Endo_seg001_a2,Radial_Velocity_Endo_seg001_a3,Radial_Velocity_Endo_seg001_a4))

Radial_Velocity_Endo_seg001_a_max = max(c(Radial_Velocity_Endo_seg001_a1,Radial_Velocity_Endo_seg001_a2,Radial_Velocity_Endo_seg001_a3,Radial_Velocity_Endo_seg001_a4))

Radial_Velocity_Endo_seg002_point1 = as.numeric(velocity_data[9,col1])

Radial_Velocity_Endo_seg002_point2 = as.numeric(velocity_data[9,col2])

Radial_Velocity_Endo_seg002_point3 = as.numeric(velocity_data[9,col3])

Radial_Velocity_Endo_seg002_point4 = as.numeric(velocity_data[9,col4])

Radial_Velocity_Endo_seg002_point5 = as.numeric(velocity_data[9,col5])

Radial_Velocity_Endo_seg002_a1 = (Radial_Velocity_Endo_seg002_point2 - Radial_Velocity_Endo_seg002_point1)/Time_duration1

Radial_Velocity_Endo_seg002_a2 = (Radial_Velocity_Endo_seg002_point3 - Radial_Velocity_Endo_seg002_point2)/Time_duration2

Radial_Velocity_Endo_seg002_a3 = (Radial_Velocity_Endo_seg002_point4 - Radial_Velocity_Endo_seg002_point3)/Time_duration3

Radial_Velocity_Endo_seg002_a4 = (Radial_Velocity_Endo_seg002_point5 - Radial_Velocity_Endo_seg002_point4)/Time_duration4

Radial_Velocity_Endo_seg002_a_min = min(c(Radial_Velocity_Endo_seg002_a1,Radial_Velocity_Endo_seg002_a2,Radial_Velocity_Endo_seg002_a3,Radial_Velocity_Endo_seg002_a4))

Radial_Velocity_Endo_seg002_a_max = max(c(Radial_Velocity_Endo_seg002_a1,Radial_Velocity_Endo_seg002_a2,Radial_Velocity_Endo_seg002_a3,Radial_Velocity_Endo_seg002_a4))

Radial_Velocity_Endo_seg003_point1 = as.numeric(velocity_data[10,col1])

Radial_Velocity_Endo_seg003_point2 = as.numeric(velocity_data[10,col2])

Radial_Velocity_Endo_seg003_point3 = as.numeric(velocity_data[10,col3])

Radial_Velocity_Endo_seg003_point4 = as.numeric(velocity_data[10,col4])

Radial_Velocity_Endo_seg003_point5 = as.numeric(velocity_data[10,col5])

Radial_Velocity_Endo_seg003_a1 = (Radial_Velocity_Endo_seg003_point2 - Radial_Velocity_Endo_seg003_point1)/Time_duration1

Radial_Velocity_Endo_seg003_a2 = (Radial_Velocity_Endo_seg003_point3 - Radial_Velocity_Endo_seg003_point2)/Time_duration2

Radial_Velocity_Endo_seg003_a3 = (Radial_Velocity_Endo_seg003_point4 - Radial_Velocity_Endo_seg003_point3)/Time_duration3

Radial_Velocity_Endo_seg003_a4 = (Radial_Velocity_Endo_seg003_point5 - Radial_Velocity_Endo_seg003_point4)/Time_duration4

Radial_Velocity_Endo_seg003_a_min = min(c(Radial_Velocity_Endo_seg003_a1,Radial_Velocity_Endo_seg003_a2,Radial_Velocity_Endo_seg003_a3,Radial_Velocity_Endo_seg003_a4))

Radial_Velocity_Endo_seg003_a_max = max(c(Radial_Velocity_Endo_seg003_a1,Radial_Velocity_Endo_seg003_a2,Radial_Velocity_Endo_seg003_a3,Radial_Velocity_Endo_seg003_a4))

Radial_Velocity_Endo_seg004_point1 = as.numeric(velocity_data[11,col1])

Radial_Velocity_Endo_seg004_point2 = as.numeric(velocity_data[11,col2])

Radial_Velocity_Endo_seg004_point3 = as.numeric(velocity_data[11,col3])

Radial_Velocity_Endo_seg004_point4 = as.numeric(velocity_data[11,col4])

Radial_Velocity_Endo_seg004_point5 = as.numeric(velocity_data[11,col5])

Radial_Velocity_Endo_seg004_a1 = (Radial_Velocity_Endo_seg004_point2 - Radial_Velocity_Endo_seg004_point1)/Time_duration1

Radial_Velocity_Endo_seg004_a2 = (Radial_Velocity_Endo_seg004_point3 - Radial_Velocity_Endo_seg004_point2)/Time_duration2

Radial_Velocity_Endo_seg004_a3 = (Radial_Velocity_Endo_seg004_point4 - Radial_Velocity_Endo_seg004_point3)/Time_duration3

Radial_Velocity_Endo_seg004_a4 = (Radial_Velocity_Endo_seg004_point5 - Radial_Velocity_Endo_seg004_point4)/Time_duration4

Radial_Velocity_Endo_seg004_a_min = min(c(Radial_Velocity_Endo_seg004_a1,Radial_Velocity_Endo_seg004_a2,Radial_Velocity_Endo_seg004_a3,Radial_Velocity_Endo_seg004_a4))

Radial_Velocity_Endo_seg004_a_max = max(c(Radial_Velocity_Endo_seg004_a1,Radial_Velocity_Endo_seg004_a2,Radial_Velocity_Endo_seg004_a3,Radial_Velocity_Endo_seg004_a4))

Radial_Velocity_Endo_seg005_point1 = as.numeric(velocity_data[12,col1])

Radial_Velocity_Endo_seg005_point2 = as.numeric(velocity_data[12,col2])

Radial_Velocity_Endo_seg005_point3 = as.numeric(velocity_data[12,col3])

Radial_Velocity_Endo_seg005_point4 = as.numeric(velocity_data[12,col4])

Radial_Velocity_Endo_seg005_point5 = as.numeric(velocity_data[12,col5])

Radial_Velocity_Endo_seg005_a1 = (Radial_Velocity_Endo_seg005_point2 - Radial_Velocity_Endo_seg005_point1)/Time_duration1

Radial_Velocity_Endo_seg005_a2 = (Radial_Velocity_Endo_seg005_point3 - Radial_Velocity_Endo_seg005_point2)/Time_duration2

Radial_Velocity_Endo_seg005_a3 = (Radial_Velocity_Endo_seg005_point4 - Radial_Velocity_Endo_seg005_point3)/Time_duration3

Radial_Velocity_Endo_seg005_a4 = (Radial_Velocity_Endo_seg005_point5 - Radial_Velocity_Endo_seg005_point4)/Time_duration4

Radial_Velocity_Endo_seg005_a_min = min(c(Radial_Velocity_Endo_seg005_a1,Radial_Velocity_Endo_seg005_a2,Radial_Velocity_Endo_seg005_a3,Radial_Velocity_Endo_seg005_a4))

Radial_Velocity_Endo_seg005_a_max = max(c(Radial_Velocity_Endo_seg005_a1,Radial_Velocity_Endo_seg005_a2,Radial_Velocity_Endo_seg005_a3,Radial_Velocity_Endo_seg005_a4))

Radial_Velocity_Endo_seg006_point1 = as.numeric(velocity_data[13,col1])

Radial_Velocity_Endo_seg006_point2 = as.numeric(velocity_data[13,col2])

Radial_Velocity_Endo_seg006_point3 = as.numeric(velocity_data[13,col3])

Radial_Velocity_Endo_seg006_point4 = as.numeric(velocity_data[13,col4])

Radial_Velocity_Endo_seg006_point5 = as.numeric(velocity_data[13,col5])

Radial_Velocity_Endo_seg006_a1 = (Radial_Velocity_Endo_seg006_point2 - Radial_Velocity_Endo_seg006_point1)/Time_duration1

Radial_Velocity_Endo_seg006_a2 = (Radial_Velocity_Endo_seg006_point3 - Radial_Velocity_Endo_seg006_point2)/Time_duration2

Radial_Velocity_Endo_seg006_a3 = (Radial_Velocity_Endo_seg006_point4 - Radial_Velocity_Endo_seg006_point3)/Time_duration3

Radial_Velocity_Endo_seg006_a4 = (Radial_Velocity_Endo_seg006_point5 - Radial_Velocity_Endo_seg006_point4)/Time_duration4

Radial_Velocity_Endo_seg006_a_min = min(c(Radial_Velocity_Endo_seg006_a1,Radial_Velocity_Endo_seg006_a2,Radial_Velocity_Endo_seg006_a3,Radial_Velocity_Endo_seg006_a4))

Radial_Velocity_Endo_seg006_a_max = max(c(Radial_Velocity_Endo_seg006_a1,Radial_Velocity_Endo_seg006_a2,Radial_Velocity_Endo_seg006_a3,Radial_Velocity_Endo_seg006_a4))

##Average values per frame Radial Velocity Endo

Average_values_per_frame_Radial_Velocity_Endo_point1 = as.numeric(velocity_data[17,col1])

Average_values_per_frame_Radial_Velocity_Endo_point2 = as.numeric(velocity_data[17,col2])

Average_values_per_frame_Radial_Velocity_Endo_point3 = as.numeric(velocity_data[17,col3])

Average_values_per_frame_Radial_Velocity_Endo_point4 = as.numeric(velocity_data[17,col4])

Average_values_per_frame_Radial_Velocity_Endo_point5 = as.numeric(velocity_data[17,col5])

Average_values_per_frame_Radial_Velocity_Endo_a1 = (Average_values_per_frame_Radial_Velocity_Endo_point2 - Average_values_per_frame_Radial_Velocity_Endo_point1)/Time_duration1

Average_values_per_frame_Radial_Velocity_Endo_a2 = (Average_values_per_frame_Radial_Velocity_Endo_point3 - Average_values_per_frame_Radial_Velocity_Endo_point2)/Time_duration2

Average_values_per_frame_Radial_Velocity_Endo_a3 = (Average_values_per_frame_Radial_Velocity_Endo_point4 - Average_values_per_frame_Radial_Velocity_Endo_point3)/Time_duration3

Average_values_per_frame_Radial_Velocity_Endo_a4 = (Average_values_per_frame_Radial_Velocity_Endo_point5 - Average_values_per_frame_Radial_Velocity_Endo_point4)/Time_duration4

Average_values_per_frame_Radial_Velocity_Endo_a_min = min(c(Average_values_per_frame_Radial_Velocity_Endo_a1,Average_values_per_frame_Radial_Velocity_Endo_a2,Average_values_per_frame_Radial_Velocity_Endo_a3,Average_values_per_frame_Radial_Velocity_Endo_a4))

Average_values_per_frame_Radial_Velocity_Endo_a_max = max(c(Average_values_per_frame_Radial_Velocity_Endo_a1,Average_values_per_frame_Radial_Velocity_Endo_a2,Average_values_per_frame_Radial_Velocity_Endo_a3,Average_values_per_frame_Radial_Velocity_Endo_a4))

##Longitudinal_Velocity_Endo

Longitudinal_Velocity_Endo_seg001_point1 = as.numeric(velocity_data[21,col1])

Longitudinal_Velocity_Endo_seg001_point2 = as.numeric(velocity_data[21,col2])

Longitudinal_Velocity_Endo_seg001_point3 = as.numeric(velocity_data[21,col3])

Longitudinal_Velocity_Endo_seg001_point4 = as.numeric(velocity_data[21,col4])

Longitudinal_Velocity_Endo_seg001_point5 = as.numeric(velocity_data[21,col5])

Longitudinal_Velocity_Endo_seg001_a1 = (Longitudinal_Velocity_Endo_seg001_point2 - Longitudinal_Velocity_Endo_seg001_point1)/Time_duration1

Longitudinal_Velocity_Endo_seg001_a2 = (Longitudinal_Velocity_Endo_seg001_point3 - Longitudinal_Velocity_Endo_seg001_point2)/Time_duration2

Longitudinal_Velocity_Endo_seg001_a3 = (Longitudinal_Velocity_Endo_seg001_point4 - Longitudinal_Velocity_Endo_seg001_point3)/Time_duration3

Longitudinal_Velocity_Endo_seg001_a4 = (Longitudinal_Velocity_Endo_seg001_point5 - Longitudinal_Velocity_Endo_seg001_point4)/Time_duration4

Longitudinal_Velocity_Endo_seg001_a_min = min(c(Longitudinal_Velocity_Endo_seg001_a1,Longitudinal_Velocity_Endo_seg001_a2,Longitudinal_Velocity_Endo_seg001_a3,Longitudinal_Velocity_Endo_seg001_a4))

Longitudinal_Velocity_Endo_seg001_a_max = max(c(Longitudinal_Velocity_Endo_seg001_a1,Longitudinal_Velocity_Endo_seg001_a2,Longitudinal_Velocity_Endo_seg001_a3,Longitudinal_Velocity_Endo_seg001_a4))

Longitudinal_Velocity_Endo_seg002_point1 = as.numeric(velocity_data[22,col1])

Longitudinal_Velocity_Endo_seg002_point2 = as.numeric(velocity_data[22,col2])

Longitudinal_Velocity_Endo_seg002_point3 = as.numeric(velocity_data[22,col3])

Longitudinal_Velocity_Endo_seg002_point4 = as.numeric(velocity_data[22,col4])

Longitudinal_Velocity_Endo_seg002_point5 = as.numeric(velocity_data[22,col5])

Longitudinal_Velocity_Endo_seg002_a1 = (Longitudinal_Velocity_Endo_seg002_point2 - Longitudinal_Velocity_Endo_seg002_point1)/Time_duration1

Longitudinal_Velocity_Endo_seg002_a2 = (Longitudinal_Velocity_Endo_seg002_point3 - Longitudinal_Velocity_Endo_seg002_point2)/Time_duration2

Longitudinal_Velocity_Endo_seg002_a3 = (Longitudinal_Velocity_Endo_seg002_point4 - Longitudinal_Velocity_Endo_seg002_point3)/Time_duration3

Longitudinal_Velocity_Endo_seg002_a4 = (Longitudinal_Velocity_Endo_seg002_point5 - Longitudinal_Velocity_Endo_seg002_point4)/Time_duration4

Longitudinal_Velocity_Endo_seg002_a_min = min(c(Longitudinal_Velocity_Endo_seg002_a1,Longitudinal_Velocity_Endo_seg002_a2,Longitudinal_Velocity_Endo_seg002_a3,Longitudinal_Velocity_Endo_seg002_a4))

Longitudinal_Velocity_Endo_seg002_a_max = max(c(Longitudinal_Velocity_Endo_seg002_a1,Longitudinal_Velocity_Endo_seg002_a2,Longitudinal_Velocity_Endo_seg002_a3,Longitudinal_Velocity_Endo_seg002_a4))

Longitudinal_Velocity_Endo_seg003_point1 = as.numeric(velocity_data[23,col1])

Longitudinal_Velocity_Endo_seg003_point2 = as.numeric(velocity_data[23,col2])

Longitudinal_Velocity_Endo_seg003_point3 = as.numeric(velocity_data[23,col3])

Longitudinal_Velocity_Endo_seg003_point4 = as.numeric(velocity_data[23,col4])

Longitudinal_Velocity_Endo_seg003_point5 = as.numeric(velocity_data[23,col5])

Longitudinal_Velocity_Endo_seg003_a1 = (Longitudinal_Velocity_Endo_seg003_point2 - Longitudinal_Velocity_Endo_seg003_point1)/Time_duration1

Longitudinal_Velocity_Endo_seg003_a2 = (Longitudinal_Velocity_Endo_seg003_point3 - Longitudinal_Velocity_Endo_seg003_point2)/Time_duration2

Longitudinal_Velocity_Endo_seg003_a3 = (Longitudinal_Velocity_Endo_seg003_point4 - Longitudinal_Velocity_Endo_seg003_point3)/Time_duration3

Longitudinal_Velocity_Endo_seg003_a4 = (Longitudinal_Velocity_Endo_seg003_point5 - Longitudinal_Velocity_Endo_seg003_point4)/Time_duration4

Longitudinal_Velocity_Endo_seg003_a_min = min(c(Longitudinal_Velocity_Endo_seg003_a1,Longitudinal_Velocity_Endo_seg003_a2,Longitudinal_Velocity_Endo_seg003_a3,Longitudinal_Velocity_Endo_seg003_a4))

Longitudinal_Velocity_Endo_seg003_a_max = max(c(Longitudinal_Velocity_Endo_seg003_a1,Longitudinal_Velocity_Endo_seg003_a2,Longitudinal_Velocity_Endo_seg003_a3,Longitudinal_Velocity_Endo_seg003_a4))

Longitudinal_Velocity_Endo_seg004_point1 = as.numeric(velocity_data[24,col1])

Longitudinal_Velocity_Endo_seg004_point2 = as.numeric(velocity_data[24,col2])

Longitudinal_Velocity_Endo_seg004_point3 = as.numeric(velocity_data[24,col3])

Longitudinal_Velocity_Endo_seg004_point4 = as.numeric(velocity_data[24,col4])

Longitudinal_Velocity_Endo_seg004_point5 = as.numeric(velocity_data[24,col5])

Longitudinal_Velocity_Endo_seg004_a1 = (Longitudinal_Velocity_Endo_seg004_point2 - Longitudinal_Velocity_Endo_seg004_point1)/Time_duration1

Longitudinal_Velocity_Endo_seg004_a2 = (Longitudinal_Velocity_Endo_seg004_point3 - Longitudinal_Velocity_Endo_seg004_point2)/Time_duration2

Longitudinal_Velocity_Endo_seg004_a3 = (Longitudinal_Velocity_Endo_seg004_point4 - Longitudinal_Velocity_Endo_seg004_point3)/Time_duration3

Longitudinal_Velocity_Endo_seg004_a4 = (Longitudinal_Velocity_Endo_seg004_point5 - Longitudinal_Velocity_Endo_seg004_point4)/Time_duration4

Longitudinal_Velocity_Endo_seg004_a_min = min(c(Longitudinal_Velocity_Endo_seg004_a1,Longitudinal_Velocity_Endo_seg004_a2,Longitudinal_Velocity_Endo_seg004_a3,Longitudinal_Velocity_Endo_seg004_a4))

Longitudinal_Velocity_Endo_seg004_a_max = max(c(Longitudinal_Velocity_Endo_seg004_a1,Longitudinal_Velocity_Endo_seg004_a2,Longitudinal_Velocity_Endo_seg004_a3,Longitudinal_Velocity_Endo_seg004_a4))

Longitudinal_Velocity_Endo_seg005_point1 = as.numeric(velocity_data[25,col1])

Longitudinal_Velocity_Endo_seg005_point2 = as.numeric(velocity_data[25,col2])

Longitudinal_Velocity_Endo_seg005_point3 = as.numeric(velocity_data[25,col3])

Longitudinal_Velocity_Endo_seg005_point4 = as.numeric(velocity_data[25,col4])

Longitudinal_Velocity_Endo_seg005_point5 = as.numeric(velocity_data[25,col5])

Longitudinal_Velocity_Endo_seg005_a1 = (Longitudinal_Velocity_Endo_seg005_point2 - Longitudinal_Velocity_Endo_seg005_point1)/Time_duration1

Longitudinal_Velocity_Endo_seg005_a2 = (Longitudinal_Velocity_Endo_seg005_point3 - Longitudinal_Velocity_Endo_seg005_point2)/Time_duration2

Longitudinal_Velocity_Endo_seg005_a3 = (Longitudinal_Velocity_Endo_seg005_point4 - Longitudinal_Velocity_Endo_seg005_point3)/Time_duration3

Longitudinal_Velocity_Endo_seg005_a4 = (Longitudinal_Velocity_Endo_seg005_point5 - Longitudinal_Velocity_Endo_seg005_point4)/Time_duration4

Longitudinal_Velocity_Endo_seg005_a_min = min(c(Longitudinal_Velocity_Endo_seg005_a1,Longitudinal_Velocity_Endo_seg005_a2,Longitudinal_Velocity_Endo_seg005_a3,Longitudinal_Velocity_Endo_seg005_a4))

Longitudinal_Velocity_Endo_seg005_a_max = max(c(Longitudinal_Velocity_Endo_seg005_a1,Longitudinal_Velocity_Endo_seg005_a2,Longitudinal_Velocity_Endo_seg005_a3,Longitudinal_Velocity_Endo_seg005_a4))

Longitudinal_Velocity_Endo_seg006_point1 = as.numeric(velocity_data[26,col1])

Longitudinal_Velocity_Endo_seg006_point2 = as.numeric(velocity_data[26,col2])

Longitudinal_Velocity_Endo_seg006_point3 = as.numeric(velocity_data[26,col3])

Longitudinal_Velocity_Endo_seg006_point4 = as.numeric(velocity_data[26,col4])

Longitudinal_Velocity_Endo_seg006_point5 = as.numeric(velocity_data[26,col5])

Longitudinal_Velocity_Endo_seg006_a1 = (Longitudinal_Velocity_Endo_seg006_point2 - Longitudinal_Velocity_Endo_seg006_point1)/Time_duration1

Longitudinal_Velocity_Endo_seg006_a2 = (Longitudinal_Velocity_Endo_seg006_point3 - Longitudinal_Velocity_Endo_seg006_point2)/Time_duration2

Longitudinal_Velocity_Endo_seg006_a3 = (Longitudinal_Velocity_Endo_seg006_point4 - Longitudinal_Velocity_Endo_seg006_point3)/Time_duration3

Longitudinal_Velocity_Endo_seg006_a4 = (Longitudinal_Velocity_Endo_seg006_point5 - Longitudinal_Velocity_Endo_seg006_point4)/Time_duration4

Longitudinal_Velocity_Endo_seg006_a_min = min(c(Longitudinal_Velocity_Endo_seg006_a1,Longitudinal_Velocity_Endo_seg006_a2,Longitudinal_Velocity_Endo_seg006_a3,Longitudinal_Velocity_Endo_seg006_a4))

Longitudinal_Velocity_Endo_seg006_a_max = max(c(Longitudinal_Velocity_Endo_seg006_a1,Longitudinal_Velocity_Endo_seg006_a2,Longitudinal_Velocity_Endo_seg006_a3,Longitudinal_Velocity_Endo_seg006_a4))

##Average values per frame Longitudinal Velocity Endo

Average_values_per_frame_Longitudinal_Velocity_Endo_point1 = as.numeric(velocity_data[30,col1])

Average_values_per_frame_Longitudinal_Velocity_Endo_point2 = as.numeric(velocity_data[30,col2])

Average_values_per_frame_Longitudinal_Velocity_Endo_point3 = as.numeric(velocity_data[30,col3])

Average_values_per_frame_Longitudinal_Velocity_Endo_point4 = as.numeric(velocity_data[30,col4])

Average_values_per_frame_Longitudinal_Velocity_Endo_point5 = as.numeric(velocity_data[30,col5])

Average_values_per_frame_Longitudinal_Velocity_Endo_a1 = (Average_values_per_frame_Longitudinal_Velocity_Endo_point2 - Average_values_per_frame_Longitudinal_Velocity_Endo_point1)/Time_duration1

Average_values_per_frame_Longitudinal_Velocity_Endo_a2 = (Average_values_per_frame_Longitudinal_Velocity_Endo_point3 - Average_values_per_frame_Longitudinal_Velocity_Endo_point2)/Time_duration2

Average_values_per_frame_Longitudinal_Velocity_Endo_a3 = (Average_values_per_frame_Longitudinal_Velocity_Endo_point4 - Average_values_per_frame_Longitudinal_Velocity_Endo_point3)/Time_duration3

Average_values_per_frame_Longitudinal_Velocity_Endo_a4 = (Average_values_per_frame_Longitudinal_Velocity_Endo_point5 - Average_values_per_frame_Longitudinal_Velocity_Endo_point4)/Time_duration4

Average_values_per_frame_Longitudinal_Velocity_Endo_a_min = min(c(Average_values_per_frame_Longitudinal_Velocity_Endo_a1,Average_values_per_frame_Longitudinal_Velocity_Endo_a2,Average_values_per_frame_Longitudinal_Velocity_Endo_a3,Average_values_per_frame_Longitudinal_Velocity_Endo_a4))

Average_values_per_frame_Longitudinal_Velocity_Endo_a_max = max(c(Average_values_per_frame_Longitudinal_Velocity_Endo_a1,Average_values_per_frame_Longitudinal_Velocity_Endo_a2,Average_values_per_frame_Longitudinal_Velocity_Endo_a3,Average_values_per_frame_Longitudinal_Velocity_Endo_a4))

##Radial Velocity Epi

Radial_Velocity_Epi_seg001_point1 = as.numeric(velocity_data[34,col1])

Radial_Velocity_Epi_seg001_point2 = as.numeric(velocity_data[34,col2])

Radial_Velocity_Epi_seg001_point3 = as.numeric(velocity_data[34,col3])

Radial_Velocity_Epi_seg001_point4 = as.numeric(velocity_data[34,col4])

Radial_Velocity_Epi_seg001_point5 = as.numeric(velocity_data[34,col5])

Radial_Velocity_Epi_seg001_a1 = (Radial_Velocity_Epi_seg001_point2 - Radial_Velocity_Epi_seg001_point1)/Time_duration1

Radial_Velocity_Epi_seg001_a2 = (Radial_Velocity_Epi_seg001_point3 - Radial_Velocity_Epi_seg001_point2)/Time_duration2

Radial_Velocity_Epi_seg001_a3 = (Radial_Velocity_Epi_seg001_point4 - Radial_Velocity_Epi_seg001_point3)/Time_duration3

Radial_Velocity_Epi_seg001_a4 = (Radial_Velocity_Epi_seg001_point5 - Radial_Velocity_Epi_seg001_point4)/Time_duration4

Radial_Velocity_Epi_seg001_a_min = min(c(Radial_Velocity_Epi_seg001_a1,Radial_Velocity_Epi_seg001_a2,Radial_Velocity_Epi_seg001_a3,Radial_Velocity_Epi_seg001_a4))

Radial_Velocity_Epi_seg001_a_max = max(c(Radial_Velocity_Epi_seg001_a1,Radial_Velocity_Epi_seg001_a2,Radial_Velocity_Epi_seg001_a3,Radial_Velocity_Epi_seg001_a4))

Radial_Velocity_Epi_seg002_point1 = as.numeric(velocity_data[35,col1])

Radial_Velocity_Epi_seg002_point2 = as.numeric(velocity_data[35,col2])

Radial_Velocity_Epi_seg002_point3 = as.numeric(velocity_data[35,col3])

Radial_Velocity_Epi_seg002_point4 = as.numeric(velocity_data[35,col4])

Radial_Velocity_Epi_seg002_point5 = as.numeric(velocity_data[35,col5])

Radial_Velocity_Epi_seg002_a1 = (Radial_Velocity_Epi_seg002_point2 - Radial_Velocity_Epi_seg002_point1)/Time_duration1

Radial_Velocity_Epi_seg002_a2 = (Radial_Velocity_Epi_seg002_point3 - Radial_Velocity_Epi_seg002_point2)/Time_duration2

Radial_Velocity_Epi_seg002_a3 = (Radial_Velocity_Epi_seg002_point4 - Radial_Velocity_Epi_seg002_point3)/Time_duration3

Radial_Velocity_Epi_seg002_a4 = (Radial_Velocity_Epi_seg002_point5 - Radial_Velocity_Epi_seg002_point4)/Time_duration4

Radial_Velocity_Epi_seg002_a_min = min(c(Radial_Velocity_Epi_seg002_a1,Radial_Velocity_Epi_seg002_a2,Radial_Velocity_Epi_seg002_a3,Radial_Velocity_Epi_seg002_a4))

Radial_Velocity_Epi_seg002_a_max = max(c(Radial_Velocity_Epi_seg002_a1,Radial_Velocity_Epi_seg002_a2,Radial_Velocity_Epi_seg002_a3,Radial_Velocity_Epi_seg002_a4))

Radial_Velocity_Epi_seg003_point1 = as.numeric(velocity_data[36,col1])

Radial_Velocity_Epi_seg003_point2 = as.numeric(velocity_data[36,col2])

Radial_Velocity_Epi_seg003_point3 = as.numeric(velocity_data[36,col3])

Radial_Velocity_Epi_seg003_point4 = as.numeric(velocity_data[36,col4])

Radial_Velocity_Epi_seg003_point5 = as.numeric(velocity_data[36,col5])

Radial_Velocity_Epi_seg003_a1 = (Radial_Velocity_Epi_seg003_point2 - Radial_Velocity_Epi_seg003_point1)/Time_duration1

Radial_Velocity_Epi_seg003_a2 = (Radial_Velocity_Epi_seg003_point3 - Radial_Velocity_Epi_seg003_point2)/Time_duration2

Radial_Velocity_Epi_seg003_a3 = (Radial_Velocity_Epi_seg003_point4 - Radial_Velocity_Epi_seg003_point3)/Time_duration3

Radial_Velocity_Epi_seg003_a4 = (Radial_Velocity_Epi_seg003_point5 - Radial_Velocity_Epi_seg003_point4)/Time_duration4

Radial_Velocity_Epi_seg003_a_min = min(c(Radial_Velocity_Epi_seg003_a1,Radial_Velocity_Epi_seg003_a2,Radial_Velocity_Epi_seg003_a3,Radial_Velocity_Epi_seg003_a4))

Radial_Velocity_Epi_seg003_a_max = max(c(Radial_Velocity_Epi_seg003_a1,Radial_Velocity_Epi_seg003_a2,Radial_Velocity_Epi_seg003_a3,Radial_Velocity_Epi_seg003_a4))

Radial_Velocity_Epi_seg004_point1 = as.numeric(velocity_data[37,col1])

Radial_Velocity_Epi_seg004_point2 = as.numeric(velocity_data[37,col2])

Radial_Velocity_Epi_seg004_point3 = as.numeric(velocity_data[37,col3])

Radial_Velocity_Epi_seg004_point4 = as.numeric(velocity_data[37,col4])

Radial_Velocity_Epi_seg004_point5 = as.numeric(velocity_data[37,col5])

Radial_Velocity_Epi_seg004_a1 = (Radial_Velocity_Epi_seg004_point2 - Radial_Velocity_Epi_seg004_point1)/Time_duration1

Radial_Velocity_Epi_seg004_a2 = (Radial_Velocity_Epi_seg004_point3 - Radial_Velocity_Epi_seg004_point2)/Time_duration2

Radial_Velocity_Epi_seg004_a3 = (Radial_Velocity_Epi_seg004_point4 - Radial_Velocity_Epi_seg004_point3)/Time_duration3

Radial_Velocity_Epi_seg004_a4 = (Radial_Velocity_Epi_seg004_point5 - Radial_Velocity_Epi_seg004_point4)/Time_duration4

Radial_Velocity_Epi_seg004_a_min = min(c(Radial_Velocity_Epi_seg004_a1,Radial_Velocity_Epi_seg004_a2,Radial_Velocity_Epi_seg004_a3,Radial_Velocity_Epi_seg004_a4))

Radial_Velocity_Epi_seg004_a_max = max(c(Radial_Velocity_Epi_seg004_a1,Radial_Velocity_Epi_seg004_a2,Radial_Velocity_Epi_seg004_a3,Radial_Velocity_Epi_seg004_a4))

Radial_Velocity_Epi_seg005_point1 = as.numeric(velocity_data[38,col1])

Radial_Velocity_Epi_seg005_point2 = as.numeric(velocity_data[38,col2])

Radial_Velocity_Epi_seg005_point3 = as.numeric(velocity_data[38,col3])

Radial_Velocity_Epi_seg005_point4 = as.numeric(velocity_data[38,col4])

Radial_Velocity_Epi_seg005_point5 = as.numeric(velocity_data[38,col5])

Radial_Velocity_Epi_seg005_a1 = (Radial_Velocity_Epi_seg005_point2 - Radial_Velocity_Epi_seg005_point1)/Time_duration1

Radial_Velocity_Epi_seg005_a2 = (Radial_Velocity_Epi_seg005_point3 - Radial_Velocity_Epi_seg005_point2)/Time_duration2

Radial_Velocity_Epi_seg005_a3 = (Radial_Velocity_Epi_seg005_point4 - Radial_Velocity_Epi_seg005_point3)/Time_duration3

Radial_Velocity_Epi_seg005_a4 = (Radial_Velocity_Epi_seg005_point5 - Radial_Velocity_Epi_seg005_point4)/Time_duration4

Radial_Velocity_Epi_seg005_a_min = min(c(Radial_Velocity_Epi_seg005_a1,Radial_Velocity_Epi_seg005_a2,Radial_Velocity_Epi_seg005_a3,Radial_Velocity_Epi_seg005_a4))

Radial_Velocity_Epi_seg005_a_max = max(c(Radial_Velocity_Epi_seg005_a1,Radial_Velocity_Epi_seg005_a2,Radial_Velocity_Epi_seg005_a3,Radial_Velocity_Epi_seg005_a4))

Radial_Velocity_Epi_seg006_point1 = as.numeric(velocity_data[39,col1])

Radial_Velocity_Epi_seg006_point2 = as.numeric(velocity_data[39,col2])

Radial_Velocity_Epi_seg006_point3 = as.numeric(velocity_data[39,col3])

Radial_Velocity_Epi_seg006_point4 = as.numeric(velocity_data[39,col4])

Radial_Velocity_Epi_seg006_point5 = as.numeric(velocity_data[39,col5])

Radial_Velocity_Epi_seg006_a1 = (Radial_Velocity_Epi_seg006_point2 - Radial_Velocity_Epi_seg006_point1)/Time_duration1

Radial_Velocity_Epi_seg006_a2 = (Radial_Velocity_Epi_seg006_point3 - Radial_Velocity_Epi_seg006_point2)/Time_duration2

Radial_Velocity_Epi_seg006_a3 = (Radial_Velocity_Epi_seg006_point4 - Radial_Velocity_Epi_seg006_point3)/Time_duration3

Radial_Velocity_Epi_seg006_a4 = (Radial_Velocity_Epi_seg006_point5 - Radial_Velocity_Epi_seg006_point4)/Time_duration4

Radial_Velocity_Epi_seg006_a_min = min(c(Radial_Velocity_Epi_seg006_a1,Radial_Velocity_Epi_seg006_a2,Radial_Velocity_Epi_seg006_a3,Radial_Velocity_Epi_seg006_a4))

Radial_Velocity_Epi_seg006_a_max = max(c(Radial_Velocity_Epi_seg006_a1,Radial_Velocity_Epi_seg006_a2,Radial_Velocity_Epi_seg006_a3,Radial_Velocity_Epi_seg006_a4))

##Average values per frame Radial Velocity Epi

Average_values_per_frame_Radial_Velocity_Epi_point1 = as.numeric(velocity_data[43,col1])

Average_values_per_frame_Radial_Velocity_Epi_point2 = as.numeric(velocity_data[43,col2])

Average_values_per_frame_Radial_Velocity_Epi_point3 = as.numeric(velocity_data[43,col3])

Average_values_per_frame_Radial_Velocity_Epi_point4 = as.numeric(velocity_data[43,col4])

Average_values_per_frame_Radial_Velocity_Epi_point5 = as.numeric(velocity_data[43,col5])

Average_values_per_frame_Radial_Velocity_Epi_a1 = (Average_values_per_frame_Radial_Velocity_Epi_point2 - Average_values_per_frame_Radial_Velocity_Epi_point1)/Time_duration1

Average_values_per_frame_Radial_Velocity_Epi_a2 = (Average_values_per_frame_Radial_Velocity_Epi_point3 - Average_values_per_frame_Radial_Velocity_Epi_point2)/Time_duration2

Average_values_per_frame_Radial_Velocity_Epi_a3 = (Average_values_per_frame_Radial_Velocity_Epi_point4 - Average_values_per_frame_Radial_Velocity_Epi_point3)/Time_duration3

Average_values_per_frame_Radial_Velocity_Epi_a4 = (Average_values_per_frame_Radial_Velocity_Epi_point5 - Average_values_per_frame_Radial_Velocity_Epi_point4)/Time_duration4

Average_values_per_frame_Radial_Velocity_Epi_a_min = min(c(Average_values_per_frame_Radial_Velocity_Epi_a1,Average_values_per_frame_Radial_Velocity_Epi_a2,Average_values_per_frame_Radial_Velocity_Epi_a3,Average_values_per_frame_Radial_Velocity_Epi_a4))

Average_values_per_frame_Radial_Velocity_Epi_a_max = max(c(Average_values_per_frame_Radial_Velocity_Epi_a1,Average_values_per_frame_Radial_Velocity_Epi_a2,Average_values_per_frame_Radial_Velocity_Epi_a3,Average_values_per_frame_Radial_Velocity_Epi_a4))

##Longitudinal Velocity Epi

Longitudinal_Velocity_Epi_seg001_point1 = as.numeric(velocity_data[47,col1])

Longitudinal_Velocity_Epi_seg001_point2 = as.numeric(velocity_data[47,col2])

Longitudinal_Velocity_Epi_seg001_point3 = as.numeric(velocity_data[47,col3])

Longitudinal_Velocity_Epi_seg001_point4 = as.numeric(velocity_data[47,col4])

Longitudinal_Velocity_Epi_seg001_point5 = as.numeric(velocity_data[47,col5])

Longitudinal_Velocity_Epi_seg001_a1 = (Longitudinal_Velocity_Epi_seg001_point2 - Longitudinal_Velocity_Epi_seg001_point1)/Time_duration1

Longitudinal_Velocity_Epi_seg001_a2 = (Longitudinal_Velocity_Epi_seg001_point3 - Longitudinal_Velocity_Epi_seg001_point2)/Time_duration2

Longitudinal_Velocity_Epi_seg001_a3 = (Longitudinal_Velocity_Epi_seg001_point4 - Longitudinal_Velocity_Epi_seg001_point3)/Time_duration3

Longitudinal_Velocity_Epi_seg001_a4 = (Longitudinal_Velocity_Epi_seg001_point5 - Longitudinal_Velocity_Epi_seg001_point4)/Time_duration4

Longitudinal_Velocity_Epi_seg001_a_min = min(c(Longitudinal_Velocity_Epi_seg001_a1,Longitudinal_Velocity_Epi_seg001_a2,Longitudinal_Velocity_Epi_seg001_a3,Longitudinal_Velocity_Epi_seg001_a4))

Longitudinal_Velocity_Epi_seg001_a_max = max(c(Longitudinal_Velocity_Epi_seg001_a1,Longitudinal_Velocity_Epi_seg001_a2,Longitudinal_Velocity_Epi_seg001_a3,Longitudinal_Velocity_Epi_seg001_a4))

Longitudinal_Velocity_Epi_seg002_point1 = as.numeric(velocity_data[48,col1])

Longitudinal_Velocity_Epi_seg002_point2 = as.numeric(velocity_data[48,col2])

Longitudinal_Velocity_Epi_seg002_point3 = as.numeric(velocity_data[48,col3])

Longitudinal_Velocity_Epi_seg002_point4 = as.numeric(velocity_data[48,col4])

Longitudinal_Velocity_Epi_seg002_point5 = as.numeric(velocity_data[48,col5])

Longitudinal_Velocity_Epi_seg002_a1 = (Longitudinal_Velocity_Epi_seg002_point2 - Longitudinal_Velocity_Epi_seg002_point1)/Time_duration1

Longitudinal_Velocity_Epi_seg002_a2 = (Longitudinal_Velocity_Epi_seg002_point3 - Longitudinal_Velocity_Epi_seg002_point2)/Time_duration2

Longitudinal_Velocity_Epi_seg002_a3 = (Longitudinal_Velocity_Epi_seg002_point4 - Longitudinal_Velocity_Epi_seg002_point3)/Time_duration3

Longitudinal_Velocity_Epi_seg002_a4 = (Longitudinal_Velocity_Epi_seg002_point5 - Longitudinal_Velocity_Epi_seg002_point4)/Time_duration4

Longitudinal_Velocity_Epi_seg002_a_min = min(c(Longitudinal_Velocity_Epi_seg002_a1,Longitudinal_Velocity_Epi_seg002_a2,Longitudinal_Velocity_Epi_seg002_a3,Longitudinal_Velocity_Epi_seg002_a4))

Longitudinal_Velocity_Epi_seg002_a_max = max(c(Longitudinal_Velocity_Epi_seg002_a1,Longitudinal_Velocity_Epi_seg002_a2,Longitudinal_Velocity_Epi_seg002_a3,Longitudinal_Velocity_Epi_seg002_a4))

Longitudinal_Velocity_Epi_seg003_point1 = as.numeric(velocity_data[49,col1])

Longitudinal_Velocity_Epi_seg003_point2 = as.numeric(velocity_data[49,col2])

Longitudinal_Velocity_Epi_seg003_point3 = as.numeric(velocity_data[49,col3])

Longitudinal_Velocity_Epi_seg003_point4 = as.numeric(velocity_data[49,col4])

Longitudinal_Velocity_Epi_seg003_point5 = as.numeric(velocity_data[49,col5])

Longitudinal_Velocity_Epi_seg003_a1 = (Longitudinal_Velocity_Epi_seg003_point2 - Longitudinal_Velocity_Epi_seg003_point1)/Time_duration1

Longitudinal_Velocity_Epi_seg003_a2 = (Longitudinal_Velocity_Epi_seg003_point3 - Longitudinal_Velocity_Epi_seg003_point2)/Time_duration2

Longitudinal_Velocity_Epi_seg003_a3 = (Longitudinal_Velocity_Epi_seg003_point4 - Longitudinal_Velocity_Epi_seg003_point3)/Time_duration3

Longitudinal_Velocity_Epi_seg003_a4 = (Longitudinal_Velocity_Epi_seg003_point5 - Longitudinal_Velocity_Epi_seg003_point4)/Time_duration4

Longitudinal_Velocity_Epi_seg003_a_min = min(c(Longitudinal_Velocity_Epi_seg003_a1,Longitudinal_Velocity_Epi_seg003_a2,Longitudinal_Velocity_Epi_seg003_a3,Longitudinal_Velocity_Epi_seg003_a4))

Longitudinal_Velocity_Epi_seg003_a_max = max(c(Longitudinal_Velocity_Epi_seg003_a1,Longitudinal_Velocity_Epi_seg003_a2,Longitudinal_Velocity_Epi_seg003_a3,Longitudinal_Velocity_Epi_seg003_a4))

Longitudinal_Velocity_Epi_seg004_point1 = as.numeric(velocity_data[50,col1])

Longitudinal_Velocity_Epi_seg004_point2 = as.numeric(velocity_data[50,col2])

Longitudinal_Velocity_Epi_seg004_point3 = as.numeric(velocity_data[50,col3])

Longitudinal_Velocity_Epi_seg004_point4 = as.numeric(velocity_data[50,col4])

Longitudinal_Velocity_Epi_seg004_point5 = as.numeric(velocity_data[50,col5])

Longitudinal_Velocity_Epi_seg004_a1 = (Longitudinal_Velocity_Epi_seg004_point2 - Longitudinal_Velocity_Epi_seg004_point1)/Time_duration1

Longitudinal_Velocity_Epi_seg004_a2 = (Longitudinal_Velocity_Epi_seg004_point3 - Longitudinal_Velocity_Epi_seg004_point2)/Time_duration2

Longitudinal_Velocity_Epi_seg004_a3 = (Longitudinal_Velocity_Epi_seg004_point4 - Longitudinal_Velocity_Epi_seg004_point3)/Time_duration3

Longitudinal_Velocity_Epi_seg004_a4 = (Longitudinal_Velocity_Epi_seg004_point5 - Longitudinal_Velocity_Epi_seg004_point4)/Time_duration4

Longitudinal_Velocity_Epi_seg004_a_min = min(c(Longitudinal_Velocity_Epi_seg004_a1,Longitudinal_Velocity_Epi_seg004_a2,Longitudinal_Velocity_Epi_seg004_a3,Longitudinal_Velocity_Epi_seg004_a4))

Longitudinal_Velocity_Epi_seg004_a_max = max(c(Longitudinal_Velocity_Epi_seg004_a1,Longitudinal_Velocity_Epi_seg004_a2,Longitudinal_Velocity_Epi_seg004_a3,Longitudinal_Velocity_Epi_seg004_a4))

Longitudinal_Velocity_Epi_seg005_point1 = as.numeric(velocity_data[51,col1])

Longitudinal_Velocity_Epi_seg005_point2 = as.numeric(velocity_data[51,col2])

Longitudinal_Velocity_Epi_seg005_point3 = as.numeric(velocity_data[51,col3])

Longitudinal_Velocity_Epi_seg005_point4 = as.numeric(velocity_data[51,col4])

Longitudinal_Velocity_Epi_seg005_point5 = as.numeric(velocity_data[51,col5])

Longitudinal_Velocity_Epi_seg005_a1 = (Longitudinal_Velocity_Epi_seg005_point2 - Longitudinal_Velocity_Epi_seg005_point1)/Time_duration1

Longitudinal_Velocity_Epi_seg005_a2 = (Longitudinal_Velocity_Epi_seg005_point3 - Longitudinal_Velocity_Epi_seg005_point2)/Time_duration2

Longitudinal_Velocity_Epi_seg005_a3 = (Longitudinal_Velocity_Epi_seg005_point4 - Longitudinal_Velocity_Epi_seg005_point3)/Time_duration3

Longitudinal_Velocity_Epi_seg005_a4 = (Longitudinal_Velocity_Epi_seg005_point5 - Longitudinal_Velocity_Epi_seg005_point4)/Time_duration4

Longitudinal_Velocity_Epi_seg005_a_min = min(c(Longitudinal_Velocity_Epi_seg005_a1,Longitudinal_Velocity_Epi_seg005_a2,Longitudinal_Velocity_Epi_seg005_a3,Longitudinal_Velocity_Epi_seg005_a4))

Longitudinal_Velocity_Epi_seg005_a_max = max(c(Longitudinal_Velocity_Epi_seg005_a1,Longitudinal_Velocity_Epi_seg005_a2,Longitudinal_Velocity_Epi_seg005_a3,Longitudinal_Velocity_Epi_seg005_a4))

Longitudinal_Velocity_Epi_seg006_point1 = as.numeric(velocity_data[52,col1])

Longitudinal_Velocity_Epi_seg006_point2 = as.numeric(velocity_data[52,col2])

Longitudinal_Velocity_Epi_seg006_point3 = as.numeric(velocity_data[52,col3])

Longitudinal_Velocity_Epi_seg006_point4 = as.numeric(velocity_data[52,col4])

Longitudinal_Velocity_Epi_seg006_point5 = as.numeric(velocity_data[52,col5])

Longitudinal_Velocity_Epi_seg006_a1 = (Longitudinal_Velocity_Epi_seg006_point2 - Longitudinal_Velocity_Epi_seg006_point1)/Time_duration1

Longitudinal_Velocity_Epi_seg006_a2 = (Longitudinal_Velocity_Epi_seg006_point3 - Longitudinal_Velocity_Epi_seg006_point2)/Time_duration2

Longitudinal_Velocity_Epi_seg006_a3 = (Longitudinal_Velocity_Epi_seg006_point4 - Longitudinal_Velocity_Epi_seg006_point3)/Time_duration3

Longitudinal_Velocity_Epi_seg006_a4 = (Longitudinal_Velocity_Epi_seg006_point5 - Longitudinal_Velocity_Epi_seg006_point4)/Time_duration4

Longitudinal_Velocity_Epi_seg006_a_min = min(c(Longitudinal_Velocity_Epi_seg006_a1,Longitudinal_Velocity_Epi_seg006_a2,Longitudinal_Velocity_Epi_seg006_a3,Longitudinal_Velocity_Epi_seg006_a4))

Longitudinal_Velocity_Epi_seg006_a_max = max(c(Longitudinal_Velocity_Epi_seg006_a1,Longitudinal_Velocity_Epi_seg006_a2,Longitudinal_Velocity_Epi_seg006_a3,Longitudinal_Velocity_Epi_seg006_a4))

##Average values per frame Longitudinal Velocity Epi

Average_values_per_frame_Longitudinal_Velocity_Epi_point1 = as.numeric(velocity_data[56,col1])

Average_values_per_frame_Longitudinal_Velocity_Epi_point2 = as.numeric(velocity_data[56,col2])

Average_values_per_frame_Longitudinal_Velocity_Epi_point3 = as.numeric(velocity_data[56,col3])

Average_values_per_frame_Longitudinal_Velocity_Epi_point4 = as.numeric(velocity_data[56,col4])

Average_values_per_frame_Longitudinal_Velocity_Epi_point5 = as.numeric(velocity_data[56,col5])

Average_values_per_frame_Longitudinal_Velocity_Epi_a1 = (Average_values_per_frame_Longitudinal_Velocity_Epi_point2 - Average_values_per_frame_Longitudinal_Velocity_Epi_point1)/Time_duration1

Average_values_per_frame_Longitudinal_Velocity_Epi_a2 = (Average_values_per_frame_Longitudinal_Velocity_Epi_point3 - Average_values_per_frame_Longitudinal_Velocity_Epi_point2)/Time_duration2

Average_values_per_frame_Longitudinal_Velocity_Epi_a3 = (Average_values_per_frame_Longitudinal_Velocity_Epi_point4 - Average_values_per_frame_Longitudinal_Velocity_Epi_point3)/Time_duration3

Average_values_per_frame_Longitudinal_Velocity_Epi_a4 = (Average_values_per_frame_Longitudinal_Velocity_Epi_point5 - Average_values_per_frame_Longitudinal_Velocity_Epi_point4)/Time_duration4

Average_values_per_frame_Longitudinal_Velocity_Epi_a_min = min(c(Average_values_per_frame_Longitudinal_Velocity_Epi_a1,Average_values_per_frame_Longitudinal_Velocity_Epi_a2,Average_values_per_frame_Longitudinal_Velocity_Epi_a3,Average_values_per_frame_Longitudinal_Velocity_Epi_a4))

Average_values_per_frame_Longitudinal_Velocity_Epi_a_max = max(c(Average_values_per_frame_Longitudinal_Velocity_Epi_a1,Average_values_per_frame_Longitudinal_Velocity_Epi_a2,Average_values_per_frame_Longitudinal_Velocity_Epi_a3,Average_values_per_frame_Longitudinal_Velocity_Epi_a4))

#Displacement

Displacement_data <- read.xlsx(i,sheetIndex = 3, header = F)

colnames(Displacement_data) <- Displacement_data[3,]

name1 = paste("fr0",time3,sep = "")

col5 = grep(name1,colnames(Displacement_data))

col4 = col5-1

col3 = col5-2

col2 = col5-3

col1 = col5-4

Timepoint1 = as.numeric(Displacement_data[4,col1])

Timepoint2 = as.numeric(Displacement_data[4,col2])

Timepoint3 = as.numeric(Displacement_data[4,col3])

Timepoint4 = as.numeric(Displacement_data[4,col4])

Timepoint5 = as.numeric(Displacement_data[4,col5])

Time_duration1 = Timepoint2 - Timepoint1

Time_duration2 = Timepoint3 - Timepoint2

Time_duration3 = Timepoint4 - Timepoint3

Time_duration4 = Timepoint5 - Timepoint4

##Radial Displacement Endo

Radial_Displacement_Endo_seg001_point1 = as.numeric(Displacement_data[8,col1])

Radial_Displacement_Endo_seg001_point2 = as.numeric(Displacement_data[8,col2])

Radial_Displacement_Endo_seg001_point3 = as.numeric(Displacement_data[8,col3])

Radial_Displacement_Endo_seg001_point4 = as.numeric(Displacement_data[8,col4])

Radial_Displacement_Endo_seg001_point5 = as.numeric(Displacement_data[8,col5])

Radial_Displacement_Endo_seg001_a1 = (Radial_Displacement_Endo_seg001_point2 - Radial_Displacement_Endo_seg001_point1)/Time_duration1

Radial_Displacement_Endo_seg001_a2 = (Radial_Displacement_Endo_seg001_point3 - Radial_Displacement_Endo_seg001_point2)/Time_duration2

Radial_Displacement_Endo_seg001_a3 = (Radial_Displacement_Endo_seg001_point4 - Radial_Displacement_Endo_seg001_point3)/Time_duration3

Radial_Displacement_Endo_seg001_a4 = (Radial_Displacement_Endo_seg001_point5 - Radial_Displacement_Endo_seg001_point4)/Time_duration4

Radial_Displacement_Endo_seg001_a_min = min(c(Radial_Displacement_Endo_seg001_a1,Radial_Displacement_Endo_seg001_a2,Radial_Displacement_Endo_seg001_a3,Radial_Displacement_Endo_seg001_a4))

Radial_Displacement_Endo_seg001_a_max = max(c(Radial_Displacement_Endo_seg001_a1,Radial_Displacement_Endo_seg001_a2,Radial_Displacement_Endo_seg001_a3,Radial_Displacement_Endo_seg001_a4))

Radial_Displacement_Endo_seg002_point1 = as.numeric(Displacement_data[9,col1])

Radial_Displacement_Endo_seg002_point2 = as.numeric(Displacement_data[9,col2])

Radial_Displacement_Endo_seg002_point3 = as.numeric(Displacement_data[9,col3])

Radial_Displacement_Endo_seg002_point4 = as.numeric(Displacement_data[9,col4])

Radial_Displacement_Endo_seg002_point5 = as.numeric(Displacement_data[9,col5])

Radial_Displacement_Endo_seg002_a1 = (Radial_Displacement_Endo_seg002_point2 - Radial_Displacement_Endo_seg002_point1)/Time_duration1

Radial_Displacement_Endo_seg002_a2 = (Radial_Displacement_Endo_seg002_point3 - Radial_Displacement_Endo_seg002_point2)/Time_duration2

Radial_Displacement_Endo_seg002_a3 = (Radial_Displacement_Endo_seg002_point4 - Radial_Displacement_Endo_seg002_point3)/Time_duration3

Radial_Displacement_Endo_seg002_a4 = (Radial_Displacement_Endo_seg002_point5 - Radial_Displacement_Endo_seg002_point4)/Time_duration4

Radial_Displacement_Endo_seg002_a_min = min(c(Radial_Displacement_Endo_seg002_a1,Radial_Displacement_Endo_seg002_a2,Radial_Displacement_Endo_seg002_a3,Radial_Displacement_Endo_seg002_a4))

Radial_Displacement_Endo_seg002_a_max = max(c(Radial_Displacement_Endo_seg002_a1,Radial_Displacement_Endo_seg002_a2,Radial_Displacement_Endo_seg002_a3,Radial_Displacement_Endo_seg002_a4))

Radial_Displacement_Endo_seg003_point1 = as.numeric(Displacement_data[10,col1])

Radial_Displacement_Endo_seg003_point2 = as.numeric(Displacement_data[10,col2])

Radial_Displacement_Endo_seg003_point3 = as.numeric(Displacement_data[10,col3])

Radial_Displacement_Endo_seg003_point4 = as.numeric(Displacement_data[10,col4])

Radial_Displacement_Endo_seg003_point5 = as.numeric(Displacement_data[10,col5])

Radial_Displacement_Endo_seg003_a1 = (Radial_Displacement_Endo_seg003_point2 - Radial_Displacement_Endo_seg003_point1)/Time_duration1

Radial_Displacement_Endo_seg003_a2 = (Radial_Displacement_Endo_seg003_point3 - Radial_Displacement_Endo_seg003_point2)/Time_duration2

Radial_Displacement_Endo_seg003_a3 = (Radial_Displacement_Endo_seg003_point4 - Radial_Displacement_Endo_seg003_point3)/Time_duration3

Radial_Displacement_Endo_seg003_a4 = (Radial_Displacement_Endo_seg003_point5 - Radial_Displacement_Endo_seg003_point4)/Time_duration4

Radial_Displacement_Endo_seg003_a_min = min(c(Radial_Displacement_Endo_seg003_a1,Radial_Displacement_Endo_seg003_a2,Radial_Displacement_Endo_seg003_a3,Radial_Displacement_Endo_seg003_a4))

Radial_Displacement_Endo_seg003_a_max = max(c(Radial_Displacement_Endo_seg003_a1,Radial_Displacement_Endo_seg003_a2,Radial_Displacement_Endo_seg003_a3,Radial_Displacement_Endo_seg003_a4))

Radial_Displacement_Endo_seg004_point1 = as.numeric(Displacement_data[11,col1])

Radial_Displacement_Endo_seg004_point2 = as.numeric(Displacement_data[11,col2])

Radial_Displacement_Endo_seg004_point3 = as.numeric(Displacement_data[11,col3])

Radial_Displacement_Endo_seg004_point4 = as.numeric(Displacement_data[11,col4])

Radial_Displacement_Endo_seg004_point5 = as.numeric(Displacement_data[11,col5])

Radial_Displacement_Endo_seg004_a1 = (Radial_Displacement_Endo_seg004_point2 - Radial_Displacement_Endo_seg004_point1)/Time_duration1

Radial_Displacement_Endo_seg004_a2 = (Radial_Displacement_Endo_seg004_point3 - Radial_Displacement_Endo_seg004_point2)/Time_duration2

Radial_Displacement_Endo_seg004_a3 = (Radial_Displacement_Endo_seg004_point4 - Radial_Displacement_Endo_seg004_point3)/Time_duration3

Radial_Displacement_Endo_seg004_a4 = (Radial_Displacement_Endo_seg004_point5 - Radial_Displacement_Endo_seg004_point4)/Time_duration4

Radial_Displacement_Endo_seg004_a_min = min(c(Radial_Displacement_Endo_seg004_a1,Radial_Displacement_Endo_seg004_a2,Radial_Displacement_Endo_seg004_a3,Radial_Displacement_Endo_seg004_a4))

Radial_Displacement_Endo_seg004_a_max = max(c(Radial_Displacement_Endo_seg004_a1,Radial_Displacement_Endo_seg004_a2,Radial_Displacement_Endo_seg004_a3,Radial_Displacement_Endo_seg004_a4))

Radial_Displacement_Endo_seg005_point1 = as.numeric(Displacement_data[12,col1])

Radial_Displacement_Endo_seg005_point2 = as.numeric(Displacement_data[12,col2])

Radial_Displacement_Endo_seg005_point3 = as.numeric(Displacement_data[12,col3])

Radial_Displacement_Endo_seg005_point4 = as.numeric(Displacement_data[12,col4])

Radial_Displacement_Endo_seg005_point5 = as.numeric(Displacement_data[12,col5])

Radial_Displacement_Endo_seg005_a1 = (Radial_Displacement_Endo_seg005_point2 - Radial_Displacement_Endo_seg005_point1)/Time_duration1

Radial_Displacement_Endo_seg005_a2 = (Radial_Displacement_Endo_seg005_point3 - Radial_Displacement_Endo_seg005_point2)/Time_duration2

Radial_Displacement_Endo_seg005_a3 = (Radial_Displacement_Endo_seg005_point4 - Radial_Displacement_Endo_seg005_point3)/Time_duration3

Radial_Displacement_Endo_seg005_a4 = (Radial_Displacement_Endo_seg005_point5 - Radial_Displacement_Endo_seg005_point4)/Time_duration4

Radial_Displacement_Endo_seg005_a_min = min(c(Radial_Displacement_Endo_seg005_a1,Radial_Displacement_Endo_seg005_a2,Radial_Displacement_Endo_seg005_a3,Radial_Displacement_Endo_seg005_a4))

Radial_Displacement_Endo_seg005_a_max = max(c(Radial_Displacement_Endo_seg005_a1,Radial_Displacement_Endo_seg005_a2,Radial_Displacement_Endo_seg005_a3,Radial_Displacement_Endo_seg005_a4))

Radial_Displacement_Endo_seg006_point1 = as.numeric(Displacement_data[13,col1])

Radial_Displacement_Endo_seg006_point2 = as.numeric(Displacement_data[13,col2])

Radial_Displacement_Endo_seg006_point3 = as.numeric(Displacement_data[13,col3])

Radial_Displacement_Endo_seg006_point4 = as.numeric(Displacement_data[13,col4])

Radial_Displacement_Endo_seg006_point5 = as.numeric(Displacement_data[13,col5])

Radial_Displacement_Endo_seg006_a1 = (Radial_Displacement_Endo_seg006_point2 - Radial_Displacement_Endo_seg006_point1)/Time_duration1

Radial_Displacement_Endo_seg006_a2 = (Radial_Displacement_Endo_seg006_point3 - Radial_Displacement_Endo_seg006_point2)/Time_duration2

Radial_Displacement_Endo_seg006_a3 = (Radial_Displacement_Endo_seg006_point4 - Radial_Displacement_Endo_seg006_point3)/Time_duration3

Radial_Displacement_Endo_seg006_a4 = (Radial_Displacement_Endo_seg006_point5 - Radial_Displacement_Endo_seg006_point4)/Time_duration4

Radial_Displacement_Endo_seg006_a_min = min(c(Radial_Displacement_Endo_seg006_a1,Radial_Displacement_Endo_seg006_a2,Radial_Displacement_Endo_seg006_a3,Radial_Displacement_Endo_seg006_a4))

Radial_Displacement_Endo_seg006_a_max = max(c(Radial_Displacement_Endo_seg006_a1,Radial_Displacement_Endo_seg006_a2,Radial_Displacement_Endo_seg006_a3,Radial_Displacement_Endo_seg006_a4))

##Average_values_per_frame_Radial_Displacement_Endo

Average_values_per_frame_Radial_Displacement_Endo_point1 = as.numeric(Displacement_data[17,col1])

Average_values_per_frame_Radial_Displacement_Endo_point2 = as.numeric(Displacement_data[17,col2])

Average_values_per_frame_Radial_Displacement_Endo_point3 = as.numeric(Displacement_data[17,col3])

Average_values_per_frame_Radial_Displacement_Endo_point4 = as.numeric(Displacement_data[17,col4])

Average_values_per_frame_Radial_Displacement_Endo_point5 = as.numeric(Displacement_data[17,col5])

Average_values_per_frame_Radial_Displacement_Endo_a1 = (Average_values_per_frame_Radial_Displacement_Endo_point2 - Average_values_per_frame_Radial_Displacement_Endo_point1)/Time_duration1

Average_values_per_frame_Radial_Displacement_Endo_a2 = (Average_values_per_frame_Radial_Displacement_Endo_point3 - Average_values_per_frame_Radial_Displacement_Endo_point2)/Time_duration2

Average_values_per_frame_Radial_Displacement_Endo_a3 = (Average_values_per_frame_Radial_Displacement_Endo_point4 - Average_values_per_frame_Radial_Displacement_Endo_point3)/Time_duration3

Average_values_per_frame_Radial_Displacement_Endo_a4 = (Average_values_per_frame_Radial_Displacement_Endo_point5 - Average_values_per_frame_Radial_Displacement_Endo_point4)/Time_duration4

Average_values_per_frame_Radial_Displacement_Endo_a_min = min(c(Average_values_per_frame_Radial_Displacement_Endo_a1,Average_values_per_frame_Radial_Displacement_Endo_a2,Average_values_per_frame_Radial_Displacement_Endo_a3,Average_values_per_frame_Radial_Displacement_Endo_a4))

Average_values_per_frame_Radial_Displacement_Endo_a_max = max(c(Average_values_per_frame_Radial_Displacement_Endo_a1,Average_values_per_frame_Radial_Displacement_Endo_a2,Average_values_per_frame_Radial_Displacement_Endo_a3,Average_values_per_frame_Radial_Displacement_Endo_a4))

##Longitudinal_Displacement_Endo

Longitudinal_Displacement_Endo_seg001_point1 = as.numeric(Displacement_data[21,col1])

Longitudinal_Displacement_Endo_seg001_point2 = as.numeric(Displacement_data[21,col2])

Longitudinal_Displacement_Endo_seg001_point3 = as.numeric(Displacement_data[21,col3])

Longitudinal_Displacement_Endo_seg001_point4 = as.numeric(Displacement_data[21,col4])

Longitudinal_Displacement_Endo_seg001_point5 = as.numeric(Displacement_data[21,col5])

Longitudinal_Displacement_Endo_seg001_a1 = (Longitudinal_Displacement_Endo_seg001_point2 - Longitudinal_Displacement_Endo_seg001_point1)/Time_duration1

Longitudinal_Displacement_Endo_seg001_a2 = (Longitudinal_Displacement_Endo_seg001_point3 - Longitudinal_Displacement_Endo_seg001_point2)/Time_duration2

Longitudinal_Displacement_Endo_seg001_a3 = (Longitudinal_Displacement_Endo_seg001_point4 - Longitudinal_Displacement_Endo_seg001_point3)/Time_duration3

Longitudinal_Displacement_Endo_seg001_a4 = (Longitudinal_Displacement_Endo_seg001_point5 - Longitudinal_Displacement_Endo_seg001_point4)/Time_duration4

Longitudinal_Displacement_Endo_seg001_a_min = min(c(Longitudinal_Displacement_Endo_seg001_a1,Longitudinal_Displacement_Endo_seg001_a2,Longitudinal_Displacement_Endo_seg001_a3,Longitudinal_Displacement_Endo_seg001_a4))

Longitudinal_Displacement_Endo_seg001_a_max = max(c(Longitudinal_Displacement_Endo_seg001_a1,Longitudinal_Displacement_Endo_seg001_a2,Longitudinal_Displacement_Endo_seg001_a3,Longitudinal_Displacement_Endo_seg001_a4))

Longitudinal_Displacement_Endo_seg002_point1 = as.numeric(Displacement_data[22,col1])

Longitudinal_Displacement_Endo_seg002_point2 = as.numeric(Displacement_data[22,col2])

Longitudinal_Displacement_Endo_seg002_point3 = as.numeric(Displacement_data[22,col3])

Longitudinal_Displacement_Endo_seg002_point4 = as.numeric(Displacement_data[22,col4])

Longitudinal_Displacement_Endo_seg002_point5 = as.numeric(Displacement_data[22,col5])

Longitudinal_Displacement_Endo_seg002_a1 = (Longitudinal_Displacement_Endo_seg002_point2 - Longitudinal_Displacement_Endo_seg002_point1)/Time_duration1

Longitudinal_Displacement_Endo_seg002_a2 = (Longitudinal_Displacement_Endo_seg002_point3 - Longitudinal_Displacement_Endo_seg002_point2)/Time_duration2

Longitudinal_Displacement_Endo_seg002_a3 = (Longitudinal_Displacement_Endo_seg002_point4 - Longitudinal_Displacement_Endo_seg002_point3)/Time_duration3

Longitudinal_Displacement_Endo_seg002_a4 = (Longitudinal_Displacement_Endo_seg002_point5 - Longitudinal_Displacement_Endo_seg002_point4)/Time_duration4

Longitudinal_Displacement_Endo_seg002_a_min = min(c(Longitudinal_Displacement_Endo_seg002_a1,Longitudinal_Displacement_Endo_seg002_a2,Longitudinal_Displacement_Endo_seg002_a3,Longitudinal_Displacement_Endo_seg002_a4))

Longitudinal_Displacement_Endo_seg002_a_max = max(c(Longitudinal_Displacement_Endo_seg002_a1,Longitudinal_Displacement_Endo_seg002_a2,Longitudinal_Displacement_Endo_seg002_a3,Longitudinal_Displacement_Endo_seg002_a4))

Longitudinal_Displacement_Endo_seg003_point1 = as.numeric(Displacement_data[23,col1])

Longitudinal_Displacement_Endo_seg003_point2 = as.numeric(Displacement_data[23,col2])

Longitudinal_Displacement_Endo_seg003_point3 = as.numeric(Displacement_data[23,col3])

Longitudinal_Displacement_Endo_seg003_point4 = as.numeric(Displacement_data[23,col4])

Longitudinal_Displacement_Endo_seg003_point5 = as.numeric(Displacement_data[23,col5])

Longitudinal_Displacement_Endo_seg003_a1 = (Longitudinal_Displacement_Endo_seg003_point2 - Longitudinal_Displacement_Endo_seg003_point1)/Time_duration1

Longitudinal_Displacement_Endo_seg003_a2 = (Longitudinal_Displacement_Endo_seg003_point3 - Longitudinal_Displacement_Endo_seg003_point2)/Time_duration2

Longitudinal_Displacement_Endo_seg003_a3 = (Longitudinal_Displacement_Endo_seg003_point4 - Longitudinal_Displacement_Endo_seg003_point3)/Time_duration3

Longitudinal_Displacement_Endo_seg003_a4 = (Longitudinal_Displacement_Endo_seg003_point5 - Longitudinal_Displacement_Endo_seg003_point4)/Time_duration4

Longitudinal_Displacement_Endo_seg003_a_min = min(c(Longitudinal_Displacement_Endo_seg003_a1,Longitudinal_Displacement_Endo_seg003_a2,Longitudinal_Displacement_Endo_seg003_a3,Longitudinal_Displacement_Endo_seg003_a4))

Longitudinal_Displacement_Endo_seg003_a_max = max(c(Longitudinal_Displacement_Endo_seg003_a1,Longitudinal_Displacement_Endo_seg003_a2,Longitudinal_Displacement_Endo_seg003_a3,Longitudinal_Displacement_Endo_seg003_a4))

Longitudinal_Displacement_Endo_seg004_point1 = as.numeric(Displacement_data[24,col1])

Longitudinal_Displacement_Endo_seg004_point2 = as.numeric(Displacement_data[24,col2])

Longitudinal_Displacement_Endo_seg004_point3 = as.numeric(Displacement_data[24,col3])

Longitudinal_Displacement_Endo_seg004_point4 = as.numeric(Displacement_data[24,col4])

Longitudinal_Displacement_Endo_seg004_point5 = as.numeric(Displacement_data[24,col5])

Longitudinal_Displacement_Endo_seg004_a1 = (Longitudinal_Displacement_Endo_seg004_point2 - Longitudinal_Displacement_Endo_seg004_point1)/Time_duration1

Longitudinal_Displacement_Endo_seg004_a2 = (Longitudinal_Displacement_Endo_seg004_point3 - Longitudinal_Displacement_Endo_seg004_point2)/Time_duration2

Longitudinal_Displacement_Endo_seg004_a3 = (Longitudinal_Displacement_Endo_seg004_point4 - Longitudinal_Displacement_Endo_seg004_point3)/Time_duration3

Longitudinal_Displacement_Endo_seg004_a4 = (Longitudinal_Displacement_Endo_seg004_point5 - Longitudinal_Displacement_Endo_seg004_point4)/Time_duration4

Longitudinal_Displacement_Endo_seg004_a_min = min(c(Longitudinal_Displacement_Endo_seg004_a1,Longitudinal_Displacement_Endo_seg004_a2,Longitudinal_Displacement_Endo_seg004_a3,Longitudinal_Displacement_Endo_seg004_a4))

Longitudinal_Displacement_Endo_seg004_a_max = max(c(Longitudinal_Displacement_Endo_seg004_a1,Longitudinal_Displacement_Endo_seg004_a2,Longitudinal_Displacement_Endo_seg004_a3,Longitudinal_Displacement_Endo_seg004_a4))

Longitudinal_Displacement_Endo_seg005_point1 = as.numeric(Displacement_data[25,col1])

Longitudinal_Displacement_Endo_seg005_point2 = as.numeric(Displacement_data[25,col2])

Longitudinal_Displacement_Endo_seg005_point3 = as.numeric(Displacement_data[25,col3])

Longitudinal_Displacement_Endo_seg005_point4 = as.numeric(Displacement_data[25,col4])

Longitudinal_Displacement_Endo_seg005_point5 = as.numeric(Displacement_data[25,col5])

Longitudinal_Displacement_Endo_seg005_a1 = (Longitudinal_Displacement_Endo_seg005_point2 - Longitudinal_Displacement_Endo_seg005_point1)/Time_duration1

Longitudinal_Displacement_Endo_seg005_a2 = (Longitudinal_Displacement_Endo_seg005_point3 - Longitudinal_Displacement_Endo_seg005_point2)/Time_duration2

Longitudinal_Displacement_Endo_seg005_a3 = (Longitudinal_Displacement_Endo_seg005_point4 - Longitudinal_Displacement_Endo_seg005_point3)/Time_duration3

Longitudinal_Displacement_Endo_seg005_a4 = (Longitudinal_Displacement_Endo_seg005_point5 - Longitudinal_Displacement_Endo_seg005_point4)/Time_duration4

Longitudinal_Displacement_Endo_seg005_a_min = min(c(Longitudinal_Displacement_Endo_seg005_a1,Longitudinal_Displacement_Endo_seg005_a2,Longitudinal_Displacement_Endo_seg005_a3,Longitudinal_Displacement_Endo_seg005_a4))

Longitudinal_Displacement_Endo_seg005_a_max = max(c(Longitudinal_Displacement_Endo_seg005_a1,Longitudinal_Displacement_Endo_seg005_a2,Longitudinal_Displacement_Endo_seg005_a3,Longitudinal_Displacement_Endo_seg005_a4))

Longitudinal_Displacement_Endo_seg006_point1 = as.numeric(Displacement_data[26,col1])

Longitudinal_Displacement_Endo_seg006_point2 = as.numeric(Displacement_data[26,col2])

Longitudinal_Displacement_Endo_seg006_point3 = as.numeric(Displacement_data[26,col3])

Longitudinal_Displacement_Endo_seg006_point4 = as.numeric(Displacement_data[26,col4])

Longitudinal_Displacement_Endo_seg006_point5 = as.numeric(Displacement_data[26,col5])

Longitudinal_Displacement_Endo_seg006_a1 = (Longitudinal_Displacement_Endo_seg006_point2 - Longitudinal_Displacement_Endo_seg006_point1)/Time_duration1

Longitudinal_Displacement_Endo_seg006_a2 = (Longitudinal_Displacement_Endo_seg006_point3 - Longitudinal_Displacement_Endo_seg006_point2)/Time_duration2

Longitudinal_Displacement_Endo_seg006_a3 = (Longitudinal_Displacement_Endo_seg006_point4 - Longitudinal_Displacement_Endo_seg006_point3)/Time_duration3

Longitudinal_Displacement_Endo_seg006_a4 = (Longitudinal_Displacement_Endo_seg006_point5 - Longitudinal_Displacement_Endo_seg006_point4)/Time_duration4

Longitudinal_Displacement_Endo_seg006_a_min = min(c(Longitudinal_Displacement_Endo_seg006_a1,Longitudinal_Displacement_Endo_seg006_a2,Longitudinal_Displacement_Endo_seg006_a3,Longitudinal_Displacement_Endo_seg006_a4))

Longitudinal_Displacement_Endo_seg006_a_max = max(c(Longitudinal_Displacement_Endo_seg006_a1,Longitudinal_Displacement_Endo_seg006_a2,Longitudinal_Displacement_Endo_seg006_a3,Longitudinal_Displacement_Endo_seg006_a4))

##Average_values_per_frame_Longitudinal_Displacement_Endo

Average_values_per_frame_Longitudinal_Displacement_Endo_point1 = as.numeric(Displacement_data[30,col1])

Average_values_per_frame_Longitudinal_Displacement_Endo_point2 = as.numeric(Displacement_data[30,col2])

Average_values_per_frame_Longitudinal_Displacement_Endo_point3 = as.numeric(Displacement_data[30,col3])

Average_values_per_frame_Longitudinal_Displacement_Endo_point4 = as.numeric(Displacement_data[30,col4])

Average_values_per_frame_Longitudinal_Displacement_Endo_point5 = as.numeric(Displacement_data[30,col5])

Average_values_per_frame_Longitudinal_Displacement_Endo_a1 = (Average_values_per_frame_Longitudinal_Displacement_Endo_point2 - Average_values_per_frame_Longitudinal_Displacement_Endo_point1)/Time_duration1

Average_values_per_frame_Longitudinal_Displacement_Endo_a2 = (Average_values_per_frame_Longitudinal_Displacement_Endo_point3 - Average_values_per_frame_Longitudinal_Displacement_Endo_point2)/Time_duration2

Average_values_per_frame_Longitudinal_Displacement_Endo_a3 = (Average_values_per_frame_Longitudinal_Displacement_Endo_point4 - Average_values_per_frame_Longitudinal_Displacement_Endo_point3)/Time_duration3

Average_values_per_frame_Longitudinal_Displacement_Endo_a4 = (Average_values_per_frame_Longitudinal_Displacement_Endo_point5 - Average_values_per_frame_Longitudinal_Displacement_Endo_point4)/Time_duration4

Average_values_per_frame_Longitudinal_Displacement_Endo_a_min = min(c(Average_values_per_frame_Longitudinal_Displacement_Endo_a1,Average_values_per_frame_Longitudinal_Displacement_Endo_a2,Average_values_per_frame_Longitudinal_Displacement_Endo_a3,Average_values_per_frame_Longitudinal_Displacement_Endo_a4))

Average_values_per_frame_Longitudinal_Displacement_Endo_a_max = max(c(Average_values_per_frame_Longitudinal_Displacement_Endo_a1,Average_values_per_frame_Longitudinal_Displacement_Endo_a2,Average_values_per_frame_Longitudinal_Displacement_Endo_a3,Average_values_per_frame_Longitudinal_Displacement_Endo_a4))

##Radial_Displacement_Epi

Radial_Displacement_Epi_seg001_point1 = as.numeric(Displacement_data[34,col1])

Radial_Displacement_Epi_seg001_point2 = as.numeric(Displacement_data[34,col2])

Radial_Displacement_Epi_seg001_point3 = as.numeric(Displacement_data[34,col3])

Radial_Displacement_Epi_seg001_point4 = as.numeric(Displacement_data[34,col4])

Radial_Displacement_Epi_seg001_point5 = as.numeric(Displacement_data[34,col5])

Radial_Displacement_Epi_seg001_a1 = (Radial_Displacement_Epi_seg001_point2 - Radial_Displacement_Epi_seg001_point1)/Time_duration1

Radial_Displacement_Epi_seg001_a2 = (Radial_Displacement_Epi_seg001_point3 - Radial_Displacement_Epi_seg001_point2)/Time_duration2

Radial_Displacement_Epi_seg001_a3 = (Radial_Displacement_Epi_seg001_point4 - Radial_Displacement_Epi_seg001_point3)/Time_duration3

Radial_Displacement_Epi_seg001_a4 = (Radial_Displacement_Epi_seg001_point5 - Radial_Displacement_Epi_seg001_point4)/Time_duration4

Radial_Displacement_Epi_seg001_a_min = min(c(Radial_Displacement_Epi_seg001_a1,Radial_Displacement_Epi_seg001_a2,Radial_Displacement_Epi_seg001_a3,Radial_Displacement_Epi_seg001_a4))

Radial_Displacement_Epi_seg001_a_max = max(c(Radial_Displacement_Epi_seg001_a1,Radial_Displacement_Epi_seg001_a2,Radial_Displacement_Epi_seg001_a3,Radial_Displacement_Epi_seg001_a4))

Radial_Displacement_Epi_seg002_point1 = as.numeric(Displacement_data[35,col1])

Radial_Displacement_Epi_seg002_point2 = as.numeric(Displacement_data[35,col2])

Radial_Displacement_Epi_seg002_point3 = as.numeric(Displacement_data[35,col3])

Radial_Displacement_Epi_seg002_point4 = as.numeric(Displacement_data[35,col4])

Radial_Displacement_Epi_seg002_point5 = as.numeric(Displacement_data[35,col5])

Radial_Displacement_Epi_seg002_a1 = (Radial_Displacement_Epi_seg002_point2 - Radial_Displacement_Epi_seg002_point1)/Time_duration1

Radial_Displacement_Epi_seg002_a2 = (Radial_Displacement_Epi_seg002_point3 - Radial_Displacement_Epi_seg002_point2)/Time_duration2

Radial_Displacement_Epi_seg002_a3 = (Radial_Displacement_Epi_seg002_point4 - Radial_Displacement_Epi_seg002_point3)/Time_duration3

Radial_Displacement_Epi_seg002_a4 = (Radial_Displacement_Epi_seg002_point5 - Radial_Displacement_Epi_seg002_point4)/Time_duration4

Radial_Displacement_Epi_seg002_a_min = min(c(Radial_Displacement_Epi_seg002_a1,Radial_Displacement_Epi_seg002_a2,Radial_Displacement_Epi_seg002_a3,Radial_Displacement_Epi_seg002_a4))

Radial_Displacement_Epi_seg002_a_max = max(c(Radial_Displacement_Epi_seg002_a1,Radial_Displacement_Epi_seg002_a2,Radial_Displacement_Epi_seg002_a3,Radial_Displacement_Epi_seg002_a4))

Radial_Displacement_Epi_seg003_point1 = as.numeric(Displacement_data[36,col1])

Radial_Displacement_Epi_seg003_point2 = as.numeric(Displacement_data[36,col2])

Radial_Displacement_Epi_seg003_point3 = as.numeric(Displacement_data[36,col3])

Radial_Displacement_Epi_seg003_point4 = as.numeric(Displacement_data[36,col4])

Radial_Displacement_Epi_seg003_point5 = as.numeric(Displacement_data[36,col5])

Radial_Displacement_Epi_seg003_a1 = (Radial_Displacement_Epi_seg003_point2 - Radial_Displacement_Epi_seg003_point1)/Time_duration1

Radial_Displacement_Epi_seg003_a2 = (Radial_Displacement_Epi_seg003_point3 - Radial_Displacement_Epi_seg003_point2)/Time_duration2

Radial_Displacement_Epi_seg003_a3 = (Radial_Displacement_Epi_seg003_point4 - Radial_Displacement_Epi_seg003_point3)/Time_duration3

Radial_Displacement_Epi_seg003_a4 = (Radial_Displacement_Epi_seg003_point5 - Radial_Displacement_Epi_seg003_point4)/Time_duration4

Radial_Displacement_Epi_seg003_a_min = min(c(Radial_Displacement_Epi_seg003_a1,Radial_Displacement_Epi_seg003_a2,Radial_Displacement_Epi_seg003_a3,Radial_Displacement_Epi_seg003_a4))

Radial_Displacement_Epi_seg003_a_max = max(c(Radial_Displacement_Epi_seg003_a1,Radial_Displacement_Epi_seg003_a2,Radial_Displacement_Epi_seg003_a3,Radial_Displacement_Epi_seg003_a4))

Radial_Displacement_Epi_seg004_point1 = as.numeric(Displacement_data[37,col1])

Radial_Displacement_Epi_seg004_point2 = as.numeric(Displacement_data[37,col2])

Radial_Displacement_Epi_seg004_point3 = as.numeric(Displacement_data[37,col3])

Radial_Displacement_Epi_seg004_point4 = as.numeric(Displacement_data[37,col4])

Radial_Displacement_Epi_seg004_point5 = as.numeric(Displacement_data[37,col5])

Radial_Displacement_Epi_seg004_a1 = (Radial_Displacement_Epi_seg004_point2 - Radial_Displacement_Epi_seg004_point1)/Time_duration1

Radial_Displacement_Epi_seg004_a2 = (Radial_Displacement_Epi_seg004_point3 - Radial_Displacement_Epi_seg004_point2)/Time_duration2

Radial_Displacement_Epi_seg004_a3 = (Radial_Displacement_Epi_seg004_point4 - Radial_Displacement_Epi_seg004_point3)/Time_duration3

Radial_Displacement_Epi_seg004_a4 = (Radial_Displacement_Epi_seg004_point5 - Radial_Displacement_Epi_seg004_point4)/Time_duration4

Radial_Displacement_Epi_seg004_a_min = min(c(Radial_Displacement_Epi_seg004_a1,Radial_Displacement_Epi_seg004_a2,Radial_Displacement_Epi_seg004_a3,Radial_Displacement_Epi_seg004_a4))

Radial_Displacement_Epi_seg004_a_max = max(c(Radial_Displacement_Epi_seg004_a1,Radial_Displacement_Epi_seg004_a2,Radial_Displacement_Epi_seg004_a3,Radial_Displacement_Epi_seg004_a4))

Radial_Displacement_Epi_seg005_point1 = as.numeric(Displacement_data[38,col1])

Radial_Displacement_Epi_seg005_point2 = as.numeric(Displacement_data[38,col2])

Radial_Displacement_Epi_seg005_point3 = as.numeric(Displacement_data[38,col3])

Radial_Displacement_Epi_seg005_point4 = as.numeric(Displacement_data[38,col4])

Radial_Displacement_Epi_seg005_point5 = as.numeric(Displacement_data[38,col5])

Radial_Displacement_Epi_seg005_a1 = (Radial_Displacement_Epi_seg005_point2 - Radial_Displacement_Epi_seg005_point1)/Time_duration1

Radial_Displacement_Epi_seg005_a2 = (Radial_Displacement_Epi_seg005_point3 - Radial_Displacement_Epi_seg005_point2)/Time_duration2

Radial_Displacement_Epi_seg005_a3 = (Radial_Displacement_Epi_seg005_point4 - Radial_Displacement_Epi_seg005_point3)/Time_duration3

Radial_Displacement_Epi_seg005_a4 = (Radial_Displacement_Epi_seg005_point5 - Radial_Displacement_Epi_seg005_point4)/Time_duration4

Radial_Displacement_Epi_seg005_a_min = min(c(Radial_Displacement_Epi_seg005_a1,Radial_Displacement_Epi_seg005_a2,Radial_Displacement_Epi_seg005_a3,Radial_Displacement_Epi_seg005_a4))

Radial_Displacement_Epi_seg005_a_max = max(c(Radial_Displacement_Epi_seg005_a1,Radial_Displacement_Epi_seg005_a2,Radial_Displacement_Epi_seg005_a3,Radial_Displacement_Epi_seg005_a4))

Radial_Displacement_Epi_seg006_point1 = as.numeric(Displacement_data[39,col1])

Radial_Displacement_Epi_seg006_point2 = as.numeric(Displacement_data[39,col2])

Radial_Displacement_Epi_seg006_point3 = as.numeric(Displacement_data[39,col3])

Radial_Displacement_Epi_seg006_point4 = as.numeric(Displacement_data[39,col4])

Radial_Displacement_Epi_seg006_point5 = as.numeric(Displacement_data[39,col5])

Radial_Displacement_Epi_seg006_a1 = (Radial_Displacement_Epi_seg006_point2 - Radial_Displacement_Epi_seg006_point1)/Time_duration1

Radial_Displacement_Epi_seg006_a2 = (Radial_Displacement_Epi_seg006_point3 - Radial_Displacement_Epi_seg006_point2)/Time_duration2

Radial_Displacement_Epi_seg006_a3 = (Radial_Displacement_Epi_seg006_point4 - Radial_Displacement_Epi_seg006_point3)/Time_duration3

Radial_Displacement_Epi_seg006_a4 = (Radial_Displacement_Epi_seg006_point5 - Radial_Displacement_Epi_seg006_point4)/Time_duration4

Radial_Displacement_Epi_seg006_a_min = min(c(Radial_Displacement_Epi_seg006_a1,Radial_Displacement_Epi_seg006_a2,Radial_Displacement_Epi_seg006_a3,Radial_Displacement_Epi_seg006_a4))

Radial_Displacement_Epi_seg006_a_max = max(c(Radial_Displacement_Epi_seg006_a1,Radial_Displacement_Epi_seg006_a2,Radial_Displacement_Epi_seg006_a3,Radial_Displacement_Epi_seg006_a4))

##Average_values_per_frame_Radial_Displacement_Epi

Average_values_per_frame_Radial_Displacement_Epi_point1 = as.numeric(Displacement_data[43,col1])

Average_values_per_frame_Radial_Displacement_Epi_point2 = as.numeric(Displacement_data[43,col2])

Average_values_per_frame_Radial_Displacement_Epi_point3 = as.numeric(Displacement_data[43,col3])

Average_values_per_frame_Radial_Displacement_Epi_point4 = as.numeric(Displacement_data[43,col4])

Average_values_per_frame_Radial_Displacement_Epi_point5 = as.numeric(Displacement_data[43,col5])

Average_values_per_frame_Radial_Displacement_Epi_a1 = (Average_values_per_frame_Radial_Displacement_Epi_point2 - Average_values_per_frame_Radial_Displacement_Epi_point1)/Time_duration1

Average_values_per_frame_Radial_Displacement_Epi_a2 = (Average_values_per_frame_Radial_Displacement_Epi_point3 - Average_values_per_frame_Radial_Displacement_Epi_point2)/Time_duration2

Average_values_per_frame_Radial_Displacement_Epi_a3 = (Average_values_per_frame_Radial_Displacement_Epi_point4 - Average_values_per_frame_Radial_Displacement_Epi_point3)/Time_duration3

Average_values_per_frame_Radial_Displacement_Epi_a4 = (Average_values_per_frame_Radial_Displacement_Epi_point5 - Average_values_per_frame_Radial_Displacement_Epi_point4)/Time_duration4

Average_values_per_frame_Radial_Displacement_Epi_a_min = min(c(Average_values_per_frame_Radial_Displacement_Epi_a1,Average_values_per_frame_Radial_Displacement_Epi_a2,Average_values_per_frame_Radial_Displacement_Epi_a3,Average_values_per_frame_Radial_Displacement_Epi_a4))

Average_values_per_frame_Radial_Displacement_Epi_a_max = max(c(Average_values_per_frame_Radial_Displacement_Epi_a1,Average_values_per_frame_Radial_Displacement_Epi_a2,Average_values_per_frame_Radial_Displacement_Epi_a3,Average_values_per_frame_Radial_Displacement_Epi_a4))

##Longitudinal_Displacement_Epi

Longitudinal_Displacement_Epi_seg001_point1 = as.numeric(Displacement_data[47,col1])

Longitudinal_Displacement_Epi_seg001_point2 = as.numeric(Displacement_data[47,col2])

Longitudinal_Displacement_Epi_seg001_point3 = as.numeric(Displacement_data[47,col3])

Longitudinal_Displacement_Epi_seg001_point4 = as.numeric(Displacement_data[47,col4])

Longitudinal_Displacement_Epi_seg001_point5 = as.numeric(Displacement_data[47,col5])

Longitudinal_Displacement_Epi_seg001_a1 = (Longitudinal_Displacement_Epi_seg001_point2 - Longitudinal_Displacement_Epi_seg001_point1)/Time_duration1

Longitudinal_Displacement_Epi_seg001_a2 = (Longitudinal_Displacement_Epi_seg001_point3 - Longitudinal_Displacement_Epi_seg001_point2)/Time_duration2

Longitudinal_Displacement_Epi_seg001_a3 = (Longitudinal_Displacement_Epi_seg001_point4 - Longitudinal_Displacement_Epi_seg001_point3)/Time_duration3

Longitudinal_Displacement_Epi_seg001_a4 = (Longitudinal_Displacement_Epi_seg001_point5 - Longitudinal_Displacement_Epi_seg001_point4)/Time_duration4

Longitudinal_Displacement_Epi_seg001_a_min = min(c(Longitudinal_Displacement_Epi_seg001_a1,Longitudinal_Displacement_Epi_seg001_a2,Longitudinal_Displacement_Epi_seg001_a3,Longitudinal_Displacement_Epi_seg001_a4))

Longitudinal_Displacement_Epi_seg001_a_max = max(c(Longitudinal_Displacement_Epi_seg001_a1,Longitudinal_Displacement_Epi_seg001_a2,Longitudinal_Displacement_Epi_seg001_a3,Longitudinal_Displacement_Epi_seg001_a4))

Longitudinal_Displacement_Epi_seg002_point1 = as.numeric(Displacement_data[48,col1])

Longitudinal_Displacement_Epi_seg002_point2 = as.numeric(Displacement_data[48,col2])

Longitudinal_Displacement_Epi_seg002_point3 = as.numeric(Displacement_data[48,col3])

Longitudinal_Displacement_Epi_seg002_point4 = as.numeric(Displacement_data[48,col4])

Longitudinal_Displacement_Epi_seg002_point5 = as.numeric(Displacement_data[48,col5])

Longitudinal_Displacement_Epi_seg002_a1 = (Longitudinal_Displacement_Epi_seg002_point2 - Longitudinal_Displacement_Epi_seg002_point1)/Time_duration1

Longitudinal_Displacement_Epi_seg002_a2 = (Longitudinal_Displacement_Epi_seg002_point3 - Longitudinal_Displacement_Epi_seg002_point2)/Time_duration2

Longitudinal_Displacement_Epi_seg002_a3 = (Longitudinal_Displacement_Epi_seg002_point4 - Longitudinal_Displacement_Epi_seg002_point3)/Time_duration3

Longitudinal_Displacement_Epi_seg002_a4 = (Longitudinal_Displacement_Epi_seg002_point5 - Longitudinal_Displacement_Epi_seg002_point4)/Time_duration4

Longitudinal_Displacement_Epi_seg002_a_min = min(c(Longitudinal_Displacement_Epi_seg002_a1,Longitudinal_Displacement_Epi_seg002_a2,Longitudinal_Displacement_Epi_seg002_a3,Longitudinal_Displacement_Epi_seg002_a4))

Longitudinal_Displacement_Epi_seg002_a_max = max(c(Longitudinal_Displacement_Epi_seg002_a1,Longitudinal_Displacement_Epi_seg002_a2,Longitudinal_Displacement_Epi_seg002_a3,Longitudinal_Displacement_Epi_seg002_a4))

Longitudinal_Displacement_Epi_seg003_point1 = as.numeric(Displacement_data[49,col1])

Longitudinal_Displacement_Epi_seg003_point2 = as.numeric(Displacement_data[49,col2])

Longitudinal_Displacement_Epi_seg003_point3 = as.numeric(Displacement_data[49,col3])

Longitudinal_Displacement_Epi_seg003_point4 = as.numeric(Displacement_data[49,col4])

Longitudinal_Displacement_Epi_seg003_point5 = as.numeric(Displacement_data[49,col5])

Longitudinal_Displacement_Epi_seg003_a1 = (Longitudinal_Displacement_Epi_seg003_point2 - Longitudinal_Displacement_Epi_seg003_point1)/Time_duration1

Longitudinal_Displacement_Epi_seg003_a2 = (Longitudinal_Displacement_Epi_seg003_point3 - Longitudinal_Displacement_Epi_seg003_point2)/Time_duration2

Longitudinal_Displacement_Epi_seg003_a3 = (Longitudinal_Displacement_Epi_seg003_point4 - Longitudinal_Displacement_Epi_seg003_point3)/Time_duration3

Longitudinal_Displacement_Epi_seg003_a4 = (Longitudinal_Displacement_Epi_seg003_point5 - Longitudinal_Displacement_Epi_seg003_point4)/Time_duration4

Longitudinal_Displacement_Epi_seg003_a_min = min(c(Longitudinal_Displacement_Epi_seg003_a1,Longitudinal_Displacement_Epi_seg003_a2,Longitudinal_Displacement_Epi_seg003_a3,Longitudinal_Displacement_Epi_seg003_a4))

Longitudinal_Displacement_Epi_seg003_a_max = max(c(Longitudinal_Displacement_Epi_seg003_a1,Longitudinal_Displacement_Epi_seg003_a2,Longitudinal_Displacement_Epi_seg003_a3,Longitudinal_Displacement_Epi_seg003_a4))

Longitudinal_Displacement_Epi_seg004_point1 = as.numeric(Displacement_data[50,col1])

Longitudinal_Displacement_Epi_seg004_point2 = as.numeric(Displacement_data[50,col2])

Longitudinal_Displacement_Epi_seg004_point3 = as.numeric(Displacement_data[50,col3])

Longitudinal_Displacement_Epi_seg004_point4 = as.numeric(Displacement_data[50,col4])

Longitudinal_Displacement_Epi_seg004_point5 = as.numeric(Displacement_data[50,col5])

Longitudinal_Displacement_Epi_seg004_a1 = (Longitudinal_Displacement_Epi_seg004_point2 - Longitudinal_Displacement_Epi_seg004_point1)/Time_duration1

Longitudinal_Displacement_Epi_seg004_a2 = (Longitudinal_Displacement_Epi_seg004_point3 - Longitudinal_Displacement_Epi_seg004_point2)/Time_duration2

Longitudinal_Displacement_Epi_seg004_a3 = (Longitudinal_Displacement_Epi_seg004_point4 - Longitudinal_Displacement_Epi_seg004_point3)/Time_duration3

Longitudinal_Displacement_Epi_seg004_a4 = (Longitudinal_Displacement_Epi_seg004_point5 - Longitudinal_Displacement_Epi_seg004_point4)/Time_duration4

Longitudinal_Displacement_Epi_seg004_a_min = min(c(Longitudinal_Displacement_Epi_seg004_a1,Longitudinal_Displacement_Epi_seg004_a2,Longitudinal_Displacement_Epi_seg004_a3,Longitudinal_Displacement_Epi_seg004_a4))

Longitudinal_Displacement_Epi_seg004_a_max = max(c(Longitudinal_Displacement_Epi_seg004_a1,Longitudinal_Displacement_Epi_seg004_a2,Longitudinal_Displacement_Epi_seg004_a3,Longitudinal_Displacement_Epi_seg004_a4))

Longitudinal_Displacement_Epi_seg005_point1 = as.numeric(Displacement_data[51,col1])

Longitudinal_Displacement_Epi_seg005_point2 = as.numeric(Displacement_data[51,col2])

Longitudinal_Displacement_Epi_seg005_point3 = as.numeric(Displacement_data[51,col3])

Longitudinal_Displacement_Epi_seg005_point4 = as.numeric(Displacement_data[51,col4])

Longitudinal_Displacement_Epi_seg005_point5 = as.numeric(Displacement_data[51,col5])

Longitudinal_Displacement_Epi_seg005_a1 = (Longitudinal_Displacement_Epi_seg005_point2 - Longitudinal_Displacement_Epi_seg005_point1)/Time_duration1

Longitudinal_Displacement_Epi_seg005_a2 = (Longitudinal_Displacement_Epi_seg005_point3 - Longitudinal_Displacement_Epi_seg005_point2)/Time_duration2

Longitudinal_Displacement_Epi_seg005_a3 = (Longitudinal_Displacement_Epi_seg005_point4 - Longitudinal_Displacement_Epi_seg005_point3)/Time_duration3

Longitudinal_Displacement_Epi_seg005_a4 = (Longitudinal_Displacement_Epi_seg005_point5 - Longitudinal_Displacement_Epi_seg005_point4)/Time_duration4

Longitudinal_Displacement_Epi_seg005_a_min = min(c(Longitudinal_Displacement_Epi_seg005_a1,Longitudinal_Displacement_Epi_seg005_a2,Longitudinal_Displacement_Epi_seg005_a3,Longitudinal_Displacement_Epi_seg005_a4))

Longitudinal_Displacement_Epi_seg005_a_max = max(c(Longitudinal_Displacement_Epi_seg005_a1,Longitudinal_Displacement_Epi_seg005_a2,Longitudinal_Displacement_Epi_seg005_a3,Longitudinal_Displacement_Epi_seg005_a4))

Longitudinal_Displacement_Epi_seg006_point1 = as.numeric(Displacement_data[52,col1])

Longitudinal_Displacement_Epi_seg006_point2 = as.numeric(Displacement_data[52,col2])

Longitudinal_Displacement_Epi_seg006_point3 = as.numeric(Displacement_data[52,col3])

Longitudinal_Displacement_Epi_seg006_point4 = as.numeric(Displacement_data[52,col4])

Longitudinal_Displacement_Epi_seg006_point5 = as.numeric(Displacement_data[52,col5])

Longitudinal_Displacement_Epi_seg006_a1 = (Longitudinal_Displacement_Epi_seg006_point2 - Longitudinal_Displacement_Epi_seg006_point1)/Time_duration1

Longitudinal_Displacement_Epi_seg006_a2 = (Longitudinal_Displacement_Epi_seg006_point3 - Longitudinal_Displacement_Epi_seg006_point2)/Time_duration2

Longitudinal_Displacement_Epi_seg006_a3 = (Longitudinal_Displacement_Epi_seg006_point4 - Longitudinal_Displacement_Epi_seg006_point3)/Time_duration3

Longitudinal_Displacement_Epi_seg006_a4 = (Longitudinal_Displacement_Epi_seg006_point5 - Longitudinal_Displacement_Epi_seg006_point4)/Time_duration4

Longitudinal_Displacement_Epi_seg006_a_min = min(c(Longitudinal_Displacement_Epi_seg006_a1,Longitudinal_Displacement_Epi_seg006_a2,Longitudinal_Displacement_Epi_seg006_a3,Longitudinal_Displacement_Epi_seg006_a4))

Longitudinal_Displacement_Epi_seg006_a_max = max(c(Longitudinal_Displacement_Epi_seg006_a1,Longitudinal_Displacement_Epi_seg006_a2,Longitudinal_Displacement_Epi_seg006_a3,Longitudinal_Displacement_Epi_seg006_a4))

##Average_values_per_frame_Longitudinal_Displacement_Epi

Average_values_per_frame_Longitudinal_Displacement_Epi_point1 = as.numeric(Displacement_data[56,col1])

Average_values_per_frame_Longitudinal_Displacement_Epi_point2 = as.numeric(Displacement_data[56,col2])

Average_values_per_frame_Longitudinal_Displacement_Epi_point3 = as.numeric(Displacement_data[56,col3])

Average_values_per_frame_Longitudinal_Displacement_Epi_point4 = as.numeric(Displacement_data[56,col4])

Average_values_per_frame_Longitudinal_Displacement_Epi_point5 = as.numeric(Displacement_data[56,col5])

Average_values_per_frame_Longitudinal_Displacement_Epi_a1 = (Average_values_per_frame_Longitudinal_Displacement_Epi_point2 - Average_values_per_frame_Longitudinal_Displacement_Epi_point1)/Time_duration1

Average_values_per_frame_Longitudinal_Displacement_Epi_a2 = (Average_values_per_frame_Longitudinal_Displacement_Epi_point3 - Average_values_per_frame_Longitudinal_Displacement_Epi_point2)/Time_duration2

Average_values_per_frame_Longitudinal_Displacement_Epi_a3 = (Average_values_per_frame_Longitudinal_Displacement_Epi_point4 - Average_values_per_frame_Longitudinal_Displacement_Epi_point3)/Time_duration3

Average_values_per_frame_Longitudinal_Displacement_Epi_a4 = (Average_values_per_frame_Longitudinal_Displacement_Epi_point5 - Average_values_per_frame_Longitudinal_Displacement_Epi_point4)/Time_duration4

Average_values_per_frame_Longitudinal_Displacement_Epi_a_min = min(c(Average_values_per_frame_Longitudinal_Displacement_Epi_a1,Average_values_per_frame_Longitudinal_Displacement_Epi_a2,Average_values_per_frame_Longitudinal_Displacement_Epi_a3,Average_values_per_frame_Longitudinal_Displacement_Epi_a4))

Average_values_per_frame_Longitudinal_Displacement_Epi_a_max = max(c(Average_values_per_frame_Longitudinal_Displacement_Epi_a1,Average_values_per_frame_Longitudinal_Displacement_Epi_a2,Average_values_per_frame_Longitudinal_Displacement_Epi_a3,Average_values_per_frame_Longitudinal_Displacement_Epi_a4))

#Strain

Strain_data <- read.xlsx(i,sheetIndex = 4, header = F)

colnames(Strain_data) <- Strain_data[3,]

name1 = paste("fr0",time3,sep = "")

col5 = grep(name1,colnames(Strain_data))

col4 = col5-1

col3 = col5-2

col2 = col5-3

col1 = col5-4

Timepoint1 = as.numeric(Strain_data[4,col1])

Timepoint2 = as.numeric(Strain_data[4,col2])

Timepoint3 = as.numeric(Strain_data[4,col3])

Timepoint4 = as.numeric(Strain_data[4,col4])

Timepoint5 = as.numeric(Strain_data[4,col5])

Time_duration1 = Timepoint2 - Timepoint1

Time_duration2 = Timepoint3 - Timepoint2

Time_duration3 = Timepoint4 - Timepoint3

Time_duration4 = Timepoint5 - Timepoint4

##Radial_Strain_Endo

Radial_Strain_Endo_seg001_point1 = as.numeric(Strain_data[8,col1])

Radial_Strain_Endo_seg001_point2 = as.numeric(Strain_data[8,col2])

Radial_Strain_Endo_seg001_point3 = as.numeric(Strain_data[8,col3])

Radial_Strain_Endo_seg001_point4 = as.numeric(Strain_data[8,col4])

Radial_Strain_Endo_seg001_point5 = as.numeric(Strain_data[8,col5])

Radial_Strain_Endo_seg001_a1 = (Radial_Strain_Endo_seg001_point2 - Radial_Strain_Endo_seg001_point1)/Time_duration1

Radial_Strain_Endo_seg001_a2 = (Radial_Strain_Endo_seg001_point3 - Radial_Strain_Endo_seg001_point2)/Time_duration2

Radial_Strain_Endo_seg001_a3 = (Radial_Strain_Endo_seg001_point4 - Radial_Strain_Endo_seg001_point3)/Time_duration3

Radial_Strain_Endo_seg001_a4 = (Radial_Strain_Endo_seg001_point5 - Radial_Strain_Endo_seg001_point4)/Time_duration4

Radial_Strain_Endo_seg001_a_min = min(c(Radial_Strain_Endo_seg001_a1,Radial_Strain_Endo_seg001_a2,Radial_Strain_Endo_seg001_a3,Radial_Strain_Endo_seg001_a4))

Radial_Strain_Endo_seg001_a_max = max(c(Radial_Strain_Endo_seg001_a1,Radial_Strain_Endo_seg001_a2,Radial_Strain_Endo_seg001_a3,Radial_Strain_Endo_seg001_a4))

Radial_Strain_Endo_seg002_point1 = as.numeric(Strain_data[9,col1])

Radial_Strain_Endo_seg002_point2 = as.numeric(Strain_data[9,col2])

Radial_Strain_Endo_seg002_point3 = as.numeric(Strain_data[9,col3])

Radial_Strain_Endo_seg002_point4 = as.numeric(Strain_data[9,col4])

Radial_Strain_Endo_seg002_point5 = as.numeric(Strain_data[9,col5])

Radial_Strain_Endo_seg002_a1 = (Radial_Strain_Endo_seg002_point2 - Radial_Strain_Endo_seg002_point1)/Time_duration1

Radial_Strain_Endo_seg002_a2 = (Radial_Strain_Endo_seg002_point3 - Radial_Strain_Endo_seg002_point2)/Time_duration2

Radial_Strain_Endo_seg002_a3 = (Radial_Strain_Endo_seg002_point4 - Radial_Strain_Endo_seg002_point3)/Time_duration3

Radial_Strain_Endo_seg002_a4 = (Radial_Strain_Endo_seg002_point5 - Radial_Strain_Endo_seg002_point4)/Time_duration4

Radial_Strain_Endo_seg002_a_min = min(c(Radial_Strain_Endo_seg002_a1,Radial_Strain_Endo_seg002_a2,Radial_Strain_Endo_seg002_a3,Radial_Strain_Endo_seg002_a4))

Radial_Strain_Endo_seg002_a_max = max(c(Radial_Strain_Endo_seg002_a1,Radial_Strain_Endo_seg002_a2,Radial_Strain_Endo_seg002_a3,Radial_Strain_Endo_seg002_a4))

Radial_Strain_Endo_seg003_point1 = as.numeric(Strain_data[10,col1])

Radial_Strain_Endo_seg003_point2 = as.numeric(Strain_data[10,col2])

Radial_Strain_Endo_seg003_point3 = as.numeric(Strain_data[10,col3])

Radial_Strain_Endo_seg003_point4 = as.numeric(Strain_data[10,col4])

Radial_Strain_Endo_seg003_point5 = as.numeric(Strain_data[10,col5])

Radial_Strain_Endo_seg003_a1 = (Radial_Strain_Endo_seg003_point2 - Radial_Strain_Endo_seg003_point1)/Time_duration1

Radial_Strain_Endo_seg003_a2 = (Radial_Strain_Endo_seg003_point3 - Radial_Strain_Endo_seg003_point2)/Time_duration2

Radial_Strain_Endo_seg003_a3 = (Radial_Strain_Endo_seg003_point4 - Radial_Strain_Endo_seg003_point3)/Time_duration3

Radial_Strain_Endo_seg003_a4 = (Radial_Strain_Endo_seg003_point5 - Radial_Strain_Endo_seg003_point4)/Time_duration4

Radial_Strain_Endo_seg003_a_min = min(c(Radial_Strain_Endo_seg003_a1,Radial_Strain_Endo_seg003_a2,Radial_Strain_Endo_seg003_a3,Radial_Strain_Endo_seg003_a4))

Radial_Strain_Endo_seg003_a_max = max(c(Radial_Strain_Endo_seg003_a1,Radial_Strain_Endo_seg003_a2,Radial_Strain_Endo_seg003_a3,Radial_Strain_Endo_seg003_a4))

Radial_Strain_Endo_seg004_point1 = as.numeric(Strain_data[11,col1])

Radial_Strain_Endo_seg004_point2 = as.numeric(Strain_data[11,col2])

Radial_Strain_Endo_seg004_point3 = as.numeric(Strain_data[11,col3])

Radial_Strain_Endo_seg004_point4 = as.numeric(Strain_data[11,col4])

Radial_Strain_Endo_seg004_point5 = as.numeric(Strain_data[11,col5])

Radial_Strain_Endo_seg004_a1 = (Radial_Strain_Endo_seg004_point2 - Radial_Strain_Endo_seg004_point1)/Time_duration1

Radial_Strain_Endo_seg004_a2 = (Radial_Strain_Endo_seg004_point3 - Radial_Strain_Endo_seg004_point2)/Time_duration2

Radial_Strain_Endo_seg004_a3 = (Radial_Strain_Endo_seg004_point4 - Radial_Strain_Endo_seg004_point3)/Time_duration3

Radial_Strain_Endo_seg004_a4 = (Radial_Strain_Endo_seg004_point5 - Radial_Strain_Endo_seg004_point4)/Time_duration4

Radial_Strain_Endo_seg004_a_min = min(c(Radial_Strain_Endo_seg004_a1,Radial_Strain_Endo_seg004_a2,Radial_Strain_Endo_seg004_a3,Radial_Strain_Endo_seg004_a4))

Radial_Strain_Endo_seg004_a_max = max(c(Radial_Strain_Endo_seg004_a1,Radial_Strain_Endo_seg004_a2,Radial_Strain_Endo_seg004_a3,Radial_Strain_Endo_seg004_a4))

Radial_Strain_Endo_seg005_point1 = as.numeric(Strain_data[12,col1])

Radial_Strain_Endo_seg005_point2 = as.numeric(Strain_data[12,col2])

Radial_Strain_Endo_seg005_point3 = as.numeric(Strain_data[12,col3])

Radial_Strain_Endo_seg005_point4 = as.numeric(Strain_data[12,col4])

Radial_Strain_Endo_seg005_point5 = as.numeric(Strain_data[12,col5])

Radial_Strain_Endo_seg005_a1 = (Radial_Strain_Endo_seg005_point2 - Radial_Strain_Endo_seg005_point1)/Time_duration1

Radial_Strain_Endo_seg005_a2 = (Radial_Strain_Endo_seg005_point3 - Radial_Strain_Endo_seg005_point2)/Time_duration2

Radial_Strain_Endo_seg005_a3 = (Radial_Strain_Endo_seg005_point4 - Radial_Strain_Endo_seg005_point3)/Time_duration3

Radial_Strain_Endo_seg005_a4 = (Radial_Strain_Endo_seg005_point5 - Radial_Strain_Endo_seg005_point4)/Time_duration4

Radial_Strain_Endo_seg005_a_min = min(c(Radial_Strain_Endo_seg005_a1,Radial_Strain_Endo_seg005_a2,Radial_Strain_Endo_seg005_a3,Radial_Strain_Endo_seg005_a4))

Radial_Strain_Endo_seg005_a_max = max(c(Radial_Strain_Endo_seg005_a1,Radial_Strain_Endo_seg005_a2,Radial_Strain_Endo_seg005_a3,Radial_Strain_Endo_seg005_a4))

Radial_Strain_Endo_seg006_point1 = as.numeric(Strain_data[13,col1])

Radial_Strain_Endo_seg006_point2 = as.numeric(Strain_data[13,col2])

Radial_Strain_Endo_seg006_point3 = as.numeric(Strain_data[13,col3])

Radial_Strain_Endo_seg006_point4 = as.numeric(Strain_data[13,col4])

Radial_Strain_Endo_seg006_point5 = as.numeric(Strain_data[13,col5])

Radial_Strain_Endo_seg006_a1 = (Radial_Strain_Endo_seg006_point2 - Radial_Strain_Endo_seg006_point1)/Time_duration1

Radial_Strain_Endo_seg006_a2 = (Radial_Strain_Endo_seg006_point3 - Radial_Strain_Endo_seg006_point2)/Time_duration2

Radial_Strain_Endo_seg006_a3 = (Radial_Strain_Endo_seg006_point4 - Radial_Strain_Endo_seg006_point3)/Time_duration3

Radial_Strain_Endo_seg006_a4 = (Radial_Strain_Endo_seg006_point5 - Radial_Strain_Endo_seg006_point4)/Time_duration4

Radial_Strain_Endo_seg006_a_min = min(c(Radial_Strain_Endo_seg006_a1,Radial_Strain_Endo_seg006_a2,Radial_Strain_Endo_seg006_a3,Radial_Strain_Endo_seg006_a4))

Radial_Strain_Endo_seg006_a_max = max(c(Radial_Strain_Endo_seg006_a1,Radial_Strain_Endo_seg006_a2,Radial_Strain_Endo_seg006_a3,Radial_Strain_Endo_seg006_a4))

##Average_values_per_frame_Radial_Strain_Endo

Average_values_per_frame_Radial_Strain_Endo_point1 = as.numeric(Strain_data[17,col1])

Average_values_per_frame_Radial_Strain_Endo_point2 = as.numeric(Strain_data[17,col2])

Average_values_per_frame_Radial_Strain_Endo_point3 = as.numeric(Strain_data[17,col3])

Average_values_per_frame_Radial_Strain_Endo_point4 = as.numeric(Strain_data[17,col4])

Average_values_per_frame_Radial_Strain_Endo_point5 = as.numeric(Strain_data[17,col5])

Average_values_per_frame_Radial_Strain_Endo_a1 = (Average_values_per_frame_Radial_Strain_Endo_point2 - Average_values_per_frame_Radial_Strain_Endo_point1)/Time_duration1

Average_values_per_frame_Radial_Strain_Endo_a2 = (Average_values_per_frame_Radial_Strain_Endo_point3 - Average_values_per_frame_Radial_Strain_Endo_point2)/Time_duration2

Average_values_per_frame_Radial_Strain_Endo_a3 = (Average_values_per_frame_Radial_Strain_Endo_point4 - Average_values_per_frame_Radial_Strain_Endo_point3)/Time_duration3

Average_values_per_frame_Radial_Strain_Endo_a4 = (Average_values_per_frame_Radial_Strain_Endo_point5 - Average_values_per_frame_Radial_Strain_Endo_point4)/Time_duration4

Average_values_per_frame_Radial_Strain_Endo_a_min = min(c(Average_values_per_frame_Radial_Strain_Endo_a1,Average_values_per_frame_Radial_Strain_Endo_a2,Average_values_per_frame_Radial_Strain_Endo_a3,Average_values_per_frame_Radial_Strain_Endo_a4))

Average_values_per_frame_Radial_Strain_Endo_a_max = max(c(Average_values_per_frame_Radial_Strain_Endo_a1,Average_values_per_frame_Radial_Strain_Endo_a2,Average_values_per_frame_Radial_Strain_Endo_a3,Average_values_per_frame_Radial_Strain_Endo_a4))

##Longitudinal_Strain_Endo

Longitudinal_Strain_Endo_seg001_point1 = as.numeric(Strain_data[21,col1])

Longitudinal_Strain_Endo_seg001_point2 = as.numeric(Strain_data[21,col2])

Longitudinal_Strain_Endo_seg001_point3 = as.numeric(Strain_data[21,col3])

Longitudinal_Strain_Endo_seg001_point4 = as.numeric(Strain_data[21,col4])

Longitudinal_Strain_Endo_seg001_point5 = as.numeric(Strain_data[21,col5])

Longitudinal_Strain_Endo_seg001_a1 = (Longitudinal_Strain_Endo_seg001_point2 - Longitudinal_Strain_Endo_seg001_point1)/Time_duration1

Longitudinal_Strain_Endo_seg001_a2 = (Longitudinal_Strain_Endo_seg001_point3 - Longitudinal_Strain_Endo_seg001_point2)/Time_duration2

Longitudinal_Strain_Endo_seg001_a3 = (Longitudinal_Strain_Endo_seg001_point4 - Longitudinal_Strain_Endo_seg001_point3)/Time_duration3

Longitudinal_Strain_Endo_seg001_a4 = (Longitudinal_Strain_Endo_seg001_point5 - Longitudinal_Strain_Endo_seg001_point4)/Time_duration4

Longitudinal_Strain_Endo_seg001_a_min = min(c(Longitudinal_Strain_Endo_seg001_a1,Longitudinal_Strain_Endo_seg001_a2,Longitudinal_Strain_Endo_seg001_a3,Longitudinal_Strain_Endo_seg001_a4))

Longitudinal_Strain_Endo_seg001_a_max = max(c(Longitudinal_Strain_Endo_seg001_a1,Longitudinal_Strain_Endo_seg001_a2,Longitudinal_Strain_Endo_seg001_a3,Longitudinal_Strain_Endo_seg001_a4))

Longitudinal_Strain_Endo_seg002_point1 = as.numeric(Strain_data[22,col1])

Longitudinal_Strain_Endo_seg002_point2 = as.numeric(Strain_data[22,col2])

Longitudinal_Strain_Endo_seg002_point3 = as.numeric(Strain_data[22,col3])

Longitudinal_Strain_Endo_seg002_point4 = as.numeric(Strain_data[22,col4])

Longitudinal_Strain_Endo_seg002_point5 = as.numeric(Strain_data[22,col5])

Longitudinal_Strain_Endo_seg002_a1 = (Longitudinal_Strain_Endo_seg002_point2 - Longitudinal_Strain_Endo_seg002_point1)/Time_duration1

Longitudinal_Strain_Endo_seg002_a2 = (Longitudinal_Strain_Endo_seg002_point3 - Longitudinal_Strain_Endo_seg002_point2)/Time_duration2

Longitudinal_Strain_Endo_seg002_a3 = (Longitudinal_Strain_Endo_seg002_point4 - Longitudinal_Strain_Endo_seg002_point3)/Time_duration3

Longitudinal_Strain_Endo_seg002_a4 = (Longitudinal_Strain_Endo_seg002_point5 - Longitudinal_Strain_Endo_seg002_point4)/Time_duration4

Longitudinal_Strain_Endo_seg002_a_min = min(c(Longitudinal_Strain_Endo_seg002_a1,Longitudinal_Strain_Endo_seg002_a2,Longitudinal_Strain_Endo_seg002_a3,Longitudinal_Strain_Endo_seg002_a4))

Longitudinal_Strain_Endo_seg002_a_max = max(c(Longitudinal_Strain_Endo_seg002_a1,Longitudinal_Strain_Endo_seg002_a2,Longitudinal_Strain_Endo_seg002_a3,Longitudinal_Strain_Endo_seg002_a4))

Longitudinal_Strain_Endo_seg003_point1 = as.numeric(Strain_data[23,col1])

Longitudinal_Strain_Endo_seg003_point2 = as.numeric(Strain_data[23,col2])

Longitudinal_Strain_Endo_seg003_point3 = as.numeric(Strain_data[23,col3])

Longitudinal_Strain_Endo_seg003_point4 = as.numeric(Strain_data[23,col4])

Longitudinal_Strain_Endo_seg003_point5 = as.numeric(Strain_data[23,col5])

Longitudinal_Strain_Endo_seg003_a1 = (Longitudinal_Strain_Endo_seg003_point2 - Longitudinal_Strain_Endo_seg003_point1)/Time_duration1

Longitudinal_Strain_Endo_seg003_a2 = (Longitudinal_Strain_Endo_seg003_point3 - Longitudinal_Strain_Endo_seg003_point2)/Time_duration2

Longitudinal_Strain_Endo_seg003_a3 = (Longitudinal_Strain_Endo_seg003_point4 - Longitudinal_Strain_Endo_seg003_point3)/Time_duration3

Longitudinal_Strain_Endo_seg003_a4 = (Longitudinal_Strain_Endo_seg003_point5 - Longitudinal_Strain_Endo_seg003_point4)/Time_duration4

Longitudinal_Strain_Endo_seg003_a_min = min(c(Longitudinal_Strain_Endo_seg003_a1,Longitudinal_Strain_Endo_seg003_a2,Longitudinal_Strain_Endo_seg003_a3,Longitudinal_Strain_Endo_seg003_a4))

Longitudinal_Strain_Endo_seg003_a_max = max(c(Longitudinal_Strain_Endo_seg003_a1,Longitudinal_Strain_Endo_seg003_a2,Longitudinal_Strain_Endo_seg003_a3,Longitudinal_Strain_Endo_seg003_a4))

Longitudinal_Strain_Endo_seg004_point1 = as.numeric(Strain_data[24,col1])

Longitudinal_Strain_Endo_seg004_point2 = as.numeric(Strain_data[24,col2])

Longitudinal_Strain_Endo_seg004_point3 = as.numeric(Strain_data[24,col3])

Longitudinal_Strain_Endo_seg004_point4 = as.numeric(Strain_data[24,col4])

Longitudinal_Strain_Endo_seg004_point5 = as.numeric(Strain_data[24,col5])

Longitudinal_Strain_Endo_seg004_a1 = (Longitudinal_Strain_Endo_seg004_point2 - Longitudinal_Strain_Endo_seg004_point1)/Time_duration1

Longitudinal_Strain_Endo_seg004_a2 = (Longitudinal_Strain_Endo_seg004_point3 - Longitudinal_Strain_Endo_seg004_point2)/Time_duration2

Longitudinal_Strain_Endo_seg004_a3 = (Longitudinal_Strain_Endo_seg004_point4 - Longitudinal_Strain_Endo_seg004_point3)/Time_duration3

Longitudinal_Strain_Endo_seg004_a4 = (Longitudinal_Strain_Endo_seg004_point5 - Longitudinal_Strain_Endo_seg004_point4)/Time_duration4

Longitudinal_Strain_Endo_seg004_a_min = min(c(Longitudinal_Strain_Endo_seg004_a1,Longitudinal_Strain_Endo_seg004_a2,Longitudinal_Strain_Endo_seg004_a3,Longitudinal_Strain_Endo_seg004_a4))

Longitudinal_Strain_Endo_seg004_a_max = max(c(Longitudinal_Strain_Endo_seg004_a1,Longitudinal_Strain_Endo_seg004_a2,Longitudinal_Strain_Endo_seg004_a3,Longitudinal_Strain_Endo_seg004_a4))

Longitudinal_Strain_Endo_seg005_point1 = as.numeric(Strain_data[25,col1])

Longitudinal_Strain_Endo_seg005_point2 = as.numeric(Strain_data[25,col2])

Longitudinal_Strain_Endo_seg005_point3 = as.numeric(Strain_data[25,col3])

Longitudinal_Strain_Endo_seg005_point4 = as.numeric(Strain_data[25,col4])

Longitudinal_Strain_Endo_seg005_point5 = as.numeric(Strain_data[25,col5])

Longitudinal_Strain_Endo_seg005_a1 = (Longitudinal_Strain_Endo_seg005_point2 - Longitudinal_Strain_Endo_seg005_point1)/Time_duration1

Longitudinal_Strain_Endo_seg005_a2 = (Longitudinal_Strain_Endo_seg005_point3 - Longitudinal_Strain_Endo_seg005_point2)/Time_duration2

Longitudinal_Strain_Endo_seg005_a3 = (Longitudinal_Strain_Endo_seg005_point4 - Longitudinal_Strain_Endo_seg005_point3)/Time_duration3

Longitudinal_Strain_Endo_seg005_a4 = (Longitudinal_Strain_Endo_seg005_point5 - Longitudinal_Strain_Endo_seg005_point4)/Time_duration4

Longitudinal_Strain_Endo_seg005_a_min = min(c(Longitudinal_Strain_Endo_seg005_a1,Longitudinal_Strain_Endo_seg005_a2,Longitudinal_Strain_Endo_seg005_a3,Longitudinal_Strain_Endo_seg005_a4))

Longitudinal_Strain_Endo_seg005_a_max = max(c(Longitudinal_Strain_Endo_seg005_a1,Longitudinal_Strain_Endo_seg005_a2,Longitudinal_Strain_Endo_seg005_a3,Longitudinal_Strain_Endo_seg005_a4))

Longitudinal_Strain_Endo_seg006_point1 = as.numeric(Strain_data[26,col1])

Longitudinal_Strain_Endo_seg006_point2 = as.numeric(Strain_data[26,col2])

Longitudinal_Strain_Endo_seg006_point3 = as.numeric(Strain_data[26,col3])

Longitudinal_Strain_Endo_seg006_point4 = as.numeric(Strain_data[26,col4])

Longitudinal_Strain_Endo_seg006_point5 = as.numeric(Strain_data[26,col5])

Longitudinal_Strain_Endo_seg006_a1 = (Longitudinal_Strain_Endo_seg006_point2 - Longitudinal_Strain_Endo_seg006_point1)/Time_duration1

Longitudinal_Strain_Endo_seg006_a2 = (Longitudinal_Strain_Endo_seg006_point3 - Longitudinal_Strain_Endo_seg006_point2)/Time_duration2

Longitudinal_Strain_Endo_seg006_a3 = (Longitudinal_Strain_Endo_seg006_point4 - Longitudinal_Strain_Endo_seg006_point3)/Time_duration3

Longitudinal_Strain_Endo_seg006_a4 = (Longitudinal_Strain_Endo_seg006_point5 - Longitudinal_Strain_Endo_seg006_point4)/Time_duration4

Longitudinal_Strain_Endo_seg006_a_min = min(c(Longitudinal_Strain_Endo_seg006_a1,Longitudinal_Strain_Endo_seg006_a2,Longitudinal_Strain_Endo_seg006_a3,Longitudinal_Strain_Endo_seg006_a4))

Longitudinal_Strain_Endo_seg006_a_max = max(c(Longitudinal_Strain_Endo_seg006_a1,Longitudinal_Strain_Endo_seg006_a2,Longitudinal_Strain_Endo_seg006_a3,Longitudinal_Strain_Endo_seg006_a4))

##Average_values_per_frame_Longitudinal_Strain_Endo

Average_values_per_frame_Longitudinal_Strain_Endo_point1 = as.numeric(Strain_data[30,col1])

Average_values_per_frame_Longitudinal_Strain_Endo_point2 = as.numeric(Strain_data[30,col2])

Average_values_per_frame_Longitudinal_Strain_Endo_point3 = as.numeric(Strain_data[30,col3])

Average_values_per_frame_Longitudinal_Strain_Endo_point4 = as.numeric(Strain_data[30,col4])

Average_values_per_frame_Longitudinal_Strain_Endo_point5 = as.numeric(Strain_data[30,col5])

Average_values_per_frame_Longitudinal_Strain_Endo_a1 = (Average_values_per_frame_Longitudinal_Strain_Endo_point2 - Average_values_per_frame_Longitudinal_Strain_Endo_point1)/Time_duration1

Average_values_per_frame_Longitudinal_Strain_Endo_a2 = (Average_values_per_frame_Longitudinal_Strain_Endo_point3 - Average_values_per_frame_Longitudinal_Strain_Endo_point2)/Time_duration2

Average_values_per_frame_Longitudinal_Strain_Endo_a3 = (Average_values_per_frame_Longitudinal_Strain_Endo_point4 - Average_values_per_frame_Longitudinal_Strain_Endo_point3)/Time_duration3

Average_values_per_frame_Longitudinal_Strain_Endo_a4 = (Average_values_per_frame_Longitudinal_Strain_Endo_point5 - Average_values_per_frame_Longitudinal_Strain_Endo_point4)/Time_duration4

Average_values_per_frame_Longitudinal_Strain_Endo_a_min = min(c(Average_values_per_frame_Longitudinal_Strain_Endo_a1,Average_values_per_frame_Longitudinal_Strain_Endo_a2,Average_values_per_frame_Longitudinal_Strain_Endo_a3,Average_values_per_frame_Longitudinal_Strain_Endo_a4))

Average_values_per_frame_Longitudinal_Strain_Endo_a_max = max(c(Average_values_per_frame_Longitudinal_Strain_Endo_a1,Average_values_per_frame_Longitudinal_Strain_Endo_a2,Average_values_per_frame_Longitudinal_Strain_Endo_a3,Average_values_per_frame_Longitudinal_Strain_Endo_a4))

##Radial_Strain_Epi

Radial_Strain_Epi_seg001_point1 = as.numeric(Strain_data[34,col1])

Radial_Strain_Epi_seg001_point2 = as.numeric(Strain_data[34,col2])

Radial_Strain_Epi_seg001_point3 = as.numeric(Strain_data[34,col3])

Radial_Strain_Epi_seg001_point4 = as.numeric(Strain_data[34,col4])

Radial_Strain_Epi_seg001_point5 = as.numeric(Strain_data[34,col5])

Radial_Strain_Epi_seg001_a1 = (Radial_Strain_Epi_seg001_point2 - Radial_Strain_Epi_seg001_point1)/Time_duration1

Radial_Strain_Epi_seg001_a2 = (Radial_Strain_Epi_seg001_point3 - Radial_Strain_Epi_seg001_point2)/Time_duration2

Radial_Strain_Epi_seg001_a3 = (Radial_Strain_Epi_seg001_point4 - Radial_Strain_Epi_seg001_point3)/Time_duration3

Radial_Strain_Epi_seg001_a4 = (Radial_Strain_Epi_seg001_point5 - Radial_Strain_Epi_seg001_point4)/Time_duration4

Radial_Strain_Epi_seg001_a_min = min(c(Radial_Strain_Epi_seg001_a1,Radial_Strain_Epi_seg001_a2,Radial_Strain_Epi_seg001_a3,Radial_Strain_Epi_seg001_a4))

Radial_Strain_Epi_seg001_a_max = max(c(Radial_Strain_Epi_seg001_a1,Radial_Strain_Epi_seg001_a2,Radial_Strain_Epi_seg001_a3,Radial_Strain_Epi_seg001_a4))

Radial_Strain_Epi_seg002_point1 = as.numeric(Strain_data[35,col1])

Radial_Strain_Epi_seg002_point2 = as.numeric(Strain_data[35,col2])

Radial_Strain_Epi_seg002_point3 = as.numeric(Strain_data[35,col3])

Radial_Strain_Epi_seg002_point4 = as.numeric(Strain_data[35,col4])

Radial_Strain_Epi_seg002_point5 = as.numeric(Strain_data[35,col5])

Radial_Strain_Epi_seg002_a1 = (Radial_Strain_Epi_seg002_point2 - Radial_Strain_Epi_seg002_point1)/Time_duration1

Radial_Strain_Epi_seg002_a2 = (Radial_Strain_Epi_seg002_point3 - Radial_Strain_Epi_seg002_point2)/Time_duration2

Radial_Strain_Epi_seg002_a3 = (Radial_Strain_Epi_seg002_point4 - Radial_Strain_Epi_seg002_point3)/Time_duration3

Radial_Strain_Epi_seg002_a4 = (Radial_Strain_Epi_seg002_point5 - Radial_Strain_Epi_seg002_point4)/Time_duration4

Radial_Strain_Epi_seg002_a_min = min(c(Radial_Strain_Epi_seg002_a1,Radial_Strain_Epi_seg002_a2,Radial_Strain_Epi_seg002_a3,Radial_Strain_Epi_seg002_a4))

Radial_Strain_Epi_seg002_a_max = max(c(Radial_Strain_Epi_seg002_a1,Radial_Strain_Epi_seg002_a2,Radial_Strain_Epi_seg002_a3,Radial_Strain_Epi_seg002_a4))

Radial_Strain_Epi_seg003_point1 = as.numeric(Strain_data[36,col1])

Radial_Strain_Epi_seg003_point2 = as.numeric(Strain_data[36,col2])

Radial_Strain_Epi_seg003_point3 = as.numeric(Strain_data[36,col3])

Radial_Strain_Epi_seg003_point4 = as.numeric(Strain_data[36,col4])

Radial_Strain_Epi_seg003_point5 = as.numeric(Strain_data[36,col5])

Radial_Strain_Epi_seg003_a1 = (Radial_Strain_Epi_seg003_point2 - Radial_Strain_Epi_seg003_point1)/Time_duration1

Radial_Strain_Epi_seg003_a2 = (Radial_Strain_Epi_seg003_point3 - Radial_Strain_Epi_seg003_point2)/Time_duration2

Radial_Strain_Epi_seg003_a3 = (Radial_Strain_Epi_seg003_point4 - Radial_Strain_Epi_seg003_point3)/Time_duration3

Radial_Strain_Epi_seg003_a4 = (Radial_Strain_Epi_seg003_point5 - Radial_Strain_Epi_seg003_point4)/Time_duration4

Radial_Strain_Epi_seg003_a_min = min(c(Radial_Strain_Epi_seg003_a1,Radial_Strain_Epi_seg003_a2,Radial_Strain_Epi_seg003_a3,Radial_Strain_Epi_seg003_a4))

Radial_Strain_Epi_seg003_a_max = max(c(Radial_Strain_Epi_seg003_a1,Radial_Strain_Epi_seg003_a2,Radial_Strain_Epi_seg003_a3,Radial_Strain_Epi_seg003_a4))

Radial_Strain_Epi_seg004_point1 = as.numeric(Strain_data[37,col1])

Radial_Strain_Epi_seg004_point2 = as.numeric(Strain_data[37,col2])

Radial_Strain_Epi_seg004_point3 = as.numeric(Strain_data[37,col3])

Radial_Strain_Epi_seg004_point4 = as.numeric(Strain_data[37,col4])

Radial_Strain_Epi_seg004_point5 = as.numeric(Strain_data[37,col5])

Radial_Strain_Epi_seg004_a1 = (Radial_Strain_Epi_seg004_point2 - Radial_Strain_Epi_seg004_point1)/Time_duration1

Radial_Strain_Epi_seg004_a2 = (Radial_Strain_Epi_seg004_point3 - Radial_Strain_Epi_seg004_point2)/Time_duration2

Radial_Strain_Epi_seg004_a3 = (Radial_Strain_Epi_seg004_point4 - Radial_Strain_Epi_seg004_point3)/Time_duration3

Radial_Strain_Epi_seg004_a4 = (Radial_Strain_Epi_seg004_point5 - Radial_Strain_Epi_seg004_point4)/Time_duration4

Radial_Strain_Epi_seg004_a_min = min(c(Radial_Strain_Epi_seg004_a1,Radial_Strain_Epi_seg004_a2,Radial_Strain_Epi_seg004_a3,Radial_Strain_Epi_seg004_a4))

Radial_Strain_Epi_seg004_a_max = max(c(Radial_Strain_Epi_seg004_a1,Radial_Strain_Epi_seg004_a2,Radial_Strain_Epi_seg004_a3,Radial_Strain_Epi_seg004_a4))

Radial_Strain_Epi_seg005_point1 = as.numeric(Strain_data[38,col1])

Radial_Strain_Epi_seg005_point2 = as.numeric(Strain_data[38,col2])

Radial_Strain_Epi_seg005_point3 = as.numeric(Strain_data[38,col3])

Radial_Strain_Epi_seg005_point4 = as.numeric(Strain_data[38,col4])

Radial_Strain_Epi_seg005_point5 = as.numeric(Strain_data[38,col5])

Radial_Strain_Epi_seg005_a1 = (Radial_Strain_Epi_seg005_point2 - Radial_Strain_Epi_seg005_point1)/Time_duration1

Radial_Strain_Epi_seg005_a2 = (Radial_Strain_Epi_seg005_point3 - Radial_Strain_Epi_seg005_point2)/Time_duration2

Radial_Strain_Epi_seg005_a3 = (Radial_Strain_Epi_seg005_point4 - Radial_Strain_Epi_seg005_point3)/Time_duration3

Radial_Strain_Epi_seg005_a4 = (Radial_Strain_Epi_seg005_point5 - Radial_Strain_Epi_seg005_point4)/Time_duration4

Radial_Strain_Epi_seg005_a_min = min(c(Radial_Strain_Epi_seg005_a1,Radial_Strain_Epi_seg005_a2,Radial_Strain_Epi_seg005_a3,Radial_Strain_Epi_seg005_a4))

Radial_Strain_Epi_seg005_a_max = max(c(Radial_Strain_Epi_seg005_a1,Radial_Strain_Epi_seg005_a2,Radial_Strain_Epi_seg005_a3,Radial_Strain_Epi_seg005_a4))

Radial_Strain_Epi_seg006_point1 = as.numeric(Strain_data[39,col1])

Radial_Strain_Epi_seg006_point2 = as.numeric(Strain_data[39,col2])

Radial_Strain_Epi_seg006_point3 = as.numeric(Strain_data[39,col3])

Radial_Strain_Epi_seg006_point4 = as.numeric(Strain_data[39,col4])

Radial_Strain_Epi_seg006_point5 = as.numeric(Strain_data[39,col5])

Radial_Strain_Epi_seg006_a1 = (Radial_Strain_Epi_seg006_point2 - Radial_Strain_Epi_seg006_point1)/Time_duration1

Radial_Strain_Epi_seg006_a2 = (Radial_Strain_Epi_seg006_point3 - Radial_Strain_Epi_seg006_point2)/Time_duration2

Radial_Strain_Epi_seg006_a3 = (Radial_Strain_Epi_seg006_point4 - Radial_Strain_Epi_seg006_point3)/Time_duration3

Radial_Strain_Epi_seg006_a4 = (Radial_Strain_Epi_seg006_point5 - Radial_Strain_Epi_seg006_point4)/Time_duration4

Radial_Strain_Epi_seg006_a_min = min(c(Radial_Strain_Epi_seg006_a1,Radial_Strain_Epi_seg006_a2,Radial_Strain_Epi_seg006_a3,Radial_Strain_Epi_seg006_a4))

Radial_Strain_Epi_seg006_a_max = max(c(Radial_Strain_Epi_seg006_a1,Radial_Strain_Epi_seg006_a2,Radial_Strain_Epi_seg006_a3,Radial_Strain_Epi_seg006_a4))

##Average_values_per_frame_Radial_Strain_Epi

Average_values_per_frame_Radial_Strain_Epi_point1 = as.numeric(Strain_data[43,col1])

Average_values_per_frame_Radial_Strain_Epi_point2 = as.numeric(Strain_data[43,col2])

Average_values_per_frame_Radial_Strain_Epi_point3 = as.numeric(Strain_data[43,col3])

Average_values_per_frame_Radial_Strain_Epi_point4 = as.numeric(Strain_data[43,col4])

Average_values_per_frame_Radial_Strain_Epi_point5 = as.numeric(Strain_data[43,col5])

Average_values_per_frame_Radial_Strain_Epi_a1 = (Average_values_per_frame_Radial_Strain_Epi_point2 - Average_values_per_frame_Radial_Strain_Epi_point1)/Time_duration1

Average_values_per_frame_Radial_Strain_Epi_a2 = (Average_values_per_frame_Radial_Strain_Epi_point3 - Average_values_per_frame_Radial_Strain_Epi_point2)/Time_duration2

Average_values_per_frame_Radial_Strain_Epi_a3 = (Average_values_per_frame_Radial_Strain_Epi_point4 - Average_values_per_frame_Radial_Strain_Epi_point3)/Time_duration3

Average_values_per_frame_Radial_Strain_Epi_a4 = (Average_values_per_frame_Radial_Strain_Epi_point5 - Average_values_per_frame_Radial_Strain_Epi_point4)/Time_duration4

Average_values_per_frame_Radial_Strain_Epi_a_min = min(c(Average_values_per_frame_Radial_Strain_Epi_a1,Average_values_per_frame_Radial_Strain_Epi_a2,Average_values_per_frame_Radial_Strain_Epi_a3,Average_values_per_frame_Radial_Strain_Epi_a4))

Average_values_per_frame_Radial_Strain_Epi_a_max = max(c(Average_values_per_frame_Radial_Strain_Epi_a1,Average_values_per_frame_Radial_Strain_Epi_a2,Average_values_per_frame_Radial_Strain_Epi_a3,Average_values_per_frame_Radial_Strain_Epi_a4))

##Longitudinal_Strain_Epi

Longitudinal_Strain_Epi_seg001_point1 = as.numeric(Strain_data[47,col1])

Longitudinal_Strain_Epi_seg001_point2 = as.numeric(Strain_data[47,col2])

Longitudinal_Strain_Epi_seg001_point3 = as.numeric(Strain_data[47,col3])

Longitudinal_Strain_Epi_seg001_point4 = as.numeric(Strain_data[47,col4])

Longitudinal_Strain_Epi_seg001_point5 = as.numeric(Strain_data[47,col5])

Longitudinal_Strain_Epi_seg001_a1 = (Longitudinal_Strain_Epi_seg001_point2 - Longitudinal_Strain_Epi_seg001_point1)/Time_duration1

Longitudinal_Strain_Epi_seg001_a2 = (Longitudinal_Strain_Epi_seg001_point3 - Longitudinal_Strain_Epi_seg001_point2)/Time_duration2

Longitudinal_Strain_Epi_seg001_a3 = (Longitudinal_Strain_Epi_seg001_point4 - Longitudinal_Strain_Epi_seg001_point3)/Time_duration3

Longitudinal_Strain_Epi_seg001_a4 = (Longitudinal_Strain_Epi_seg001_point5 - Longitudinal_Strain_Epi_seg001_point4)/Time_duration4

Longitudinal_Strain_Epi_seg001_a_min = min(c(Longitudinal_Strain_Epi_seg001_a1,Longitudinal_Strain_Epi_seg001_a2,Longitudinal_Strain_Epi_seg001_a3,Longitudinal_Strain_Epi_seg001_a4))

Longitudinal_Strain_Epi_seg001_a_max = max(c(Longitudinal_Strain_Epi_seg001_a1,Longitudinal_Strain_Epi_seg001_a2,Longitudinal_Strain_Epi_seg001_a3,Longitudinal_Strain_Epi_seg001_a4))

Longitudinal_Strain_Epi_seg002_point1 = as.numeric(Strain_data[48,col1])

Longitudinal_Strain_Epi_seg002_point2 = as.numeric(Strain_data[48,col2])

Longitudinal_Strain_Epi_seg002_point3 = as.numeric(Strain_data[48,col3])

Longitudinal_Strain_Epi_seg002_point4 = as.numeric(Strain_data[48,col4])

Longitudinal_Strain_Epi_seg002_point5 = as.numeric(Strain_data[48,col5])

Longitudinal_Strain_Epi_seg002_a1 = (Longitudinal_Strain_Epi_seg002_point2 - Longitudinal_Strain_Epi_seg002_point1)/Time_duration1

Longitudinal_Strain_Epi_seg002_a2 = (Longitudinal_Strain_Epi_seg002_point3 - Longitudinal_Strain_Epi_seg002_point2)/Time_duration2

Longitudinal_Strain_Epi_seg002_a3 = (Longitudinal_Strain_Epi_seg002_point4 - Longitudinal_Strain_Epi_seg002_point3)/Time_duration3

Longitudinal_Strain_Epi_seg002_a4 = (Longitudinal_Strain_Epi_seg002_point5 - Longitudinal_Strain_Epi_seg002_point4)/Time_duration4

Longitudinal_Strain_Epi_seg002_a_min = min(c(Longitudinal_Strain_Epi_seg002_a1,Longitudinal_Strain_Epi_seg002_a2,Longitudinal_Strain_Epi_seg002_a3,Longitudinal_Strain_Epi_seg002_a4))

Longitudinal_Strain_Epi_seg002_a_max = max(c(Longitudinal_Strain_Epi_seg002_a1,Longitudinal_Strain_Epi_seg002_a2,Longitudinal_Strain_Epi_seg002_a3,Longitudinal_Strain_Epi_seg002_a4))

Longitudinal_Strain_Epi_seg003_point1 = as.numeric(Strain_data[49,col1])

Longitudinal_Strain_Epi_seg003_point2 = as.numeric(Strain_data[49,col2])

Longitudinal_Strain_Epi_seg003_point3 = as.numeric(Strain_data[49,col3])

Longitudinal_Strain_Epi_seg003_point4 = as.numeric(Strain_data[49,col4])

Longitudinal_Strain_Epi_seg003_point5 = as.numeric(Strain_data[49,col5])

Longitudinal_Strain_Epi_seg003_a1 = (Longitudinal_Strain_Epi_seg003_point2 - Longitudinal_Strain_Epi_seg003_point1)/Time_duration1

Longitudinal_Strain_Epi_seg003_a2 = (Longitudinal_Strain_Epi_seg003_point3 - Longitudinal_Strain_Epi_seg003_point2)/Time_duration2

Longitudinal_Strain_Epi_seg003_a3 = (Longitudinal_Strain_Epi_seg003_point4 - Longitudinal_Strain_Epi_seg003_point3)/Time_duration3

Longitudinal_Strain_Epi_seg003_a4 = (Longitudinal_Strain_Epi_seg003_point5 - Longitudinal_Strain_Epi_seg003_point4)/Time_duration4

Longitudinal_Strain_Epi_seg003_a_min = min(c(Longitudinal_Strain_Epi_seg003_a1,Longitudinal_Strain_Epi_seg003_a2,Longitudinal_Strain_Epi_seg003_a3,Longitudinal_Strain_Epi_seg003_a4))

Longitudinal_Strain_Epi_seg003_a_max = max(c(Longitudinal_Strain_Epi_seg003_a1,Longitudinal_Strain_Epi_seg003_a2,Longitudinal_Strain_Epi_seg003_a3,Longitudinal_Strain_Epi_seg003_a4))

Longitudinal_Strain_Epi_seg004_point1 = as.numeric(Strain_data[50,col1])

Longitudinal_Strain_Epi_seg004_point2 = as.numeric(Strain_data[50,col2])

Longitudinal_Strain_Epi_seg004_point3 = as.numeric(Strain_data[50,col3])

Longitudinal_Strain_Epi_seg004_point4 = as.numeric(Strain_data[50,col4])

Longitudinal_Strain_Epi_seg004_point5 = as.numeric(Strain_data[50,col5])

Longitudinal_Strain_Epi_seg004_a1 = (Longitudinal_Strain_Epi_seg004_point2 - Longitudinal_Strain_Epi_seg004_point1)/Time_duration1

Longitudinal_Strain_Epi_seg004_a2 = (Longitudinal_Strain_Epi_seg004_point3 - Longitudinal_Strain_Epi_seg004_point2)/Time_duration2

Longitudinal_Strain_Epi_seg004_a3 = (Longitudinal_Strain_Epi_seg004_point4 - Longitudinal_Strain_Epi_seg004_point3)/Time_duration3

Longitudinal_Strain_Epi_seg004_a4 = (Longitudinal_Strain_Epi_seg004_point5 - Longitudinal_Strain_Epi_seg004_point4)/Time_duration4

Longitudinal_Strain_Epi_seg004_a_min = min(c(Longitudinal_Strain_Epi_seg004_a1,Longitudinal_Strain_Epi_seg004_a2,Longitudinal_Strain_Epi_seg004_a3,Longitudinal_Strain_Epi_seg004_a4))

Longitudinal_Strain_Epi_seg004_a_max = max(c(Longitudinal_Strain_Epi_seg004_a1,Longitudinal_Strain_Epi_seg004_a2,Longitudinal_Strain_Epi_seg004_a3,Longitudinal_Strain_Epi_seg004_a4))

Longitudinal_Strain_Epi_seg005_point1 = as.numeric(Strain_data[51,col1])

Longitudinal_Strain_Epi_seg005_point2 = as.numeric(Strain_data[51,col2])

Longitudinal_Strain_Epi_seg005_point3 = as.numeric(Strain_data[51,col3])

Longitudinal_Strain_Epi_seg005_point4 = as.numeric(Strain_data[51,col4])

Longitudinal_Strain_Epi_seg005_point5 = as.numeric(Strain_data[51,col5])

Longitudinal_Strain_Epi_seg005_a1 = (Longitudinal_Strain_Epi_seg005_point2 - Longitudinal_Strain_Epi_seg005_point1)/Time_duration1

Longitudinal_Strain_Epi_seg005_a2 = (Longitudinal_Strain_Epi_seg005_point3 - Longitudinal_Strain_Epi_seg005_point2)/Time_duration2

Longitudinal_Strain_Epi_seg005_a3 = (Longitudinal_Strain_Epi_seg005_point4 - Longitudinal_Strain_Epi_seg005_point3)/Time_duration3

Longitudinal_Strain_Epi_seg005_a4 = (Longitudinal_Strain_Epi_seg005_point5 - Longitudinal_Strain_Epi_seg005_point4)/Time_duration4

Longitudinal_Strain_Epi_seg005_a_min = min(c(Longitudinal_Strain_Epi_seg005_a1,Longitudinal_Strain_Epi_seg005_a2,Longitudinal_Strain_Epi_seg005_a3,Longitudinal_Strain_Epi_seg005_a4))

Longitudinal_Strain_Epi_seg005_a_max = max(c(Longitudinal_Strain_Epi_seg005_a1,Longitudinal_Strain_Epi_seg005_a2,Longitudinal_Strain_Epi_seg005_a3,Longitudinal_Strain_Epi_seg005_a4))

Longitudinal_Strain_Epi_seg006_point1 = as.numeric(Strain_data[52,col1])

Longitudinal_Strain_Epi_seg006_point2 = as.numeric(Strain_data[52,col2])

Longitudinal_Strain_Epi_seg006_point3 = as.numeric(Strain_data[52,col3])

Longitudinal_Strain_Epi_seg006_point4 = as.numeric(Strain_data[52,col4])

Longitudinal_Strain_Epi_seg006_point5 = as.numeric(Strain_data[52,col5])

Longitudinal_Strain_Epi_seg006_a1 = (Longitudinal_Strain_Epi_seg006_point2 - Longitudinal_Strain_Epi_seg006_point1)/Time_duration1

Longitudinal_Strain_Epi_seg006_a2 = (Longitudinal_Strain_Epi_seg006_point3 - Longitudinal_Strain_Epi_seg006_point2)/Time_duration2

Longitudinal_Strain_Epi_seg006_a3 = (Longitudinal_Strain_Epi_seg006_point4 - Longitudinal_Strain_Epi_seg006_point3)/Time_duration3

Longitudinal_Strain_Epi_seg006_a4 = (Longitudinal_Strain_Epi_seg006_point5 - Longitudinal_Strain_Epi_seg006_point4)/Time_duration4

Longitudinal_Strain_Epi_seg006_a_min = min(c(Longitudinal_Strain_Epi_seg006_a1,Longitudinal_Strain_Epi_seg006_a2,Longitudinal_Strain_Epi_seg006_a3,Longitudinal_Strain_Epi_seg006_a4))

Longitudinal_Strain_Epi_seg006_a_max = max(c(Longitudinal_Strain_Epi_seg006_a1,Longitudinal_Strain_Epi_seg006_a2,Longitudinal_Strain_Epi_seg006_a3,Longitudinal_Strain_Epi_seg006_a4))

##Average_values_per_frame_Longitudinal_Strain_Epi

Average_values_per_frame_Longitudinal_Strain_Epi_point1 = as.numeric(Strain_data[56,col1])

Average_values_per_frame_Longitudinal_Strain_Epi_point2 = as.numeric(Strain_data[56,col2])

Average_values_per_frame_Longitudinal_Strain_Epi_point3 = as.numeric(Strain_data[56,col3])

Average_values_per_frame_Longitudinal_Strain_Epi_point4 = as.numeric(Strain_data[56,col4])

Average_values_per_frame_Longitudinal_Strain_Epi_point5 = as.numeric(Strain_data[56,col5])

Average_values_per_frame_Longitudinal_Strain_Epi_a1 = (Average_values_per_frame_Longitudinal_Strain_Epi_point2 - Average_values_per_frame_Longitudinal_Strain_Epi_point1)/Time_duration1

Average_values_per_frame_Longitudinal_Strain_Epi_a2 = (Average_values_per_frame_Longitudinal_Strain_Epi_point3 - Average_values_per_frame_Longitudinal_Strain_Epi_point2)/Time_duration2

Average_values_per_frame_Longitudinal_Strain_Epi_a3 = (Average_values_per_frame_Longitudinal_Strain_Epi_point4 - Average_values_per_frame_Longitudinal_Strain_Epi_point3)/Time_duration3

Average_values_per_frame_Longitudinal_Strain_Epi_a4 = (Average_values_per_frame_Longitudinal_Strain_Epi_point5 - Average_values_per_frame_Longitudinal_Strain_Epi_point4)/Time_duration4

Average_values_per_frame_Longitudinal_Strain_Epi_a_min = min(c(Average_values_per_frame_Longitudinal_Strain_Epi_a1,Average_values_per_frame_Longitudinal_Strain_Epi_a2,Average_values_per_frame_Longitudinal_Strain_Epi_a3,Average_values_per_frame_Longitudinal_Strain_Epi_a4))

Average_values_per_frame_Longitudinal_Strain_Epi_a_max = max(c(Average_values_per_frame_Longitudinal_Strain_Epi_a1,Average_values_per_frame_Longitudinal_Strain_Epi_a2,Average_values_per_frame_Longitudinal_Strain_Epi_a3,Average_values_per_frame_Longitudinal_Strain_Epi_a4))

#Strain_rate

Strain_Rate_data <- read.xlsx(i,sheetIndex = 5, header = F)

colnames(Strain_Rate_data) <- Strain_Rate_data[3,]

name1 = paste("fr0",time3,sep = "")

col5 = grep(name1,colnames(Strain_Rate_data))

col4 = col5-1

col3 = col5-2

col2 = col5-3

col1 = col5-4

Timepoint1 = as.numeric(Strain_Rate_data[4,col1])

Timepoint2 = as.numeric(Strain_Rate_data[4,col2])

Timepoint3 = as.numeric(Strain_Rate_data[4,col3])

Timepoint4 = as.numeric(Strain_Rate_data[4,col4])

Timepoint5 = as.numeric(Strain_Rate_data[4,col5])

Time_duration1 = Timepoint2 - Timepoint1

Time_duration2 = Timepoint3 - Timepoint2

Time_duration3 = Timepoint4 - Timepoint3

Time_duration4 = Timepoint5 - Timepoint4

##Radial_Strain_Rate_Endo

Radial_Strain_Rate_Endo_seg001_point1 = as.numeric(Strain_Rate_data[8,col1])

Radial_Strain_Rate_Endo_seg001_point2 = as.numeric(Strain_Rate_data[8,col2])

Radial_Strain_Rate_Endo_seg001_point3 = as.numeric(Strain_Rate_data[8,col3])

Radial_Strain_Rate_Endo_seg001_point4 = as.numeric(Strain_Rate_data[8,col4])

Radial_Strain_Rate_Endo_seg001_point5 = as.numeric(Strain_Rate_data[8,col5])

Radial_Strain_Rate_Endo_seg001_a1 = (Radial_Strain_Rate_Endo_seg001_point2 - Radial_Strain_Rate_Endo_seg001_point1)/Time_duration1

Radial_Strain_Rate_Endo_seg001_a2 = (Radial_Strain_Rate_Endo_seg001_point3 - Radial_Strain_Rate_Endo_seg001_point2)/Time_duration2

Radial_Strain_Rate_Endo_seg001_a3 = (Radial_Strain_Rate_Endo_seg001_point4 - Radial_Strain_Rate_Endo_seg001_point3)/Time_duration3

Radial_Strain_Rate_Endo_seg001_a4 = (Radial_Strain_Rate_Endo_seg001_point5 - Radial_Strain_Rate_Endo_seg001_point4)/Time_duration4

Radial_Strain_Rate_Endo_seg001_a_min = min(c(Radial_Strain_Rate_Endo_seg001_a1,Radial_Strain_Rate_Endo_seg001_a2,Radial_Strain_Rate_Endo_seg001_a3,Radial_Strain_Rate_Endo_seg001_a4))

Radial_Strain_Rate_Endo_seg001_a_max = max(c(Radial_Strain_Rate_Endo_seg001_a1,Radial_Strain_Rate_Endo_seg001_a2,Radial_Strain_Rate_Endo_seg001_a3,Radial_Strain_Rate_Endo_seg001_a4))

Radial_Strain_Rate_Endo_seg002_point1 = as.numeric(Strain_Rate_data[9,col1])

Radial_Strain_Rate_Endo_seg002_point2 = as.numeric(Strain_Rate_data[9,col2])

Radial_Strain_Rate_Endo_seg002_point3 = as.numeric(Strain_Rate_data[9,col3])

Radial_Strain_Rate_Endo_seg002_point4 = as.numeric(Strain_Rate_data[9,col4])

Radial_Strain_Rate_Endo_seg002_point5 = as.numeric(Strain_Rate_data[9,col5])

Radial_Strain_Rate_Endo_seg002_a1 = (Radial_Strain_Rate_Endo_seg002_point2 - Radial_Strain_Rate_Endo_seg002_point1)/Time_duration1

Radial_Strain_Rate_Endo_seg002_a2 = (Radial_Strain_Rate_Endo_seg002_point3 - Radial_Strain_Rate_Endo_seg002_point2)/Time_duration2

Radial_Strain_Rate_Endo_seg002_a3 = (Radial_Strain_Rate_Endo_seg002_point4 - Radial_Strain_Rate_Endo_seg002_point3)/Time_duration3

Radial_Strain_Rate_Endo_seg002_a4 = (Radial_Strain_Rate_Endo_seg002_point5 - Radial_Strain_Rate_Endo_seg002_point4)/Time_duration4

Radial_Strain_Rate_Endo_seg002_a_min = min(c(Radial_Strain_Rate_Endo_seg002_a1,Radial_Strain_Rate_Endo_seg002_a2,Radial_Strain_Rate_Endo_seg002_a3,Radial_Strain_Rate_Endo_seg002_a4))

Radial_Strain_Rate_Endo_seg002_a_max = max(c(Radial_Strain_Rate_Endo_seg002_a1,Radial_Strain_Rate_Endo_seg002_a2,Radial_Strain_Rate_Endo_seg002_a3,Radial_Strain_Rate_Endo_seg002_a4))

Radial_Strain_Rate_Endo_seg003_point1 = as.numeric(Strain_Rate_data[10,col1])

Radial_Strain_Rate_Endo_seg003_point2 = as.numeric(Strain_Rate_data[10,col2])

Radial_Strain_Rate_Endo_seg003_point3 = as.numeric(Strain_Rate_data[10,col3])

Radial_Strain_Rate_Endo_seg003_point4 = as.numeric(Strain_Rate_data[10,col4])

Radial_Strain_Rate_Endo_seg003_point5 = as.numeric(Strain_Rate_data[10,col5])

Radial_Strain_Rate_Endo_seg003_a1 = (Radial_Strain_Rate_Endo_seg003_point2 - Radial_Strain_Rate_Endo_seg003_point1)/Time_duration1

Radial_Strain_Rate_Endo_seg003_a2 = (Radial_Strain_Rate_Endo_seg003_point3 - Radial_Strain_Rate_Endo_seg003_point2)/Time_duration2

Radial_Strain_Rate_Endo_seg003_a3 = (Radial_Strain_Rate_Endo_seg003_point4 - Radial_Strain_Rate_Endo_seg003_point3)/Time_duration3

Radial_Strain_Rate_Endo_seg003_a4 = (Radial_Strain_Rate_Endo_seg003_point5 - Radial_Strain_Rate_Endo_seg003_point4)/Time_duration4

Radial_Strain_Rate_Endo_seg003_a_min = min(c(Radial_Strain_Rate_Endo_seg003_a1,Radial_Strain_Rate_Endo_seg003_a2,Radial_Strain_Rate_Endo_seg003_a3,Radial_Strain_Rate_Endo_seg003_a4))

Radial_Strain_Rate_Endo_seg003_a_max = max(c(Radial_Strain_Rate_Endo_seg003_a1,Radial_Strain_Rate_Endo_seg003_a2,Radial_Strain_Rate_Endo_seg003_a3,Radial_Strain_Rate_Endo_seg003_a4))

Radial_Strain_Rate_Endo_seg004_point1 = as.numeric(Strain_Rate_data[11,col1])

Radial_Strain_Rate_Endo_seg004_point2 = as.numeric(Strain_Rate_data[11,col2])

Radial_Strain_Rate_Endo_seg004_point3 = as.numeric(Strain_Rate_data[11,col3])

Radial_Strain_Rate_Endo_seg004_point4 = as.numeric(Strain_Rate_data[11,col4])

Radial_Strain_Rate_Endo_seg004_point5 = as.numeric(Strain_Rate_data[11,col5])

Radial_Strain_Rate_Endo_seg004_a1 = (Radial_Strain_Rate_Endo_seg004_point2 - Radial_Strain_Rate_Endo_seg004_point1)/Time_duration1

Radial_Strain_Rate_Endo_seg004_a2 = (Radial_Strain_Rate_Endo_seg004_point3 - Radial_Strain_Rate_Endo_seg004_point2)/Time_duration2

Radial_Strain_Rate_Endo_seg004_a3 = (Radial_Strain_Rate_Endo_seg004_point4 - Radial_Strain_Rate_Endo_seg004_point3)/Time_duration3

Radial_Strain_Rate_Endo_seg004_a4 = (Radial_Strain_Rate_Endo_seg004_point5 - Radial_Strain_Rate_Endo_seg004_point4)/Time_duration4

Radial_Strain_Rate_Endo_seg004_a_min = min(c(Radial_Strain_Rate_Endo_seg004_a1,Radial_Strain_Rate_Endo_seg004_a2,Radial_Strain_Rate_Endo_seg004_a3,Radial_Strain_Rate_Endo_seg004_a4))

Radial_Strain_Rate_Endo_seg004_a_max = max(c(Radial_Strain_Rate_Endo_seg004_a1,Radial_Strain_Rate_Endo_seg004_a2,Radial_Strain_Rate_Endo_seg004_a3,Radial_Strain_Rate_Endo_seg004_a4))

Radial_Strain_Rate_Endo_seg005_point1 = as.numeric(Strain_Rate_data[12,col1])

Radial_Strain_Rate_Endo_seg005_point2 = as.numeric(Strain_Rate_data[12,col2])

Radial_Strain_Rate_Endo_seg005_point3 = as.numeric(Strain_Rate_data[12,col3])

Radial_Strain_Rate_Endo_seg005_point4 = as.numeric(Strain_Rate_data[12,col4])

Radial_Strain_Rate_Endo_seg005_point5 = as.numeric(Strain_Rate_data[12,col5])

Radial_Strain_Rate_Endo_seg005_a1 = (Radial_Strain_Rate_Endo_seg005_point2 - Radial_Strain_Rate_Endo_seg005_point1)/Time_duration1

Radial_Strain_Rate_Endo_seg005_a2 = (Radial_Strain_Rate_Endo_seg005_point3 - Radial_Strain_Rate_Endo_seg005_point2)/Time_duration2

Radial_Strain_Rate_Endo_seg005_a3 = (Radial_Strain_Rate_Endo_seg005_point4 - Radial_Strain_Rate_Endo_seg005_point3)/Time_duration3

Radial_Strain_Rate_Endo_seg005_a4 = (Radial_Strain_Rate_Endo_seg005_point5 - Radial_Strain_Rate_Endo_seg005_point4)/Time_duration4

Radial_Strain_Rate_Endo_seg005_a_min = min(c(Radial_Strain_Rate_Endo_seg005_a1,Radial_Strain_Rate_Endo_seg005_a2,Radial_Strain_Rate_Endo_seg005_a3,Radial_Strain_Rate_Endo_seg005_a4))

Radial_Strain_Rate_Endo_seg005_a_max = max(c(Radial_Strain_Rate_Endo_seg005_a1,Radial_Strain_Rate_Endo_seg005_a2,Radial_Strain_Rate_Endo_seg005_a3,Radial_Strain_Rate_Endo_seg005_a4))

Radial_Strain_Rate_Endo_seg006_point1 = as.numeric(Strain_Rate_data[13,col1])

Radial_Strain_Rate_Endo_seg006_point2 = as.numeric(Strain_Rate_data[13,col2])

Radial_Strain_Rate_Endo_seg006_point3 = as.numeric(Strain_Rate_data[13,col3])

Radial_Strain_Rate_Endo_seg006_point4 = as.numeric(Strain_Rate_data[13,col4])

Radial_Strain_Rate_Endo_seg006_point5 = as.numeric(Strain_Rate_data[13,col5])

Radial_Strain_Rate_Endo_seg006_a1 = (Radial_Strain_Rate_Endo_seg006_point2 - Radial_Strain_Rate_Endo_seg006_point1)/Time_duration1

Radial_Strain_Rate_Endo_seg006_a2 = (Radial_Strain_Rate_Endo_seg006_point3 - Radial_Strain_Rate_Endo_seg006_point2)/Time_duration2

Radial_Strain_Rate_Endo_seg006_a3 = (Radial_Strain_Rate_Endo_seg006_point4 - Radial_Strain_Rate_Endo_seg006_point3)/Time_duration3

Radial_Strain_Rate_Endo_seg006_a4 = (Radial_Strain_Rate_Endo_seg006_point5 - Radial_Strain_Rate_Endo_seg006_point4)/Time_duration4

Radial_Strain_Rate_Endo_seg006_a_min = min(c(Radial_Strain_Rate_Endo_seg006_a1,Radial_Strain_Rate_Endo_seg006_a2,Radial_Strain_Rate_Endo_seg006_a3,Radial_Strain_Rate_Endo_seg006_a4))

Radial_Strain_Rate_Endo_seg006_a_max = max(c(Radial_Strain_Rate_Endo_seg006_a1,Radial_Strain_Rate_Endo_seg006_a2,Radial_Strain_Rate_Endo_seg006_a3,Radial_Strain_Rate_Endo_seg006_a4))

##Average_values_per_frame_Radial_Strain_Rate_Endo

Average_values_per_frame_Radial_Strain_Rate_Endo_point1 = as.numeric(Strain_Rate_data[17,col1])

Average_values_per_frame_Radial_Strain_Rate_Endo_point2 = as.numeric(Strain_Rate_data[17,col2])

Average_values_per_frame_Radial_Strain_Rate_Endo_point3 = as.numeric(Strain_Rate_data[17,col3])

Average_values_per_frame_Radial_Strain_Rate_Endo_point4 = as.numeric(Strain_Rate_data[17,col4])

Average_values_per_frame_Radial_Strain_Rate_Endo_point5 = as.numeric(Strain_Rate_data[17,col5])

Average_values_per_frame_Radial_Strain_Rate_Endo_a1 = (Average_values_per_frame_Radial_Strain_Rate_Endo_point2 - Average_values_per_frame_Radial_Strain_Rate_Endo_point1)/Time_duration1

Average_values_per_frame_Radial_Strain_Rate_Endo_a2 = (Average_values_per_frame_Radial_Strain_Rate_Endo_point3 - Average_values_per_frame_Radial_Strain_Rate_Endo_point2)/Time_duration2

Average_values_per_frame_Radial_Strain_Rate_Endo_a3 = (Average_values_per_frame_Radial_Strain_Rate_Endo_point4 - Average_values_per_frame_Radial_Strain_Rate_Endo_point3)/Time_duration3

Average_values_per_frame_Radial_Strain_Rate_Endo_a4 = (Average_values_per_frame_Radial_Strain_Rate_Endo_point5 - Average_values_per_frame_Radial_Strain_Rate_Endo_point4)/Time_duration4

Average_values_per_frame_Radial_Strain_Rate_Endo_a_min = min(c(Average_values_per_frame_Radial_Strain_Rate_Endo_a1,Average_values_per_frame_Radial_Strain_Rate_Endo_a2,Average_values_per_frame_Radial_Strain_Rate_Endo_a3,Average_values_per_frame_Radial_Strain_Rate_Endo_a4))

Average_values_per_frame_Radial_Strain_Rate_Endo_a_max = max(c(Average_values_per_frame_Radial_Strain_Rate_Endo_a1,Average_values_per_frame_Radial_Strain_Rate_Endo_a2,Average_values_per_frame_Radial_Strain_Rate_Endo_a3,Average_values_per_frame_Radial_Strain_Rate_Endo_a4))

##Longitudinal_Strain_Rate_Endo

Longitudinal_Strain_Rate_Endo_seg001_point1 = as.numeric(Strain_Rate_data[21,col1])

Longitudinal_Strain_Rate_Endo_seg001_point2 = as.numeric(Strain_Rate_data[21,col2])

Longitudinal_Strain_Rate_Endo_seg001_point3 = as.numeric(Strain_Rate_data[21,col3])

Longitudinal_Strain_Rate_Endo_seg001_point4 = as.numeric(Strain_Rate_data[21,col4])

Longitudinal_Strain_Rate_Endo_seg001_point5 = as.numeric(Strain_Rate_data[21,col5])

Longitudinal_Strain_Rate_Endo_seg001_a1 = (Longitudinal_Strain_Rate_Endo_seg001_point2 - Longitudinal_Strain_Rate_Endo_seg001_point1)/Time_duration1

Longitudinal_Strain_Rate_Endo_seg001_a2 = (Longitudinal_Strain_Rate_Endo_seg001_point3 - Longitudinal_Strain_Rate_Endo_seg001_point2)/Time_duration2

Longitudinal_Strain_Rate_Endo_seg001_a3 = (Longitudinal_Strain_Rate_Endo_seg001_point4 - Longitudinal_Strain_Rate_Endo_seg001_point3)/Time_duration3

Longitudinal_Strain_Rate_Endo_seg001_a4 = (Longitudinal_Strain_Rate_Endo_seg001_point5 - Longitudinal_Strain_Rate_Endo_seg001_point4)/Time_duration4

Longitudinal_Strain_Rate_Endo_seg001_a_min = min(c(Longitudinal_Strain_Rate_Endo_seg001_a1,Longitudinal_Strain_Rate_Endo_seg001_a2,Longitudinal_Strain_Rate_Endo_seg001_a3,Longitudinal_Strain_Rate_Endo_seg001_a4))

Longitudinal_Strain_Rate_Endo_seg001_a_max = max(c(Longitudinal_Strain_Rate_Endo_seg001_a1,Longitudinal_Strain_Rate_Endo_seg001_a2,Longitudinal_Strain_Rate_Endo_seg001_a3,Longitudinal_Strain_Rate_Endo_seg001_a4))

Longitudinal_Strain_Rate_Endo_seg002_point1 = as.numeric(Strain_Rate_data[22,col1])

Longitudinal_Strain_Rate_Endo_seg002_point2 = as.numeric(Strain_Rate_data[22,col2])

Longitudinal_Strain_Rate_Endo_seg002_point3 = as.numeric(Strain_Rate_data[22,col3])

Longitudinal_Strain_Rate_Endo_seg002_point4 = as.numeric(Strain_Rate_data[22,col4])

Longitudinal_Strain_Rate_Endo_seg002_point5 = as.numeric(Strain_Rate_data[22,col5])

Longitudinal_Strain_Rate_Endo_seg002_a1 = (Longitudinal_Strain_Rate_Endo_seg002_point2 - Longitudinal_Strain_Rate_Endo_seg002_point1)/Time_duration1

Longitudinal_Strain_Rate_Endo_seg002_a2 = (Longitudinal_Strain_Rate_Endo_seg002_point3 - Longitudinal_Strain_Rate_Endo_seg002_point2)/Time_duration2

Longitudinal_Strain_Rate_Endo_seg002_a3 = (Longitudinal_Strain_Rate_Endo_seg002_point4 - Longitudinal_Strain_Rate_Endo_seg002_point3)/Time_duration3

Longitudinal_Strain_Rate_Endo_seg002_a4 = (Longitudinal_Strain_Rate_Endo_seg002_point5 - Longitudinal_Strain_Rate_Endo_seg002_point4)/Time_duration4

Longitudinal_Strain_Rate_Endo_seg002_a_min = min(c(Longitudinal_Strain_Rate_Endo_seg002_a1,Longitudinal_Strain_Rate_Endo_seg002_a2,Longitudinal_Strain_Rate_Endo_seg002_a3,Longitudinal_Strain_Rate_Endo_seg002_a4))

Longitudinal_Strain_Rate_Endo_seg002_a_max = max(c(Longitudinal_Strain_Rate_Endo_seg002_a1,Longitudinal_Strain_Rate_Endo_seg002_a2,Longitudinal_Strain_Rate_Endo_seg002_a3,Longitudinal_Strain_Rate_Endo_seg002_a4))

Longitudinal_Strain_Rate_Endo_seg003_point1 = as.numeric(Strain_Rate_data[23,col1])

Longitudinal_Strain_Rate_Endo_seg003_point2 = as.numeric(Strain_Rate_data[23,col2])

Longitudinal_Strain_Rate_Endo_seg003_point3 = as.numeric(Strain_Rate_data[23,col3])

Longitudinal_Strain_Rate_Endo_seg003_point4 = as.numeric(Strain_Rate_data[23,col4])

Longitudinal_Strain_Rate_Endo_seg003_point5 = as.numeric(Strain_Rate_data[23,col5])

Longitudinal_Strain_Rate_Endo_seg003_a1 = (Longitudinal_Strain_Rate_Endo_seg003_point2 - Longitudinal_Strain_Rate_Endo_seg003_point1)/Time_duration1

Longitudinal_Strain_Rate_Endo_seg003_a2 = (Longitudinal_Strain_Rate_Endo_seg003_point3 - Longitudinal_Strain_Rate_Endo_seg003_point2)/Time_duration2

Longitudinal_Strain_Rate_Endo_seg003_a3 = (Longitudinal_Strain_Rate_Endo_seg003_point4 - Longitudinal_Strain_Rate_Endo_seg003_point3)/Time_duration3

Longitudinal_Strain_Rate_Endo_seg003_a4 = (Longitudinal_Strain_Rate_Endo_seg003_point5 - Longitudinal_Strain_Rate_Endo_seg003_point4)/Time_duration4

Longitudinal_Strain_Rate_Endo_seg003_a_min = min(c(Longitudinal_Strain_Rate_Endo_seg003_a1,Longitudinal_Strain_Rate_Endo_seg003_a2,Longitudinal_Strain_Rate_Endo_seg003_a3,Longitudinal_Strain_Rate_Endo_seg003_a4))

Longitudinal_Strain_Rate_Endo_seg003_a_max = max(c(Longitudinal_Strain_Rate_Endo_seg003_a1,Longitudinal_Strain_Rate_Endo_seg003_a2,Longitudinal_Strain_Rate_Endo_seg003_a3,Longitudinal_Strain_Rate_Endo_seg003_a4))

Longitudinal_Strain_Rate_Endo_seg004_point1 = as.numeric(Strain_Rate_data[24,col1])

Longitudinal_Strain_Rate_Endo_seg004_point2 = as.numeric(Strain_Rate_data[24,col2])

Longitudinal_Strain_Rate_Endo_seg004_point3 = as.numeric(Strain_Rate_data[24,col3])

Longitudinal_Strain_Rate_Endo_seg004_point4 = as.numeric(Strain_Rate_data[24,col4])

Longitudinal_Strain_Rate_Endo_seg004_point5 = as.numeric(Strain_Rate_data[24,col5])

Longitudinal_Strain_Rate_Endo_seg004_a1 = (Longitudinal_Strain_Rate_Endo_seg004_point2 - Longitudinal_Strain_Rate_Endo_seg004_point1)/Time_duration1

Longitudinal_Strain_Rate_Endo_seg004_a2 = (Longitudinal_Strain_Rate_Endo_seg004_point3 - Longitudinal_Strain_Rate_Endo_seg004_point2)/Time_duration2

Longitudinal_Strain_Rate_Endo_seg004_a3 = (Longitudinal_Strain_Rate_Endo_seg004_point4 - Longitudinal_Strain_Rate_Endo_seg004_point3)/Time_duration3

Longitudinal_Strain_Rate_Endo_seg004_a4 = (Longitudinal_Strain_Rate_Endo_seg004_point5 - Longitudinal_Strain_Rate_Endo_seg004_point4)/Time_duration4

Longitudinal_Strain_Rate_Endo_seg004_a_min = min(c(Longitudinal_Strain_Rate_Endo_seg004_a1,Longitudinal_Strain_Rate_Endo_seg004_a2,Longitudinal_Strain_Rate_Endo_seg004_a3,Longitudinal_Strain_Rate_Endo_seg004_a4))

Longitudinal_Strain_Rate_Endo_seg004_a_max = max(c(Longitudinal_Strain_Rate_Endo_seg004_a1,Longitudinal_Strain_Rate_Endo_seg004_a2,Longitudinal_Strain_Rate_Endo_seg004_a3,Longitudinal_Strain_Rate_Endo_seg004_a4))

Longitudinal_Strain_Rate_Endo_seg005_point1 = as.numeric(Strain_Rate_data[25,col1])

Longitudinal_Strain_Rate_Endo_seg005_point2 = as.numeric(Strain_Rate_data[25,col2])

Longitudinal_Strain_Rate_Endo_seg005_point3 = as.numeric(Strain_Rate_data[25,col3])

Longitudinal_Strain_Rate_Endo_seg005_point4 = as.numeric(Strain_Rate_data[25,col4])

Longitudinal_Strain_Rate_Endo_seg005_point5 = as.numeric(Strain_Rate_data[25,col5])

Longitudinal_Strain_Rate_Endo_seg005_a1 = (Longitudinal_Strain_Rate_Endo_seg005_point2 - Longitudinal_Strain_Rate_Endo_seg005_point1)/Time_duration1

Longitudinal_Strain_Rate_Endo_seg005_a2 = (Longitudinal_Strain_Rate_Endo_seg005_point3 - Longitudinal_Strain_Rate_Endo_seg005_point2)/Time_duration2

Longitudinal_Strain_Rate_Endo_seg005_a3 = (Longitudinal_Strain_Rate_Endo_seg005_point4 - Longitudinal_Strain_Rate_Endo_seg005_point3)/Time_duration3

Longitudinal_Strain_Rate_Endo_seg005_a4 = (Longitudinal_Strain_Rate_Endo_seg005_point5 - Longitudinal_Strain_Rate_Endo_seg005_point4)/Time_duration4

Longitudinal_Strain_Rate_Endo_seg005_a_min = min(c(Longitudinal_Strain_Rate_Endo_seg005_a1,Longitudinal_Strain_Rate_Endo_seg005_a2,Longitudinal_Strain_Rate_Endo_seg005_a3,Longitudinal_Strain_Rate_Endo_seg005_a4))

Longitudinal_Strain_Rate_Endo_seg005_a_max = max(c(Longitudinal_Strain_Rate_Endo_seg005_a1,Longitudinal_Strain_Rate_Endo_seg005_a2,Longitudinal_Strain_Rate_Endo_seg005_a3,Longitudinal_Strain_Rate_Endo_seg005_a4))

Longitudinal_Strain_Rate_Endo_seg006_point1 = as.numeric(Strain_Rate_data[26,col1])

Longitudinal_Strain_Rate_Endo_seg006_point2 = as.numeric(Strain_Rate_data[26,col2])

Longitudinal_Strain_Rate_Endo_seg006_point3 = as.numeric(Strain_Rate_data[26,col3])

Longitudinal_Strain_Rate_Endo_seg006_point4 = as.numeric(Strain_Rate_data[26,col4])

Longitudinal_Strain_Rate_Endo_seg006_point5 = as.numeric(Strain_Rate_data[26,col5])

Longitudinal_Strain_Rate_Endo_seg006_a1 = (Longitudinal_Strain_Rate_Endo_seg006_point2 - Longitudinal_Strain_Rate_Endo_seg006_point1)/Time_duration1

Longitudinal_Strain_Rate_Endo_seg006_a2 = (Longitudinal_Strain_Rate_Endo_seg006_point3 - Longitudinal_Strain_Rate_Endo_seg006_point2)/Time_duration2

Longitudinal_Strain_Rate_Endo_seg006_a3 = (Longitudinal_Strain_Rate_Endo_seg006_point4 - Longitudinal_Strain_Rate_Endo_seg006_point3)/Time_duration3

Longitudinal_Strain_Rate_Endo_seg006_a4 = (Longitudinal_Strain_Rate_Endo_seg006_point5 - Longitudinal_Strain_Rate_Endo_seg006_point4)/Time_duration4

Longitudinal_Strain_Rate_Endo_seg006_a_min = min(c(Longitudinal_Strain_Rate_Endo_seg006_a1,Longitudinal_Strain_Rate_Endo_seg006_a2,Longitudinal_Strain_Rate_Endo_seg006_a3,Longitudinal_Strain_Rate_Endo_seg006_a4))

Longitudinal_Strain_Rate_Endo_seg006_a_max = max(c(Longitudinal_Strain_Rate_Endo_seg006_a1,Longitudinal_Strain_Rate_Endo_seg006_a2,Longitudinal_Strain_Rate_Endo_seg006_a3,Longitudinal_Strain_Rate_Endo_seg006_a4))

##Average_values_per_frame_Longitudinal_Strain_Rate_Endo

Average_values_per_frame_Longitudinal_Strain_Rate_Endo_point1 = as.numeric(Strain_Rate_data[30,col1])

Average_values_per_frame_Longitudinal_Strain_Rate_Endo_point2 = as.numeric(Strain_Rate_data[30,col2])

Average_values_per_frame_Longitudinal_Strain_Rate_Endo_point3 = as.numeric(Strain_Rate_data[30,col3])

Average_values_per_frame_Longitudinal_Strain_Rate_Endo_point4 = as.numeric(Strain_Rate_data[30,col4])

Average_values_per_frame_Longitudinal_Strain_Rate_Endo_point5 = as.numeric(Strain_Rate_data[30,col5])

Average_values_per_frame_Longitudinal_Strain_Rate_Endo_a1 = (Average_values_per_frame_Longitudinal_Strain_Rate_Endo_point2 - Average_values_per_frame_Longitudinal_Strain_Rate_Endo_point1)/Time_duration1

Average_values_per_frame_Longitudinal_Strain_Rate_Endo_a2 = (Average_values_per_frame_Longitudinal_Strain_Rate_Endo_point3 - Average_values_per_frame_Longitudinal_Strain_Rate_Endo_point2)/Time_duration2

Average_values_per_frame_Longitudinal_Strain_Rate_Endo_a3 = (Average_values_per_frame_Longitudinal_Strain_Rate_Endo_point4 - Average_values_per_frame_Longitudinal_Strain_Rate_Endo_point3)/Time_duration3

Average_values_per_frame_Longitudinal_Strain_Rate_Endo_a4 = (Average_values_per_frame_Longitudinal_Strain_Rate_Endo_point5 - Average_values_per_frame_Longitudinal_Strain_Rate_Endo_point4)/Time_duration4

Average_values_per_frame_Longitudinal_Strain_Rate_Endo_a_min = min(c(Average_values_per_frame_Longitudinal_Strain_Rate_Endo_a1,Average_values_per_frame_Longitudinal_Strain_Rate_Endo_a2,Average_values_per_frame_Longitudinal_Strain_Rate_Endo_a3,Average_values_per_frame_Longitudinal_Strain_Rate_Endo_a4))

Average_values_per_frame_Longitudinal_Strain_Rate_Endo_a_max = max(c(Average_values_per_frame_Longitudinal_Strain_Rate_Endo_a1,Average_values_per_frame_Longitudinal_Strain_Rate_Endo_a2,Average_values_per_frame_Longitudinal_Strain_Rate_Endo_a3,Average_values_per_frame_Longitudinal_Strain_Rate_Endo_a4))

##Radial_Strain_Rate_Epi

Radial_Strain_Rate_Epi_seg001_point1 = as.numeric(Strain_Rate_data[34,col1])

Radial_Strain_Rate_Epi_seg001_point2 = as.numeric(Strain_Rate_data[34,col2])

Radial_Strain_Rate_Epi_seg001_point3 = as.numeric(Strain_Rate_data[34,col3])

Radial_Strain_Rate_Epi_seg001_point4 = as.numeric(Strain_Rate_data[34,col4])

Radial_Strain_Rate_Epi_seg001_point5 = as.numeric(Strain_Rate_data[34,col5])

Radial_Strain_Rate_Epi_seg001_a1 = (Radial_Strain_Rate_Epi_seg001_point2 - Radial_Strain_Rate_Epi_seg001_point1)/Time_duration1

Radial_Strain_Rate_Epi_seg001_a2 = (Radial_Strain_Rate_Epi_seg001_point3 - Radial_Strain_Rate_Epi_seg001_point2)/Time_duration2

Radial_Strain_Rate_Epi_seg001_a3 = (Radial_Strain_Rate_Epi_seg001_point4 - Radial_Strain_Rate_Epi_seg001_point3)/Time_duration3

Radial_Strain_Rate_Epi_seg001_a4 = (Radial_Strain_Rate_Epi_seg001_point5 - Radial_Strain_Rate_Epi_seg001_point4)/Time_duration4

Radial_Strain_Rate_Epi_seg001_a_min = min(c(Radial_Strain_Rate_Epi_seg001_a1,Radial_Strain_Rate_Epi_seg001_a2,Radial_Strain_Rate_Epi_seg001_a3,Radial_Strain_Rate_Epi_seg001_a4))

Radial_Strain_Rate_Epi_seg001_a_max = max(c(Radial_Strain_Rate_Epi_seg001_a1,Radial_Strain_Rate_Epi_seg001_a2,Radial_Strain_Rate_Epi_seg001_a3,Radial_Strain_Rate_Epi_seg001_a4))

Radial_Strain_Rate_Epi_seg002_point1 = as.numeric(Strain_Rate_data[35,col1])

Radial_Strain_Rate_Epi_seg002_point2 = as.numeric(Strain_Rate_data[35,col2])

Radial_Strain_Rate_Epi_seg002_point3 = as.numeric(Strain_Rate_data[35,col3])

Radial_Strain_Rate_Epi_seg002_point4 = as.numeric(Strain_Rate_data[35,col4])

Radial_Strain_Rate_Epi_seg002_point5 = as.numeric(Strain_Rate_data[35,col5])

Radial_Strain_Rate_Epi_seg002_a1 = (Radial_Strain_Rate_Epi_seg002_point2 - Radial_Strain_Rate_Epi_seg002_point1)/Time_duration1

Radial_Strain_Rate_Epi_seg002_a2 = (Radial_Strain_Rate_Epi_seg002_point3 - Radial_Strain_Rate_Epi_seg002_point2)/Time_duration2

Radial_Strain_Rate_Epi_seg002_a3 = (Radial_Strain_Rate_Epi_seg002_point4 - Radial_Strain_Rate_Epi_seg002_point3)/Time_duration3

Radial_Strain_Rate_Epi_seg002_a4 = (Radial_Strain_Rate_Epi_seg002_point5 - Radial_Strain_Rate_Epi_seg002_point4)/Time_duration4

Radial_Strain_Rate_Epi_seg002_a_min = min(c(Radial_Strain_Rate_Epi_seg002_a1,Radial_Strain_Rate_Epi_seg002_a2,Radial_Strain_Rate_Epi_seg002_a3,Radial_Strain_Rate_Epi_seg002_a4))

Radial_Strain_Rate_Epi_seg002_a_max = max(c(Radial_Strain_Rate_Epi_seg002_a1,Radial_Strain_Rate_Epi_seg002_a2,Radial_Strain_Rate_Epi_seg002_a3,Radial_Strain_Rate_Epi_seg002_a4))

Radial_Strain_Rate_Epi_seg003_point1 = as.numeric(Strain_Rate_data[36,col1])

Radial_Strain_Rate_Epi_seg003_point2 = as.numeric(Strain_Rate_data[36,col2])

Radial_Strain_Rate_Epi_seg003_point3 = as.numeric(Strain_Rate_data[36,col3])

Radial_Strain_Rate_Epi_seg003_point4 = as.numeric(Strain_Rate_data[36,col4])

Radial_Strain_Rate_Epi_seg003_point5 = as.numeric(Strain_Rate_data[36,col5])

Radial_Strain_Rate_Epi_seg003_a1 = (Radial_Strain_Rate_Epi_seg003_point2 - Radial_Strain_Rate_Epi_seg003_point1)/Time_duration1

Radial_Strain_Rate_Epi_seg003_a2 = (Radial_Strain_Rate_Epi_seg003_point3 - Radial_Strain_Rate_Epi_seg003_point2)/Time_duration2

Radial_Strain_Rate_Epi_seg003_a3 = (Radial_Strain_Rate_Epi_seg003_point4 - Radial_Strain_Rate_Epi_seg003_point3)/Time_duration3

Radial_Strain_Rate_Epi_seg003_a4 = (Radial_Strain_Rate_Epi_seg003_point5 - Radial_Strain_Rate_Epi_seg003_point4)/Time_duration4

Radial_Strain_Rate_Epi_seg003_a_min = min(c(Radial_Strain_Rate_Epi_seg003_a1,Radial_Strain_Rate_Epi_seg003_a2,Radial_Strain_Rate_Epi_seg003_a3,Radial_Strain_Rate_Epi_seg003_a4))

Radial_Strain_Rate_Epi_seg003_a_max = max(c(Radial_Strain_Rate_Epi_seg003_a1,Radial_Strain_Rate_Epi_seg003_a2,Radial_Strain_Rate_Epi_seg003_a3,Radial_Strain_Rate_Epi_seg003_a4))

Radial_Strain_Rate_Epi_seg004_point1 = as.numeric(Strain_Rate_data[37,col1])

Radial_Strain_Rate_Epi_seg004_point2 = as.numeric(Strain_Rate_data[37,col2])

Radial_Strain_Rate_Epi_seg004_point3 = as.numeric(Strain_Rate_data[37,col3])

Radial_Strain_Rate_Epi_seg004_point4 = as.numeric(Strain_Rate_data[37,col4])

Radial_Strain_Rate_Epi_seg004_point5 = as.numeric(Strain_Rate_data[37,col5])

Radial_Strain_Rate_Epi_seg004_a1 = (Radial_Strain_Rate_Epi_seg004_point2 - Radial_Strain_Rate_Epi_seg004_point1)/Time_duration1

Radial_Strain_Rate_Epi_seg004_a2 = (Radial_Strain_Rate_Epi_seg004_point3 - Radial_Strain_Rate_Epi_seg004_point2)/Time_duration2

Radial_Strain_Rate_Epi_seg004_a3 = (Radial_Strain_Rate_Epi_seg004_point4 - Radial_Strain_Rate_Epi_seg004_point3)/Time_duration3

Radial_Strain_Rate_Epi_seg004_a4 = (Radial_Strain_Rate_Epi_seg004_point5 - Radial_Strain_Rate_Epi_seg004_point4)/Time_duration4

Radial_Strain_Rate_Epi_seg004_a_min = min(c(Radial_Strain_Rate_Epi_seg004_a1,Radial_Strain_Rate_Epi_seg004_a2,Radial_Strain_Rate_Epi_seg004_a3,Radial_Strain_Rate_Epi_seg004_a4))

Radial_Strain_Rate_Epi_seg004_a_max = max(c(Radial_Strain_Rate_Epi_seg004_a1,Radial_Strain_Rate_Epi_seg004_a2,Radial_Strain_Rate_Epi_seg004_a3,Radial_Strain_Rate_Epi_seg004_a4))

Radial_Strain_Rate_Epi_seg005_point1 = as.numeric(Strain_Rate_data[38,col1])

Radial_Strain_Rate_Epi_seg005_point2 = as.numeric(Strain_Rate_data[38,col2])

Radial_Strain_Rate_Epi_seg005_point3 = as.numeric(Strain_Rate_data[38,col3])

Radial_Strain_Rate_Epi_seg005_point4 = as.numeric(Strain_Rate_data[38,col4])

Radial_Strain_Rate_Epi_seg005_point5 = as.numeric(Strain_Rate_data[38,col5])

Radial_Strain_Rate_Epi_seg005_a1 = (Radial_Strain_Rate_Epi_seg005_point2 - Radial_Strain_Rate_Epi_seg005_point1)/Time_duration1

Radial_Strain_Rate_Epi_seg005_a2 = (Radial_Strain_Rate_Epi_seg005_point3 - Radial_Strain_Rate_Epi_seg005_point2)/Time_duration2

Radial_Strain_Rate_Epi_seg005_a3 = (Radial_Strain_Rate_Epi_seg005_point4 - Radial_Strain_Rate_Epi_seg005_point3)/Time_duration3

Radial_Strain_Rate_Epi_seg005_a4 = (Radial_Strain_Rate_Epi_seg005_point5 - Radial_Strain_Rate_Epi_seg005_point4)/Time_duration4

Radial_Strain_Rate_Epi_seg005_a_min = min(c(Radial_Strain_Rate_Epi_seg005_a1,Radial_Strain_Rate_Epi_seg005_a2,Radial_Strain_Rate_Epi_seg005_a3,Radial_Strain_Rate_Epi_seg005_a4))

Radial_Strain_Rate_Epi_seg005_a_max = max(c(Radial_Strain_Rate_Epi_seg005_a1,Radial_Strain_Rate_Epi_seg005_a2,Radial_Strain_Rate_Epi_seg005_a3,Radial_Strain_Rate_Epi_seg005_a4))

Radial_Strain_Rate_Epi_seg006_point1 = as.numeric(Strain_Rate_data[39,col1])

Radial_Strain_Rate_Epi_seg006_point2 = as.numeric(Strain_Rate_data[39,col2])

Radial_Strain_Rate_Epi_seg006_point3 = as.numeric(Strain_Rate_data[39,col3])

Radial_Strain_Rate_Epi_seg006_point4 = as.numeric(Strain_Rate_data[39,col4])

Radial_Strain_Rate_Epi_seg006_point5 = as.numeric(Strain_Rate_data[39,col5])

Radial_Strain_Rate_Epi_seg006_a1 = (Radial_Strain_Rate_Epi_seg006_point2 - Radial_Strain_Rate_Epi_seg006_point1)/Time_duration1

Radial_Strain_Rate_Epi_seg006_a2 = (Radial_Strain_Rate_Epi_seg006_point3 - Radial_Strain_Rate_Epi_seg006_point2)/Time_duration2

Radial_Strain_Rate_Epi_seg006_a3 = (Radial_Strain_Rate_Epi_seg006_point4 - Radial_Strain_Rate_Epi_seg006_point3)/Time_duration3

Radial_Strain_Rate_Epi_seg006_a4 = (Radial_Strain_Rate_Epi_seg006_point5 - Radial_Strain_Rate_Epi_seg006_point4)/Time_duration4

Radial_Strain_Rate_Epi_seg006_a_min = min(c(Radial_Strain_Rate_Epi_seg006_a1,Radial_Strain_Rate_Epi_seg006_a2,Radial_Strain_Rate_Epi_seg006_a3,Radial_Strain_Rate_Epi_seg006_a4))

Radial_Strain_Rate_Epi_seg006_a_max = max(c(Radial_Strain_Rate_Epi_seg006_a1,Radial_Strain_Rate_Epi_seg006_a2,Radial_Strain_Rate_Epi_seg006_a3,Radial_Strain_Rate_Epi_seg006_a4))

##Average_values_per_frame_Radial_Strain_Rate_Epi

Average_values_per_frame_Radial_Strain_Rate_Epi_point1 = as.numeric(Strain_Rate_data[43,col1])

Average_values_per_frame_Radial_Strain_Rate_Epi_point2 = as.numeric(Strain_Rate_data[43,col2])

Average_values_per_frame_Radial_Strain_Rate_Epi_point3 = as.numeric(Strain_Rate_data[43,col3])

Average_values_per_frame_Radial_Strain_Rate_Epi_point4 = as.numeric(Strain_Rate_data[43,col4])

Average_values_per_frame_Radial_Strain_Rate_Epi_point5 = as.numeric(Strain_Rate_data[43,col5])

Average_values_per_frame_Radial_Strain_Rate_Epi_a1 = (Average_values_per_frame_Radial_Strain_Rate_Epi_point2 - Average_values_per_frame_Radial_Strain_Rate_Epi_point1)/Time_duration1

Average_values_per_frame_Radial_Strain_Rate_Epi_a2 = (Average_values_per_frame_Radial_Strain_Rate_Epi_point3 - Average_values_per_frame_Radial_Strain_Rate_Epi_point2)/Time_duration2

Average_values_per_frame_Radial_Strain_Rate_Epi_a3 = (Average_values_per_frame_Radial_Strain_Rate_Epi_point4 - Average_values_per_frame_Radial_Strain_Rate_Epi_point3)/Time_duration3

Average_values_per_frame_Radial_Strain_Rate_Epi_a4 = (Average_values_per_frame_Radial_Strain_Rate_Epi_point5 - Average_values_per_frame_Radial_Strain_Rate_Epi_point4)/Time_duration4

Average_values_per_frame_Radial_Strain_Rate_Epi_a_min = min(c(Average_values_per_frame_Radial_Strain_Rate_Epi_a1,Average_values_per_frame_Radial_Strain_Rate_Epi_a2,Average_values_per_frame_Radial_Strain_Rate_Epi_a3,Average_values_per_frame_Radial_Strain_Rate_Epi_a4))

Average_values_per_frame_Radial_Strain_Rate_Epi_a_max = max(c(Average_values_per_frame_Radial_Strain_Rate_Epi_a1,Average_values_per_frame_Radial_Strain_Rate_Epi_a2,Average_values_per_frame_Radial_Strain_Rate_Epi_a3,Average_values_per_frame_Radial_Strain_Rate_Epi_a4))

##Longitudinal_Strain_Rate_Epi

Longitudinal_Strain_Rate_Epi_seg001_point1 = as.numeric(Strain_Rate_data[47,col1])

Longitudinal_Strain_Rate_Epi_seg001_point2 = as.numeric(Strain_Rate_data[47,col2])

Longitudinal_Strain_Rate_Epi_seg001_point3 = as.numeric(Strain_Rate_data[47,col3])

Longitudinal_Strain_Rate_Epi_seg001_point4 = as.numeric(Strain_Rate_data[47,col4])

Longitudinal_Strain_Rate_Epi_seg001_point5 = as.numeric(Strain_Rate_data[47,col5])

Longitudinal_Strain_Rate_Epi_seg001_a1 = (Longitudinal_Strain_Rate_Epi_seg001_point2 - Longitudinal_Strain_Rate_Epi_seg001_point1)/Time_duration1

Longitudinal_Strain_Rate_Epi_seg001_a2 = (Longitudinal_Strain_Rate_Epi_seg001_point3 - Longitudinal_Strain_Rate_Epi_seg001_point2)/Time_duration2

Longitudinal_Strain_Rate_Epi_seg001_a3 = (Longitudinal_Strain_Rate_Epi_seg001_point4 - Longitudinal_Strain_Rate_Epi_seg001_point3)/Time_duration3

Longitudinal_Strain_Rate_Epi_seg001_a4 = (Longitudinal_Strain_Rate_Epi_seg001_point5 - Longitudinal_Strain_Rate_Epi_seg001_point4)/Time_duration4

Longitudinal_Strain_Rate_Epi_seg001_a_min = min(c(Longitudinal_Strain_Rate_Epi_seg001_a1,Longitudinal_Strain_Rate_Epi_seg001_a2,Longitudinal_Strain_Rate_Epi_seg001_a3,Longitudinal_Strain_Rate_Epi_seg001_a4))

Longitudinal_Strain_Rate_Epi_seg001_a_max = max(c(Longitudinal_Strain_Rate_Epi_seg001_a1,Longitudinal_Strain_Rate_Epi_seg001_a2,Longitudinal_Strain_Rate_Epi_seg001_a3,Longitudinal_Strain_Rate_Epi_seg001_a4))

Longitudinal_Strain_Rate_Epi_seg002_point1 = as.numeric(Strain_Rate_data[48,col1])

Longitudinal_Strain_Rate_Epi_seg002_point2 = as.numeric(Strain_Rate_data[48,col2])

Longitudinal_Strain_Rate_Epi_seg002_point3 = as.numeric(Strain_Rate_data[48,col3])

Longitudinal_Strain_Rate_Epi_seg002_point4 = as.numeric(Strain_Rate_data[48,col4])

Longitudinal_Strain_Rate_Epi_seg002_point5 = as.numeric(Strain_Rate_data[48,col5])

Longitudinal_Strain_Rate_Epi_seg002_a1 = (Longitudinal_Strain_Rate_Epi_seg002_point2 - Longitudinal_Strain_Rate_Epi_seg002_point1)/Time_duration1

Longitudinal_Strain_Rate_Epi_seg002_a2 = (Longitudinal_Strain_Rate_Epi_seg002_point3 - Longitudinal_Strain_Rate_Epi_seg002_point2)/Time_duration2

Longitudinal_Strain_Rate_Epi_seg002_a3 = (Longitudinal_Strain_Rate_Epi_seg002_point4 - Longitudinal_Strain_Rate_Epi_seg002_point3)/Time_duration3

Longitudinal_Strain_Rate_Epi_seg002_a4 = (Longitudinal_Strain_Rate_Epi_seg002_point5 - Longitudinal_Strain_Rate_Epi_seg002_point4)/Time_duration4

Longitudinal_Strain_Rate_Epi_seg002_a_min = min(c(Longitudinal_Strain_Rate_Epi_seg002_a1,Longitudinal_Strain_Rate_Epi_seg002_a2,Longitudinal_Strain_Rate_Epi_seg002_a3,Longitudinal_Strain_Rate_Epi_seg002_a4))

Longitudinal_Strain_Rate_Epi_seg002_a_max = max(c(Longitudinal_Strain_Rate_Epi_seg002_a1,Longitudinal_Strain_Rate_Epi_seg002_a2,Longitudinal_Strain_Rate_Epi_seg002_a3,Longitudinal_Strain_Rate_Epi_seg002_a4))

Longitudinal_Strain_Rate_Epi_seg003_point1 = as.numeric(Strain_Rate_data[49,col1])

Longitudinal_Strain_Rate_Epi_seg003_point2 = as.numeric(Strain_Rate_data[49,col2])

Longitudinal_Strain_Rate_Epi_seg003_point3 = as.numeric(Strain_Rate_data[49,col3])

Longitudinal_Strain_Rate_Epi_seg003_point4 = as.numeric(Strain_Rate_data[49,col4])

Longitudinal_Strain_Rate_Epi_seg003_point5 = as.numeric(Strain_Rate_data[49,col5])

Longitudinal_Strain_Rate_Epi_seg003_a1 = (Longitudinal_Strain_Rate_Epi_seg003_point2 - Longitudinal_Strain_Rate_Epi_seg003_point1)/Time_duration1

Longitudinal_Strain_Rate_Epi_seg003_a2 = (Longitudinal_Strain_Rate_Epi_seg003_point3 - Longitudinal_Strain_Rate_Epi_seg003_point2)/Time_duration2

Longitudinal_Strain_Rate_Epi_seg003_a3 = (Longitudinal_Strain_Rate_Epi_seg003_point4 - Longitudinal_Strain_Rate_Epi_seg003_point3)/Time_duration3

Longitudinal_Strain_Rate_Epi_seg003_a4 = (Longitudinal_Strain_Rate_Epi_seg003_point5 - Longitudinal_Strain_Rate_Epi_seg003_point4)/Time_duration4

Longitudinal_Strain_Rate_Epi_seg003_a_min = min(c(Longitudinal_Strain_Rate_Epi_seg003_a1,Longitudinal_Strain_Rate_Epi_seg003_a2,Longitudinal_Strain_Rate_Epi_seg003_a3,Longitudinal_Strain_Rate_Epi_seg003_a4))

Longitudinal_Strain_Rate_Epi_seg003_a_max = max(c(Longitudinal_Strain_Rate_Epi_seg003_a1,Longitudinal_Strain_Rate_Epi_seg003_a2,Longitudinal_Strain_Rate_Epi_seg003_a3,Longitudinal_Strain_Rate_Epi_seg003_a4))

Longitudinal_Strain_Rate_Epi_seg004_point1 = as.numeric(Strain_Rate_data[50,col1])

Longitudinal_Strain_Rate_Epi_seg004_point2 = as.numeric(Strain_Rate_data[50,col2])

Longitudinal_Strain_Rate_Epi_seg004_point3 = as.numeric(Strain_Rate_data[50,col3])

Longitudinal_Strain_Rate_Epi_seg004_point4 = as.numeric(Strain_Rate_data[50,col4])

Longitudinal_Strain_Rate_Epi_seg004_point5 = as.numeric(Strain_Rate_data[50,col5])

Longitudinal_Strain_Rate_Epi_seg004_a1 = (Longitudinal_Strain_Rate_Epi_seg004_point2 - Longitudinal_Strain_Rate_Epi_seg004_point1)/Time_duration1

Longitudinal_Strain_Rate_Epi_seg004_a2 = (Longitudinal_Strain_Rate_Epi_seg004_point3 - Longitudinal_Strain_Rate_Epi_seg004_point2)/Time_duration2

Longitudinal_Strain_Rate_Epi_seg004_a3 = (Longitudinal_Strain_Rate_Epi_seg004_point4 - Longitudinal_Strain_Rate_Epi_seg004_point3)/Time_duration3

Longitudinal_Strain_Rate_Epi_seg004_a4 = (Longitudinal_Strain_Rate_Epi_seg004_point5 - Longitudinal_Strain_Rate_Epi_seg004_point4)/Time_duration4

Longitudinal_Strain_Rate_Epi_seg004_a_min = min(c(Longitudinal_Strain_Rate_Epi_seg004_a1,Longitudinal_Strain_Rate_Epi_seg004_a2,Longitudinal_Strain_Rate_Epi_seg004_a3,Longitudinal_Strain_Rate_Epi_seg004_a4))

Longitudinal_Strain_Rate_Epi_seg004_a_max = max(c(Longitudinal_Strain_Rate_Epi_seg004_a1,Longitudinal_Strain_Rate_Epi_seg004_a2,Longitudinal_Strain_Rate_Epi_seg004_a3,Longitudinal_Strain_Rate_Epi_seg004_a4))

Longitudinal_Strain_Rate_Epi_seg005_point1 = as.numeric(Strain_Rate_data[51,col1])

Longitudinal_Strain_Rate_Epi_seg005_point2 = as.numeric(Strain_Rate_data[51,col2])

Longitudinal_Strain_Rate_Epi_seg005_point3 = as.numeric(Strain_Rate_data[51,col3])

Longitudinal_Strain_Rate_Epi_seg005_point4 = as.numeric(Strain_Rate_data[51,col4])

Longitudinal_Strain_Rate_Epi_seg005_point5 = as.numeric(Strain_Rate_data[51,col5])

Longitudinal_Strain_Rate_Epi_seg005_a1 = (Longitudinal_Strain_Rate_Epi_seg005_point2 - Longitudinal_Strain_Rate_Epi_seg005_point1)/Time_duration1

Longitudinal_Strain_Rate_Epi_seg005_a2 = (Longitudinal_Strain_Rate_Epi_seg005_point3 - Longitudinal_Strain_Rate_Epi_seg005_point2)/Time_duration2

Longitudinal_Strain_Rate_Epi_seg005_a3 = (Longitudinal_Strain_Rate_Epi_seg005_point4 - Longitudinal_Strain_Rate_Epi_seg005_point3)/Time_duration3

Longitudinal_Strain_Rate_Epi_seg005_a4 = (Longitudinal_Strain_Rate_Epi_seg005_point5 - Longitudinal_Strain_Rate_Epi_seg005_point4)/Time_duration4

Longitudinal_Strain_Rate_Epi_seg005_a_min = min(c(Longitudinal_Strain_Rate_Epi_seg005_a1,Longitudinal_Strain_Rate_Epi_seg005_a2,Longitudinal_Strain_Rate_Epi_seg005_a3,Longitudinal_Strain_Rate_Epi_seg005_a4))

Longitudinal_Strain_Rate_Epi_seg005_a_max = max(c(Longitudinal_Strain_Rate_Epi_seg005_a1,Longitudinal_Strain_Rate_Epi_seg005_a2,Longitudinal_Strain_Rate_Epi_seg005_a3,Longitudinal_Strain_Rate_Epi_seg005_a4))

Longitudinal_Strain_Rate_Epi_seg006_point1 = as.numeric(Strain_Rate_data[52,col1])

Longitudinal_Strain_Rate_Epi_seg006_point2 = as.numeric(Strain_Rate_data[52,col2])

Longitudinal_Strain_Rate_Epi_seg006_point3 = as.numeric(Strain_Rate_data[52,col3])

Longitudinal_Strain_Rate_Epi_seg006_point4 = as.numeric(Strain_Rate_data[52,col4])

Longitudinal_Strain_Rate_Epi_seg006_point5 = as.numeric(Strain_Rate_data[52,col5])

Longitudinal_Strain_Rate_Epi_seg006_a1 = (Longitudinal_Strain_Rate_Epi_seg006_point2 - Longitudinal_Strain_Rate_Epi_seg006_point1)/Time_duration1

Longitudinal_Strain_Rate_Epi_seg006_a2 = (Longitudinal_Strain_Rate_Epi_seg006_point3 - Longitudinal_Strain_Rate_Epi_seg006_point2)/Time_duration2

Longitudinal_Strain_Rate_Epi_seg006_a3 = (Longitudinal_Strain_Rate_Epi_seg006_point4 - Longitudinal_Strain_Rate_Epi_seg006_point3)/Time_duration3

Longitudinal_Strain_Rate_Epi_seg006_a4 = (Longitudinal_Strain_Rate_Epi_seg006_point5 - Longitudinal_Strain_Rate_Epi_seg006_point4)/Time_duration4

Longitudinal_Strain_Rate_Epi_seg006_a_min = min(c(Longitudinal_Strain_Rate_Epi_seg006_a1,Longitudinal_Strain_Rate_Epi_seg006_a2,Longitudinal_Strain_Rate_Epi_seg006_a3,Longitudinal_Strain_Rate_Epi_seg006_a4))

Longitudinal_Strain_Rate_Epi_seg006_a_max = max(c(Longitudinal_Strain_Rate_Epi_seg006_a1,Longitudinal_Strain_Rate_Epi_seg006_a2,Longitudinal_Strain_Rate_Epi_seg006_a3,Longitudinal_Strain_Rate_Epi_seg006_a4))

##Average_values_per_frame_Longitudinal_Strain_Rate_Epi

Average_values_per_frame_Longitudinal_Strain_Rate_Epi_point1 = as.numeric(Strain_Rate_data[56,col1])

Average_values_per_frame_Longitudinal_Strain_Rate_Epi_point2 = as.numeric(Strain_Rate_data[56,col2])

Average_values_per_frame_Longitudinal_Strain_Rate_Epi_point3 = as.numeric(Strain_Rate_data[56,col3])

Average_values_per_frame_Longitudinal_Strain_Rate_Epi_point4 = as.numeric(Strain_Rate_data[56,col4])

Average_values_per_frame_Longitudinal_Strain_Rate_Epi_point5 = as.numeric(Strain_Rate_data[56,col5])

Average_values_per_frame_Longitudinal_Strain_Rate_Epi_a1 = (Average_values_per_frame_Longitudinal_Strain_Rate_Epi_point2 - Average_values_per_frame_Longitudinal_Strain_Rate_Epi_point1)/Time_duration1

Average_values_per_frame_Longitudinal_Strain_Rate_Epi_a2 = (Average_values_per_frame_Longitudinal_Strain_Rate_Epi_point3 - Average_values_per_frame_Longitudinal_Strain_Rate_Epi_point2)/Time_duration2

Average_values_per_frame_Longitudinal_Strain_Rate_Epi_a3 = (Average_values_per_frame_Longitudinal_Strain_Rate_Epi_point4 - Average_values_per_frame_Longitudinal_Strain_Rate_Epi_point3)/Time_duration3

Average_values_per_frame_Longitudinal_Strain_Rate_Epi_a4 = (Average_values_per_frame_Longitudinal_Strain_Rate_Epi_point5 - Average_values_per_frame_Longitudinal_Strain_Rate_Epi_point4)/Time_duration4

Average_values_per_frame_Longitudinal_Strain_Rate_Epi_a_min = min(c(Average_values_per_frame_Longitudinal_Strain_Rate_Epi_a1,Average_values_per_frame_Longitudinal_Strain_Rate_Epi_a2,Average_values_per_frame_Longitudinal_Strain_Rate_Epi_a3,Average_values_per_frame_Longitudinal_Strain_Rate_Epi_a4))

Average_values_per_frame_Longitudinal_Strain_Rate_Epi_a_max = max(c(Average_values_per_frame_Longitudinal_Strain_Rate_Epi_a1,Average_values_per_frame_Longitudinal_Strain_Rate_Epi_a2,Average_values_per_frame_Longitudinal_Strain_Rate_Epi_a3,Average_values_per_frame_Longitudinal_Strain_Rate_Epi_a4))

#Shear

Shear_data <- read.xlsx(i,sheetIndex = 6, header = F)

colnames(Shear_data) <- Shear_data[3,]

name1 = paste("fr0",time3,sep = "")

col5 = grep(name1,colnames(Shear_data))

col4 = col5-1

col3 = col5-2

col2 = col5-3

col1 = col5-4

Timepoint1 = as.numeric(Shear_data[4,col1])

Timepoint2 = as.numeric(Shear_data[4,col2])

Timepoint3 = as.numeric(Shear_data[4,col3])

Timepoint4 = as.numeric(Shear_data[4,col4])

Timepoint5 = as.numeric(Shear_data[4,col5])

Time_duration1 = Timepoint2 - Timepoint1

Time_duration2 = Timepoint3 - Timepoint2

Time_duration3 = Timepoint4 - Timepoint3

Time_duration4 = Timepoint5 - Timepoint4

##Shear

Shear_seg001_point1 = as.numeric(Shear_data[8,col1])

Shear_seg001_point2 = as.numeric(Shear_data[8,col2])

Shear_seg001_point3 = as.numeric(Shear_data[8,col3])

Shear_seg001_point4 = as.numeric(Shear_data[8,col4])

Shear_seg001_point5 = as.numeric(Shear_data[8,col5])

Shear_seg001_a1 = (Shear_seg001_point2 - Shear_seg001_point1)/Time_duration1

Shear_seg001_a2 = (Shear_seg001_point3 - Shear_seg001_point2)/Time_duration2

Shear_seg001_a3 = (Shear_seg001_point4 - Shear_seg001_point3)/Time_duration3

Shear_seg001_a4 = (Shear_seg001_point5 - Shear_seg001_point4)/Time_duration4

Shear_seg001_a_min = min(c(Shear_seg001_a1,Shear_seg001_a2,Shear_seg001_a3,Shear_seg001_a4))

Shear_seg001_a_max = max(c(Shear_seg001_a1,Shear_seg001_a2,Shear_seg001_a3,Shear_seg001_a4))

Shear_seg002_point1 = as.numeric(Shear_data[9,col1])

Shear_seg002_point2 = as.numeric(Shear_data[9,col2])

Shear_seg002_point3 = as.numeric(Shear_data[9,col3])

Shear_seg002_point4 = as.numeric(Shear_data[9,col4])

Shear_seg002_point5 = as.numeric(Shear_data[9,col5])

Shear_seg002_a1 = (Shear_seg002_point2 - Shear_seg002_point1)/Time_duration1

Shear_seg002_a2 = (Shear_seg002_point3 - Shear_seg002_point2)/Time_duration2

Shear_seg002_a3 = (Shear_seg002_point4 - Shear_seg002_point3)/Time_duration3

Shear_seg002_a4 = (Shear_seg002_point5 - Shear_seg002_point4)/Time_duration4

Shear_seg002_a_min = min(c(Shear_seg002_a1,Shear_seg002_a2,Shear_seg002_a3,Shear_seg002_a4))

Shear_seg002_a_max = max(c(Shear_seg002_a1,Shear_seg002_a2,Shear_seg002_a3,Shear_seg002_a4))

Shear_seg003_point1 = as.numeric(Shear_data[10,col1])

Shear_seg003_point2 = as.numeric(Shear_data[10,col2])

Shear_seg003_point3 = as.numeric(Shear_data[10,col3])

Shear_seg003_point4 = as.numeric(Shear_data[10,col4])

Shear_seg003_point5 = as.numeric(Shear_data[10,col5])

Shear_seg003_a1 = (Shear_seg003_point2 - Shear_seg003_point1)/Time_duration1

Shear_seg003_a2 = (Shear_seg003_point3 - Shear_seg003_point2)/Time_duration2

Shear_seg003_a3 = (Shear_seg003_point4 - Shear_seg003_point3)/Time_duration3

Shear_seg003_a4 = (Shear_seg003_point5 - Shear_seg003_point4)/Time_duration4

Shear_seg003_a_min = min(c(Shear_seg003_a1,Shear_seg003_a2,Shear_seg003_a3,Shear_seg003_a4))

Shear_seg003_a_max = max(c(Shear_seg003_a1,Shear_seg003_a2,Shear_seg003_a3,Shear_seg003_a4))

Shear_seg004_point1 = as.numeric(Shear_data[11,col1])

Shear_seg004_point2 = as.numeric(Shear_data[11,col2])

Shear_seg004_point3 = as.numeric(Shear_data[11,col3])

Shear_seg004_point4 = as.numeric(Shear_data[11,col4])

Shear_seg004_point5 = as.numeric(Shear_data[11,col5])

Shear_seg004_a1 = (Shear_seg004_point2 - Shear_seg004_point1)/Time_duration1

Shear_seg004_a2 = (Shear_seg004_point3 - Shear_seg004_point2)/Time_duration2

Shear_seg004_a3 = (Shear_seg004_point4 - Shear_seg004_point3)/Time_duration3

Shear_seg004_a4 = (Shear_seg004_point5 - Shear_seg004_point4)/Time_duration4

Shear_seg004_a_min = min(c(Shear_seg004_a1,Shear_seg004_a2,Shear_seg004_a3,Shear_seg004_a4))

Shear_seg004_a_max = max(c(Shear_seg004_a1,Shear_seg004_a2,Shear_seg004_a3,Shear_seg004_a4))

Shear_seg005_point1 = as.numeric(Shear_data[12,col1])

Shear_seg005_point2 = as.numeric(Shear_data[12,col2])

Shear_seg005_point3 = as.numeric(Shear_data[12,col3])

Shear_seg005_point4 = as.numeric(Shear_data[12,col4])

Shear_seg005_point5 = as.numeric(Shear_data[12,col5])

Shear_seg005_a1 = (Shear_seg005_point2 - Shear_seg005_point1)/Time_duration1

Shear_seg005_a2 = (Shear_seg005_point3 - Shear_seg005_point2)/Time_duration2

Shear_seg005_a3 = (Shear_seg005_point4 - Shear_seg005_point3)/Time_duration3

Shear_seg005_a4 = (Shear_seg005_point5 - Shear_seg005_point4)/Time_duration4

Shear_seg005_a_min = min(c(Shear_seg005_a1,Shear_seg005_a2,Shear_seg005_a3,Shear_seg005_a4))

Shear_seg005_a_max = max(c(Shear_seg005_a1,Shear_seg005_a2,Shear_seg005_a3,Shear_seg005_a4))

Shear_seg006_point1 = as.numeric(Shear_data[13,col1])

Shear_seg006_point2 = as.numeric(Shear_data[13,col2])

Shear_seg006_point3 = as.numeric(Shear_data[13,col3])

Shear_seg006_point4 = as.numeric(Shear_data[13,col4])

Shear_seg006_point5 = as.numeric(Shear_data[13,col5])

Shear_seg006_a1 = (Shear_seg006_point2 - Shear_seg006_point1)/Time_duration1

Shear_seg006_a2 = (Shear_seg006_point3 - Shear_seg006_point2)/Time_duration2

Shear_seg006_a3 = (Shear_seg006_point4 - Shear_seg006_point3)/Time_duration3

Shear_seg006_a4 = (Shear_seg006_point5 - Shear_seg006_point4)/Time_duration4

Shear_seg006_a_min = min(c(Shear_seg006_a1,Shear_seg006_a2,Shear_seg006_a3,Shear_seg006_a4))

Shear_seg006_a_max = max(c(Shear_seg006_a1,Shear_seg006_a2,Shear_seg006_a3,Shear_seg006_a4))

##Average_values_per_frame_Shear

Average_values_per_frame_Shear_point1 = as.numeric(Shear_data[17,col1])

Average_values_per_frame_Shear_point2 = as.numeric(Shear_data[17,col2])

Average_values_per_frame_Shear_point3 = as.numeric(Shear_data[17,col3])

Average_values_per_frame_Shear_point4 = as.numeric(Shear_data[17,col4])

Average_values_per_frame_Shear_point5 = as.numeric(Shear_data[17,col5])

Average_values_per_frame_Shear_a1 = (Average_values_per_frame_Shear_point2 - Average_values_per_frame_Shear_point1)/Time_duration1

Average_values_per_frame_Shear_a2 = (Average_values_per_frame_Shear_point3 - Average_values_per_frame_Shear_point2)/Time_duration2

Average_values_per_frame_Shear_a3 = (Average_values_per_frame_Shear_point4 - Average_values_per_frame_Shear_point3)/Time_duration3

Average_values_per_frame_Shear_a4 = (Average_values_per_frame_Shear_point5 - Average_values_per_frame_Shear_point4)/Time_duration4

Average_values_per_frame_Shear_a_min = min(c(Average_values_per_frame_Shear_a1,Average_values_per_frame_Shear_a2,Average_values_per_frame_Shear_a3,Average_values_per_frame_Shear_a4))

Average_values_per_frame_Shear_a_max = max(c(Average_values_per_frame_Shear_a1,Average_values_per_frame_Shear_a2,Average_values_per_frame_Shear_a3,Average_values_per_frame_Shear_a4))

##Shear_Rate

Shear_Rate_seg001_point1 = as.numeric(Shear_data[21,col1])

Shear_Rate_seg001_point2 = as.numeric(Shear_data[21,col2])

Shear_Rate_seg001_point3 = as.numeric(Shear_data[21,col3])

Shear_Rate_seg001_point4 = as.numeric(Shear_data[21,col4])

Shear_Rate_seg001_point5 = as.numeric(Shear_data[21,col5])

Shear_Rate_seg001_a1 = (Shear_Rate_seg001_point2 - Shear_Rate_seg001_point1)/Time_duration1

Shear_Rate_seg001_a2 = (Shear_Rate_seg001_point3 - Shear_Rate_seg001_point2)/Time_duration2

Shear_Rate_seg001_a3 = (Shear_Rate_seg001_point4 - Shear_Rate_seg001_point3)/Time_duration3

Shear_Rate_seg001_a4 = (Shear_Rate_seg001_point5 - Shear_Rate_seg001_point4)/Time_duration4

Shear_Rate_seg001_a_min = min(c(Shear_Rate_seg001_a1,Shear_Rate_seg001_a2,Shear_Rate_seg001_a3,Shear_Rate_seg001_a4))

Shear_Rate_seg001_a_max = max(c(Shear_Rate_seg001_a1,Shear_Rate_seg001_a2,Shear_Rate_seg001_a3,Shear_Rate_seg001_a4))

Shear_Rate_seg002_point1 = as.numeric(Shear_data[22,col1])

Shear_Rate_seg002_point2 = as.numeric(Shear_data[22,col2])

Shear_Rate_seg002_point3 = as.numeric(Shear_data[22,col3])

Shear_Rate_seg002_point4 = as.numeric(Shear_data[22,col4])

Shear_Rate_seg002_point5 = as.numeric(Shear_data[22,col5])

Shear_Rate_seg002_a1 = (Shear_Rate_seg002_point2 - Shear_Rate_seg002_point1)/Time_duration1

Shear_Rate_seg002_a2 = (Shear_Rate_seg002_point3 - Shear_Rate_seg002_point2)/Time_duration2

Shear_Rate_seg002_a3 = (Shear_Rate_seg002_point4 - Shear_Rate_seg002_point3)/Time_duration3

Shear_Rate_seg002_a4 = (Shear_Rate_seg002_point5 - Shear_Rate_seg002_point4)/Time_duration4
[truncated: 67,396 more chars]
